# Pro-Apoptotic Antitumoral Effect of Novel Acridine-Core Naphthoquinone Compounds against Oral Squamous Cell Carcinoma

**DOI:** 10.3390/molecules27165148

**Published:** 2022-08-12

**Authors:** Bruna Costa Zorzanelli, Gabriel Ouverney, Fernanda P. Pauli, Anna Carolina Carvalho da Fonseca, Elan Cardozo Paes de Almeida, Danielle Gonçalves de Carvalho, Patricia Abrão Possik, Vitor Won-Held Rabelo, Paula Alvarez Abreu, Bruno Pontes, Vitor Francisco Ferreira, Luana da Silva Magalhães Forezi, Fernando de Carvalho da Silva, Bruno Kaufmann Robbs

**Affiliations:** 1 Departamento de Química Orgânica, Instituto de Química, Campus do Valonguinho, Universidade Federal Fluminense, Niterói CEP 24020-141, Brazil; 2 Departamento de Ciência Básica, Campus Universitário de Nova Friburgo, Universidade Federal Fluminense, Nova Friburgo CEP 28625-650, Brazil; 3 Programa de Pós-graduação em Odontologia, Instituto de Saúde de Nova Friburgo, Universidade Federal Fluminense, Nova Friburgo CEP 28625-650, Brazil; 4 Program of Immunology and Tumor Biology, Brazilian National Cancer Institute, Division of Experimental and Translational Research, Rio de Janeiro CEP 20231-050, Brazil; 5 Instituto de Biodiversidade e Sustentabilidade, Universidade Federal do Rio de Janeiro, Macaé CEP 27965-045, Brazil; 6 Instituto de Ciências Biomédicas, Universidade Federal do Rio de Janeiro, Rio de Janeiro CEP 21941-902, Brazil; 7 Departamento de Tecnologia Farmacêutica, Faculdade de Farmácia, Universidade Federal Fluminense, Niterói CEP 24241-000, Brazil

**Keywords:** oral squamous cell carcinoma, naphthoquinones, acridines, pyruvate kinase m2, selectivity

## Abstract

Oral squamous cell carcinoma (OSCC) is a global public health problem with high incidence and mortality. The chemotherapeutic agents used in the clinic, alone or in combination, usually lead to important side effects. Thus, the discovery and development of new antineoplastic drugs are essential to improve disease prognosis and reduce toxicity. In the present study, acridine-core naphthoquinone compounds were synthesized and evaluated for their antitumor activity in OSCC cells. The mechanism of action, pharmacokinetics, and toxicity parameters of the most promising compound was further analyzed using in silico, in vitro, and in vivo methods. Among the derivatives, compound **4e** was highly cytotoxic (29.99 µM) and selective (SI 2.9) at levels comparable and generally superior to chemotherapeutic controls. Besides, compound **4e** proved to be non-hemolytic, stable, and well tolerated in animals at all doses tested. Mechanistically, compound **4e** promoted cell death by apoptosis in the OSCC cell, and molecular docking studies suggested this compound possibly targets enzymes important for tumor progression, such as RSK2, PKM2, and topoisomerase IIα. Importantly, compound **4e** presented a pharmacological profile within desirable parameters for drug development, showing promise for future preclinical trials.

## 1. Introduction

Cancer is one of the leading causes of death worldwide, comprising more than 100 different types of malignancies. Oral squamous cell carcinoma (OSCC), the main type of oral cancer, is a global public health problem with high incidence and mortality, with approximately 377,000 new cases and 177,000 deaths worldwide in 2020 [1]. Despite the availability of therapeutic options, the overall 5-year survival rate is 65%, ranging from 84% for localized cancers to only 39% for metastatic cancers [2], mainly due to the diagnosis occurring in advanced stages of the disease.

**Figure 1:**
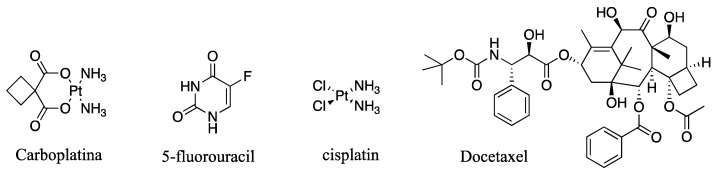
Most common chemotherapeutic agents for the treatment of OSCC.

According to the stage of the disease, the approaches used in the clinic are radiotherapy, surgery, and/or chemotherapy [3,4]. The most common chemotherapeutic agents for OSCC include carboplatin, 5-fluorouracil, cisplatin, and docetaxel (Figure 1), alone or in combination, but they lead to important side effects, such as diarrhea, nerve damage, and kidney damage [5]. Thus, the discovery and development of new antineoplastic drugs are essential to improve disease prognosis and reduce toxicity.

Several drugs used in cancer treatment are natural products or derivatives of them [6]. Indeed, naphthoquinones are representative of natural or synthetic compounds used in the development of health products, such as antibacterial, antifungal, antiprotozoal, antiviral, cytotoxic, and antitumor medicines [7,8]. One approach used in an attempt to improve the antitumor profile of naphthoquinones is to promote structural modifications in their core. Acridine derivatives are highly used in medicinal chemistry, presenting wide biological effects, with emphasis on anticancer actions mainly due to its ability to intercalate into DNA [9,10,11].

We herein report on the synthesis of a novel series of acridine-core naphthoquinone compounds and their evaluation against OSCC and normal oral human cells. Further, the mechanism of action, pharmacokinetic, and toxicity parameters of the most promising derivative were investigated by employing in silico, in vitro, and in vivo approaches.

## 2. Results and Discussion

### 2.1. Chemistry

**Scheme 1:**
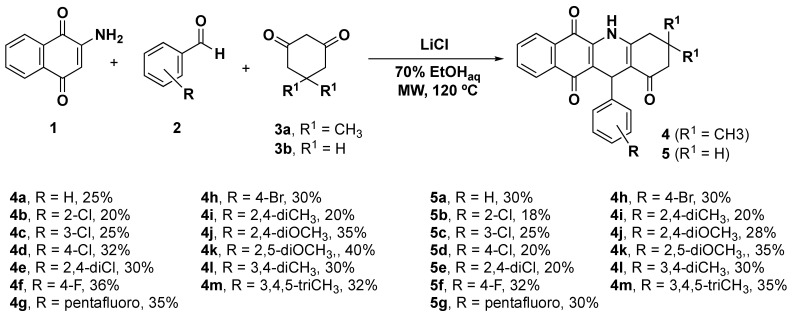
Synthesis of acridine-core naphthoquinone compounds **4** and **5**.

We performed the multicomponent synthesis of compounds **4** and **5** through a reaction between 2-amino-1,4-naphthoquinone (**1**) [12], substituted benzaldehydes (**2**), and 5,5-dimethyl-1,3-cyclohexanedione (dimedone, **3a**) or 1,3-cyclohexanedione (**3b**) in the presence of lithium chloride and a water/ethanol mixture as a solvent in a microwave reactor (Scheme 1). The synthesis of acridines was achieved in satisfactory yields as racemic mixtures, and their structures were confirmed by IR, NMR, and high-resolution mass spectrometry (See Supplementary Spectral Data SI).

**Scheme 2:**
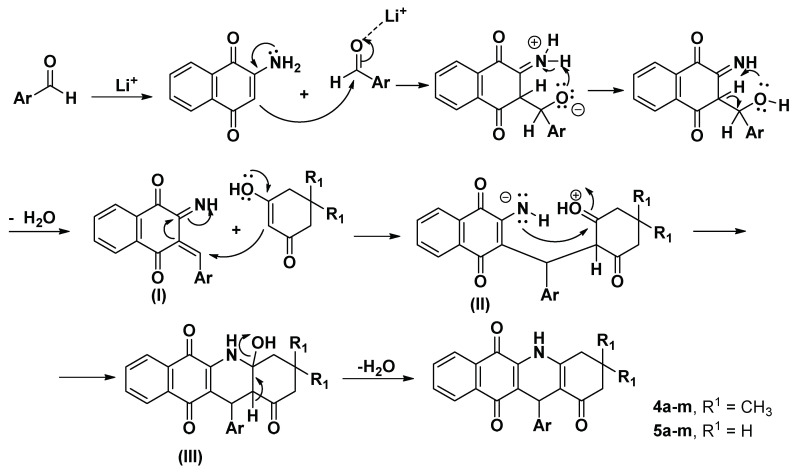
Proposed reaction mechanism for the synthesis of acridine-core naphthoquinone compounds **4** and **5**.

The proposed mechanism for this reaction is shown in Scheme 2 and occurs through nucleophilic attack on the carbonyl of the aldehyde by the C-3 carbon of 2-amino-1,4-naphthoquinone forming intermediate I, which will react with enol II of the 1,3-dicarbonylated compound to give intermediate III which upon dehydration will yield the condensation product 1,4 (**4** and **5**).

### 2.2. Biological Assays

#### 2.2.1. Cytotoxicity and Selectivity, Hemolytic Potential, and Biological Stability of the New Compounds

All 26 acridine-core naphthoquinone compounds were submitted to the MTT assay to assess their cytotoxicity. However, ten of them (**4a**, **4b**, **4f**, **4g**, **4i, 5a**, **5b**, **5f**, **5g**, **5i**) were highly insoluble and formed well-structured crystals at low concentrations, meaning it was impossible to test its cytotoxicity with precision, in vitro, and possibly turning them into bad drugs candidates. These compounds were excluded from further biological experimentation. First, the assay was conducted on the SCC9 OSCC cell line and the results were analyzed by a nonlinear regression curve to determine the value of the half maximal inhibitory concentration (IC_50_). As controls, we also assayed carboplatin, a standard chemotherapeutic agent for oral cancer [13], and the naphthoquinones doxorubicin, used in the clinic for breast cancer [14,15], shikonin and lapachol, both presenting antitumor potential [16,17]. Among all compounds, fifteen achieved 50% of viability reduction in the tested concentrations (Table 1) and presented IC_50_ values lower than controls, except shikonin and doxorubicin. Anticancer activities of these compounds are reported here for the first time.

**Figure 2:**
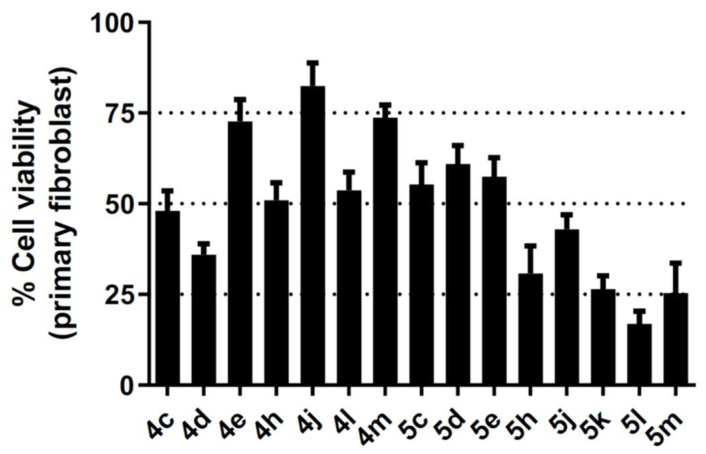
Determination of selective acridine-core naphthoquinone compounds in normal fibroblasts. Normal human gingival fibroblasts were treated with twice the calculated IC_50_ (2 × IC_50_) of the indicated compounds for 48 h and cell viability was determined by MTT assay. The IC_50_ of each compound was previously calculated on SCC9 (OSCC) cells and are demonstrated in Table 1. Results from at least three independent experiments.

The Selective Index (SI) represents the degree of selectivity of investigated molecules. A SI value ≥ 2 indicates selective toxicity towards cancer cells, while an SI value < 2 is considered generally toxic, meaning that it can also cause cytotoxicity in normal cells [18,19,20]. Primary human gingival fibroblasts were treated with 2 × IC_50_ (two times the calculated IC_50_) of each compound, and the ones that surpassed 75% of cell viability (**4e**, **4j,** and **4m**) (Figure 2), suggesting an SI > 2, were selected to have their IC_50_ calculated in fibroblasts and to be tested on two other OSCC cell lines, SCC4 and SCC25, which are less sensitive to antitumor agents.

The IC_50_ values for each cell line are lower than lapachol and significantly lower than carboplatin (Table 2). For each compound, the SI value was also calculated using the given formula: SI = IC_50_ normal cell/IC_50_ cancer cells (Table 2).

Among the three evaluated compounds, the derivative **4e** exhibited the greatest selectivity on all OSCC cell lines tested, with SI values higher than the controls, lapachol and shikonin (Table 2). It is noteworthy that compound **4e** is highly selective on the SCC9 and SCC25 OSCC cell lines, being even more effective than carboplatin used in the clinic. Thus, compound **4e** was chosen for the following assays.

**Figure 3:**
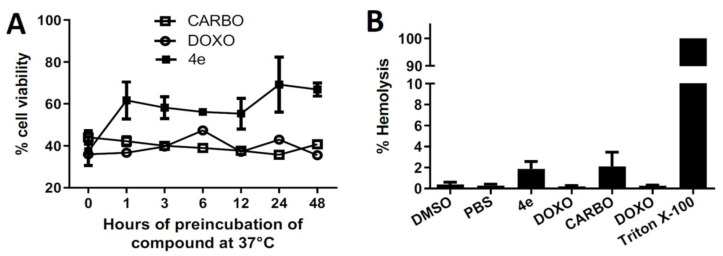
Determination of stability and induced hemolysis of the most selective naphthoquinone compound (**4e**) in OSCC cells. SCC9 cells were used for both assays. (**A**) For the stability test, compound **4e** and the control carboplatin were diluted to 2 × IC_50_ and maintained at 37 °C for 1, 3, 6, 12, 24, and 48 h prior to SCC9 cells treatment for a further 48 h followed by MTT assay. (**B**) The hemolytic activity of compound 4e was carried out using the concentration of 300 μM (approximately 10 × IC_50_). One-way ANOVA with Dunnett’s Posttest was performed where all columns were significantly different from the control (Triton X) with *p* < 0.0001.

It is desired that studied compounds present relative biological activity stability at 37 °C as the drugs already in use. To evaluate the biological activity stability of the new compound, **4e** or the control were pre-incubated for different time intervals at 37 °C (0, 1, 3, 6, 12, 24, and 48 h) and further tested for 48 h for their cytotoxicity against SCC9 cells. The results showed that, after the first hour, compound **4e** is biological stable in a similar fashion as carboplatin (Figure 3A) with little variation in the cytotoxicity; however, further analysis is needed to determine if the compound is chemically stable.

Next, a hemolytic assay was performed to discard any surfactant activity of compound **4e**, which could induce unspecific cytotoxicity through cellular membrane damage. Figure 3B shows that compound **4e** and the controls lack hemolytic potential, with less than 3% of hemolysis compared to the positive control, triton x-100, which represents 100% of lysis in red blood cells. Indeed, this result corroborates others in the literature where synthetic naphthoquinones also have no hemolytic potential [19,20,21]. Taken together, compound **4e** was proven to be selective against OSCC cells and non-hemolytic, encouraging in vivo testing.

#### 2.2.2. Acute Toxicity in Vivo

Pre-clinical tests are important for drug development and the comprehension of the therapeutic potential of new molecules [22]. Thus, an acute toxicity assay was performed using compound **4e** in C56BL/6 mice to investigate its toxic potential. Three different groups of animals received an intraperitoneal single dose of 100, 200, or 400 mg/kg of compound **4e** and were accompanied for 14 days and presented no changes in morbidity and mortality (Table 3). The histopathological analysis indicated that the 200 mg/kg and 400 mg/kg groups demonstrated high numbers of Bronchus-Associated Lymphoid Tissue (BALT) and Perivascular and periportal lymphocyte focus (Table 3 and Table S1). These are signs of a possible inflammation in the lungs and liver that are comparable to the control and almost absent in the lower dose group (100 mg/Kg). Moreover, there was no significant difference in body weight and food consumption relative to control animals in any dose (Supplementary Figure S1A,B). In conclusion, we found no apparent limiting toxic effects of compound **4e** on mice in the tested concentrations, turning this molecule into a good candidate for further anticancer in vivo tests in lower doses.

#### 2.2.3. Prediction of Anticancer Targets of **4e** by Reverse Docking

Next, we continued the investigation of possible cell death mechanisms involved in compound **4e** action by reverse docking. Herein, we prepared a protein pool containing six proven targets of lapachol and other naphthoquinones which are also related to anticancer activity, and we performed a reverse screening to predict the potential target of **4e**. Of note, both enantiomers of **4e** were evaluated in this study, but the results presented here refer to the *S* enantiomer of this compound as this one was suggested to contribute mostly to its biological activity according to our docking studies.

**Figure 4:**
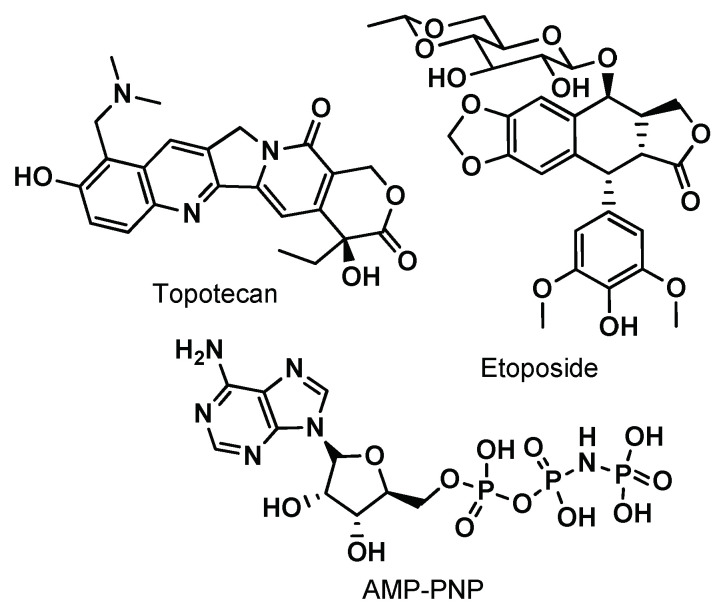
Structures of the co-crystallized inhibitors with the DNA-binding domain of topoisomerase I (topotecan) and topoisomerase IIα and IIβ (etoposide) as well as with the ATPase domain of topoisomerase IIα (AMP-PNP).

Initially, we evaluated the binding mode of **4e** with the DNA-binding domain of topoisomerases I, IIα, and IIβ. Etoposide and topotecan are anticancer drugs used for treating different cancer types and are known to inhibit DNA topoisomerases through intercalation with DNA [23]. Since the 3D structures of these proteins complexed with these drugs have been solved experimentally, they were used in the docking studies for comparison purposes. Although **4e** shares a similar polycyclic moiety to etoposide and topotecan (Figure 4), this compound was not able to intercalate between the DNA nucleobases within topoisomerases complexes as the known inhibitors (data not shown).

**Figure 5:**
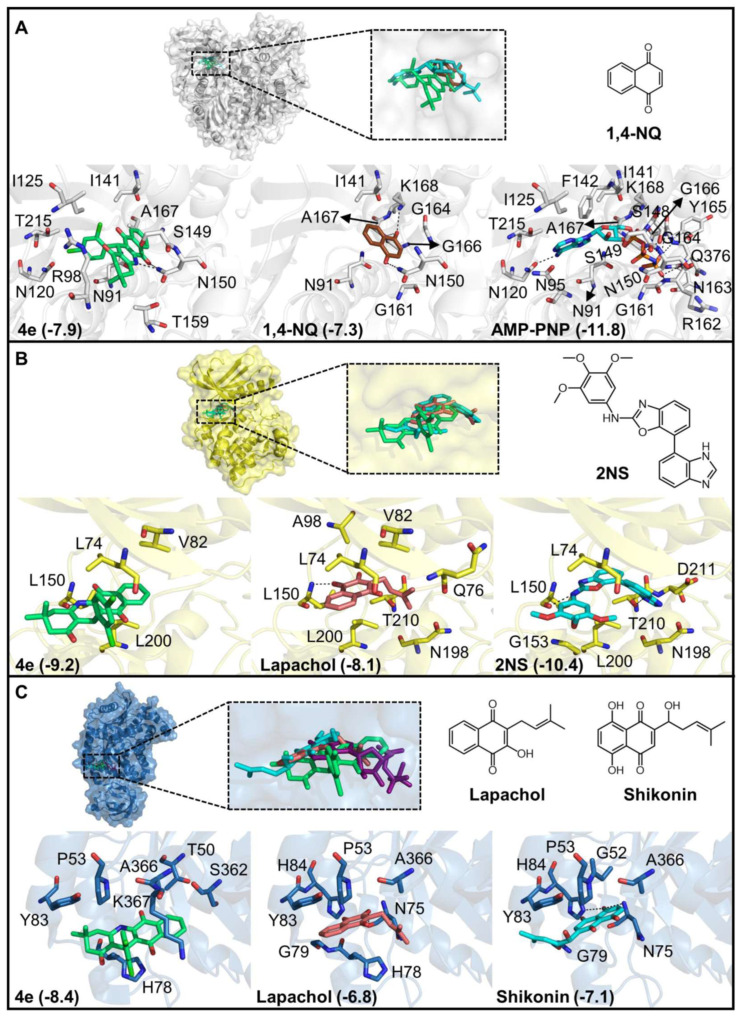
Docking of compound 4e with its putative anticancer targets and comparison with the known inhibitors. Superimposition and interaction pattern of (**A**) **4e** (green), 1,4-naphthoquinone (1,4-NQ; brown), and AMP-PNP (cyan) with the ATPase domain of topoisomerase IIα; (**B**) **4e** (green), lapachol (pink), and **2NS** (cyan) with RSK2; (**C**) **4e** (green), lapachol (pink), and shikonin (cyan) with PKM2. The ATP bound to the rabbit PKM2 is also shown in purple in the superimposition. Hydrogen bonds and salt bridges are shown as dashed lines. The predicted binding energies of the compounds with the respective enzymes are shown in parenthesis (in Kcal/mol). The binding energies of the co-crystallized inhibitors were calculated by redocking studies.

On the other hand, **4e** bound within the ATP binding site of the ATPase domain of topoisomerase IIα, similarly to the co-crystallized ligand AMP-PNP (Figure 5A), which is a nonhydrolyzable ATP analog (Figure 4) that competes for the ATP binding site and inhibits topoisomerases II activities [23]. In addition, the compound 1,4-naphthoquinone (1,4-NQ) was previously shown to inhibit the ATPase activity of this enzyme [24] and, since **4e** is a 1,4-naphthoquinone derivative, we also performed docking studies with 1,4-NQ. Interestingly, **4e** exhibited a similar interaction network compared to 1,4-NQ (Figure 5A and Table S2), suggesting this enzyme is a potential target. Although **4e** presented comparable binding energy to 1,4-NQ (−7.9 and −7.2 Kcal/mol, respectively), both naphthoquinones exhibited lower theoretical affinity with this enzyme than AMP-PNP (binding energy = −11.8 Kcal/mol).

In 2019, Zu and coworkers demonstrated that the inhibition of RSK2 (ribosomal protein S6 kinase 2) triggers the cytotoxicity of lapachol in squamous cell carcinoma [25]. Considering that lapachol is a 1,4-naphthoquinone derivative with anticancer potential [16], we further investigated the binding mode of **4e** with this protein and compared it with lapachol and **2NS**, a benzoxazole derivative co-crystallized with this protein that inhibits RSK2 selectively and exhibits cytotoxic effects towards breast cancer cell lines [26]. Interestingly, the naphthoquinone derivative **4e** exhibited a similar binding manner as observed for lapachol and the inhibitor **2NS** (Figure 5B). However, **2NS** and **4e** presented higher binding affinity with this protein than lapachol (−10.4, −9.2, and −8.1 Kcal/mol, respectively). In addition, compound **4e** conserved several interactions noticed for **2NS** and lapachol (Figure 5B and Table S2), including a hydrogen-bond interaction with L150 that is likely critical for the binding of several RSK2 inhibitors [26,27,28], which, in turn, suggests this enzyme as a putative anticancer target of compound **4e**.

Furthermore, lapachol and shikonin are 1,4-NQ derivatives and blocked the glycolytic pathway in cancer cells by targeting the PKM2 (human M2 pyruvate kinase) [29,30]. Hence, we also investigated how compound **4e** would interact with this enzyme. Like lapachol and shikonin, compound **4e** explored the entrance of the ATP binding site within PKM2 (Figure 5C), though **4e** (−8.4 Kcal/mol) exhibited a higher affinity for PKM2 than for the other naphthoquinones lapachol (−6.8 Kcal/mol) and shikonin (−7.1 Kcal/mol). Importantly, these naphthoquinone derivatives shared similar contacts with the human enzyme (Figure 5C and Table S2), which was also comparable to the interactions of ATP bound with the rabbit homologous protein [31] and further supports the potential of the compound **4e** to bind and inhibit this enzyme.

Interestingly, our data suggested that compound **4e** resembles the binding mode of known inhibitors and natural ligands within the ATP binding sites of three proteins. However, these proteins belong to different classes and exhibit diverse functions: PKM2 is a carbohydrate kinase [32]; RSK2 is a serine/threonine protein kinase [25]; and the ATPase domain of topoisomerase IIα catalyzes the ATP hydrolysis for energy generation [33]. Consequently, they do not share highly similar active sites (Table S3). In fact, RSK2 exhibits a more hydrophobic active site among the proteins. On the other hand, the other proteins also possess a high number of nonpolar residues in their active site, which suggests that hydrophobic interactions play an important role in anchoring the natural ligand and were also tested by the compound **4e**. Besides, other structural features may contribute to the binding of naphthoquinone inhibitors within these three proteins. For instance, polar residues found in more-exposed regions are important for the binding of the purine and sugar moieties of the ATP and also seem to stabilize **4e** in the predicted targets as discussed. As well, charged residues located within deeper regions of ATP binding sites are essential for the binding of ions and phosphate groups and their transfer during the enzyme reaction and could be further explored in the design of novel derivatives.

Collectively, our findings suggest that compound **4e** could bind to the ATPase domain of topoisomerase IIα, RSK2, and PKM2 similarly to proven inhibitors with comparable or even greater affinities than the inhibitors of these targets (e.g., in PKM2). Indeed, these enzymes are important for tumor progression [32,34,35]. Therefore, the anticancer activity and higher selectivity of this compound may occur through its effects on these enzymes.

#### 2.2.4. Predicted Toxicity and Pharmacokinetic Properties of **4e**

In silico assays are part of computational pharmacology, allowing to predict and understand how drugs affect biological systems, which in turn can improve clinical use and avoid unwanted side effects [36]. In this way, a set of relevant properties of compound **4e** was calculated and compared with controls used in the clinic (carboplatin and doxorubicin) using SwissADME and admetSAR 2 servers. Lipinski’s “rule of 5” was used to evaluate oral bioavailability according to four parameters: (1) the logarithm of the octanol/water partition coefficient (cLogP ≤ 5); (2) the number of hydrogen bond acceptors (nON ≤ 10); (3) the number of hydrogen bond donors (nOH/NH ≤ 5), and (4) molecular weight (MW ≤ 500 Da) [37]. Compounds with two or more violations of these criteria probably do not have good permeation and absorption. Overall, compound **4e** fulfilled the Lipinski “rule of 5”, while the control drugs doxorubicin and carboplatin had three and no violations, respectively (Table 4).

Additionally, the topological polar surface area (TPSA) is one of the parameters used to predict drug cell permeability, oral bioavailability, and intestinal absorption. Compounds with TPSA above 140 Å^2^ have low membrane permeability whereas compounds with TPSA less than 60 Å^2^ have high permeability and human intestinal absorption [38]. The values in Table 4 show that **4e** has a low value of TPSA and is close to the ideal (63.2 Å^2^), demonstrating that this compound has good permeability. By contrast, carboplatin (126.6 Å^2^) and doxorubicin (206.1 Å^2^) showed higher values of TPSA, indicating that these compounds are likely to exhibit low cell permeability and intestinal absorption.

In the last decade, about 50% of drugs under development failed in absorption, distribution, metabolism, excretion, and toxicity, parameters that, when abbreviated in an acronym, are called ADMET [39]. Thus, to reinforce the rule-based prediction of absorption and permeability, we also predicted the bioavailability of compound **4e** using a QSAR-based method available within the admetSAR 2.0 server. Interestingly, compound **4e** was predicted to present a good oral bioavailability, whereas the controls doxorubicin and carboplatin were predicted to exhibit poor oral bioavailability. Indeed, experimental studies have demonstrated the low oral bioavailability of these drugs [40,41], proving the reliability of our predictions which, in turn, supports that compound **4e** is suitable for oral delivery, unlike the anticancer drugs evaluated.

Since phosphoglycoprotein-P (Pg-P) is linked to drug resistance, we also evaluated whether compound **4e** could act as a substrate or inhibitor of this protein. Compound **4e** was not predicted to be a substrate or inhibitor of Pg-P (Table 5). Likewise, carboplatin was not suggested to act as a substrate or inhibitor of Pg-P, but doxorubicin was indicated to be a substrate, but not an inhibitor, of this protein, implying that it is transported by Pg-P and expelled through these efflux pumps. These predictions are in agreement with the experimental data available for both control drugs [42]. Therefore, the in silico analyses suggest that compound **4e**, in addition to having a good pharmacological profile, might be orally absorbed, and is not a substrate of Pg-P, increasing its likelihood as a good drug lead.

#### 2.2.5. Cell Death Investigation

In view of the results showing that compound **4e** is selective and well-tolerated in mice, we next focused on determining the possible cell death mechanism and pathway involved. Chemotherapy induces different types of cell death, and the identification of the exact pathway is important in the development of new anticancer agents [43].

**Figure 6:**
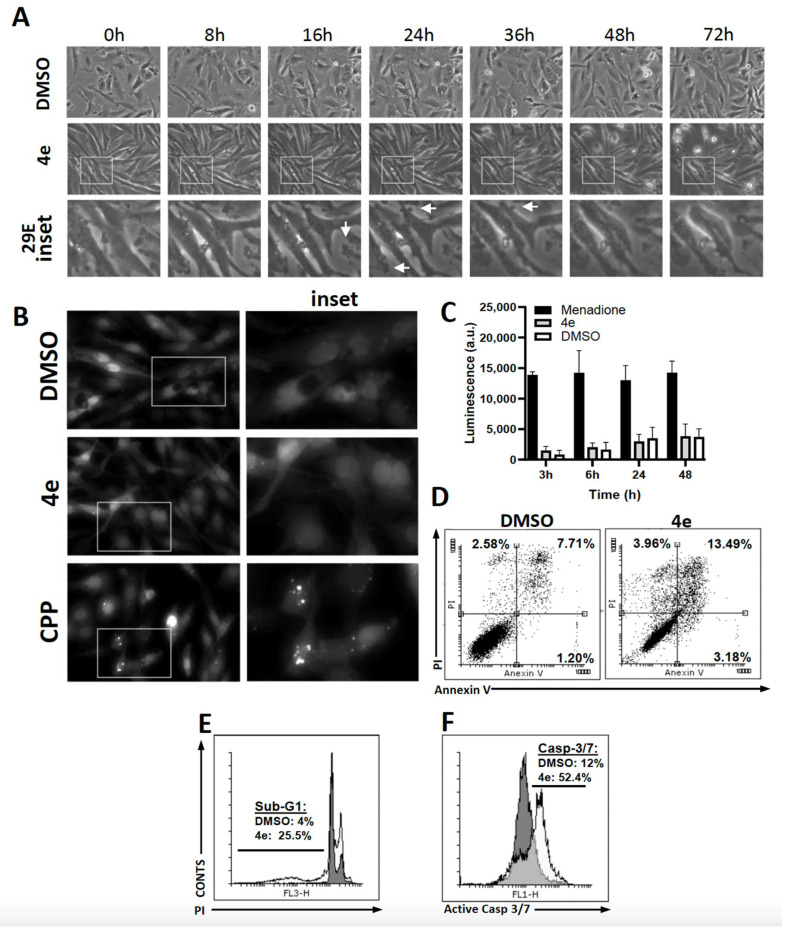
Cell death mechanism investigation for the most selective naphthoquinone derivative 4e in OSCC cells. All experiments were performed using SCC9 cells. (**A**) Time-lapse video microscopy images obtained from Supplementary Video S1 reveal that **4e** (2 × IC_50_) induces the appearance of membrane blebs, the loss of membrane integrity, and cellular rupture, which were intensified at 36 and 48 h. The same result was not observed for the DMSO control. (**B**) Determination of autophagosome formation. SCC9 expressing LC3 fused to GFP protein were treated with 2 × IC_50_ of **4e**, DMSO, or calcium phosphate precipitate (CPP) for 48 h, and puncta formation was observed by fluorescent microscopy. (**C**) Quantification of produced reactive oxygen species (ROS) by SCC9 cells treated with compound **4e** or controls (DMSO) for 3, 6, 24, and 48 h. Menadione was used as a positive control. (**D**) Analysis of phosphatidylserine exposure 48 h after plating. Cells were stained with annexin-V conjugated to FITC and propidium iodide (P.I.) and analyzed by flow cytometry. (**E**) Representative flow cytometry dot plot of DNA fragmentation (sub-G0 DNA content) using P.I. staining. After 72 h of treatment with compound **4e** or control (DMSO), cells were trypsinized and stained with propidium iodide in the presence of NP-40. (**F**) Representative flow cytometry dot plot of caspase 3/7 activity. After 48 h of treatment with compound **4e** or control (DMSO), cells were trypsinized and stained for active caspase 3/7. Results from at least three independent experiments.

Changes in the morphology of cells are a potent indicator of which cell death process is taking place under a certain stimulation. In apoptosis, cells shrink, and DNA fragments and apoptotic bodies are released [44]. Through time-lapse microscopy (Figure 6A and Supplementary Video S1), oral cancer cells treated with compound **4e** presented the formation of membrane blebs at early times, between 16 and 48 h, followed by cells shrinkage, both suggestive of apoptosis. Decreases in cell proliferation and migration were also observed after **4e** treatment when compared to the control condition (DMSO) (Supplementary Video S1).

In order to better investigate the cytotoxic process and exclude other pathways, we tested the possibility of cell death induced by autophagy. For that, we used SCC9 cells expressing the microtubule-associated protein 1A/1B-light chain 3 (LC3) fused with GFP protein. LC3 is a protein that, during autophagy, is recruited to autophagosomes which can be visualized as *punctas* in microscopy. As seen in Figure 6B, while calcium phosphate precipitate (CPP), an autophagy inducer [45] induces an LC3-GFP puncta formation indicative of autophagy, neither DMSO nor **4e** were able to induce this cell death process.

Different naphthoquinones can produce ROS (reactive oxygen species), being one of the properties that most confers antineoplastic action and an inducer of apoptosis to this class of substances [46,47]. Therefore, regarding the cell death mechanism, we investigated ROS production in our OSCC in vitro model, where the naphthoquinone menadione was used as a positive control. In Figure 6C, it is notable that a low production of ROS in SCC9 cells treated with compound **4e** at all times tested similar to that observed for controls, indicating that ROS is not a mechanism of cell death induction by compound **4e**.

To validate these morphological phenotypes indicative of apoptosis, we performed flow cytometry assays. The treatment of SCC9 cells with compound **4e** showed increased single (Annexin V) and double (Annexin V + PI) staining (Figure 6D), induced DNA fragmentation in ~25% of the cells (Figure 6E, Sub-G1 DNA-content), and activated effector caspase 3/7 labeling in ~52% of cells (Figure 6F). Altogether, the results demonstrate that compound **4e** promotes oral cancer cell death by apoptosis.

## 3. Materials and Methods

### 3.1. Chemistry

The reagents were purchased from Sigma-Aldrich Brazil and were used without further purification. The reactions were carried out in a microwave reactor (CEM, Corporation, Matthews, NC, USA, Discovery system model 908005). Column chromatography was performed with silica gel 60 (Merck 70–230 mesh). Analytical thin layer chromatography was performed with silica gel plates (Merck, TLC silica gel 60 F_254_), and the plates were visualized using UV light. Melting points were obtained on a Fisher-Johns apparatus and were uncorrected. Infrared spectra were collected using KBr pellets on a Perkin-Elmer model 1420 FT-IR spectrophotometer, and the spectra were calibrated relative to the 1601.8 cm^−1^ absorbance of polystyrene. NMR spectra were recorded at room temperature using a Varian Unity Plus VXR (500.00 or 300.00 MHz), in solutions of DMSO-*d*_6_ or CDCl_3_. The chemical shift data were reported in units of δ (ppm) downfield from solvent, and the solvent was used as an internal standard; coupling constants (*J*) are reported in Hertz and refer to apparent peak multiplicities. High-resolution mass spectra (HRMS) were recorded on a MICROMASS Q-TOF mass spectrometer (Waters) or on a QExactive TM Hybrid Quadrupole Orbitrap Mass Spectrometer (Thermo Fisher Scientific, Waltham, MA, USA) using electrospray ionization (ESI).

General Procedure for the synthesis of acridine-core naphthoquinone compounds **4** and **5**.

In a microwave reactor flask, 1 mmol of 2-amino-1,4-naphthoquinone (**1**) [12] 1.5 mmol of substituted benzaldehydes (**2**), 1.5 mmol of 5,5-dimethyl-1,3-cyclohexanedione (**3a**) or 1,3-cyclohexanedione (**3b**), and 1 mmol of lithium chloride were added in an EtOH/H_2_O (7:3) mixture (5 mL). The reaction was maintained at 1200 rpm, 5.5 bar, and 120 °C for 20–30 min, depending on the benzaldehyde used. Then, the reaction mixture was extracted with dichloromethane (3 × 10 mL) and the organic phase dried over anhydrous sodium sulfate, filtered, and the solvent removed in vacuo. The resulting solid was purified by preparative plate chromatography using a hexane/ethyl acetate mixture as eluent.

**4a**: 12-(3,3-dimethylphenyl)-3,4-dihydrobenzo[*b*]acridine-1,6,11(2*H*,5*H*,12*H*)-trione. Red solid, m.p. 296–298 °C, yield 25%. IR (KBr) ν cm^−1^: 3340,2959, 1668, 1638, 1604, 1590, 1487, 1392, 1340, 1222, 1141, 725, 709, 696. ^1^H NMR (500.00 MHz, CDCl_3_) δ (ppm): 8.05 (1H, dd, *J* = 7.6 and 1.0 Hz), 8.02 (1H, dd, *J* = 7.6 and 1.0 Hz), 7.70 (1H, dt, *J* = 7.5 and 1.3 Hz), 7.60–7.65 (1H, m), 7.40 (1H, d, *J* = 1.3 Hz), 7.38 (1H, d, *J* = 1.0 Hz), 7.22 (2H, t, *J* = 7.4 Hz), 7.11 (1H, t, *J* = 7.3 Hz), 5.41 (1H, s), 2.52 (1H, d, *J* = 16.8 Hz), 2.47 (1H, d, *J* = 16.8 Hz), 2.30 (1H, d, *J* = 16.3 Hz), 2.24 (1H, d, *J* = 16.3 Hz), 1.13 (3H, s), 1.04 (3H, s).^13^C NMR (75.0 MHz, CDCl_3_) δ (ppm): 195.2, 182.1, 180.1, 147.3, 144.9, 136.7, 134.8, 133.7, 132.7, 132.7, 130.1, 130.1, 129.2, 128.4, 128.3, 126.7, 126.0, 121.1, 112.2, 50.6, 41.2, 34.6, 32.7, 29.2, 27.3. HRMS *m/z*: [M+Na]^+^ = 406.1422 (Calculated for C_25_H_21_NO_3_Na^+^ = 406.1419).

**4b**: 12-(2-chlorophenyl)-3,3-dimethyl-3,4-dihydrobenzo[*b*]acridine-1,6,11(2*H*,5*H*,12*H*)-trione. Redsolid, m.p. 253–254 °C, yield 20%. IR (KBr) ν cm^−1^: 3315,2957, 1719, 1674, 1642, 1608, 1470, 1382, 1224, 1142, 1072, 1036, 744, 655.^1^H NMR (300.00 MHz, CDCl_3_) δ (ppm): 8.02–8.09 (2H, m), 7.66–7.73 (2H, m), 7.29–7.36 (2H, m), 7.14–7.19 (1H, m), 7.07–7.11 (1H, m), 5.39 (1H, s), 2.51 (2H, s), 2.27 (2H, d, *J* = 3.3 Hz), 1.13 (3H, s), 1.05 (3H, s). ^13^C NMR (125.0 MHz, CDCl_3_) δ (ppm): 195.1, 182.1, 179.9, 147.5, 141.8, 137.4, 136.5, 134.9, 133.5, 132.7, 130.3, 129.3, 127.9, 127.6, 126.4, 126.0, 119.6, 115.6, 111.3, 50.6, 41.2, 32.0, 31.5, 29.2, 27.3. HRMS *m/z*: [M+H]^+^ = 418.1212 (Calculated for C_25_H_20_ClNO_3_H^+^ = 418.1210).

**4c**: 12-(3-chlorophenyl)-3,3-dimethyl-3,4-dihydrobenzo[*b*]acridine-1,6,11(2*H*,5*H*,12*H*)-trione. Redsolid, m.p. 249–251 °C, yield 25%. IR (KBr) ν cm^−1^: 3343,2885, 1636, 1587, 1474, 1380, 1351, 1217, 1136, 934, 798, 721, 641. ^1^H NMR (500.00 MHz, CDCl_3_) δ (ppm): 8.06 (1H, dd, *J* = 7.6 and 1.1 Hz), 8.02 (1H, dd, *J* = 7.6 and 1.1 Hz), 7.71 (1H, t, *J* = 7.4 Hz), 7.65 (1H, t, *J* = 7.4 Hz), 7.34 (1H, d, *J* = 7.6 Hz), 7.30 (1H, sl), 7.14–7.17 (1H, m), 7.06–7.09 (1H, m), 5.39 (1H, s), 2.50 (2H, s), 2.29 (1H, d, *J* = 16.4 Hz), 2.24 (1H, d, *J* = 16.4 Hz), 1.13 (3H, s), 1.05 (3H, s).^13^C NMR (125.0 MHz, CDCl_3_) δ (ppm): 195.0, 181.9, 179.9, 147.4, 146.8, 136.8, 134.9, 134.1, 132.9, 132.6, 130.0, 128.2, 126.9, 126.9, 126.7, 126.1, 124.9, 120.3, 111.7, 50.6, 41.2, 34.6, 32.8, 29.2, 27.4. HRMS *m/z*: [M+H]^+^ = 418.1211 (Calculated for C_25_H_20_ClNO_3_H^+^ = 418.1210).

**4d**: 12-(4-chlorophenyl)-3,3-dimethyl-3,4-dihydrobenzo[*b*]acridine-1,6,11(2*H*,5*H*,12*H*)-trione. Redsolid, m.p. 287–288 °C, yield 32%. IR (KBr) ν cm^−1^: 3375,2958, 1669, 1645, 1607, 1589, 1470, 1381, 1335, 1219, 1085, 1014, 722. ^1^H NMR (500.00 MHz, CDCl_3_) δ (ppm): 8.06 (1H, d, *J* = 7.5 Hz), 8.03 (1H, d, *J* = 7.5 Hz), 7.71 (1H, dt, *J* = 7.5 and 1.3 Hz), 7.65 (1H, dt, *J* = 7.5 and 1.3 Hz), 7.33 (1H, d, *J* = 8.4 Hz), 7.18 (2H, d, *J* = 8.4 Hz), 5.38 (1H, s), 2.52 (1H, d, *J* = 16.7 Hz), 2.46 (1H, d, *J* = 16.7 Hz), 2.29 (1H, d, *J* = 16.3 Hz), 2.23 (1H, d, *J* = 16.3 Hz), 1.13 (3H, s), 1.03 (3H, s).^13^C NMR (125.0 MHz, CDCl_3_) δ (ppm): 195.0, 182.0, 179.9, 147.2, 143.5, 136.8, 134.9, 132.9, 132.6, 130.0, 129.7, 128.4, 128.3, 128.1, 126.7, 126.1, 120.6, 115.3, 111.9, 50.6, 41.2, 34.3, 32.8, 32.4. HRMS *m/z*: [M+H]^+^ = 418.1205. (Calculated for C_25_H_20_ClNO_3_H^+^ = 418.1210).

**4e**:12-(2,4-dichlorophenyl)-3,3-dimethyl-3,4-dihydrobenzo[*b*]acridine-1,6,11(2*H*,5*H*,12*H*)-trione. Redsolid, m.p. 262–263 °C, yield 30%. IR (KBr) ν cm^−1^: 3391,2956, 1678, 1650, 1630, 1596, 1472, 1390, 1349, 1226, 1141, 1048, 721.^1^H NMR (500.00 MHz, CDCl_3_) δ (ppm): 8.06 (1H, dd, *J* = 7.6 and 1.1 Hz), 8.00 (1H, dd, *J* = 7.6 and 1.1 Hz), 7.70 (1H, dt, *J* = 7.5 and 1.3 Hz), 7.65 (1H, dt, *J* = 7.5 and 1.3 Hz), 7.47 (1H, d, *J* = 8.3 Hz), 7.27–7.30 (1H, m), 7.15 (1H, dd, *J* = 8.3 and 2.1 Hz), 5.64 (1H, s), 2.49 (1H, d, *J* = 16.6 Hz), 2.42 (1H, d, *J* = 16.7 Hz), 2.26 (1H, d, *J* = 16.4 Hz), 2.19 (1H, d, *J* = 16.4 Hz), 1.12 (3H, s), 1.03 (3H, s).^13^C NMR (75.0 MHz, CDCl_3_) δ (ppm): 195.1, 182.2, 179.8, 147.8, 140.3, 137.5, 135.0, 134.2, 133.4, 132.9, 132.8, 132.5, 129.9, 129.7, 126.8, 126.6, 126.1, 119.1, 110.9, 50.5, 41.2, 34.9, 32.5, 29.2, 27.3. HRMS *m/z*: [M+Na]^+^ = 474.0640. (Calculated for C_25_H_19_Cl_2_NO_3_Na^+^ = 474.0640).

**4f**: 12-(4-fluorophenyl)-3,3-dimethyl-3,4-dihydrobenzo[*b*]acridine-1,6,11(2*H*,5*H*,12*H*)-trione. Red solid, m.p. 295–297 °C, yield 36%. IR (KBr) ν cm^−1^: 3346,2927, 1637, 1603, 1485, 1381, 1340, 1219, 1141, 1016, 924, 835, 721, 631.^1^H NMR (500.00 MHz, CDCl_3_) δ (ppm): 8.06 (1H, dd, *J* = 7.5 and 1.0 Hz), 8.02 (1H, dd, *J* = 7.6 and 1.1 Hz), 7.71 (1H, dt, *J* = 7.5 and 1.3 Hz), 7.65 (1H, dt, *J* = 7.5 and 1.3 Hz), 7.36 (2H, dd, *J* = 8.8 and 5.5 Hz), 7.20 (1H, sl), 6.90 (2H, t, *J* = 8.7 Hz), 5.39 (1H, s), 2.52 (1H, d, *J* = 16.7 Hz), 2.46 (1H, d, *J* = 16.9 Hz), 2.29 (1H, d, *J* = 16.3 Hz), 2.23 (1H, d, *J* = 16.3 Hz), 1.13 (3H, s), 1.03 (3H, s).^13^C NMR (75.0 MHz, CDCl_3_) δ (ppm): 195.0, 182.1, 180.0, 161.5 (d, *J* = 244.8 Hz), 147.1, 140.9, 136.7, 134.9, 132.8, 132.6, 130.0, 129.8 (d, *J* = 8.1 Hz), 126.6, 126.1, 120.8, 115.1 (d, *J* = 21.5 Hz), 112.2, 50.6, 41.2, 34.0, 32.7, 29.2, 27.3.HRMS *m/z*: [M+H]^+^ = 402.1521 (Calculated for C_25_H_20_FNO_3_H^+^ = 402.1505).

**4g**: 12-(perfluorophenyl)-3,3-dimethyl-3,4-dihydrobenzo[*b*]acridine-1,6,11(2*H*,5*H*,12*H*)-trione. Redsolid, m.p. 260–261 °C, yield 35%. IR (KBr) ν cm^−1^: 3328,2942, 1675, 1639, 1606, 1480, 1383, 1337, 1220, 1110, 991, 956, 722.^1^H NMR (500.00 MHz, CDCl_3_) δ (ppm): 8.07 (1H, dd, *J* = 7.6 and 1.0 Hz), 8.02 (1H, dd, *J* = 7.6 and 1.0 Hz), 7.74 (1H, dt, *J* = 7.5 and 1.3 Hz), 7.68 (1H, dt, *J* = 7.5 and 1.3 Hz), 7.38 (1H, sl), 5.72 (1H, s), 2.50 (1H, d, *J* = 16.7 Hz), 2.43 (1H, d, *J* = 16.7 Hz), 2.30 (1H, d, *J* = 16.3 Hz), 2.22 (1H, d, *J* = 16.3 Hz), 1.13 (3H, s), 1.03 (3H, s).^13^C NMR (125.0 MHz, CDCl_3_) δ (ppm): 195.0, 181.9, 179.4, 148.7, 137.9, 135.2, 133.1, 132.3, 129.9, 126.6, 126.3, 116.6, 108.6, 50.4, 41.1, 32.6, 29.1, 27.1, 26.2. HRMS *m/z*: [M+H]^+^ = 474.1141 (Calculated for C_25_H_16_F_5_NO_3_H^+^ = 474.1129).

**4h**: 12-(4-bromophenyl)-3,3-dimethyl-3,4-dihydrobenzo[*b*]acridine-1,6,11(2*H*,5*H*,12*H*)-trione. Redsolid, m.p. 293–295 °C, yield 30%. IR (KBr) ν cm^−1^: 3340,2950, 1665, 1642, 1605, 1590, 1484, 1384, 1338, 1222, 1140, 1024, 723. ^1^H NMR (500.00 MHz, CDCl_3_) δ (ppm): 8.06 (1H, dd, *J* = 7.5 and 1.0 Hz), 8.02 (1H, dd, *J* = 7.6 and 1.0 Hz), 7.71 (1H, dt, *J* = 7.5 and 1.3 Hz), 7.65 (1H, dt, *J* = 7.5 and 1.3 Hz), 7.34 (2H, d, *J* = 8.5 Hz), 7.27 (2H, d, *J* = 8.5 Hz), 5.36 (1H, s), 2.52 (1H, d, *J* = 16.7 Hz), 2.46 (1H, d, *J* = 16.7 Hz), 2.29 (1H, d, *J* = 16.3 Hz), 2.23 (1H, d, *J* = 16.3 Hz), 1.13 (3H, s), 1.03 (3H, s).^13^C NMR (75.0 MHz, CDCl_3_) δ (ppm): 195.0, 182.0, 179.9, 147.3, 144.0, 136.8, 135.0, 132.9, 132.6, 131.4, 130.1, 130.0, 126.7, 126.1, 120.5, 111.9, 109.9, 50.6, 41.2, 34.4, 32.8, 29.2, 27.3. HRMS *m/z*: [M+Na]^+^ = 484.0521 (Calculated for C_25_H_20_BrNO_3_Na^+^ = 484.0524).

**4i**: 12-(2,4-dimethylphenyl)-3,3-dimethyl-3,4-dihydrobenzo[*b*]acridine-1,6,11(2*H*,5*H*,12*H*)-trione. Redsolid, m.p. 264–265 °C, yield 20%. IR (KBr) ν cm^−1^: 3317,2952, 1772, 1671, 1640, 1614, 1487, 1371, 1335, 1218, 1141, 1098, 935, 723.^1^H NMR (500.00 MHz, CDCl_3_) δ (ppm): 8.04 (1H, dd, *J* = 7.6 and 1.2 Hz), 7.99 (1H, dd, *J* = 7.6 and 1.2 Hz), 7.68 (1H, dt, *J* = 7.5 and 1.3 Hz), 7.62 (1H, dt, *J* = 7.5 and 1.3 Hz), 7.17 (1H, sl), 7.13 (1H, d, *J* = 8.0 Hz), 7.00 (1H, d, *J* = 8.0 Hz), 6.94 (1H, d, *J* = 7.3 Hz), 5.39 (1H, s), 2.91 (3H, s), 2.51 (2H, d, *J* = 16.6 Hz), 2.43 (2H, d, *J* = 16.6 Hz), 2.12 (1H, s), 1.19 (3H, s), 1.00 (3H, s).^13^C NMR (75.0 MHz, CDCl_3_) δ (ppm): 195.3, 189.4, 189.2, 146.6, 142.2, 136.6, 136.0, 135.7, 134.8, 133.6, 132.7, 132.1, 131.0, 130.0, 128.4, 127.5, 126.3, 122.8, 116.4, 50.7, 41.3, 31.7, 31.5, 30.6, 28.3, 20.9, 20.4. HRMS *m/z*: [(M-CH_3_)+Na]^+^ = 419.2204. (Calculated for C_26_H_22_ClNO_3_Na^+^ = 419.1497).

**4j**: 12-(2,4-dimethoxyphenyl)-3,3-dimethyl-3,4-dihydrobenzo[*b*]acridine-1,6,11(2*H*,5*H*,12*H*)-trione. Redsolid, m.p. 250–251 °C, yield 35%. IR (KBr) ν cm^−1^: 3364,2957, 1632, 1587, 1465, 1385, 1351, 1295, 1212, 1124, 1029, 925, 835, 720, 635. ^1^H NMR (500.00 MHz, CDCl_3_) δ (ppm): 8.03 (1H, dd, *J* = 7.6 and 1.0 Hz), 7.99 (1H, dd, *J* = 7.6 and 1.0 Hz), 7.67 (1H, dt, *J* = 7.5 and 1.3 Hz), 7.61 (1H, dt, *J* = 7.5 and 1.3 Hz), 7.43 (1H, d, *J* = 8.5 Hz), 7.20 (1H, sl), 6.43 (1H, dd, *J* = 8.4 and 2.4 Hz), 6.34 (1H, d, *J* = 2.4 Hz), 5.46 (1H, s), 3.73 (6H, d, *J* = 2.2 Hz), 2.48 (1H, d, *J* = 16.5 Hz), 2.33 (1H, d, *J* = 16.5 Hz), 2.24 (1H, d, *J* = 16.4 Hz), 2.17 (1H, d, *J* = 16.4 Hz), 1.10 (3H, s), 0.98 (3H, s).^13^C NMR (75.0 MHz, CDCl_3_) δ (ppm): 195.2, 182.3, 180.3, 159.6, 158.9, 147.4, 137.2, 134.7, 132.8, 132.4, 132.3, 130.0, 126.5, 125.8, 125.0, 119.9, 104.3, 98.8, 55.4, 55.1, 50.7, 41.3, 32.6, 32.2, 29.4, 26.7.HRMS *m/z*: [M+Na]^+^ = 466.1638 (Calculated for C_27_H_25_NO_5_Na^+^ = 466.1630).

**4k**: 12-(2,5-dimethoxyphenyl)-3,3-dimethyl-3,4-dihydrobenzo[*b*]acridine-1,6,11(2*H*,5*H*,12*H*)-trione. Redsolid, m.p. 288–290 °C, yield 40%. IR(KBr) ν cm^−1^: 3343,2952, 1635, 1605, 1472, 1383, 1352, 1219, 1141, 1046, 1021, 720. ^1^H NMR (500.00 MHz, CDCl_3_) δ (ppm): 8.02 (1H, dd, *J* = 7.5 and 1.0 Hz), 7.99 (1H, dd, *J* = 7.6 and 1.1 Hz), 7.67 (1H, dt, *J* = 7.5 and 1.3 Hz), 7.61 (1H, dt, *J* = 7.5 and 1.3 Hz), 7.04 (1H, d, *J* = 3.0 Hz), 6.72 (1H, d, *J* = 8.8 Hz), 6.63–6.65 (1H, m), 5.51 (1H, s), 3.76 (6H, d, *J* = 8.8 Hz), 2.48 (1H, d, *J* = 16.5 Hz), 2.37 (1H, d, *J* = 16.5 Hz), 2.25 (1H, d, *J* = 16.4 Hz), 2.19 (1H, d, *J* = 16.4 Hz), 1.10 (3H, s), 0.99 (3H, s).^13^C NMR (75.0 MHz, CDCl_3_) δ (ppm): 195.2, 182.1, 180.2, 153.4, 152.4, 147.9, 137.2, 134.7, 133.6, 132.8, 132.5, 130.0, 126.6, 125.9, 120.1, 117.3, 112.8, 112.4, 111.0, 56.4, 55.6, 50.5, 41.3, 32.6, 32.4, 29.3, 26.9. HRMS *m/z*: [M+Na]^+^ = 466.1642 (Calculated for C_27_H_25_NO_5_Na^+^ = 466.1630).

**4l**: 12-(3,4-dimethoxyphenyl)-3,3-dimethyl-3,4-dihydrobenzo[*b*]acridine-1,6,11(2*H*,5*H*,12*H*)-trione. Redsolid, m.p. 238–239 °C, yield 30%. IR (KBr) ν cm^−1^: 3344,2968, 1664, 1636, 1604, 1590, 1484, 1384, 1351, 1223, 1130, 1018, 724. ^1^H NMR (500.00 MHz, CDCl_3_) δ (ppm): 8.01–8.03 (2H, m), 7.69 (1H, dt, *J* = 7.5 and 1.3 Hz), 7.62 (1H, dt, *J* = 7.5 and 1.3 Hz), 7.27 (1H, sl), 7.03 (1H, d, *J* = 2.0 Hz), 6.82 (1H, dd, *J* = 8.3 and 2.0 Hz), 6.69 (1H, d, *J* = 8.3 Hz), 5.34 (1H, s), 3.85 (3H, s), 3.75 (3H, s), 2.52 (1H, d, *J* = 16.7 Hz), 2.45 (1H, d, *J* = 16.7 Hz), 2.29 (1H, d, *J* = 16.4 Hz), 2.24 (1H, d, *J* = 16.4 Hz), 1.12 (3H, s), 1.05 (3H, s).^13^C NMR (125.0 MHz, CDCl_3_) δ (ppm): 195.2, 182.2, 180.0, 148.6, 147.7, 147.1, 137.8, 136.4, 134.8, 132.7, 130.0, 126.6, 125.9, 121.1, 120.1, 112.3, 112.2, 111.0, 55.8, 55.7, 50.7, 41.2, 33.9, 32.7, 29.3, 27.3.HRMS *m/z*: [M+Na]^+^ = 466.1639 (Calculated for C_27_H_25_NO_5_Na^+^ = 466.1630).

**4m**: 12-(3,4,5-trimethoxyphenyl)-3,3-dimethyl-3,4-dihydrobenzo[*b*]acridine-1,6,11(2*H*,5*H*,12*H*)-trione. Redsolid, m.p. 242–243 °C, yield 32%. IR (KBr) ν cm^−1^: 3136,2937, 1676, 1644, 1585, 1490, 1379, 1300, 1227, 1125, 999, 923, 731, 645.^1^H NMR (500.00 MHz, CDCl_3_) δ (ppm): 8.06 (1H, dd, *J* = 7.6 and 1.2 Hz), 7.72 (1H, dt, *J* = 7.5 and 1.3 Hz), 7.65 (1H, dt, *J* = 7.5 and 1.3 Hz), 7.20 (1H, sl), 6.62 (2H, s), 5.36 (1H, s), 3.80 (6H, s), 3.75 (3H, s), 2.56 (1H, d, *J* = 16.7 Hz), 2.46 (1H, d, *J* = 16.7 Hz), 2.32 (1H, d, *J* = 16.5 Hz), 2.28 (1H, d, *J* = 16.5 Hz), 1.15 (3H, s), 1.11 (3H, s). ^13^C NMR (125.0 MHz, CDCl_3_) δ (ppm): 195.1, 182.1, 180.0, 153.0, 147.3, 140.2, 136.4, 134.9, 132.8, 132.7, 130.1, 126.7, 126.0, 121.0, 111.9, 105.8, 60.6, 56.1, 50.7, 41.3, 34.6, 32.7, 29.5, 27.1. HRMS *m/z*: [M+Na]^+^ = 496.1741 (Calculated for C_28_H_27_NO_6_Na^+^ = 496.1736).

**5a**: 12-phenyl-3,4-dihydrobenzo[*b*]acridine-1,6,11(2*H*,5*H*,12*H*)-trione. Redsolid, m.p. 287–288 °C, yield 30%. IR (KBr) ν cm^−1^: 3335,2925, 1665, 1632, 1596, 1467, 1386, 1337, 1227, 1199, 1130, 997, 941, 775, 709.^1^H NMR (500.00 MHz, CDCl_3_) δ (ppm): 8.05 (1H, dd, *J* = 7.6 and 1.2 Hz), 8.03 (1H, dd, *J* = 7.6 and 1.2 Hz), 7.70 (1H, dt, *J* = 7.5 and 1.3 Hz), 7.64 (1H, dt, *J* = 7.5 and 1.3 Hz), 7.38 (2H, d, *J* = 7.2 Hz), 7.22 (2H, t, *J* = 7.5 Hz), 7.11 (1H, t, *J* = 7.3 Hz), 5.45 (1H, s), 2.58–2.70 (2H, m), 2.33–2.48 (2H, m), 2.01–2.14 (2H, m).^13^C NMR (75.0 MHz, CDCl_3_) δ (ppm): 195.2, 182.1, 180.0, 148.8, 145.0, 136.6, 134.8, 132.8, 132.7, 130.1, 128.3, 126.7, 126.0, 121.1, 113.3, 37.0, 34.4, 27.6, 21.0. HRMS *m/z*: [M+Na]^+^ = 378.1111 (Calculated for C_23_H_17_NO_3_Na^+^ = 378.1106).

**5b**: 12-(2-chlorophenyl)-3,4-dihydrobenzo[*b*]acridine-1,6,11(2*H*,5*H*,12*H*)-trione. Redsolid, m.p. 250–251 °C, yield 18%. IR (KBr) ν cm^−1^: 3290,2941, 1675, 1642, 1594, 1469, 1386, 1336, 1229, 1134, 999, 939, 722.^1^H NMR (500.00 MHz, CDCl_3_) δ (ppm): 8.05 (1H, dd, *J* = 7.6 and 1.2 Hz), 8.00 (1H, dd, *J* = 7.6 and 1.2 Hz), 7.69 (1H, dt, *J* = 7.5 and 1.3 Hz), 7.64 (1H, dt, *J* = 7.5 and 1.3 Hz), 7.52 (1H, dd, *J* = 7.7 and 1.6 Hz), 7.34 (1H, sl), 7.25 (1H, dd, *J* = 7.9 and 1.2 Hz), 7.16 (2H, dt, *J* = 7.5 and 1.3 Hz), 7.06 (1H, dt, *J* = 7.9 and 1.7 Hz), 5.70 (1H, s), 2.59–2.62 (2H, m), 2.34–2.38 (2H, m), 2.01–2.09 (2H, m).^13^C NMR (125.0 MHz, CDCl_3_) δ (ppm): 195.2, 182.1, 179.9, 149.3, 141.8, 137.3, 134.9, 133.7, 132.7, 130.0, 129.9, 127.9, 126.6, 126.5, 126.0, 119.6, 112.4, 36.9, 35.0, 27.6, 20.9. HRMS *m/z*: [M+Na]^+^ = 412.0730 (Calculated for C_23_H_16_ClNO_3_Na^+^ = 412.0716).

**5c**: 12-(3-chlorophenyl)-3,4-dihydrobenzo[*b*]acridine-1,6,11(2*H*,5*H*,12*H*)-trione. Redsolid, m.p. 217–218 °C, yield 25%. IR (KBr) ν cm^−1^: 3328,2959, 1637, 1592, 1471, 1382, 1356, 1230, 1190, 1174, 1129, 999, 957, 724, 683. ^1^H NMR (500.00 MHz, CDCl_3_) δ (ppm): 8.07 (1H, dd, *J* = 7.6 and 1.1 Hz), 8.03 (1H, dd, *J* = 7.6 and 1.1 Hz), 7.72 (1H, dt, *J* = 7.6 and 1.4 Hz), 7.66 (1H, dt, *J* = 7.6 and 1.4 Hz), 7.34–7.36 (1H, m), 7.27–7.28 (1H, m), 7.16–7.17 (2H, m), 5.42 (1H, s), 2.67–2.70 (2H, m), 2.56–2.59 (2H, m), 2.36–2.38 (2H, m).^13^C NMR (125.0 MHz, CDCl_3_) δ (ppm): 196.3, 182.0, 179.8, 146.2, 136.8, 134.9, 133.9, 132.9, 132.6, 130.0, 129.2, 128.0, 127.1, 126.9, 126.6, 126.1, 120.3, 116.3, 112.9, 36.8, 31.5, 27.1, 20.2. HRMS *m/z*: [M+Na]^+^ = 412.0713 (Calculated for C_23_H_16_ClNO_3_Na^+^ = 412.0716).

**5d**: 12-(4-chlorophenyl)-3,4-dihydrobenzo[*b*]acridine-1,6,11(2*H*,5*H*,12*H*)-trione. Redsolid, m.p. 230–231 °C, yield 20%. IR (KBr) ν cm^−1^: 3236,2949, 1720, 1670, 1642, 1591, 1467, 1390, 1357, 1229, 1131, 1086, 997, 952, 834, 722.^1^H NMR (500.00 MHz, CDCl_3_) δ (ppm): 8.05 (1H, dd, *J* = 7.5 and 1.0 Hz), 8.03 (1H, dd, *J* = 7.6 and 1.1 Hz), 7.71 (1H, dt, *J* = 7.6 and 1.3 Hz), 7.65 (1H, dt, *J* = 7.6 and 1.3 Hz), 7.32 (1H, d, *J* = 8.4 Hz), 7.18 (1H, d, *J* = 8.4 Hz), 5.41 (1H, s), 2.62–2.67 (2H, m), 2.40–2.47 (2H, m), 1.98–2.05 (2H, m). ^13^C NMR (75.0 MHz, CDCl_3_) δ (ppm): 195.2, 182.1, 179.9, 149.0, 143.5, 136.7, 134.9, 132.9, 132.6, 132.4, 130.0, 129.7, 128.4, 128.2, 126.7, 126.1, 120.6, 113.1, 37.0, 34.1, 27.6, 21.0. HRMS *m/z*: [M+H]^+^ = 390.0898 (Calculated for C_23_H_16_ClNO_3_H^+^ = 390.0897).

**5e**: 12-(2.4-dichlorophenyl)-3,4-dihydrobenzo[*b*]acridine-1,6,11(2*H*,5*H*,12*H*)-trione. Redsolid, m.p. 271–272 °C, yield 20%. IR (KBr) ν cm^−1^: 3275,2952, 1676, 1629, 1589, 1468, 1384, 1335, 1234, 1199, 1134, 996, 936, 722.^1^H NMR (500.00 MHz, CDCl_3_) δ (ppm): 8.06 (1H, dd, *J* = 7.5 and 1.0 Hz), 8.00 (1H, dd, *J* = 7.5 and 1.0 Hz), 7.71 (1H, dt, *J* = 7.5 and 1.3 Hz), 7.65 (1H, dt, *J* = 7.5 and 1.3 Hz), 7.48 (1H, d, *J* = 8.3 Hz), 7.35 (1H, sl), 7.27 (1H, d, *J* = 2.1 Hz), 7.15 (1H, dd, *J* = 8.3 and 2.1 Hz), 5.65 (1H, s), 2.59–2.64 (2H, m), 2.33–2.41 (2H, m), 1.97–2.10 (2H, m). ^13^C NMR (75.0 MHz, CDCl_3_) δ (ppm): 195.2, 182.1, 179.7, 149.5, 140.3, 137.3, 135.0, 134.3, 133.6, 130.0, 132.8, 132.5, 129.9, 129.7, 126.8, 126.6, 126.1, 119.1, 112.0, 36.9, 35.0, 27.6, 20.9. HRMS *m/z*: [M+H]^+^ = 424.0500 (Calculated for C_23_H_15_Cl_2_NO_3_H^+^ = 424.0507).

**5f**: 12-(4-fluorophenyl)-3,4-dihydrobenzo[*b*]acridine-1,6,11(2*H*,5*H*,12*H*)-trione. Redsolid, m.p. 255–256 °C, yield 32%. IR (KBr) ν cm^−1^: 3239,2943, 1675, 1643, 1625, 1592, 1505, 1467, 1393, 1337, 1228, 1136, 997, 935, 723.^1^H NMR (500.00 MHz, CDCl_3_) δ (ppm): 8.05 (1H, dd, *J* = 7.5 and 1.0 Hz), 8.02 (1H, dd, *J* = 7.5 and 1.0 Hz), 7.71 (1H, dt, *J* = 7.5 and 1.3 Hz), 7.65 (1H, dt, *J* = 7.5 and 1.3 Hz), 7.35 (2H, dd, *J* = 8.8 and 5.5 Hz), 7.30 (1H, sl), 6.90 (2H, t, *J* = 8.7 Hz), 5.42 (1H, s), 2.59–2.69 (2H, m), 2.32–2.47 (2H, m), 1.97–2.13 (2H, m).^13^C NMR (125.0 MHz, CDCl_3_) δ (ppm): 195.3, 182.1, 179.9, 161.5 (d, *J* = 244.9 Hz), 149.0, 140.9, 136.6, 134.9, 132.8, 132.6, 130.0, 129.8 (d, *J* = 8.0 Hz), 126.6, 126.0, 120.8, 115.1 (d. *J* = 21.3 Hz), 113.3, 37.0, 33.8, 27.5, 21.0. HRMS *m/z*: [M+H]^+^ = 374.1193 (Calculated for C_23_H_16_FNO_3_H^+^ = 374.1192).

**5g**: 12-(perfluorophenyl)-3,4-dihydrobenzo[*b*]acridine-1,6,11(2*H*,5*H*,12*H*)-trione. Redsolid, m.p. 295–296 °C, yield 30%. IR (KBr) ν cm^−1^: 3166,2941, 1678, 1651, 1633, 1590, 1487, 1390, 1358, 1232, 1137, 991, 956, 721.^1^H NMR (500.00 MHz, CDCl_3_) δ (ppm): 8.08 (1H, dd, *J* = 7.5 and 1.0 Hz), 8.02 (1H, dd, *J* = 7.5 and 1.0 Hz), 7.74 (1H, dt, *J* = 7.5 and 1.3 Hz), 7.68 (1H, dt, *J* = 7.5 and 1.3 Hz), 7.42 (1H, sl), 5.73 (1H, s), 2.57–2.66 (2H, m), 2.39–2.41 (2H, m), 2.01–2.11 (2H, m).^13^C NMR (125.0 MHz, CDCl_3_) δ (ppm): 195.1, 181.9, 179.4, 150.4, 137.8, 135.2, 133.1, 132.3, 129.9, 126.6, 126.3, 116.6, 109.8, 36.7, 27.5, 26.3, 20.9. HRMS *m/z*: [M+H]^+^ = 446.0796 (Calculated for C_23_H_12_F_5_NO_3_H^+^ = 446.0816).

**5h**: 12-(4-bromophenyl)-3,4-dihydrobenzo[*b*]acridine-1,6,11(2*H*,5*H*,12*H*)-trione. Redsolid, m.p. 257–258 °C, yield 30%. IR (KBr) ν cm^−1^: 3268,2951, 1725, 1673, 1644, 1592, 1469, 1385, 1335, 1234, 1198, 1130, 997, 944, 721.^1^H NMR (500.00 MHz, CDCl_3_) δ (ppm): 8.05 (1H, dd, *J* = 7.5 and 1.0 Hz), 8.02 (1H, dd, *J* = 7.6 and 1.1 Hz), 7.71 (1H, dt, *J* = 7.5 and 1.3 Hz), 7.65 (1H, dt, *J* = 7.5 and 1.3 Hz), 7.32–7.34 (2H, m), 7.27–7.28 (2H, m), 5.40 (1H, s), 2.62–2.66 (2H, m), 2.34–2.47 (2H, m), 2.06–2.12 (2H, m). ^13^C NMR (125.0 MHz, CDCl_3_) δ (ppm): 182.0, 179.9, 148.9, 144.0, 136.7, 135.0, 132.9, 132.6, 131.4, 130.1, 130.0, 126.7, 126.1, 120.6, 120.5, 113.0, 37.0, 34.0, 27.6, 21.0. HRMS *m/z*: [M+H]^+^ = 434.0385 (Calculated for C_23_H_16_BrNO_3_H^+^ = 434.0392).

**5i**: 12-(2,4-dimethylphenyl)-3,4-dihydrobenzo[*b*]acridine-1,6,11(2*H*,5*H*,12*H*)-trione. Redsolid, m.p. 263–264 °C, yield 20%. IR (KBr) ν cm^−1^: 3294,2956, 1731, 1674, 1644, 1629, 1593, 1472, 1386, 1336, 1233, 1135, 997, 947, 723. ^1^H NMR (500.00 MHz, CDCl_3_) δ (ppm): 8.04 (1H, dd, *J* = 7.5 and 1.0 Hz), 7.98 (1H, dd, *J* = 7.5 and 1.0 Hz), 7.67 (1H, dt, *J* = 7.5 and 1.3 Hz), 7.62 (1H, dt, *J* = 7.5 and 1.3 Hz), 7.23 (1H, sl), 6.99 (1H, d, *J* = 7.8 Hz), 6.90 (1H, s), 6.80 (1H, d, *J* = 7.8 Hz), 5.41 (1H, s), 2.90 (3H, s), 2.60–2.67 (2H, m), 2.32–2.41 (2H, m), 2.18 (3H, s), 2.06–2.08 (2H, m). ^13^C NMR (125.0 MHz, CDCl_3_) δ (ppm): 195.4, 182.3, 180.2, 148.2, 142.5, 136.5, 136.1, 134.7, 132.7, 130.9, 130.0, 128.5, 126.8, 126.6, 125.9, 122.8, 115.2, 37.0, 30.6, 27.6, 20.9, 19.8, 14.1. HRMS *m/z*: [M+Na]^+^ = 406.1411 (Calculated for C_25_H_21_NO_3_Na^+^ = 406.1419).

**5j**: 12-(2,4-dimethoxyphenyl)-3,4-dihydrobenzo[*b*]acridine-1,6,11(2*H*,5*H*,12*H*)-trione. Redsolid, m.p. 252–253 °C, yield 28%. IR (KBr) ν cm^−1^: 3359,2959, 1670, 1633, 1604, 1471, 1396, 1361, 1230, 1022, 931, 858, 721.^1^H NMR (500.00 MHz, CDCl_3_) δ (ppm): 8.03 (1H, dd, *J* = 7.5 and 1.0 Hz), 7.99 (1H, dd, *J* = 7.5 and 1.0 Hz), 7.67 (1H, dt, *J* = 7.5 and 1.3 Hz), 7.61 (1H, dt, *J* = 7.5 and 1.3 Hz), 7.41 (1H, d, *J* = 8.4 Hz), 7.23 (1H, sl), 6.43 (1H, dd, *J* = 8.4 and 2.4 Hz), 6.36 (1H, d, *J* = 2.4 Hz), 5.49 (1H, s), 3.75 (3H, s), 3.72 (3H, s), 2.55–2.58 (2H, m), 2.29–2.40 (2H, m), 1.94–2.07 (2H, m).^13^C NMR (125.0 MHz, CDCl_3_) δ (ppm): 195.4, 182.3, 180.3, 159.7, 159.1, 137.1, 134.7, 132.8, 132.5, 132.2, 130.0, 126.6, 125.8, 125.5, 120.2, 112.3, 104.5, 99.2, 55.7, 55.1, 36.9, 32.0, 27.6, 21.1. HRMS *m/z*: [M+H]^+^ = 416.1495 (Calculated for C_25_H_21_NO_5_H^+^ = 416.1498).

**5k**: 12-(2,5-dimethoxyphenyl)-3,4-dihydrobenzo[*b*]acridine-1,6,11(2*H*,5*H*,12*H*)-trione. Redsolid, m.p. 219–220 °C, yield 35%. IR (KBr) ν cm^−1^: 3353,2946, 1669, 1639, 1604, 1591, 1481, 1392, 1355, 1225, 1045, 1000, 934, 722. ^1^H NMR (500.00 MHz, CDCl_3_) δ (ppm): 8.03 (1H, dd, *J* = 7.5 and 1.0 Hz), 7.99 (1H, dd, *J* = 7.5 and 1.0 Hz), 7.67 (1H, dt, *J* = 7.5 and 1.3 Hz), 7.61 (1H, dt, *J* = 7.5 and 1.3 Hz), 7.32 (1H, sl), 7.02 (1H, d, *J* = 3.0 Hz), 6.75 (1H, d, *J* = 8.8 Hz), 6.64 (1H, dd, *J* = 8.8 and 3.0 Hz), 5.54 (1H, s), 3.78 (3H, s), 3.75 (3H, s), 2.57–2.60 (2H, m), 2.28–2.42 (2H, m), 1.94–2.07 (2H, m). ^13^C NMR (75.0 MHz, CDCl_3_) δ (ppm): 195.2, 182.1, 180.2, 153.5, 152.5, 149.1, 137.1, 134.7, 134.5, 132.8, 132.5, 130.0, 126.6, 125.8, 120.3, 117.3, 113.2, 112.6, 112.5, 56.9, 55.5, 37.0, 32.1, 27.6, 21.0. HRMS *m/z*: [M+H]^+^ = 416.1499 (Calculated for C_25_H_21_NO_5_H^+^ = 416.1498).

**5l**: 12-(3,4-dimethoxyphenyl)-3,4-dihydrobenzo[*b*]acridine-1,6,11(2*H*,5*H*,12*H*)-trione. Redsolid, m.p. 228–230 °C, yield 30%. IR (KBr) ν cm^−1^: 3347,2942, 1637, 1604, 1513, 1462, 1389, 1337, 1232, 1131, 1021, 945, 812, 718. ^1^H NMR (500.00 MHz, CDCl_3_) δ (ppm): 8.02–8.05 (2H, m), 7.70 (1H, dt, *J* = 7.5 and 1.3 Hz), 7.64 (1H, dt, *J* = 7.5 and 1.3 Hz), 7.27 (1H, sl), 7.07 (1H, d, *J* = 2.0 Hz), 6.78 (1H, dd, *J* = 8.3 and 2.0 Hz), 6.69 (1H, d, *J* = 8.3 Hz), 5.39 (1H, s), 3.87 (3H, s), 3.76 (3H, s), 2.59–2.68 (2H, m), 2.35–2.49 (2H, m), 2.02–2.13 (2H, m). ^13^C NMR (75.0 MHz, CDCl_3_) δ (ppm): 195.5, 180.1, 178.8, 148.7, 147.8, 137.9, 136.4, 134.8, 132.8, 130.1, 126.7, 126.0, 125.7, 121.1, 119.9, 118.1, 113.4, 112.4, 111.0, 55.9, 55.7, 37.1, 33.8, 27.6, 21.1. HRMS *m/z*: [M+Na]^+^ = 418.1312 (Calculated for C_25_H_21_NO_5_Na^+^ = 418.1317).

**5m**: 12-(3,4,5-trimethoxyphenyl)-3,4-dihydrobenzo[*b*]acridine-1,6,11(2*H*,5*H*,12*H*)-trione. Redsolid, m.p. 236–237 °C, yield 35%. IR (KBr) ν cm^−1^: 3235,2950, 1673, 1633, 1592, 1466, 1381, 1358, 1227, 1092, 996, 936, 723.^1^H NMR (500.00 MHz, CDCl_3_) δ (ppm): 8.00–8.02 (2H, m), 7.68 (1H, dt, *J* = 7.5 and 1.3 Hz), 7.62 (1H, dt, *J* = 7.5 and 1.3 Hz), 7.41 (1H, sl), 6.60 (2H, s), 5.38 (1H, s), 3.79 (3H, s), 3.72 (3H, s), 2.59–2.70 (2H, m), 2.45–2.50 (1H, m), 2.35–2.41 (1H, m), 2.00–2.13 (2H, m).^13^C NMR (125.0 MHz, CDCl_3_) δ (ppm): 195.4, 182.1, 179.9, 153.0, 149.1, 140.4, 136.9, 136.5, 134.8, 132.8, 132.6, 130.0, 126.6, 126.0, 120.7, 112.9, 105.7, 60.6, 56.1, 37.0, 34.2, 27.5, 21.1.HRMS *m/z*: [M+H]^+^ = 446.1596 (Calculated for C_26_H_23_NO_6_H^+^ = 446.1604).

### 3.2. Biological Assays

Cells and reagents. Human SCC-4, SCC-9, and SCC-25 cells, derived from a human oral tongue SCC (squamous cell carcinoma) were obtained from the ATCC (CRL-1624, CRL-1629, and CRL-1628, respectively) and maintained in 1:1 DMEM/F12 (Dulbecco’s modified Eagle medium and Ham’s F12 medium; Gibco (Thermo Fisher, Waltham, MA, USA)) supplemented with 10% (*v*/*v*) FBS (fetal bovine serum; Invitrogen, Thermo Fisher, Waltham, MA, USA) and 400 ng/mL hydrocortisone (Sigma-Aldrich Co., St. Louis, MO, USA). Primary normal human gingival fibroblasts were obtained from the ATCC (PCS-201-018) and maintained in DMEM supplemented with 10% (*v*/*v*) FBS and were used in a maximum of six passages. The cells were grown in a humidified environment containing 5% CO_2_ at 37 °C. For all biological experiments, the compounds and controls lapachol, shikonin, and doxorubicin were solubilized in 100% DMSO (all Sigma-Aldrich) to a final concentration of 10 mM. Carboplatin stock was prepared in water (Fauldcarbo^®^; LibbsFarmacêutica, São Paulo, SP, Brazil) and was used as a standard anticancer compound.

Cell viability assay (cytotoxicity) and compound stability assay. The viability of SCC cell lines and primary human fibroblast cells was evaluated using the MTT assay as in [48]. Briefly, the cells were grown in triplicates in 96-well plates (5 × 10^3^ cells/well) until confluence. Then, the medium was removed, fresh medium was added, and the cells were returned to the incubator in the presence of different compounds. DMSO at the same concentrations was used as a 100% cell viability control. After 48 h, the cells were incubated with 5 mg/mL MTT reagent (3-(4,5-dimethyl-2-thiazolyl)-2,5-diphenyl-2-H-tetrazolium bromide) (Sigma-Aldrich Co., St. Louis, MO, USA) for 3.5 h. After that, formazan crystals were dissolved in MTT solvent solution (DMSO/methanol 1:1 *v*/*v*) and the absorbance at 560 nm was evaluated using an EPOCH microplate spectrophotometer (BioTek Instruments, Winooski, VT, USA) with the background absorbance at 670 nm subtracted. Each of the 16 compounds was tested at six or seven different concentrations, ranging from 0.3 µM to 200 µM in cancer cell lines (SCC-4, SCC-9, and SCC-25) and initially at 2 × IC_50_ (two times the calculated IC_50_ for each compound) in primary normal human gingival fibroblasts. Afterwards, only compounds that showed less than 25% of cytotoxicity in fibroblasts had their IC_50_ calculated to determine their selectivity. Controls (carboplatin, lapachol, shikonin, and doxorubicin) were tested at six or seven different concentrations ranging from 0.05 µM to 1000 µM in cancer cells and normal cells, depending on the compound.

For the stability assay, compound **4e** and the control carboplatin were prepared at a concentration of 2 × IC_50_ and stored in the incubator for 0, 1, 3, 6, 12, 24, or 48 h before the treatment of SCC9 cells (5 × 10^3^ cells/well). All wells were treated at the same time and incubated for a further 48 h in the presence of the different heat-treated compounds. After this time, an MTT assay was performed at the end of the experiment to determine their biological activity (cytotoxicity).

Hemolysis assay. To determine the surfactant power of substances in biological membranes, a hemolysis assay was performed using human blood approved by the Research Ethics Committee of Universidade Federal Fluminense (CAAE: 43134721.4.0000.5626). Erythrocytes were collected for centrifugation at 1500 rpm for 15 min, washed with PBS (phosphate-buffered saline) supplemented with 10 mM glucose, and counted in an automatic cell counter (Thermo Fisher, Waltham, MA, USA). Erythrocytes were then plated in 96-well plates at a concentration of 4 × 10^8^ cells/well in triplicates, and 10 µL of compounds was added at a final concentration of 300 µM in PBS with glucose (final volume 100 µL). In total, 10 µL of PBS was used as a negative control and 10 µL of PBS with 0.1% Triton ×100 as a positive control. Data reading was performed with EPOCH (BioTek Instruments, Winooski, VT, USA) at an absorbance of 540 nm, and the statistical data were generated using the GRAPHPAD Prism 5.0 program (Intuitive Software for Science, San Diego, CA, USA).

In vivo acute toxicity study. The acute toxicity study for compound **4e** was performed in twelve-week-old C57BL/6 female mice via intraperitoneal injection and was approved by the University Animal Ethics Board under registration number 982. All experiments were performed in accordance with Brazilian guidelines and regulations. Dosing and analysis were performed according to 423 OECD guidelines and reviewed by [22]. Each animal group had *n* = 3 or 4 (as indicated) and received only one intraperitoneal injection (Day 0) of compound **4e** dissolved in 3 mL PBS and 3% DMSO. The control group animals received only 3% DMSO in PBS. The first dose of the compound was 100 mg/kg. Subsequent dose levels (200 mg/kg and 400 mg/kg) were determined based on the result obtained from the previous dosing. The animals were examined every day, twice a day, for mortality and morbidity for 14 days, when all animals were anesthetized (ketamine 100 mg/kg and xylazine 10 mg/kg) followed by cervical dislocation. Gross necropsy and histology of the main organs were performed. The animals’ body weights and average food consumption were measured every 7 days. As an indication of morbidity, the following signs were assessed: tremors; convulsion; salivation; diarrhea; lethargy; coma; pain signs; increased rear arching; and mobility impairment. The necropsy included an examination of the external characteristics of the carcass; external body orifices; the abdominal, thoracic, and cranial cavities; organs/tissues of the liver, thymus, right kidney, right testicle, heart, and lung. Major organs were analyzed by histopathology. For more details, see Supplementary Methods.

Video microscopy. SCC9 cells were plated in 35 mm culture dishes the day before the experiment, treated with 2 × IC_50_ of compound **4e** or DMSO (control), and transferred to a culture chamber adapted to a Nikon Eclipse TE300 microscope (Nikon, Melville, NY, USA) under controlled conditions of CO_2_ and temperature (5% and 37 °C, respectively). For 72 h, phase-contrast images of the same field were captured every minute using a Hamamatsu C2400 CCD camera (Hamamatsu, Japan). The images of each experimental condition were integrated into videos using ImageJ software (National Institute of Health, Bethesda, MD, USA), and different times (indicated) were selected according to the morphological change observed during the treatment.

ROS production. The hydrogen peroxide (H_2_O_2_) luminescence assay was performed using the ROS-Glo™ H_2_O_2_ assay kit (Promega Corporation, Madison, WI, USA). To carry out the experiment, 1 × 10^4^ SCC9 cells per well were plated in a 96-well plate and, after 24 h of incubation, the medium was removed and the wells were treated with 2 × IC_50_ of compound **4e**, or with DMSO and doxorubicin controls. A plate with wells without cells was used as a control. The cells and cell-free wells remained in treatment at the times of interest and, 2 h before the completion of the times, the well volumes were removed to reduce to 40 µL per well. Then, the positive control of menadione, a polycyclic aromatic ketone based on 1,4-naphthoquinone, was used to treat its respective well, and H_2_O_2_ substrate was added for all treatments, followed by the incubation of the plate for the 2 h remaining. After the times of 3, 6, 24, and 48 h, the detection solution was added, and the plates remained at room temperature for 20 min, following the reading in the luminometer.

Autophagy assay. To determine if compound **4e** induces autophagy, SCC9 cells were stably transduced with LC3-GFP expressing plasmid. For the generation of SCC9 cell lines harboring LC3-GFP, HEK293FT cells were transfected with 3 µg of each helper plasmids (pMDLglpRRE, pHCMV g, and pRSVrev) and 8 µg of the pLV-CMV-SV40-Puro-GFP-LC3 using calcium phosphate and 25 µM chloroquine. Twenty-four hours later, a lentivirus-containing medium was harvested, supplemented with 8 µg/mL of Polybrene, and used to transduce SCC9 cells. Twenty-four hours later, the transduced cells were selected with 5 µg/mL of Puromycin for 7 days. The cells were then cultured with 2.5 µg/mL Puromycin for further experiments. The lentiviral construct encoding pLV-CMV-SV40-Puro-GFP-LC3 was a gift from Dr. SilvyaStuchi Maria Engler. Briefly, 5 × 10^4^ SCC9-LC3-GFP cells were plated at 24-wells plates and 24 h later treated with 2 × IC_50_ of compound **4e** or DMSO at the same concentration and incubated for a further 48 h before visualization with an inverted fluorescence Zeiss Axio Observer microscope. As a positive control, calcium phosphate precipitates (CPP) were prepared by adding CaCl_2_ dropwise to Na_2_HPO_4_ (in Hepes, pH 7.05) as previously described [45]. CPP 20% (*v*/*v*) was administrated to the cells 4–6 h before analysis.

Cell cycle and SubG1 analysis. To evaluate the action of compound **4e** on the cell cycle and DNA fragmentation, cells of the SCC9 cell line were plated in a 6-well plate (5 × 10^5^ cells/well). After 48 (cell cycle) or 72 h (DNA fragmentation) of treatment, the cells were trypsinized and stained with propidium iodide (75 µM) in the presence of NP-40. The DNA content was analyzed by collecting 10,000 events using a FACScalibur flow cytometer. The data were analyzed using CellQuest (BD Biosciences, Franklin Lakes, NJ, USA) and FlowJo (Tree Star Inc., Ashland, OR, USA) software as in [49].

Phosphatidylserine exposure analysis (apoptosis). Cells of the SCC9 cell line were plated in 6-well plates (5 × 10^5^ cells/well), trypsinized 48 h after treatment, labeled using the Annexin V-FITC Apoptosis Detection Kit according to the manufacturer’s protocol (#BMS500FI/300, Invitrogen), and analyzed by FACScalibur flow cytometry as in [50]. Furthermore, 5 × 10^4^ SCC9 cells were plated in a 24-well plate containing 1 mL of DMEM/F12 with 10% FBS per well. CellEvent™ Caspase-3/7 Reagent (#R37111, Invitrogen) was diluted in a culture medium according to the manufacturer’s instructions. Twenty-four hours after plating, the cells were treated with Caspase-3/7 Reagent and 2× IC_50_ of compound **4e** or DMSO as a control. The cells were analyzed by flow cytometry at the time of 48 h of treatment.

Statistical analysis, IC_50_ calculation. The data are presented as means ±\SD. IC_50_ values for the MTT assays were obtained by nonlinear regression using the GRAPHPAD 5.0 program (Intuitive Software for Science, San Diego, CA, USA) from at least three independent experiments. A dose–response (inhibitor) vs. response curve using the least squares method was used to determine the IC_50_, SD, and R^2^ of the data. The selectivity index was calculated as SI = IC_50_ of the compound in normal oral fibroblast cells/IC_50_ of the same compound for each oral cancer cell line (SCC-4, SCC-9, and SCC-25) and the mean was calculated when indicated.

### 3.3. In Silico Studies

Reverse Docking. The three-dimensional structures of both the R and S enantiomers of compound 4e were constructed using the software Spartan’10 (Wavefunction Inc., Irvine, CA, USA). Initially, a conformational analysis was carried out using the MMFF force field. The lowest-energy conformer was submitted to a geometry optimization step using the semi-empirical method RM1. Finally, an energy calculation was performed using the density functional theory method with the B3LYP/6-31G* basis set. The same procedure was carried out to obtain the structures of lapachol, shikonin, and 1,4-naphthoquinone (1,4-NQ), which were used as controls in the docking studies. After conducting a literature search, the anticancer targets of lapachol and other naphthoquinones were selected for the computational target fishing strategy. The three-dimensional structures of the chosen targets were obtained from the Protein Data Bank (PDB) under the codes: ribosomal protein S6 kinase 2 (RSK2; PDB 4NW6), human M2 pyruvate kinase (PKM2; PDB 3SRD), DNA-binding domain of topoisomerase I (PDB 1K4T), topoisomerase IIα (PDB 5GWK) and topoisomerase IIβ (PDB 3QX3), and the ATPase domain of topoisomerase IIα (PDB 1ZXM). For the reverse docking screening, we employed Autodock Tools 1.5.7 and Autodock Vina 1.1.2 [51]. The preparation of proteins and ligands, as well as the docking protocols, are reported elsewhere by our group [52], except for the docking parameters for RSK2. The docking method of this protein was validated by redocking a 2-amino-7-substituted benzoxazole derivative (**2NS**) co-crystallized with the N-terminal of RSK2 (PDB code 4NW6). The grid box was centered on L200 (CG atom), and its dimensions were set to 24 × 18 × 20 Å^3^. The search parameters were kept as default. The docked and crystalized binding poses of this inhibitor were superimposed and showed an RMSD value of 0.58 Å, proving the excellent prediction accuracy of the docking protocol. In all studies, the ligand’s pose with the lowest binding energy obtained in the docking was selected for visual inspection and interaction analysis, which were performed using Discovery Studio Visualizer 2019 (Dassault Systèmes BIOVIA, San Diego, CA, USA, 2019) and Pymol v. 1.2r2 (The PyMOL Molecular Graphics System, Version 1.2r2, Schrödinger, LLC, New York, NY, USA).

Prediction of toxicity and pharmacokinetic properties. The Smile structure of the compounds evaluated was obtained using the ChemDraw software (https://www.perkinelmer.com/category/chemdraw (accessed on 20 July 2022). The values of the calculated octanol-water partition coefficient (cLogP), molecular weight (MW), number of hydrogen bond acceptors (nON), number of hydrogen bond donors (nOH/NH), and topological polar surface area (TPSA) were calculated using the SwissADME web server (http://www.swissadme.ch/ (accessed on 20 July 2022). Other predictions, such as absorption, distribution, and metabolism, were performed with the admetSAR 2.0 server (http://lmmd.ecust.edu.cn/admetsar2 (accessed on 20 July 2022). The most selective compound was analyzed, and carboplatin and doxorubicin were used as controls.

## 4. Conclusions

In summary, twenty-six new synthetic acridine-core naphthoquinone compounds were synthesized, employing a multicomponent reaction. Sixteen of these compounds were tested for their antitumor potential in OSCC cells. Ten of them were not evaluated due to their insolubility. Compound **4e** was highly cytotoxic (29.99 µM) and selective (SI 2.93) among those tested and at levels comparable and superior to chemotherapeutic controls. Besides, compound **4e** proved to be well-tolerated in animals at all doses tested. Mechanistically, this compound is possibly able to bind to and inhibit enzymes important for tumor progression, such as RSK2, PKM2, and topoisomerase IIα. Our data also demonstrate that compound **4e** promotes cell death by apoptosis in the OSCC. Importantly, compound **4e** presented a pharmacological profile within desirable parameters for drug development, showing promise for future preclinical trials.

## Figures and Tables

**Table 1:** Determination of IC_50_ of acridine-core naphthoquinone compounds. SCC9 (OSCC cell) were treated with the indicated compounds for 48 h and cell viability was determined by MTT assay. Results from at least 3 independent experiments. SD = standard deviation.

Compounds	SCC9—Oral Cancer	
	IC_50_ (µM)	S.D.
**4c**	14.69	0.11
**4d**	51.57	0.18
**4e**	14.71	0.12
**4h**	5.86	0.15
**4j**	4.10	0.07
**4k**	N.D.	N.D.
**4l**	4.46	0.07
**4m**	2.06	0.07
**5c**	8.93	0.09
**5d**	2.21	0.12
**5e**	4.75	0.06
**5h**	7.63	0.09
**5j**	6.84	0.14
**5k**	7.20	0.08
**5l**	5.90	0.14
**5m**	3.32	0.13
Doxorubicin	7.8	0.10
Carboplatine	250.0	0.14
Lapachol	58.32	0.06
Shikonin	1.22	0.02

**Table 2:** Characterization of the most selective acridine-core naphthoquinone compounds in other OSCC cells. The IC_50_ (μM) of three different OSCC cell lines (SCC4, SCC9, and SCC25) for compounds **4e**, **4j,** and **4m** were calculated. S.I. = IC_50_ of the compound in cells of normal oral fibroblasts/IC_50_ of the same compound for each oral cancer cell line.

COMPOUND	Oral Tumor Cells									Primary Gingival Fibroblast	
	SCC9			SCC25			SCC4				
	IC_50_	S.D.	S.I.	IC_50_	S.D.	S.I.	IC_50_	S.D.	S.I.	IC_50_	S.D.
**4e**	14.71	0.12	6.0	21.52	0.07	4.1	53.75	0.09	1.6	87.99	0.07
**4j**	4.10	0.07	5.7	11.50	0.05	2.0	18.86	0.09	1.2	23.56	0.08
**4m**	2.06	0.07	3.8	7.41	0.06	1.1	10.99	0.65	0.7	7.86	0.04
**Doxorubicin**	7.8	0.10	1.6	1.27	0.06	9.7	4.56	0.03	2.7	12.30	0.20
**Carboplatin**	250.0	0.14	2.2	186.3	0.06	3.0	180.4	0.03	3.1	559.6	0.06
**Lapachol**	58.32	0.06	1.8	93.81	0.03	1.1	76.93	0.08	1.4	105.6	0.15
**Shikonin**	1.22	0.02	2.0	2.47	0.03	1.0	3.19	0.03	0.8	2.48	0.03

All experiments are results of at least three independent experiments. SD = standard deviation.

**Table 3:** Acute toxicity study: morbidity, mortality, gross organs necropsy, and histology of mice treated with compound **4e**. For more information and results, see supplemental Table S1.

Treatment	Dose mg/kg ^a^	Change in Body Weight	Change in Food Consumption	Morbidity ^b^	Mortality	Gross Necropsy ^c^	Histology ^d^
Control	0 N = 3	Absent	Absent	Absent	Absent	No alteration	Normal
**4e**	100 N = 3	Absent	Absent	Absent	Absent	No alteration	No significant alteration in comparison to control
	200 N = 4	Absent	Absent	Absent	Absent	No alteration	Presence of mild Perivascular and periportal lymphocyte focus and Bronchus-Associated Lymphoid Tissue (BALT)
	400 N = 4	Absent	Absent	Absent	Absent	No alteration	Presence of mild Perivascular and periportal lymphocyte focus and Bronchus-Associated Lymphoid Tissue (BALT)

^a^ Dose level for each subsequent group was determined based on the result after the previous lower dosing. N stands for the number of animals used in each experiment. ^b^ Morbidity symptoms were analyzed every day, two times a day, and were considered as: tremors, convulsion, salivation, diarrhea, lethargy, coma, signs of pain, and mobility defect. ^c^ Gross organ necropsy of the liver, thymus, right kidney, right testicle, heart, major lymph nodes, and lung. ^d^ Histopathology of the lung, kidney, heart, liver, and spleen was accessed by a trained pathologist and are summarized here.

**Table 4:** Physicochemical descriptors of compound **4e** and the control chemotherapy drugs doxorubicin and carboplatin.

Compounds	cLogP	nON	nOH/NH	MW	Lipinski’s Violations ^a^	TPSA (Å^2^)
**4e**	3.37	3	1	452.3	0	63.2
Doxorubicin	−2.10	12	6	543.3	3	206.1
Carboplatin	−1.79	6	4	371.2	0	126.6

^a^ Number of violations to the Lipinski “rule of 5”: cLogP ≤ 5; MW, molecular weight ≤ 500; nON, number of hydrogen bond acceptors ≤ 10; and nOH/NH, number of hydrogen bond donors ≤ 5.

**Table 5:** Predicted pharmacokinetic properties of compound **4e** and the chemotherapeutic agents, carboplatin, and doxorubicin, using the admetSAR 2.0 server.

ADMET	4e	Carboplatin	Doxoribicin
Oral Bioavailability	+0.66	−0.60	−0.91
P-glycoprotein inhibitor	−0.44	−0.99	−0.92
P-glycoprotein substrate	−0.72	−0.99	+0.95

## Data Availability

Not applicable.

## Supplementary Materials

The following supporting information can be downloaded at: https://www.mdpi.com/article/10.3390/molecules27165148/s1, Figure S1. Acute toxicity study shows the mean body weight variation (Figure S1A) and food consumption (Figure S1B); Figure S2. Differences in cell cycle distribution; Table S1: Average histopathological findings of 3 animals’ group (4 animals at 200 and 400 mg/kg) treated with indicated compound and concentration. Legend: +: Discreet alteration; ++: Mild alteration; +++: High degree of alteration; N: No; Y: Yes; N/A: No alteration.; Table S2. Molecular interactions of compound 4e with the putative targets predicted by reverse docking studies and comparison to known inhibitors used as reference compounds; Table S3. Residues comprising the ATP binding site of the predicted targets of compound 4e. Binding site residues were defined as the residues within a 5 Å distance around cocrystallized Ligands; Video S1: acridinas; Supplementary Spectral data SI: IR, NMR, and high-resolution mass spectrometry. References [24,25,26,27,28,29,30,31,53,54,55,56] are cited in the Supplementary Materials.

## Sample Availability

Samples of the compounds are not applicable.
