# Supplementary material for: Pro-Apoptotic Antitumoral Effect of Novel Acridine-Core Naphthoquinone Compounds against Oral Squamous Cell Carcinoma
Source: Molecules. 2022 Aug 12;27(16):5148. doi: 10.3390/molecules27165148 (PMC9415509; doi:10.3390/molecules27165148)
Supplement: Supplementary file 1 [file molecules-27-05148-s001.zip › Supplementary Biological Assays.pdf]

SUPPLEMENTARY MATERIALS

Pro-apoptotic Antitumoral Effect of Novel Acridine-Core Naphthoquinone Compounds Against Oral Squamous Cell Carcinoma

Bruna Costa Zorzanelli[1,‡], Gabriel Ouverney [2,‡], Fernanda P. Pauli[1], Anna Carolina Carvalho da Fonseca [3], Elan Cardozo Paes de Almeida [2], Danielle Gonçalves de Carvalho [4], Patricia Abrão Possik [4], Vitor Won-Held Rabelo [5], Paula Alvarez Abreu [5], Bruno Pontes [6], Vitor Francisco Ferreira [7], Luana da Silva Magalhães Forezi [1], Fernando de Carvalho da Silva [1,]*and Bruno Kaufmann Robbs [2,]*

[1] Universidade Federal Fluminense, Departamento de Química Orgânica, Instituto de Química, Campus do Valonguinho, CEP 24020-150, Niterói-RJ, Brasil.

[2] Universidade Federal Fluminense, Campus Universitário de Nova Friburgo, Departamento de Ciência Básica, CEP 28625-650, Nova Friburgo-RJ, Brasil.

[3] Universidade Federal Fluminense, Programa de Pós-graduação em Odontologia, Instituto de Saúde de Nova Friburgo, CEP 28625-650, Nova Friburgo-RJ, Brasil.

[4] Brazilian National Cancer Institute, Program of Immunology and Tumor Biology, Division of Experimental and Translational Research, Rio de Janeiro, RJ, 20231-050, Brazil.

[5] Universidade Federal do Rio de Janeiro, Instituto de Biodiversidade e Sustentabilidade, CEP 27965-045, Macaé-RJ, Brasil.

[6] Universidade Federal do Rio de Janeiro, Instituto de Ciências Biomédicas, CEP 21941-902, Rio de Janeiro-RJ, Brasil.

[7] Universidade Federal Fluminense, Faculdade de Farmácia, Departamento de Tecnologia Farmacêutica, CEP 24241-000, Niterói-RJ, Brasil.

‡ Authors contributed equally

Corresponding authors:

Chemistry: Fernando de Carvalho da Silva, Universidade Federal Fluminense, Department of Organic Chemistry, Institute of Chemistry, Campus do Valonguinho, CEP 24020-150, Niterói-RJ, Brazil

Biological Assays: Bruno K. Robbs, Fluminense Federal University, Department of Basic Sciences, Nova Friburgo Health Institute, CEP 28625-650, Nova Friburgo-RJ, Brazil.

Biological Assays

Supplementary Results and Methods

Acute toxicity

Histopathology

Fragments of the lungs, heart, spleen, liver and kidneys of the animals of the different groups were collected. Fragments were fixed in 10% buffered formalin solution, dehydrated in decreasing dilutions of alcohol, diaphanized, and embedded in paraffin. The blocks were cut at 5 µm and the sections stained with routine staining Hematoxylin-Eosin (HE). The reading of the slides was performed in Nikon Eclipse E-200 microscope at different magnifications (40 X, 100 X, 200 X and 400 X) and the morphological alterations of each organ were duly observed and noted.

Table S1: Average histopathological findings of 3 animals' group (4 animals at 200 and 400 mg/kg) treated with indicated compound and concentration. Legend: +: Discreet alteration; ++: Mild alteration; +++: High degree of alteration; N: No; Y: Yes; N/A: No alteration.

Organ	Histopathological findings	0 mg/kg			100 mg/kg			200 mg/kg				400 mg/kg			
		**Control**			**4e**			**4e**				**4e**			
Lung	Alveolar edema	N	N	N	N	N	N	N	N	N	N	N	Y	N	N
	Arterial and venous hyperemia	N	N	+	N	N	+	+	+	+	++	++	++	+	+
	Compensatory emphysema	N	N	N	N	N	N	N	N	N	N	N	N	N	N
	Obvious regional lymph node	N/A	N/A	N/A	N/A	N/A	N/A	N/A	N/A	N/A	N/A	N/A	N/A	N/A	N/A
	Bronchus-Associated Lymphoid Tissue (BALT)	+	+	+	+	+	+	++	++	+	++	+	++	+	++
Heart	Myocardium	N/A	N/A	N/A	N/A	N/A	N/A	N/A	N/A	N/A	N/A	N/A	N/A	N/A	N/A
	Valves	N/A	N/A	N/A	N/A	N/A	N/A	N/A	N/A	N/A	N/A	N/A	N/A	N/A	N/A
	Base Vessels	N/A	N/A	N/A	N/A	N/A	N/A	N/A	N/A	N/A	N/A	N/A	N/A	N/A	N/A
Kidney	Hyperemia	++	++	+++	++	++	+++	+	++	+	++	+	++	+	++
	Lymphocyte infiltration	+	+	+	+	+	+	+	+	+	+	+	+	+	+
Liver	Centrolobular hyperemia	+	+	+	+	+	+	++	++	++	+	+	+	+	+
	Portal hyperemia	++	++	++	++	++	++	+	+	+	+	+	+	+	+
	Megalocytosis	+	+	+	+	+	+	+	+	+	+	+	+	+	+
	Intracytoplasmic vacuolar degeneration	+	+	+	+	+	+	+	N	N	+	+	++	+	++
	Obvious hepatocyte cords	N	N	N	N	N	N	N	N	N	N	N	N	N	N
	Perivascular and periportal lymphocyte focus	N	+	+	N	+	+	++	++	++	++	++	++	++	++
	Hepatocyte Binucleation	+	+	+	+	+	+	+	+	+	+	+	+	+	+
Spleem	Red Pulp	N/A	N/A	N/A	N/A	N/A	N/A	N/A	N/A	N/A	N/A	N/A	N/A	N/A	N/A
	White Pulp	N/A	N/A	N/A	N/A	N/A	N/A	N/A	N/A	N/A	N/A	N/A	N/A	N/A	N/A

(A)

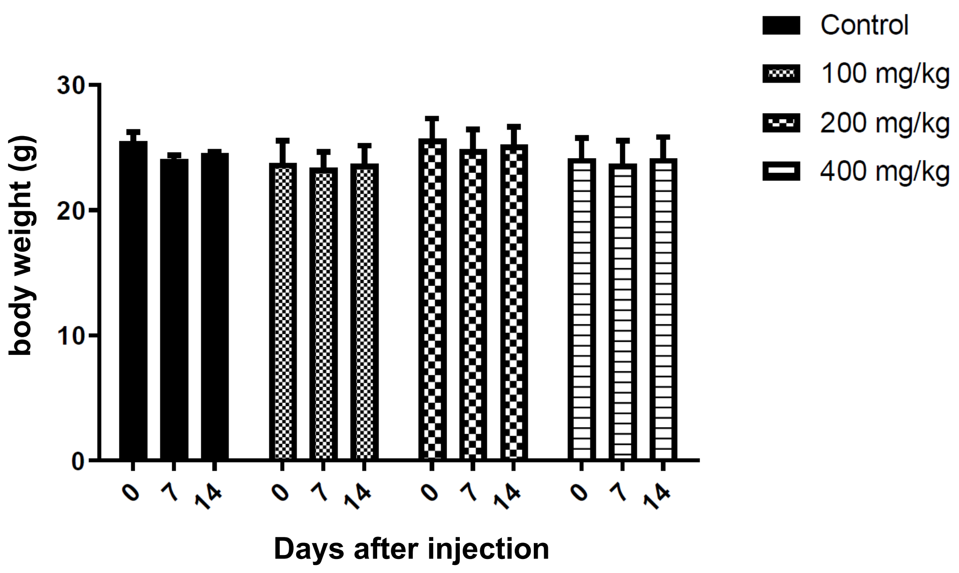

(B)

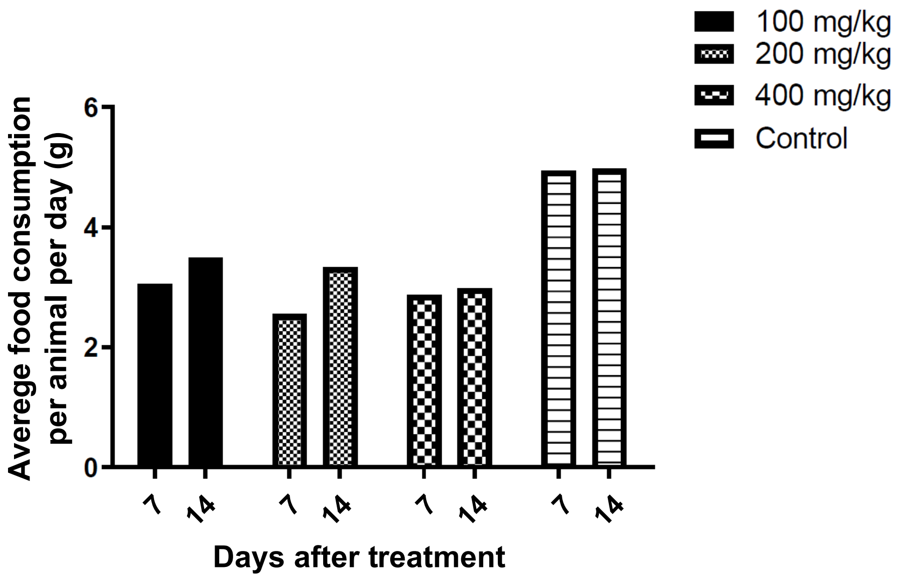

Figure S1. Acute toxicity study shows the mean body weight variation (Figure S1A) and food consumption (Figure S1B). Three groups of 3 or 4 mice were treated intraperitoneally with a dose (100 mg/Kg, 200 mg/Kg; 400 mg/Kg) of substance **4e** and followed for 14 days to analyze morbidity and mortality; euthanasia, necropsy, and histology were performed. A: Average food consumption during treatment, the error bar corresponds to the standard deviation. B: Mean body weight of each group during treatment, the error bar corresponds to the standard deviation.

Prediction of anticancer targets of 4e by reverse docking

Reverse docking is a valuable tool for screening a target database and predict potential targets for a compound, which, in turn, could help in the design of more potent and safer compounds [1,2]. Herein, we prepared a protein pool containing six proved targets of lapachol and other naphthoquinones which are also related to anticancer activity, and we performed a reverse screening to predict the potential target of **4e**. Of note, both enantiomers of **4e** were evaluated in this study, but the results presented here refer to the S enantiomer of this compound as this one was suggested to contribute mostly to its biological activity according to our docking studies.

Although **4e** shares a similar polycyclic moiety like etoposide and topotecan, this compound was not able to intercalate between the DNA nucleobases within topoisomerases complexes as the known inhibitors (data not shown). The dichlorophenyl group directly tethered to the polycyclic ring likely causes steric clashes with the surrounding nucleobases and prevents the intercalation into the DNA. The introduction of linking groups between these moieties might allow the intercalation and improvement of the compound's cytotoxicity.

On the other hand, **4e** bound within the ATP binding site of the ATPase domain of topoisomerase IIα, similarly to the co-crystallized inhibitor AMP-PNP, suggesting this enzyme is a potential target (Figure 1). The polycyclic nucleus of **4e** bound in the same region as the ribose group of AMP-PNP and explored residues tested by the phosphorous-containing groups of this inhibitor. This nucleus was anchored by two hydrogen bonds with N150 and was also involved in van der Waals contacts with N91, I141, S149, T159, and A167. Interestingly, 1,4-NQ is a naphthoquinone derivative that inhibits the ATPase activity of this enzyme [3], and it showed a similar interaction network comparing to compound **4e**. Besides, the dichlorophenyl ring of this compound was superimposed with the adenine ring of AMP-PNP, which allowed the establishment of related contacts with N120, I125, and T215 as well as a cation-π interaction with R98.

More recently, Zu and coworkers [4] demonstrated that the inhibition of RSK2 triggers the cytotoxicity of lapachol in squamous cell carcinoma. Interestingly, we observed that compound **4e** shows a similar binding manner with this enzyme, as observed for lapachol and the co-crystallized inhibitor 2NS (Figure 1). The polycyclic nucleus of **4e** was bound at the gatekeeper area and was hydrogen-bonded with L150. This interaction was observed for lapachol and 2NS, and it seems to be critical for the

binding of several RSK2 inhibitors [5-7]. The naphthoquinone part of this nucleus was oriented towards under the P-loop and interacted with residues L74, V82 and L200. The dichlorophenyl ring was positioned towards outside the active site, interacting with L74 within the β-strand β1. Interestingly, lapachol and 2NS were also shown to conserve these interactions, suggesting this enzyme as a putative anticancer target of compound **4e**.

Furthermore, lapachol and shikonin are 1,4-naphthoquinone derivatives and were shown to block the glycolytic pathway in cancer cells by targeting the PKM2 enzyme [8,9]. Like lapachol and shikonin, compound **4e** explored the entrance of the ATP binding site within PKM2, where the adenine ring of ATP binds in the rabbit enzyme, but also explored the region where the ribose group binds (Figure 1). As a result, we observed that the naphthoquinone part of the polycyclic nucleus of **4e** was sandwiched between H78 and A366 by π-π stacking and van der Waals interactions, respectively. These residues were also in close contact with lapachol and shikonin. In addition, we observed that the polycyclic ring of **4e** was involved in van der Waals contacts with the residues T50, Y83, and S362. The dichlorophenyl ring bound towards outside the active site and interacted with P53 and K367 via van der Waals interactions. Overall, these compounds shared similar contacts with the human enzyme, which was also comparable to the interactions of ATP bound with the rabbit homologous protein [10].

Collectively, our findings suggest that compound **4e** could bind to the ATPase domain of topoisomerase IIα, RSK2, and PKM2 in a similar manner than the known inhibitors of these targets. The lowest binding energy was found for the complex of **4e** with RSK2, but values of binding energies were comparable with the known inhibitors of the three targets. Therefore, the anticancer activity of this compound may occur through its effects on these three enzymes.

Table S2. Molecular interactions of compound **4e** with the putative targets predicted by reverse docking studies and comparison to known inhibitors used as reference compounds.

Protein	Ligand	Molecular interactions				
		van der Waals	Hydrogen bond	π-π stacking	Cation-π	Anion-π
**ATPase domain of Topoisomerase IIα**	**4e**	N91, N120, I125, I141, S149, T159, A167, T215	N150	-	R98	-
	1,4-NQ	N91, I141, G161, G164, G166	N150, A167, K168	-	-	-
	AMP-PNP	N91, N95, I125, I141, F142, G161, K168, T215	N120, S148, S149, N150, R162, R163, G164, Y165, G166, A167, Q376	-	-	-
**RSK2**	**4e**	L74, V82, L200	L150	-	-	-
	Lapachol	L74, Q76, V82, A98, N198, L200, T210	L150	-	-	-
	**2NS**	L74, V82, A98, G153, N198, L200	L150, T210	-	-	D211
**PKM2**	**4e**	T50, P53, Y83, S362, A366, K367	-	H78	-	-
	Lapachol	P53, H78, G79, H84, A366	N75	Y83	-	-
	Shikonin	P53, G62, G79, A366	N75, H84	Y83	-	-

Table S3. Residues comprising the ATP binding site of the predicted targets of compound **4e**. Binding site residues were defined as the residues within a 5 Å distance around co-crystallized ligands.

Protein	Residues interacting with co-crystallized ligands within 5 Å			
	Polar	Nonpolar	Basic	Acidic
**ATPase domain of Topoisomerase IIα (PDB code 1ZXM)**	N91, N95, N120, T147, S148, S149, N150, N163, Y165, T215, Q376	A92, G124, I125, I141, F142, G160, G161, G164, G166, A167	R98, K123, R162, K168, K378	E87, D94
**RSK2 (PDB code 4NW6)**	Q76, N198, T210	L74, G75, G77, V82, A98, V131, L147, F149, L150, G152, G153, L200	R151	D148, D154, E197, D211
**PKM2 (PDB code 3SDR)[a]**	T50, N75, S77, Y83, T328, S362	I51, G52, P53, G363, A366	R73, H78, H84, R120, K270, K367	D113, E118, E272, D296

[a] Residues were defined relative to the ATP bound to the rabbit PKM2 (PDB code 1A49) which shares an overall sequence identity of 93.21% with the human PKM2.

Cell cycle Analysis

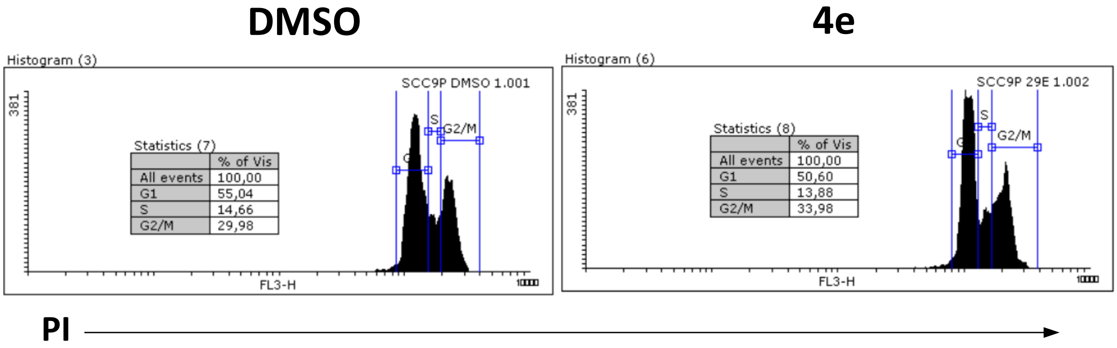

Figure S2. Differences in cell cycle distribution. Cell cycle distribution was analyzed after propidium iodide staining and FACS analyses. SCC9 lineage cells were plated in a 6-well plate (5 x 10^5 cells/well). The phases (sub G1), G0/G1, S and G2 of the cell cycle were classified based on the DNA content after staining with iodide of propidium (PI). Each image is representative of at least three independent experiments.

Some antitumor compounds can bind to DNA with the possibility of generating a change in the dynamics of the cell cycle; this arrest in some phases of the cycle has already been evidenced in some naphthoquinones. For example, naphthoquinone doxorubicin is capable of inducing a cell cycle arrest in the G0/G1 or G2 phases [11]. The naphthoquinone plumbagin, capable of inducing cell death by autophagy, acts in the cell cycle with an arrest in the G2/M phase [12]. In this way, the effect the antiproliferative effect of compound **4e** was also evaluated by analyzing the cell cycle in flow cytometry (Figure S2). The observed results show that there was no significant difference in the number of cells in the G1, S and G2/M phases after 48 hours of treatment, the same was observed for the DMSO control. This indicates that the treatment with compound **4e** does not interfere with the cell cycle.

References

1. Lee, A.; Kim, D. CRDS: Consensus Reverse Docking System for target fishing. *Bioinformatics* **2020**, *36*, 959-960.

2. Lee, A.; Lee, K.; Kim, D. Using reverse docking for target identification and its applications for drug discovery. *Exp. Opin. Drug Discov.* **2016**, *11*, 707-715.

3. Gurbani, D.; Kukshal, V.; Laubenthal, J.; Kumar, A.; Pandey, A.; Tripathi, S.; Arora, A.; Jain, S.K.; Ramachandran, R.; Anderson, D.; Dhawan, A. Mechanism of inhibition of the ATpase domain of human topoisomerase IIα by 1,4-benzoquinone, 1,2-naphthoquinone, 1,4-naphthoquinone, and 9,10-phenanthroquinone. *Toxicol. Sci.* **2012**, *126*, 372-390.

4. Zu, X.; Xie, X.; Zhang, Y.; Liu, K.; Bode, A.M.; Dong, Z.; Kim, D.J. Lapachol is a novel ribosomal protein S6 kinase 2 inhibitor that suppresses growth and induces intrinsic apoptosis in esophageal squamous cell carcinoma cells. *Phytother. Res.* **2019**, *33*, 2337-2346.

5. Aronchik, I.; Appleton, B.A.; Basham, S.E.; Crawford, K.; del Rosario, M.; Doyle, L. V.; Estacio, W.F.; Lan, J.; Lindvall, M.K.; Luu, C.A.; Ornelas, E.; Venetsanakos, E.; Shafer, C.M.; Jefferson, A.B. Novel potent and selective inhibitors of p90 ribosomal S6 kinase reveal the heterogeneity of RSK function in MAPK-driven cancers. *Mol. Cancer Res.* **2014**, *12*, 803-812.

6. Costales, A.; Mathur, M.; Ramurthy, S.; Lan, J.; Subramanian, S.; Jain, R.; Atallah, G.; Setti, L.; Lindvall, M.; Appleton, B.A.; Ornelas, E.; Feucht, P.; Warne, B.; Doyle, L.; Basham, S.E.; Aronchik, I.; Jefferson, A.B.; Shafer, C.M. 2-Amino-7-substituted benzoxazole analogs as potent RSK2 inhibitors. *Bioorg. Med. Chem. Lett.* **2014**, *24*, 1592-1596.

7. Jain, R.; Mathur, M.; Lan, J.; Costales, A.; Atallah, G.; Ramurthy, S.; Subramanian, S.; Setti, L.; Feucht, P.; Warne, B.; Doyle, L.; Basham, S.; Jefferson, A.B.; Lindvall, M.; Appleton, B.A.; Shafer, C.M. Discovery of Potent and Selective RSK Inhibitors as Biological Probes. *J. Med. Chem.* **2015**, *58*, 6766-6783.

8. Chen, J.; Xie, J.; Jiang, Z.; Wang, B.; Wang, Y.; Hu, X. Shikonin and its analogs inhibit cancer cell glycolysis by targeting tumor pyruvate kinase-M2. *Oncogene* **2011**, *30*, 4297-4306.

9. Babu, M.S.; Mahanta, S.; Lakhter, A.J.; Hato, T.; Paul, S.; Naidu, S.R. Lapachol inhibits glycolysis in cancer cells by targeting pyruvate kinase M2. *PLoS One* **2018**, *13*, e0191419.

10. Larsen, T.M.; Benning, M.M.; Rayment, I.; Reed, G.H. Structure of the Bis(Mg2+)-ATP-oxalate complex of the rabbit muscle pyruvate kinase at 2.1 Å resolution: ATP binding over a barrel. *Biochemistry* **1998**, *37*, 6247-6255.

11. Lüpertz, R.; Wätjen, W.; Kahl, R.; Chovolou, Y. Dose- and time-dependent effects of doxorubicin on cytotoxicity, cell cycle and apoptotic cell death in human colon cancer cells. *Toxicology* **2010**, *271*, 115-121.

12. Ma, X.; Yin, X.; Liu, H.; Chen, Q.; Feng, Y.; Ma, Xi.; Liu, W. Antiproliferative activity of plumbagin (5-hydroxy-2-methyl-1,4-naphthoquinone) in human gastric carcinoma cells is facilitated via activation of autophagic pathway, mitochondrialmediated programmed cell death and inhibition of cell migration and invasion. *Journal of BUON* **2019**, *24*, 2000-2005.
