# Supplementary material for: Pro-Apoptotic Antitumoral Effect of Novel Acridine-Core Naphthoquinone Compounds against Oral Squamous Cell Carcinoma
Source: Molecules. 2022 Aug 12;27(16):5148. doi: 10.3390/molecules27165148 (PMC9415509; doi:10.3390/molecules27165148)
Supplement: Supplementary file 1 [file molecules-27-05148-s001.zip › Supplementary Spectral data SI.pdf]

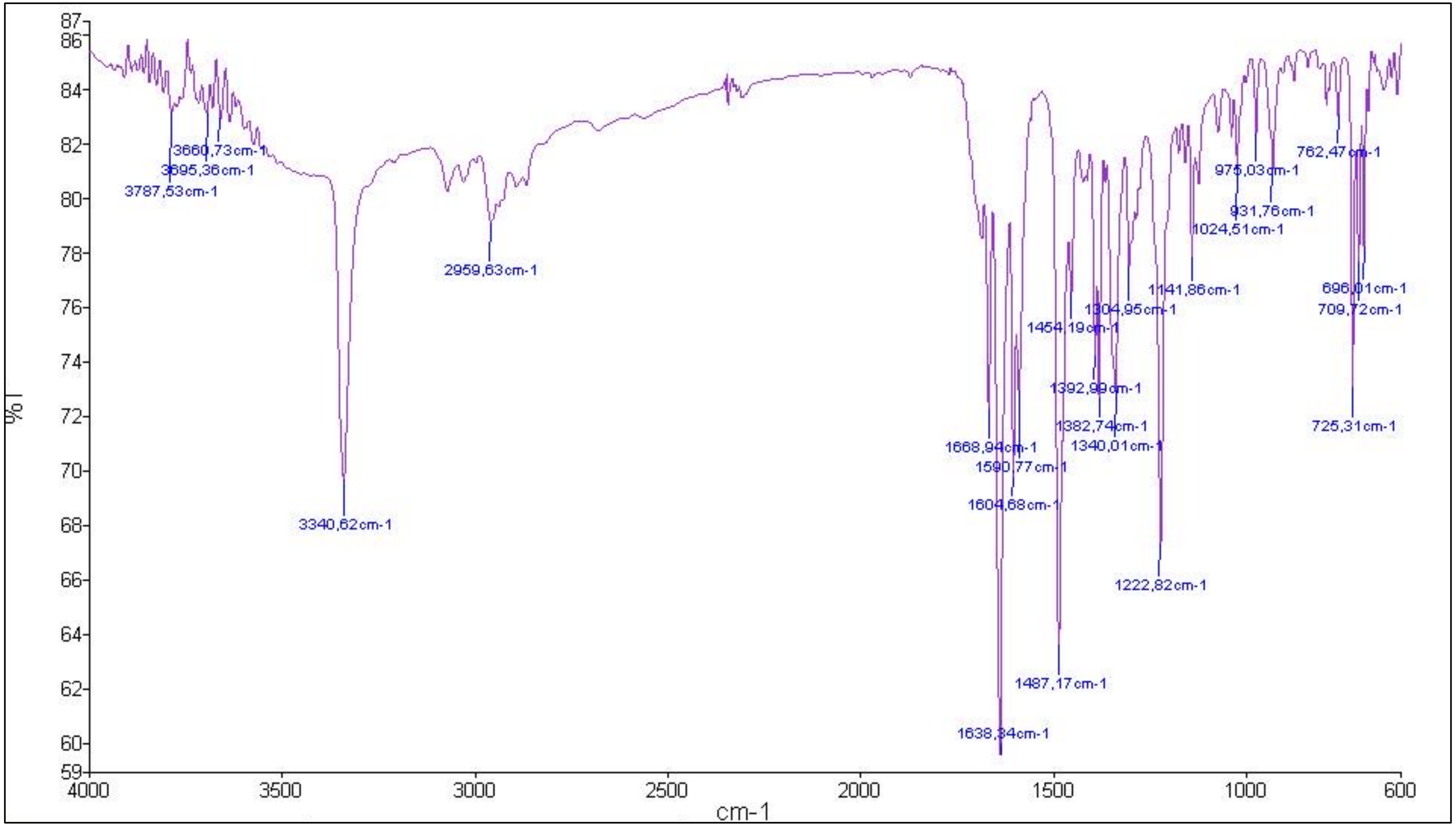

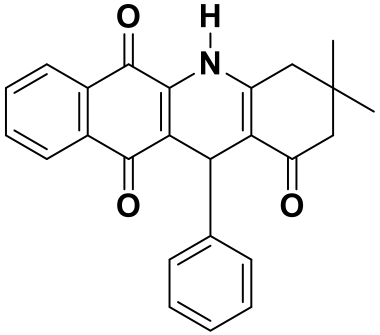

4a

IR spectrum of compound **4a**

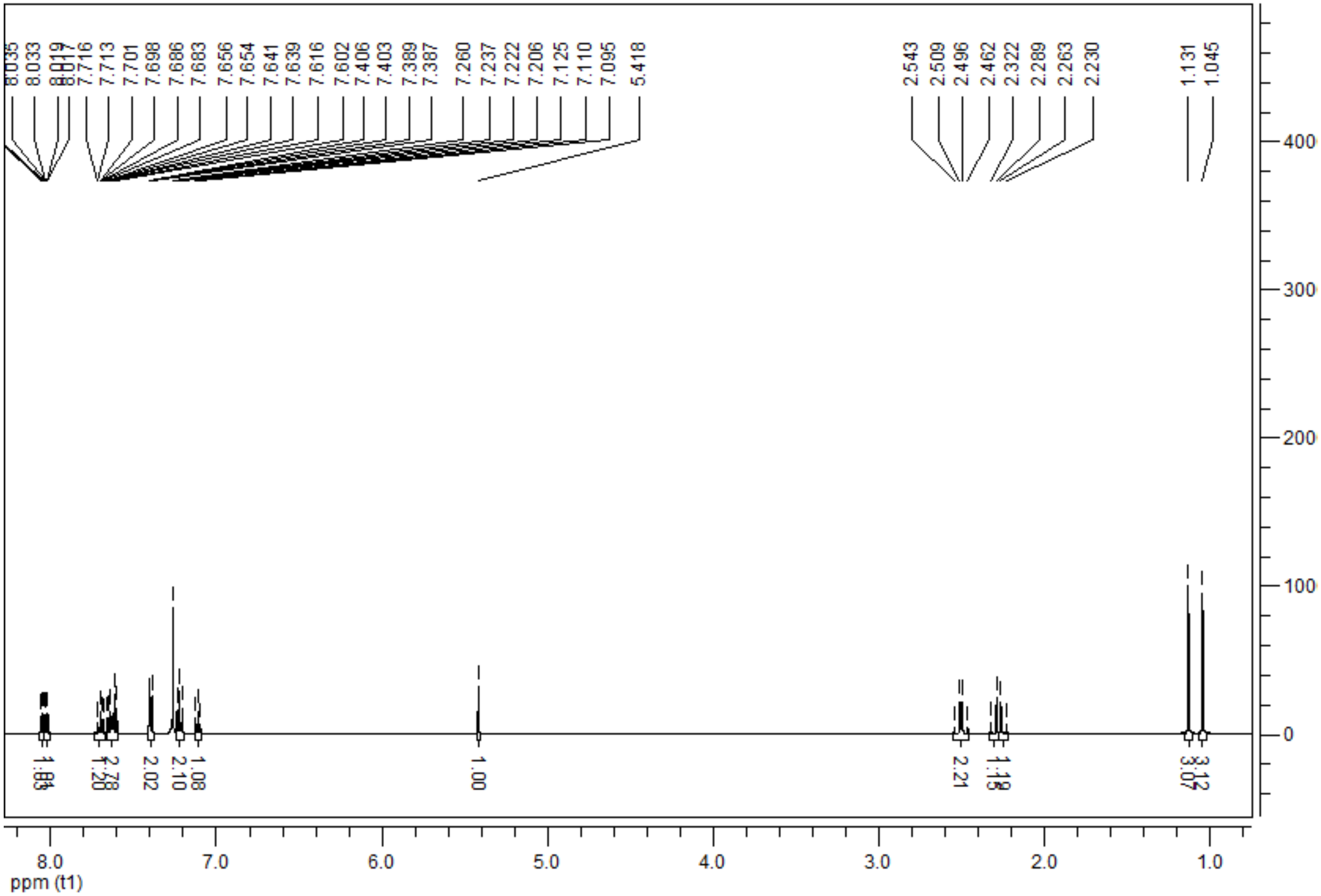

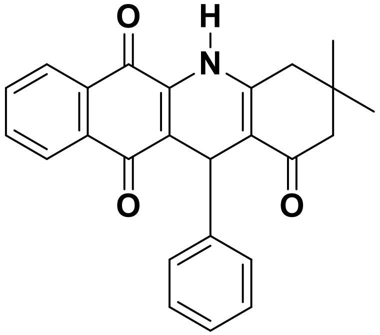

4a

^{1}H NMR spectrum of compound **4a** (500.00 MHz, $CDCl_3$)

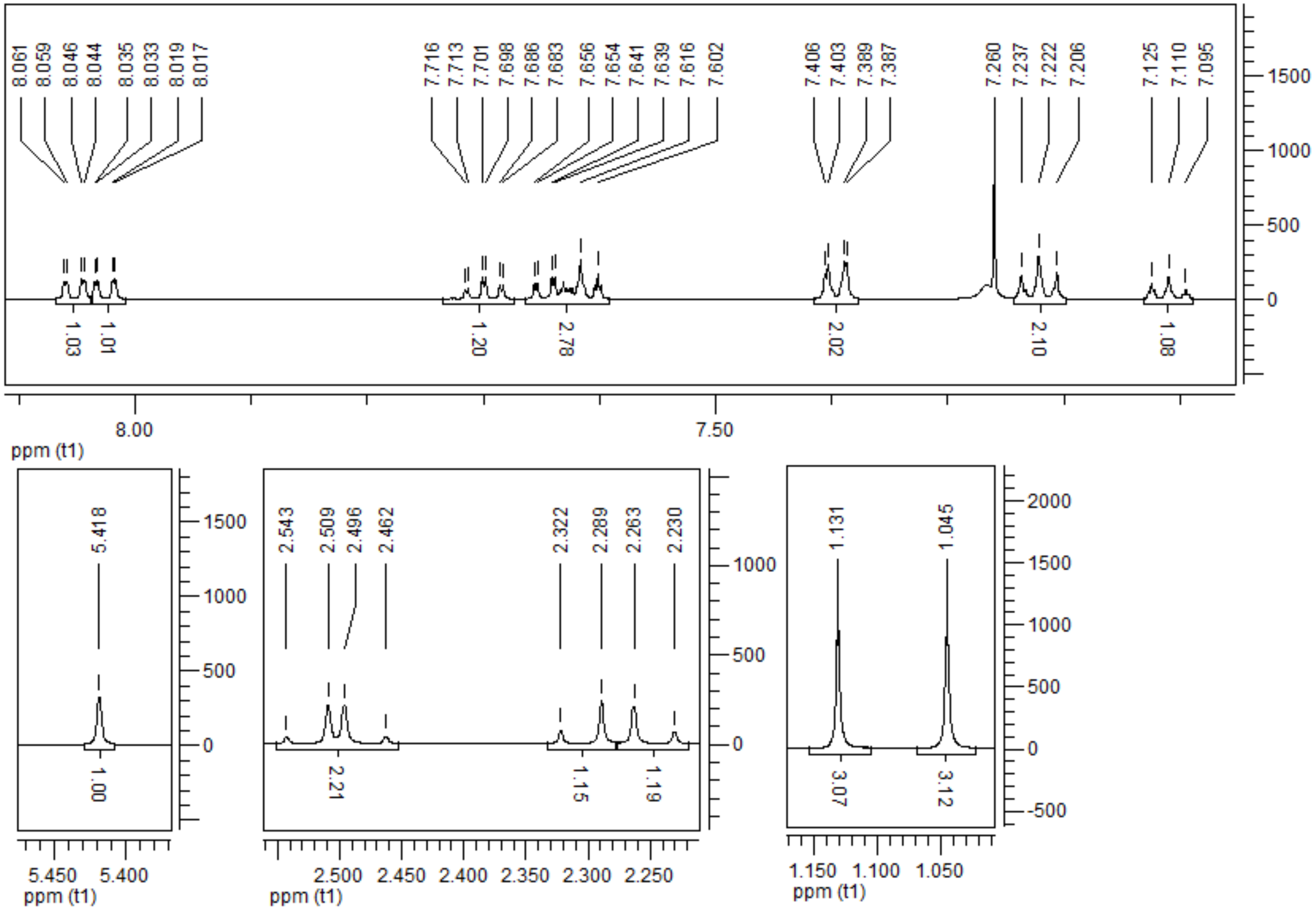

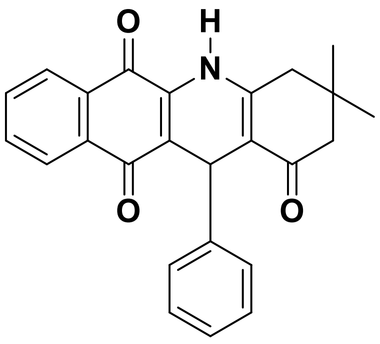

4a

^{1}H NMR spectrum of compound **4a** (500.00 MHz, $CDCl_3$)

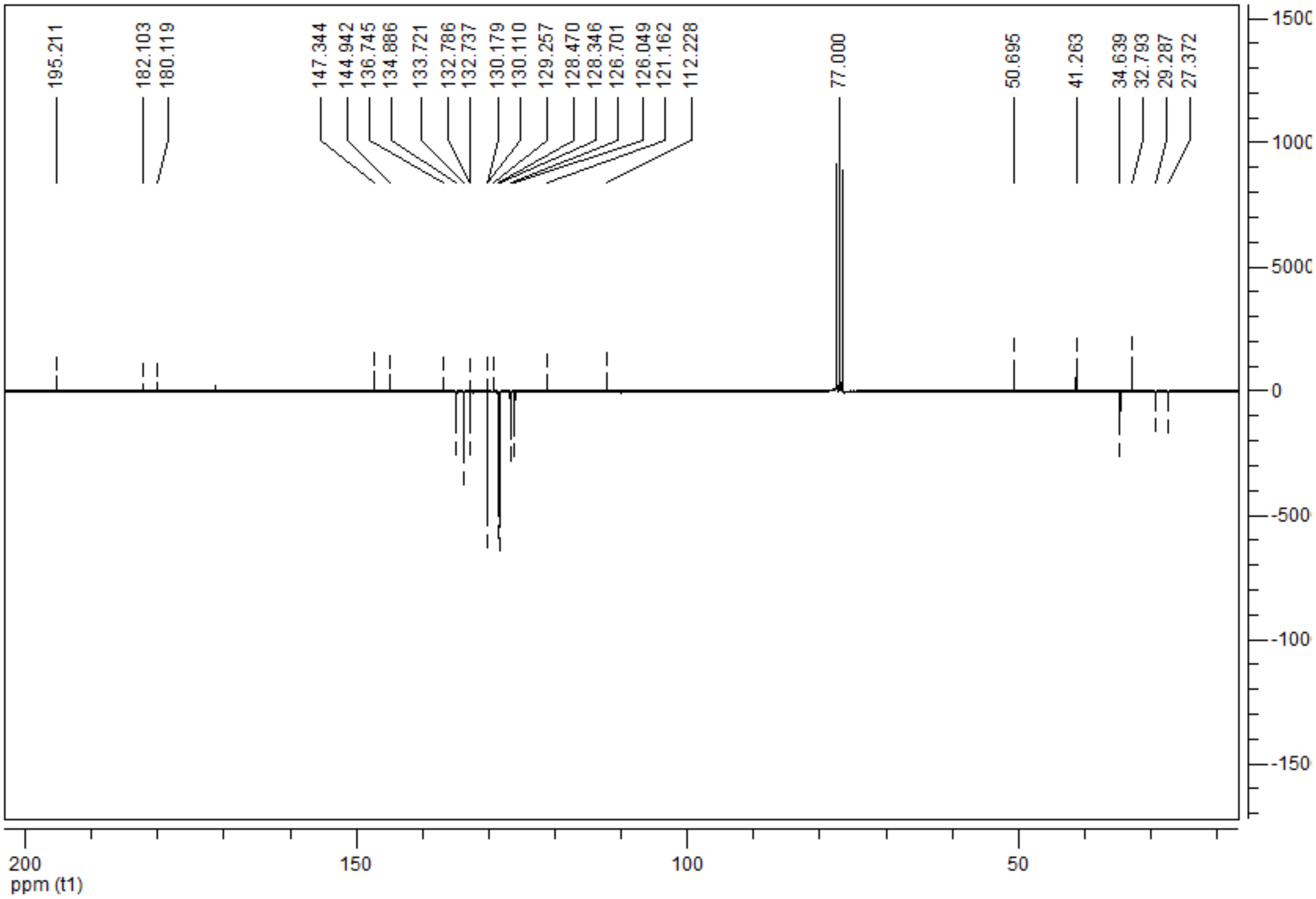

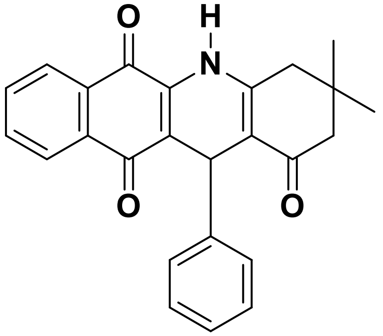

4a

^{13}C NMR spectrum of compound **4a** (75.0 MHz, $CDCl_3$)

+MS, 0.3-0.9min #15-50

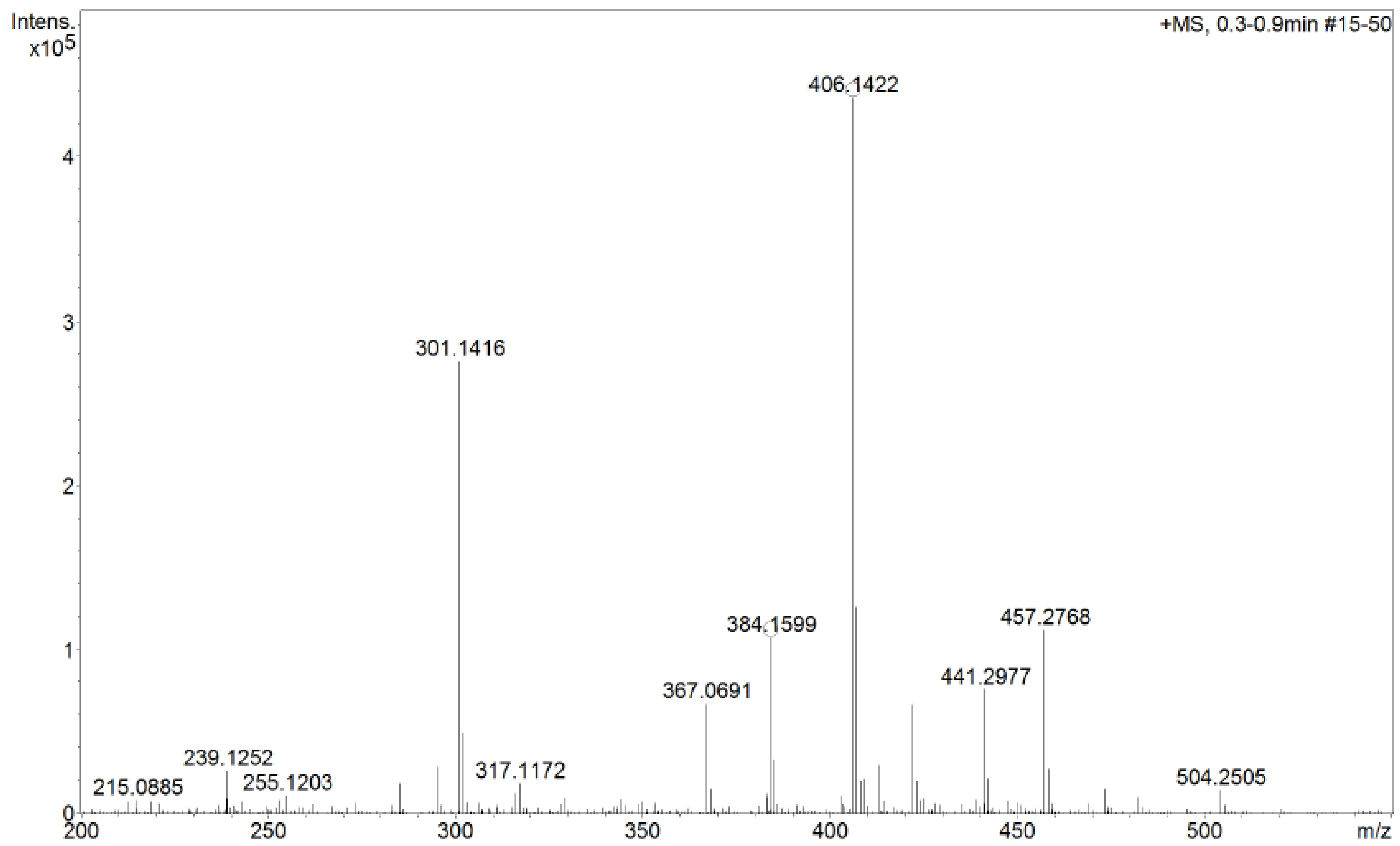

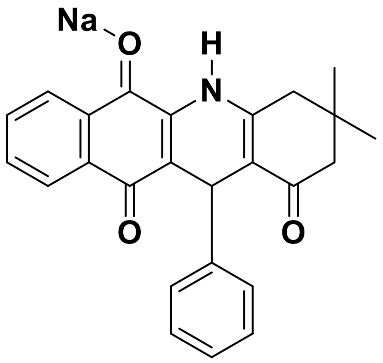

Exact Mass: 406,1419

4a

Meas. m/z	#	Ion Formula	m/z	err [ppm]	Mean err [ppm]	rdb	N-Rule	e⁻ Conf	mSigma	Std I	Std Mean m/z	Std I VarNorm	Std m/z Diff	Std Comb Dev
384.159934	1	C25H22NO3	384.159420	-1.3	0.9	15.5	ok	even	16.7	25.8	n.a.	n.a.	n.a.	n.a.
	2	C21H18N7O	384.156735	-8.3	-6.9	16.5	ok	even	30.9	49.5	n.a.	n.a.	n.a.	n.a.
406.142155	1	C25H21NNaO3	406.141364	-1.9	-1.0	15.5	ok	even	7.4	11.1	n.a.	n.a.	n.a.	n.a.
	2	C21H17N7NaO	406.138679	-8.6	-8.4	16.5	ok	even	21.5	34.6	n.a.	n.a.	n.a.	n.a.

HRMS of compound **4a**

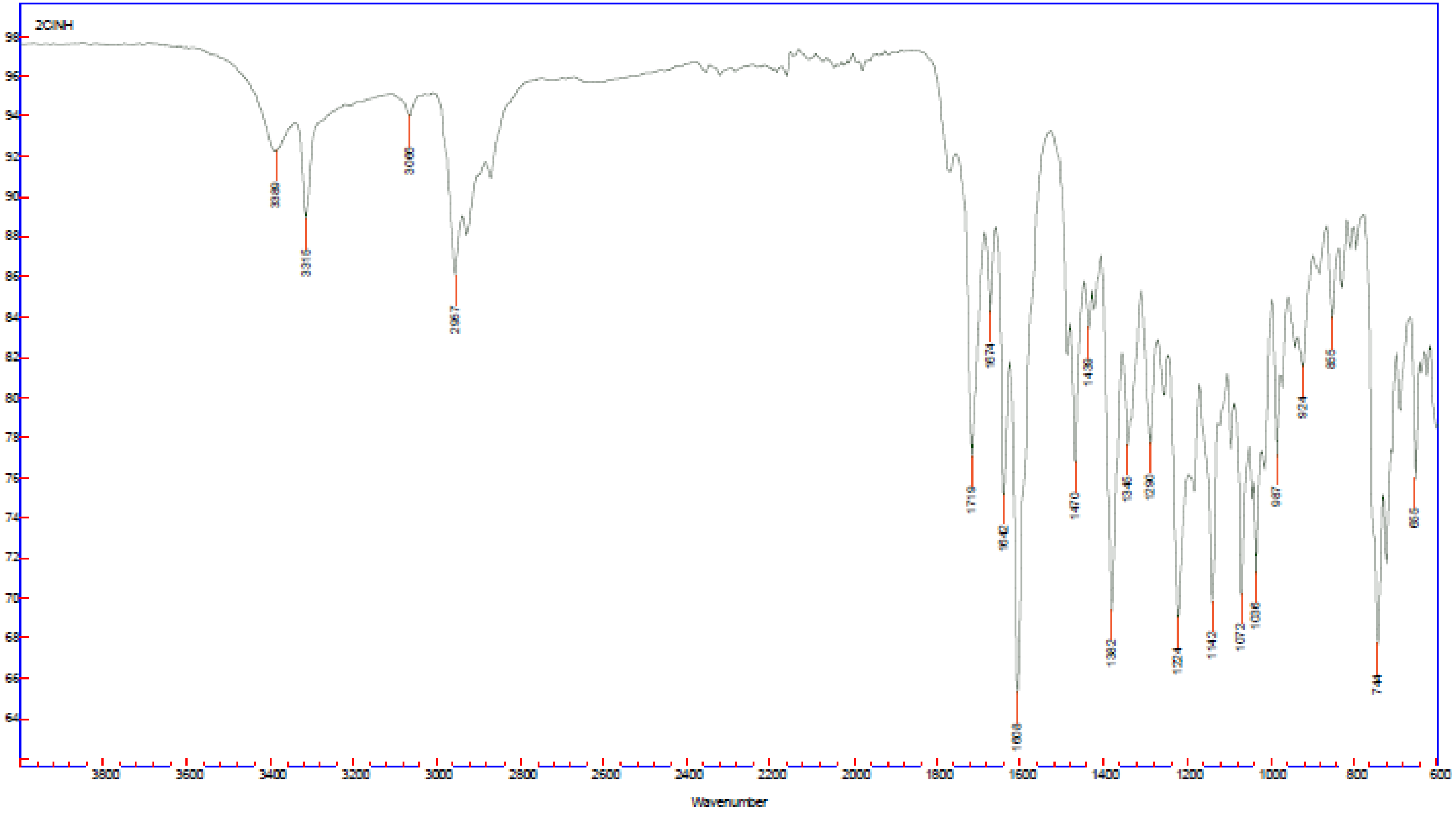

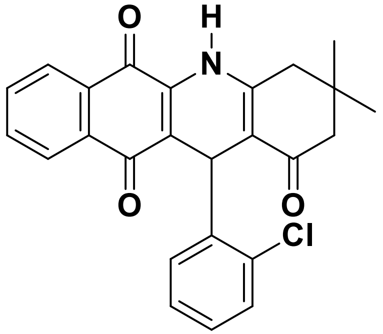

4b

IR spectrum of compound **4b**

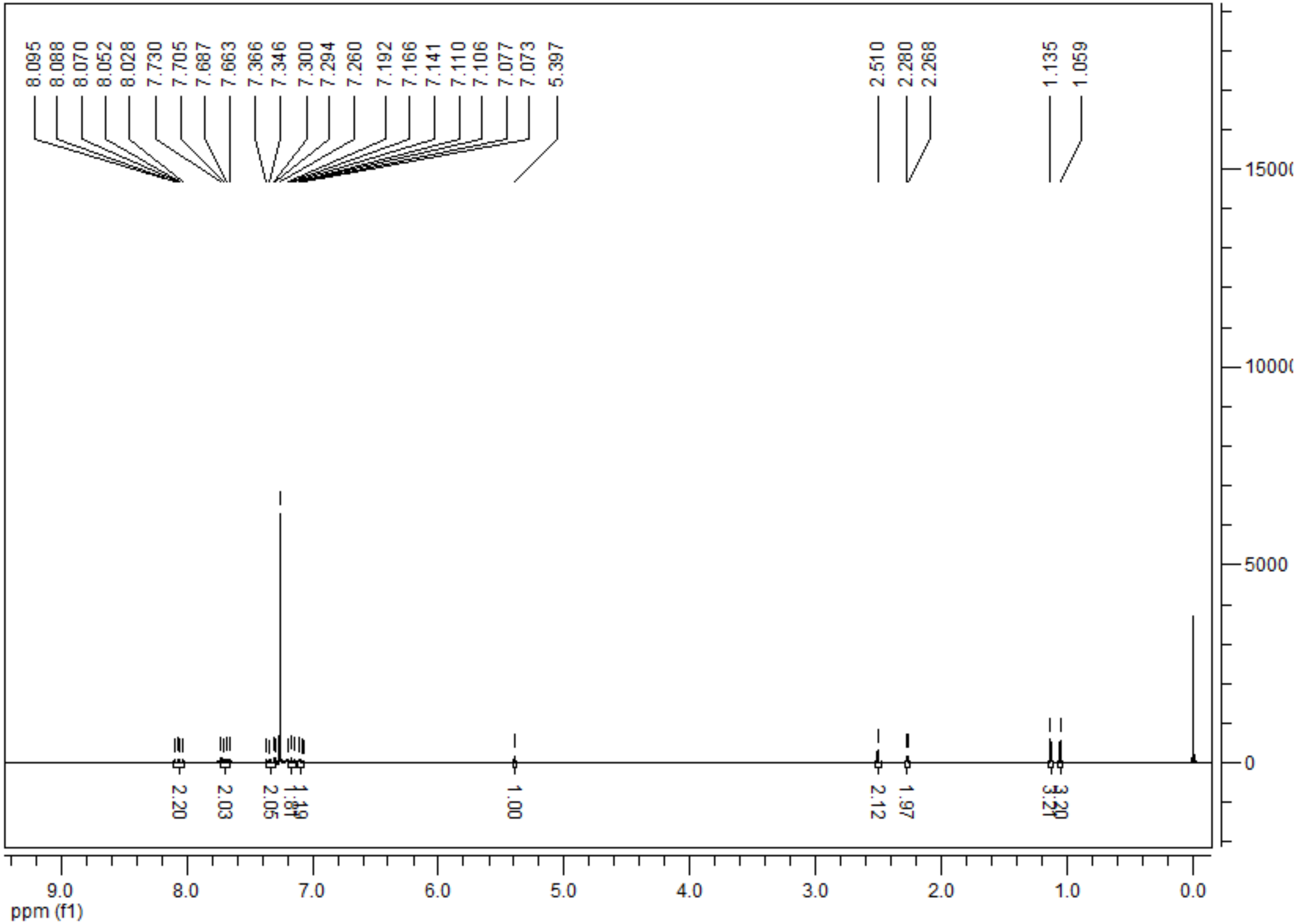

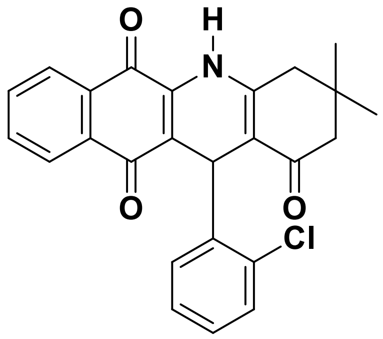

4b

^{1}H NMR spectrum of compound **4b** (300.00 MHz, $CDCl_3$)

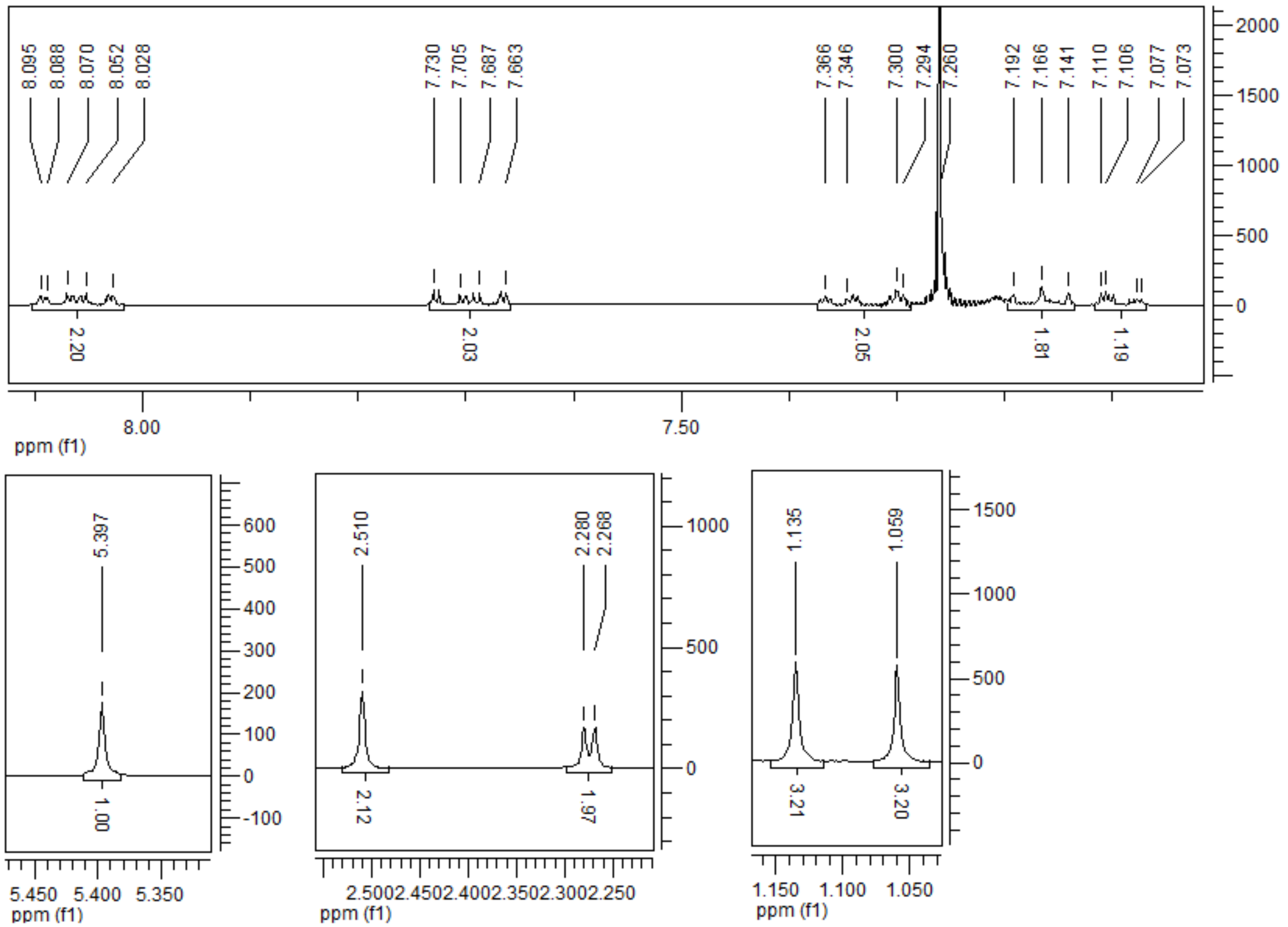

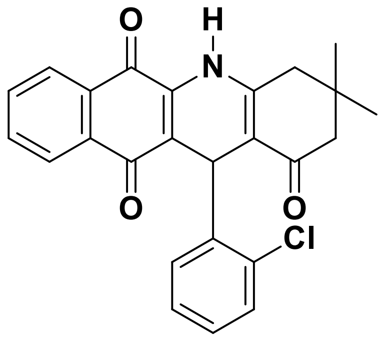

4b

^{1}H NMR spectrum of compound **4b** (300.00 MHz, $CDCl_3$)

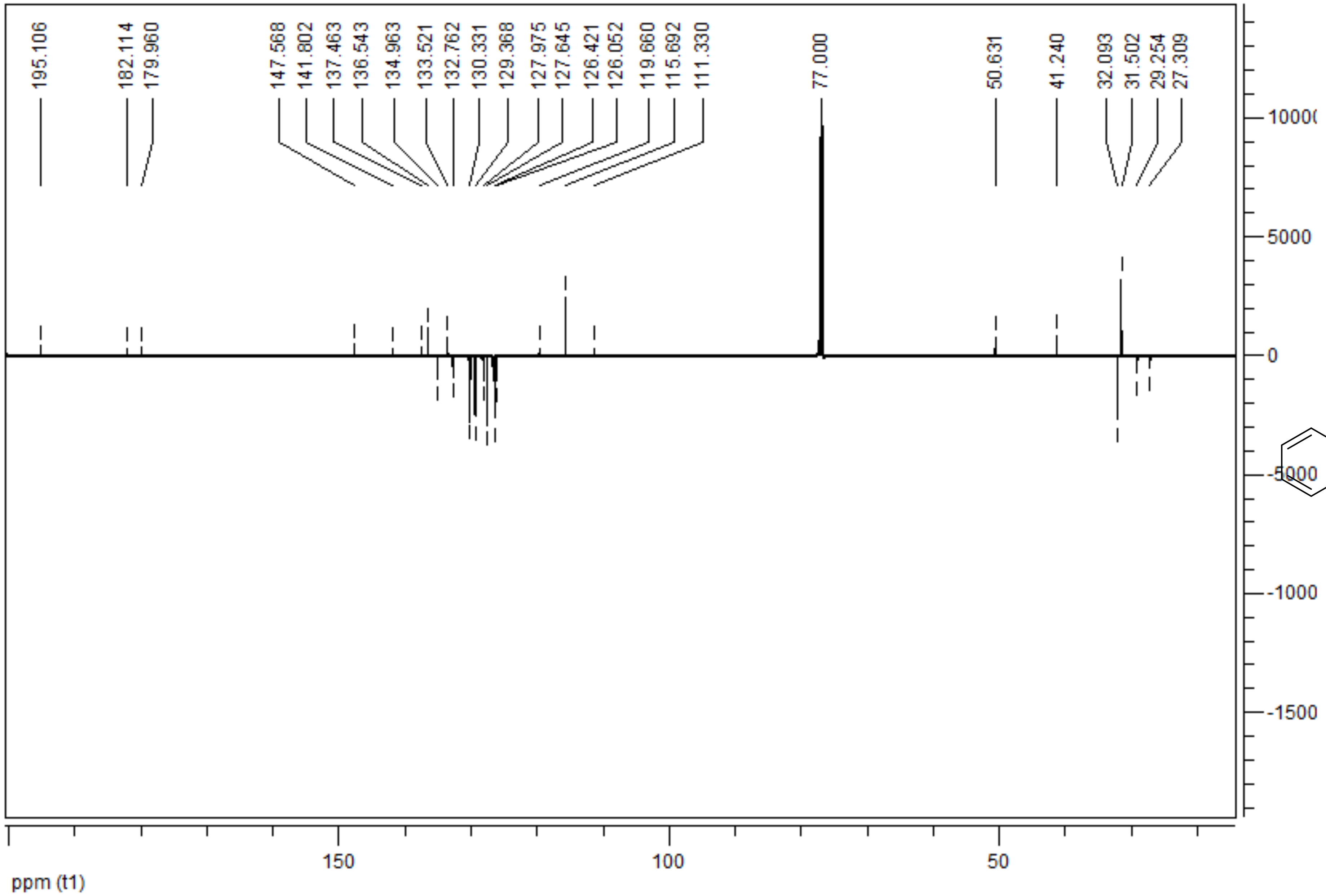

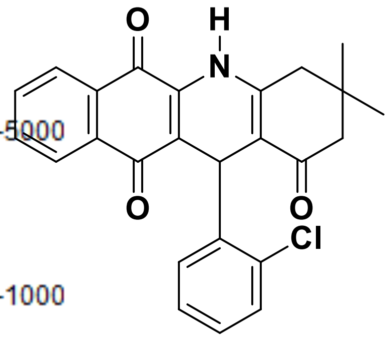

4b

^{13}C NMR spectrum of compound **4b** (125.0 MHz, $CDCl_3$)

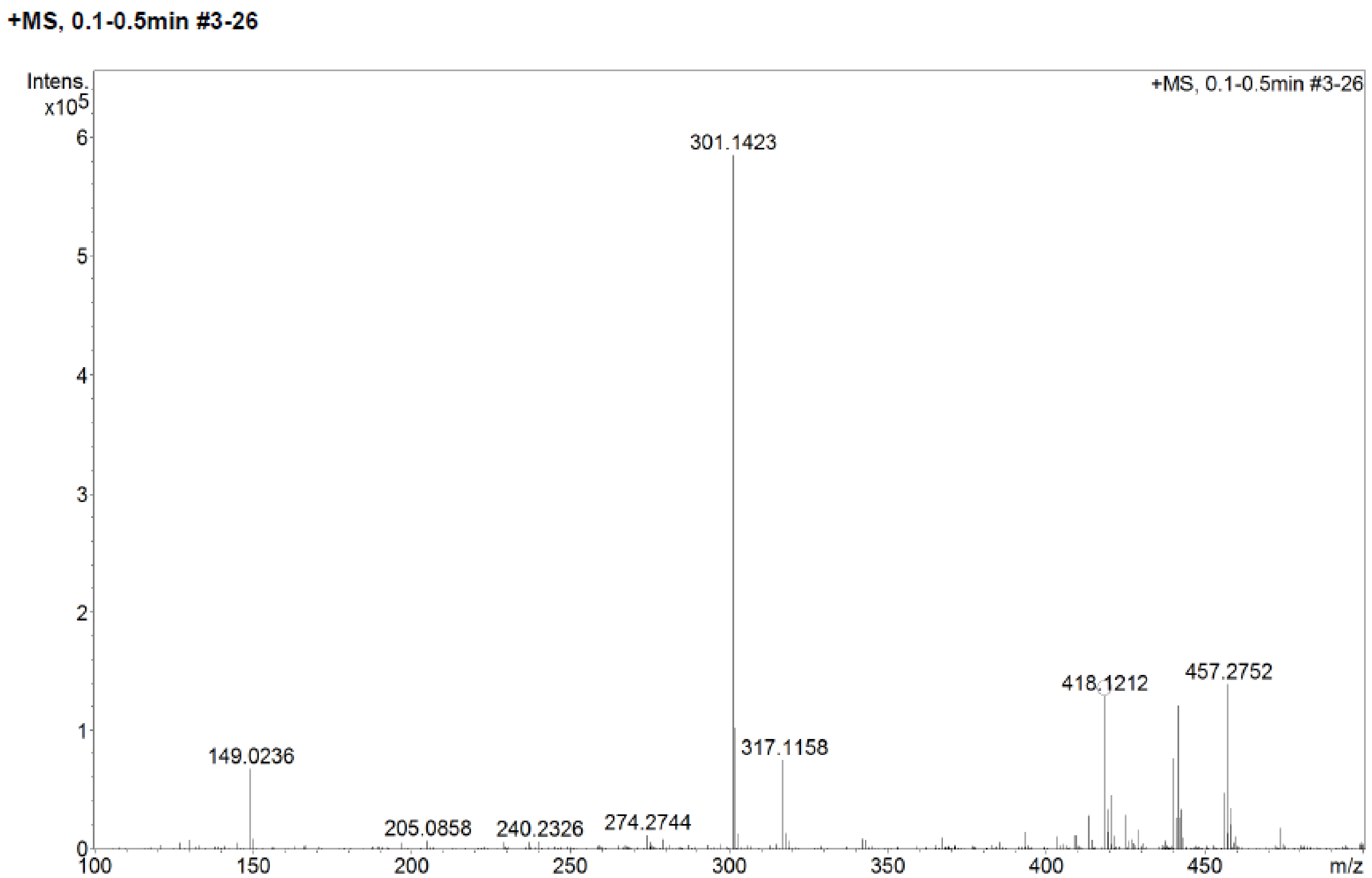

Meas. m/z	#	Ion Formula	m/z	err [ppm]	Mean err [ppm]	rdb	N-Rule	e⁻ Conf	mSigma	Std I	Std Mean m/z	Std I VarNorm	Std m/z Diff	Std Comb Dev
418.121246	1	C21H17ClN7O	418.117762	-8.3	-9.0	16.5	ok	even	3.6	5.7	n.a.	n.a.	n.a.	n.a.
	2	C25H21ClNO3	418.120448	-1.9	-1.6	15.5	ok	even	10.2	14.4	n.a.	n.a.	n.a.	n.a.

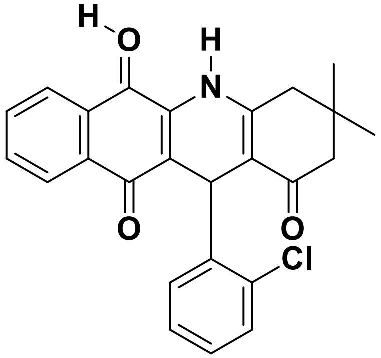

Exact Mass: 418,1210

4b

HRMS of compound **4b**

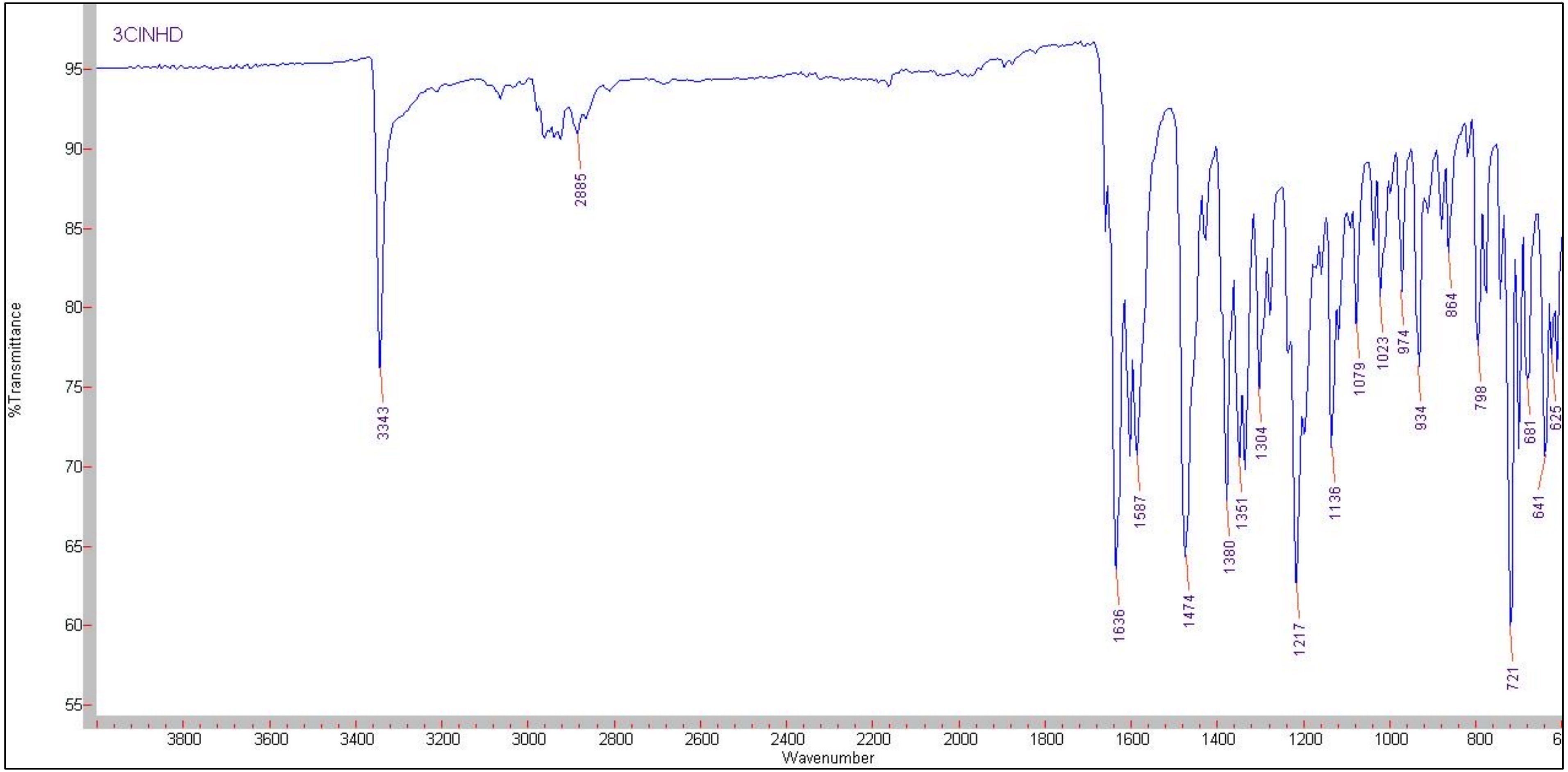

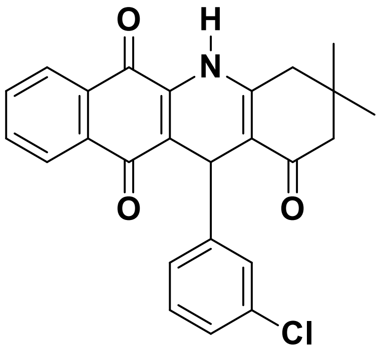

4c

IR spectrum of compound **4c**

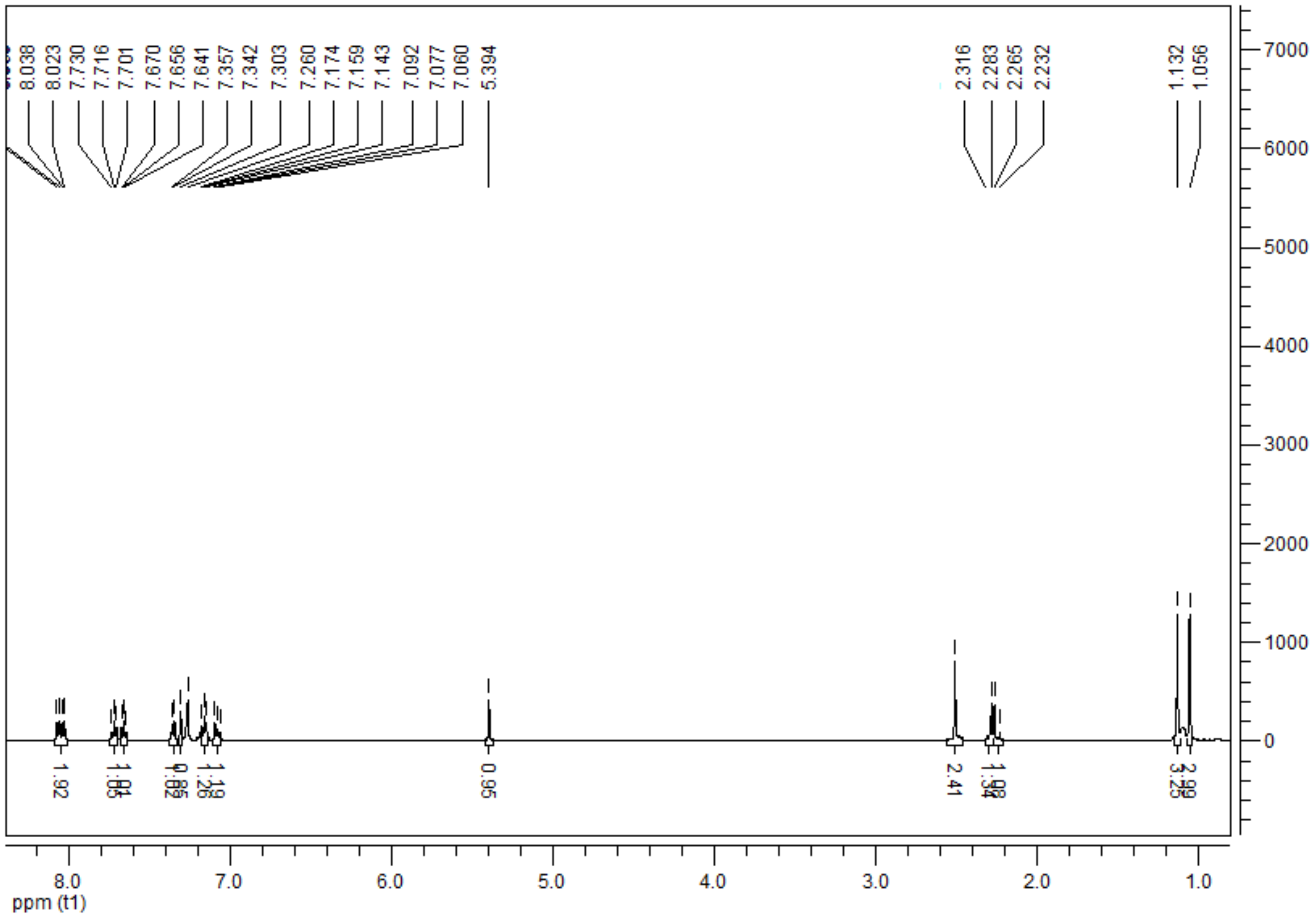

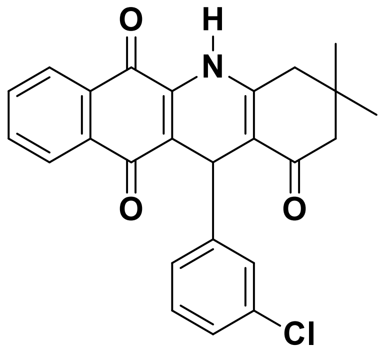

4c

^{1}H NMR spectrum of compound **4c** (500.00 MHz, $CDCl_3$)

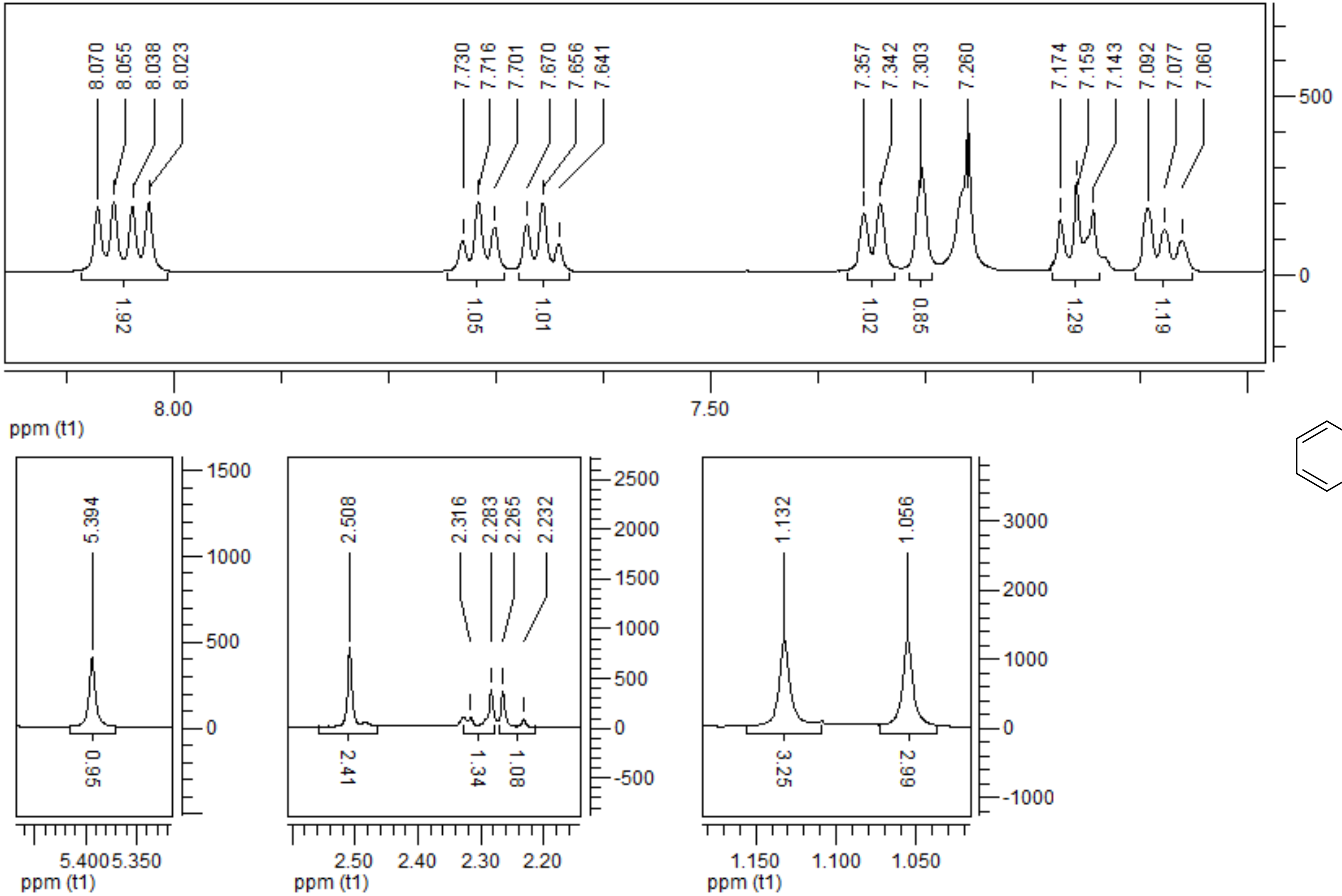

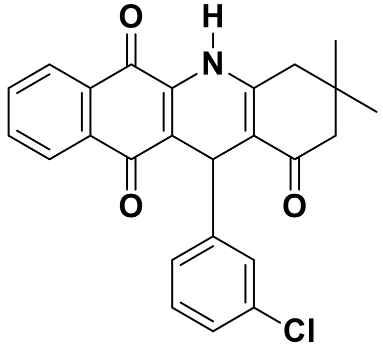

4c

^{1}H NMR spectrum of compound **4c** (500.00 MHz, $CDCl_3$)

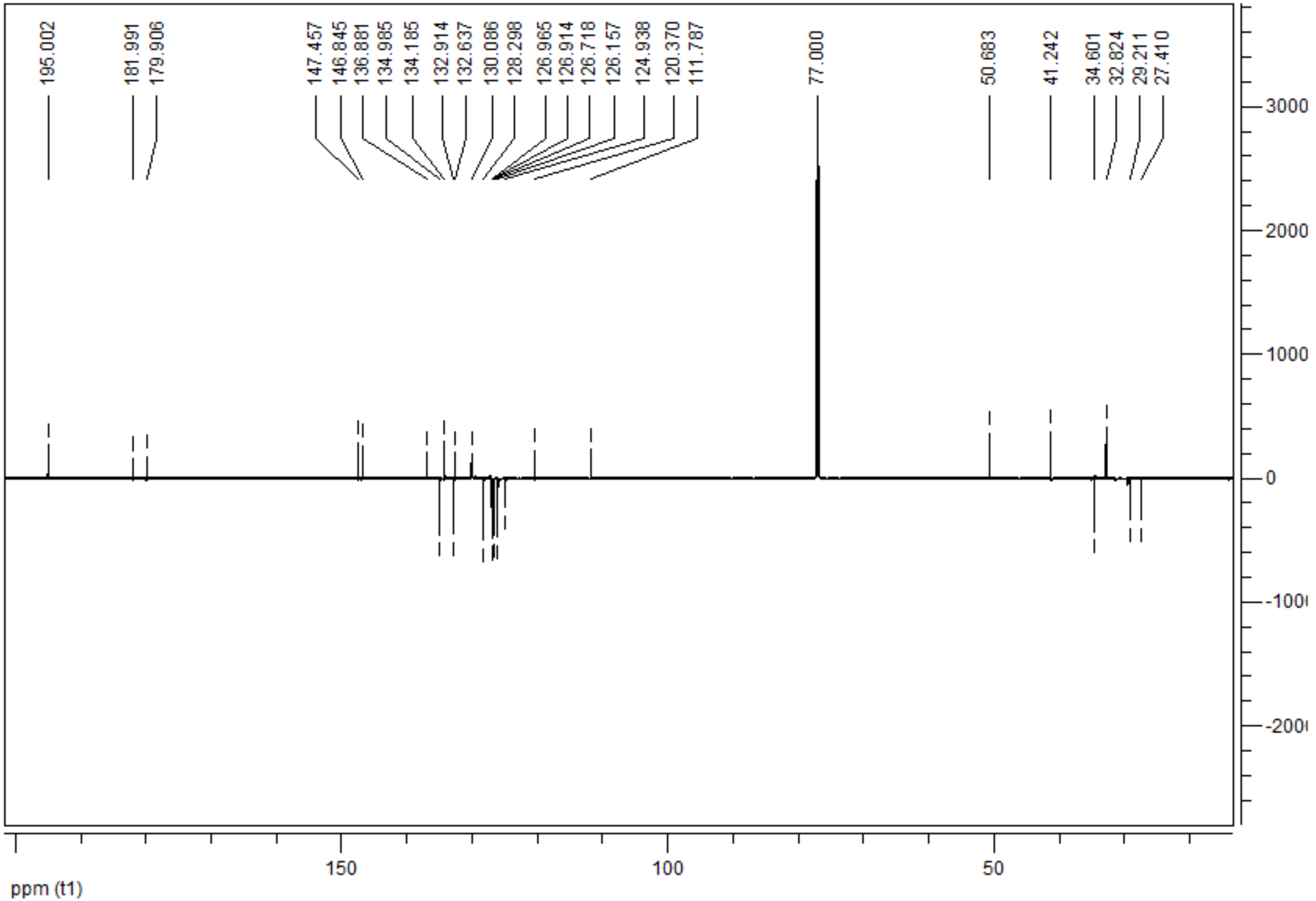

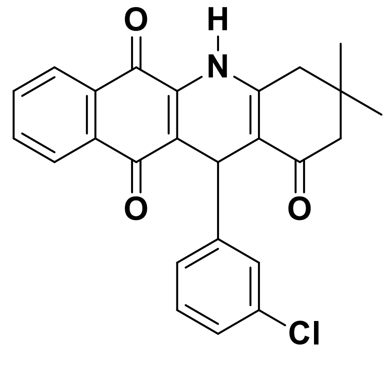

4c

^{13}C NMR spectrum of compound **4c** (75.0 MHz, $CDCl_3$)

+MS, 0.1-0.5min #3-27

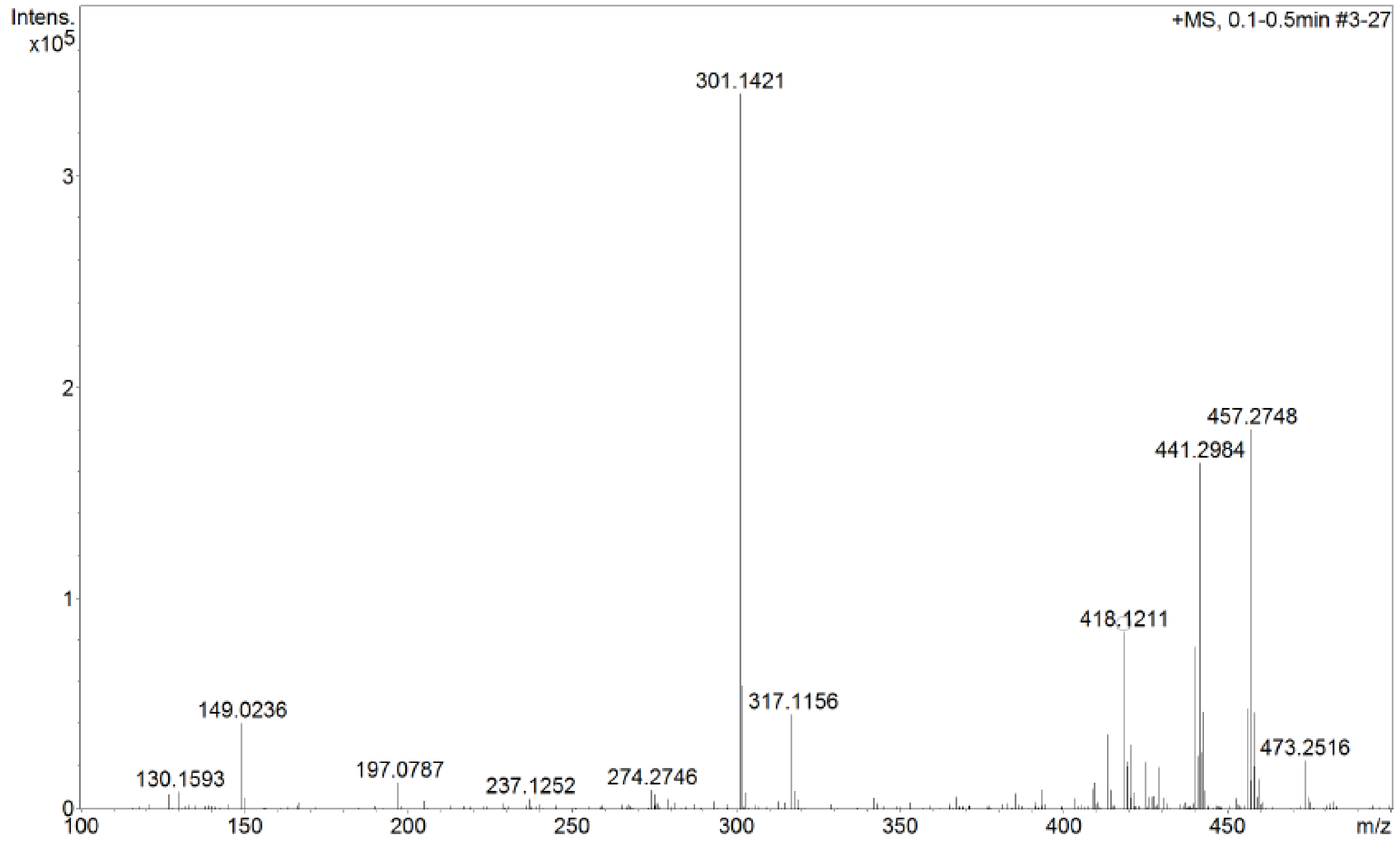

Meas. m/z	#	Ion Formula	m/z	err [ppm]	Mean err [ppm]	rdb	N-Rule	e⁻ Conf	mSigma	Std I	Std Mean m/z	Std I VarNorm	Std m/z Diff	Std Comb Dev
418.121080	1	$C_{25}H_{21}ClNO_3$	418.120448	-1.5	-0.8	15.5	ok	even	5.6	7.7	n.a.	n.a.	n.a.	n.a.
	2	$C_{21}H_{17}ClN_7O$	418.117762	-7.9	-8.2	16.5	ok	even	6.7	11.2	n.a.	n.a.	n.a.	n.a.

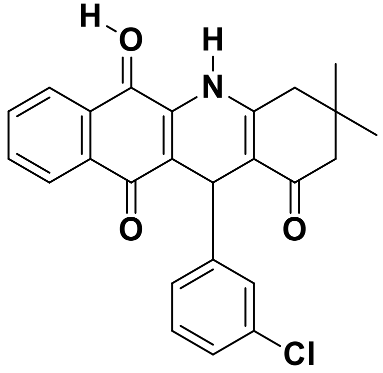

Exact Mass: 418,1210

4c

HRMS of compound **4c**

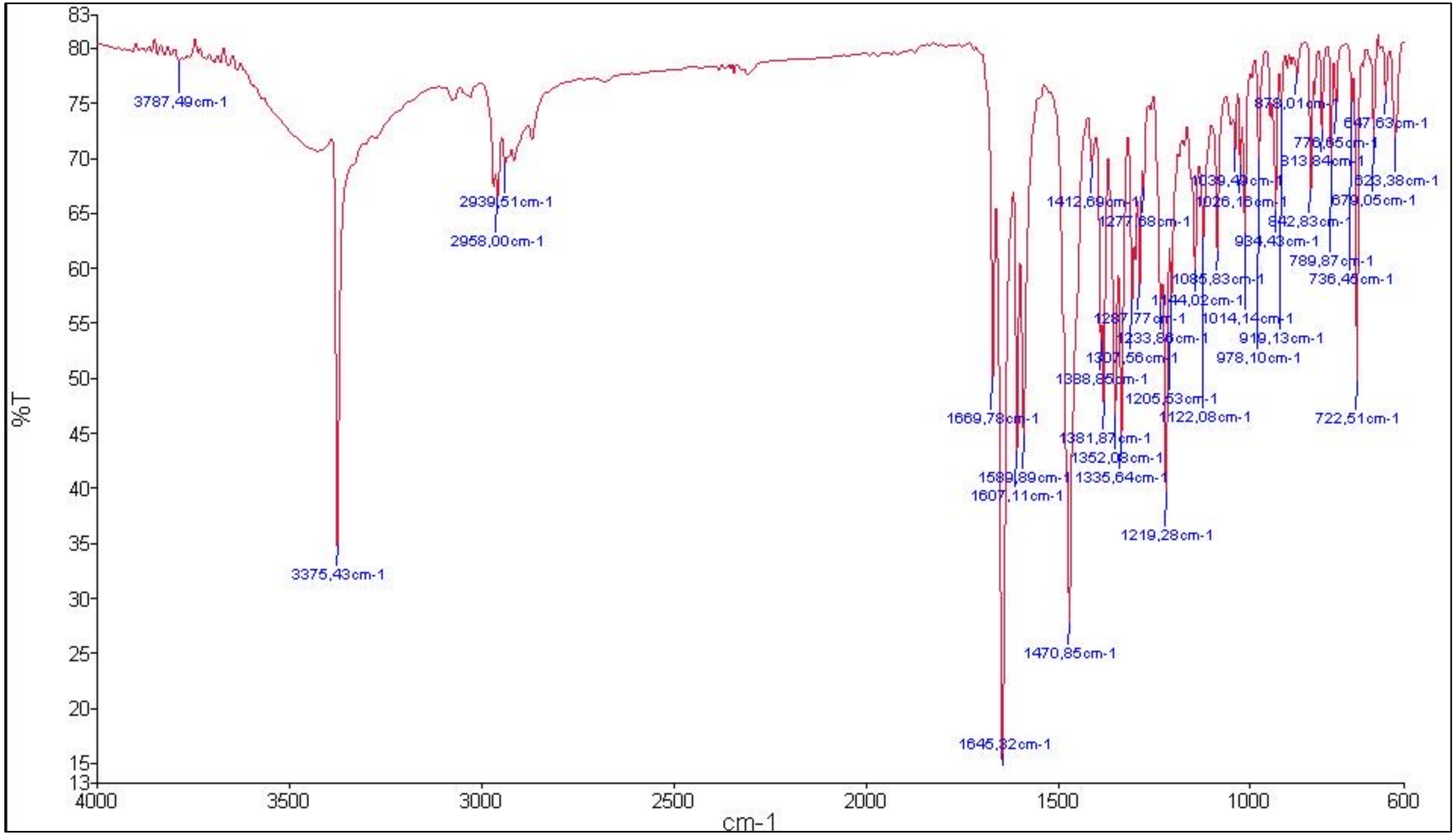

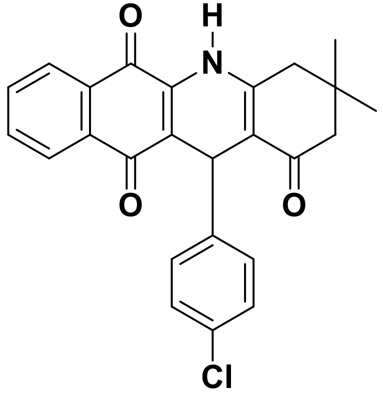

4d

IR spectrum of compound **4d**

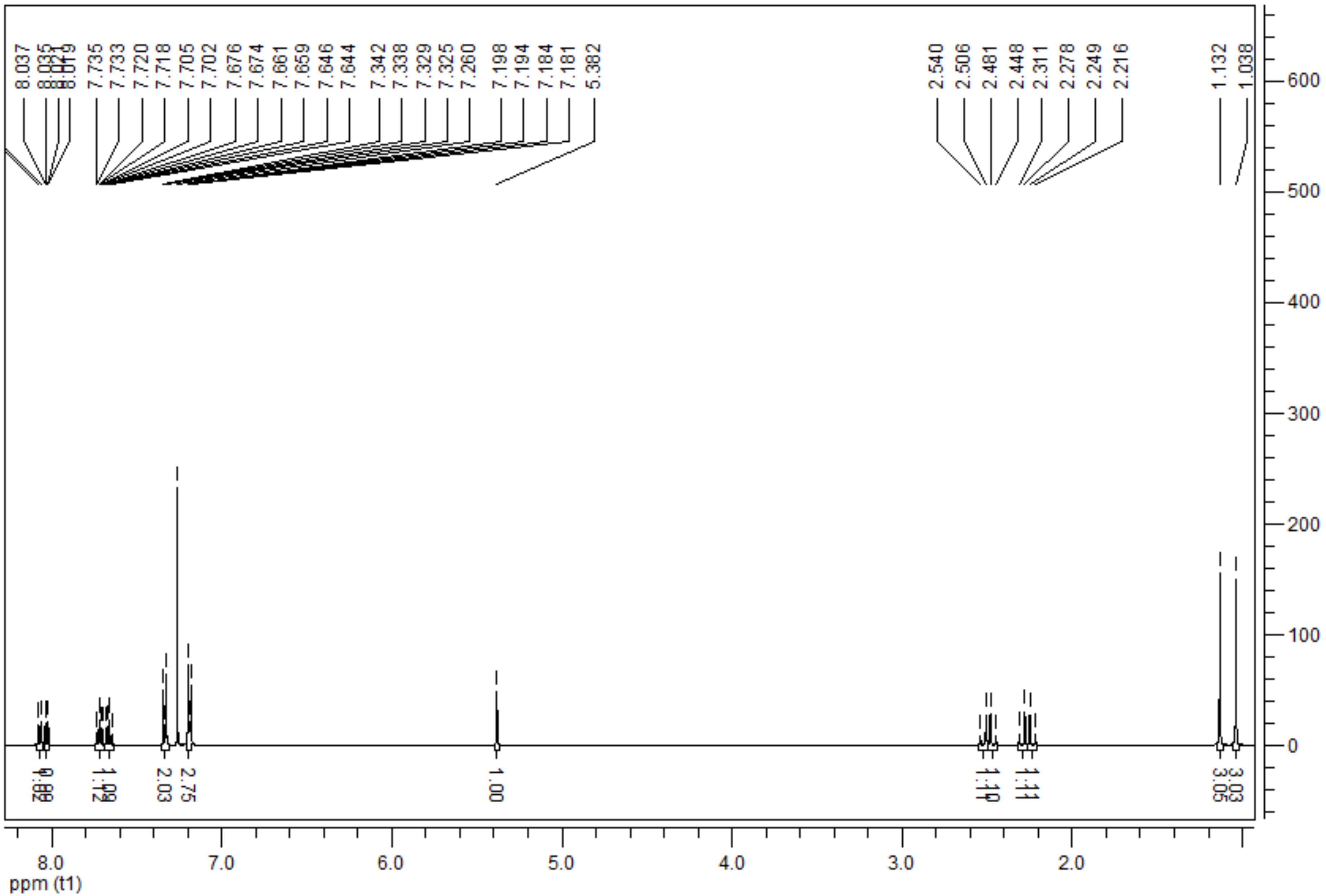

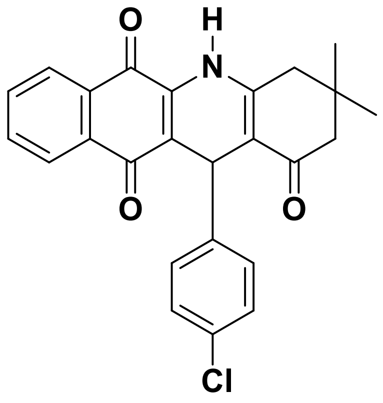

4d

^{1}H NMR spectrum of compound **4d** (500.00 MHz, $CDCl_3$)

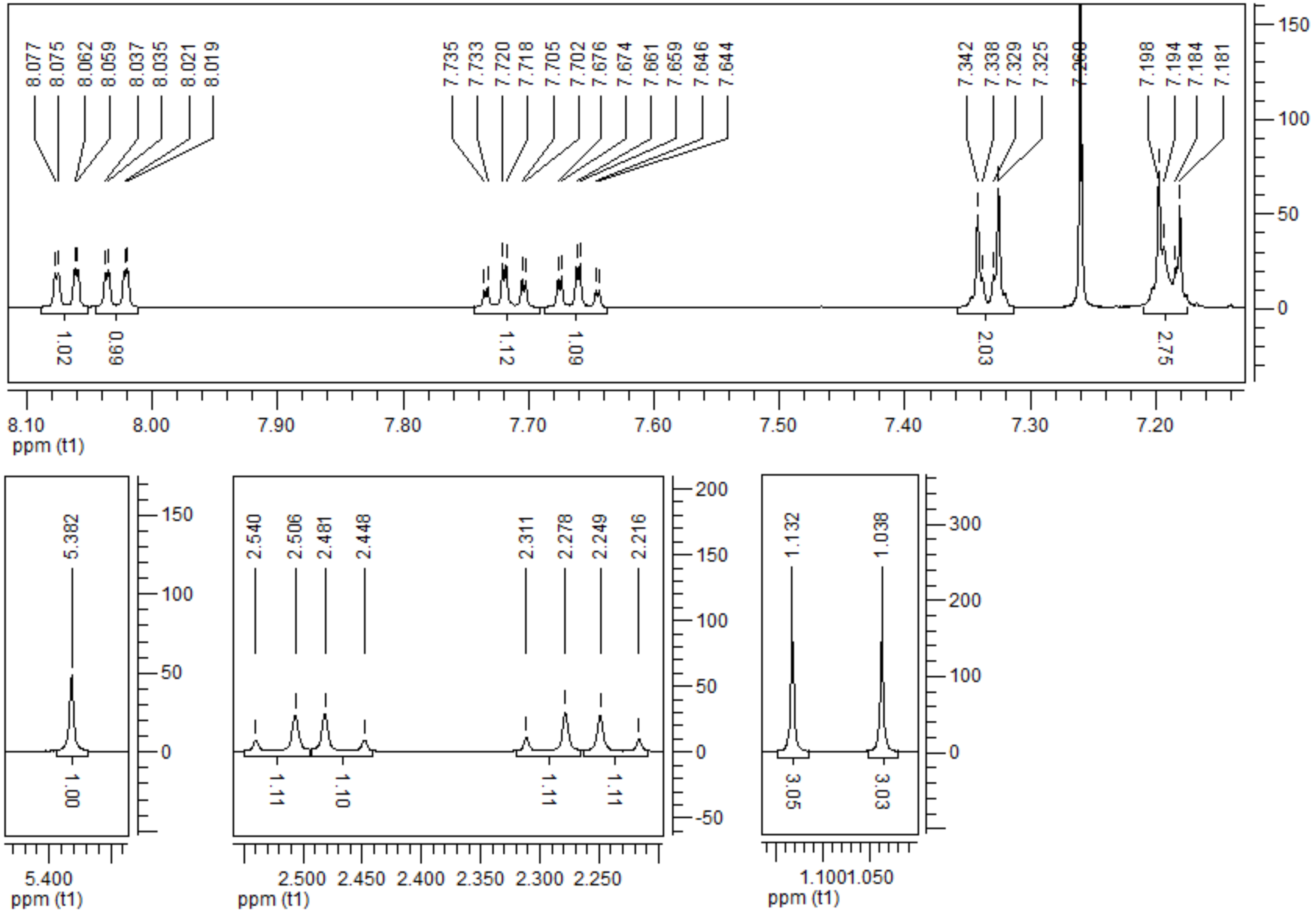

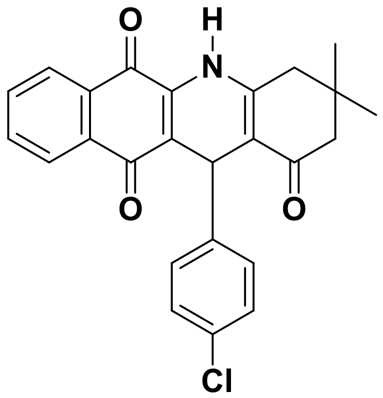

4d

^{1}H NMR spectrum of compound **4d** (500.00 MHz, $CDCl_3$)

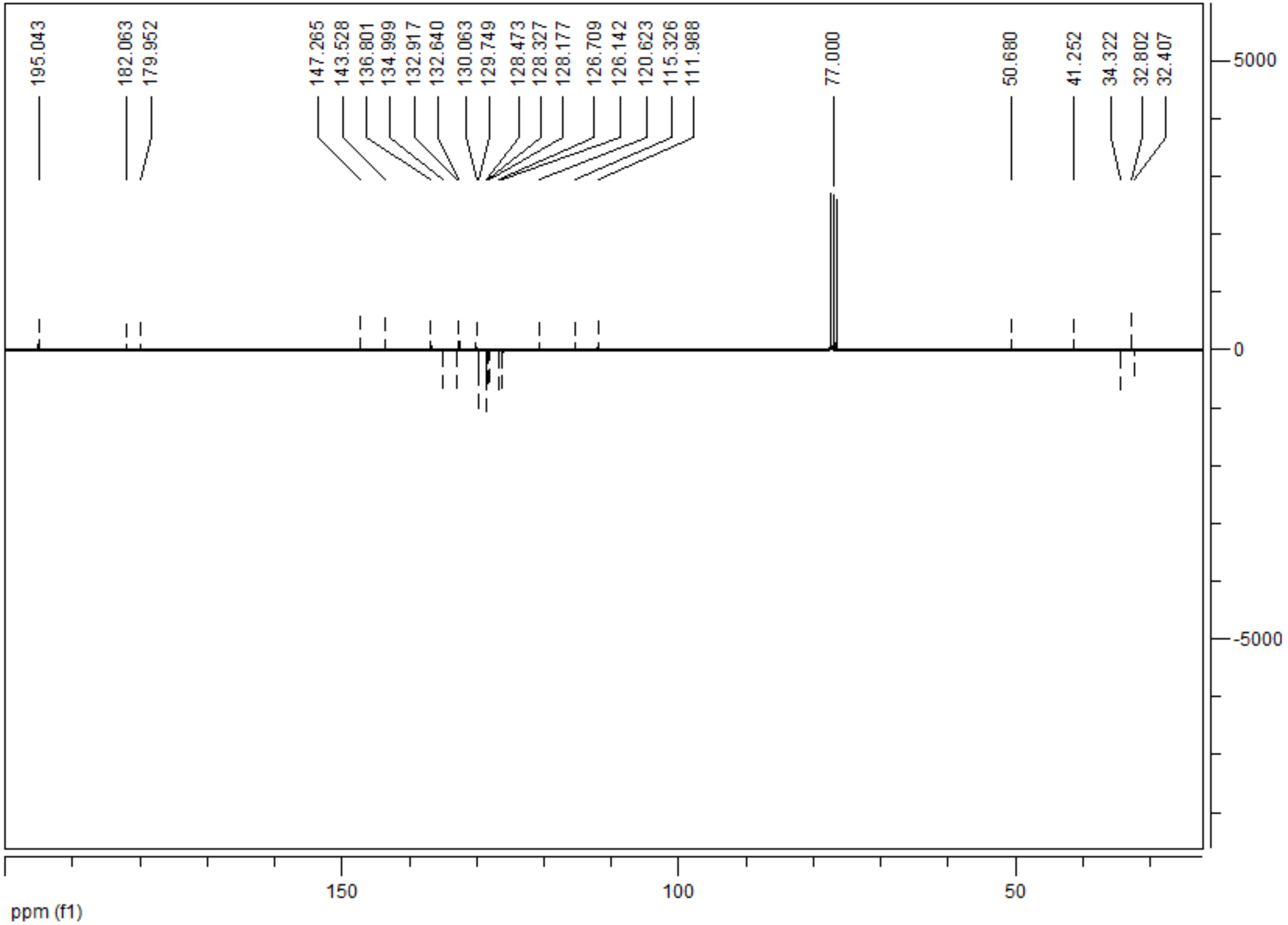

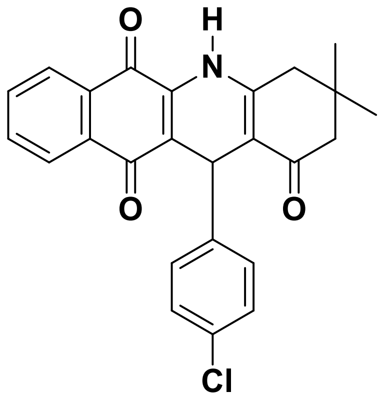

4d

^{13}C NMR spectrum of compound **4d** (75.0 MHz, $CDCl_3$)

+MS, 0.6-1.0min #34-56

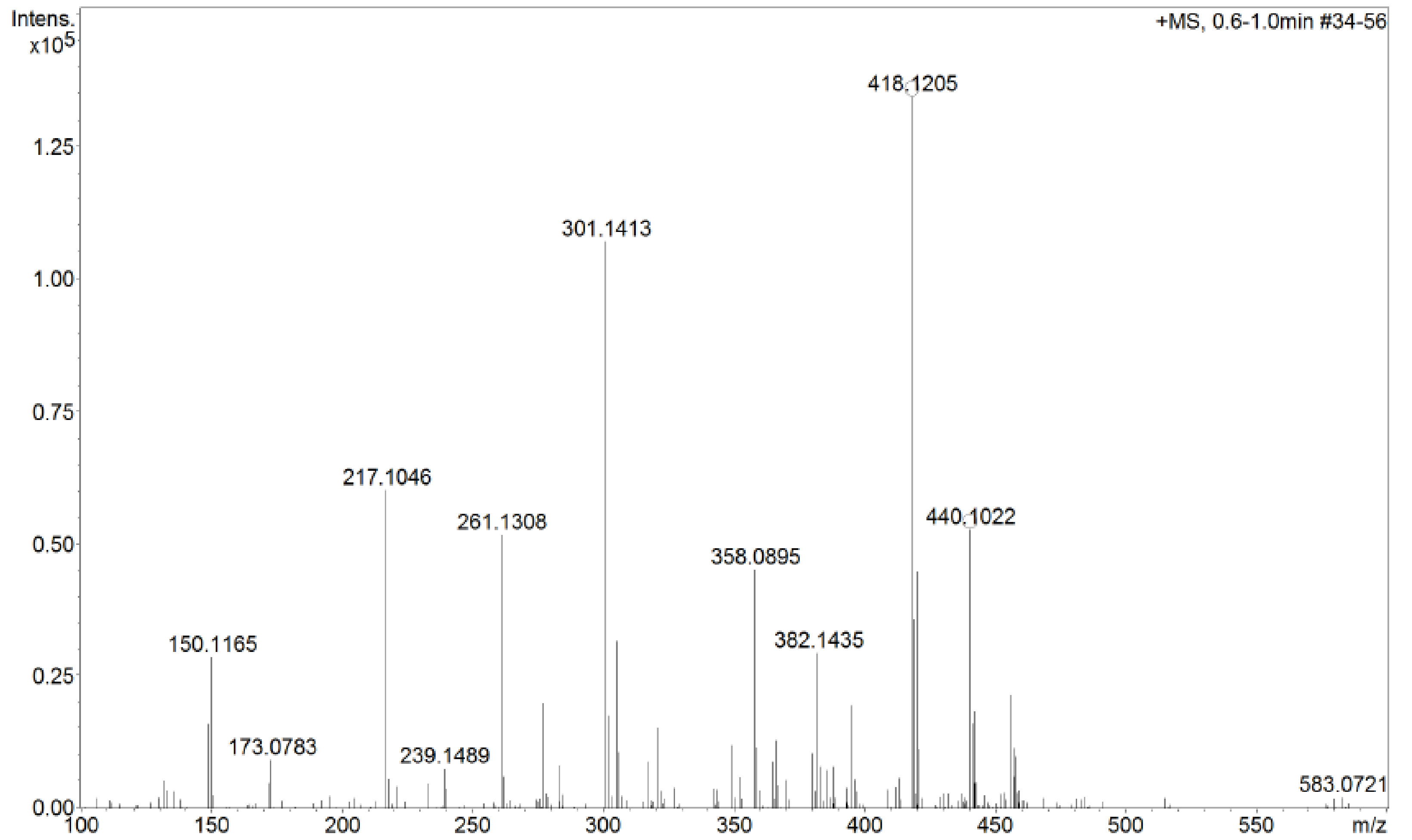

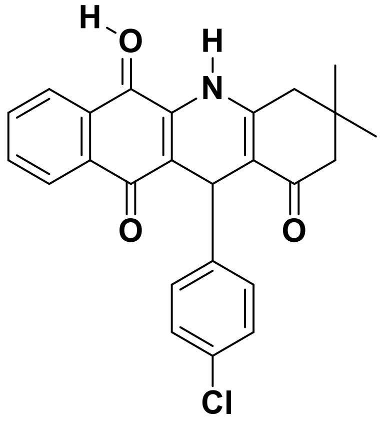

Exact Mass: 418,1210

4d

Meas. m/z	#	Ion Formula	m/z	err [ppm]	Mean err [ppm]	rdb	N-Rule	e⁻ Conf	mSigma	Std I	Std Mean m/z	Std I VarNorm	Std m/z Diff	Std Comb Dev
418.120541	1	C25H21ClNO3	418.120448	-0.2	1.1	15.5	ok	even	14.8	21.2	n.a.	n.a.	n.a.	n.a.
440.102237	1	C25H20ClNNaO3	440.102392	0.4	1.6	15.5	ok	even	13.8	20.5	n.a.	n.a.	n.a.	n.a.

HRMS of compound **4d**

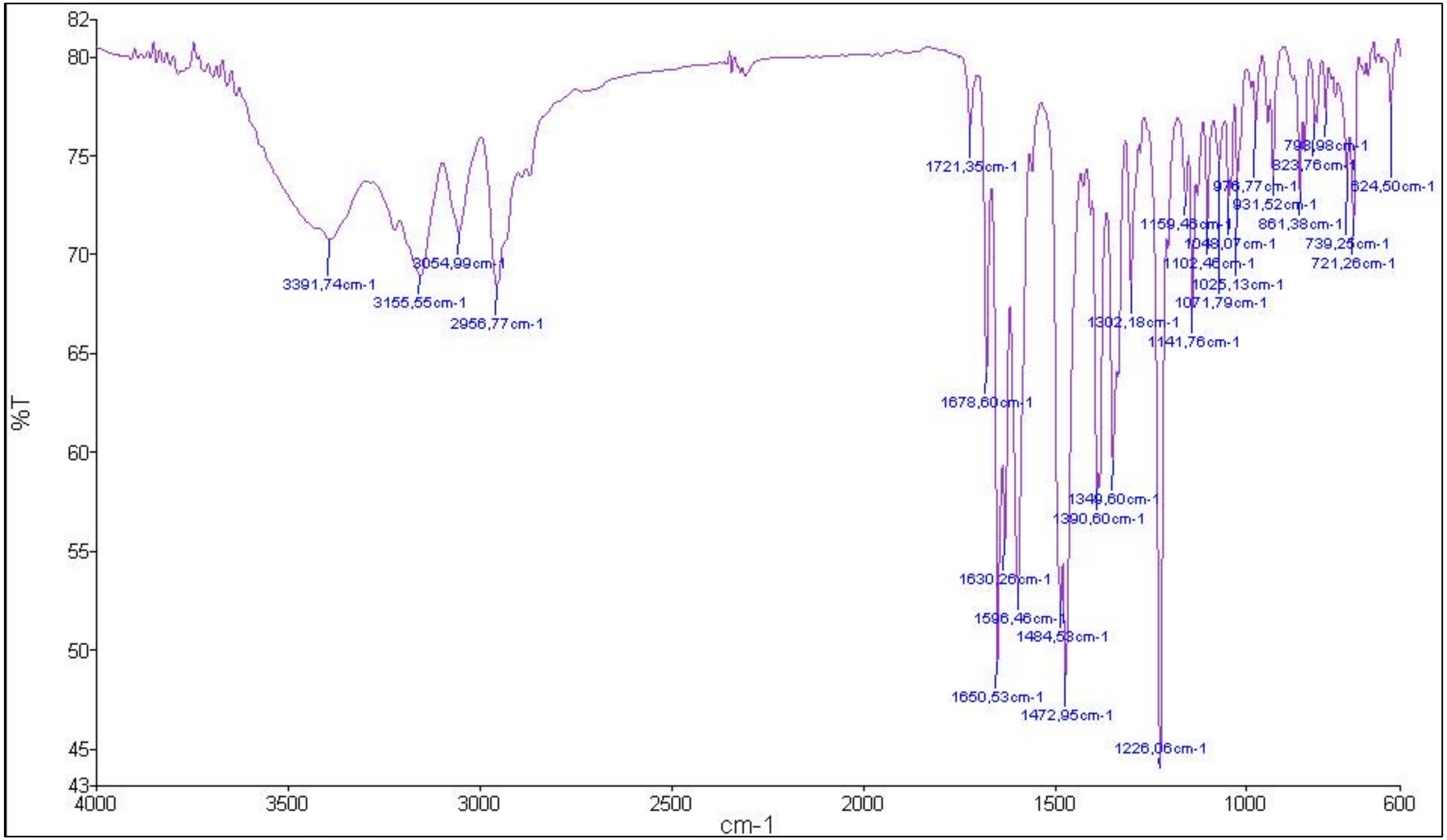

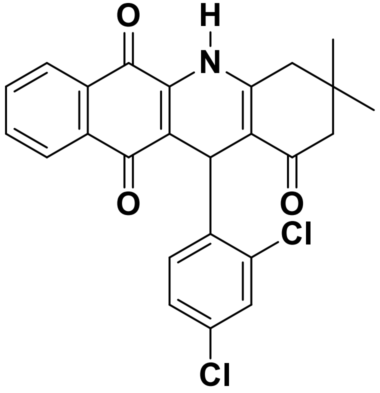

4e

IR spectrum of compound **4e**

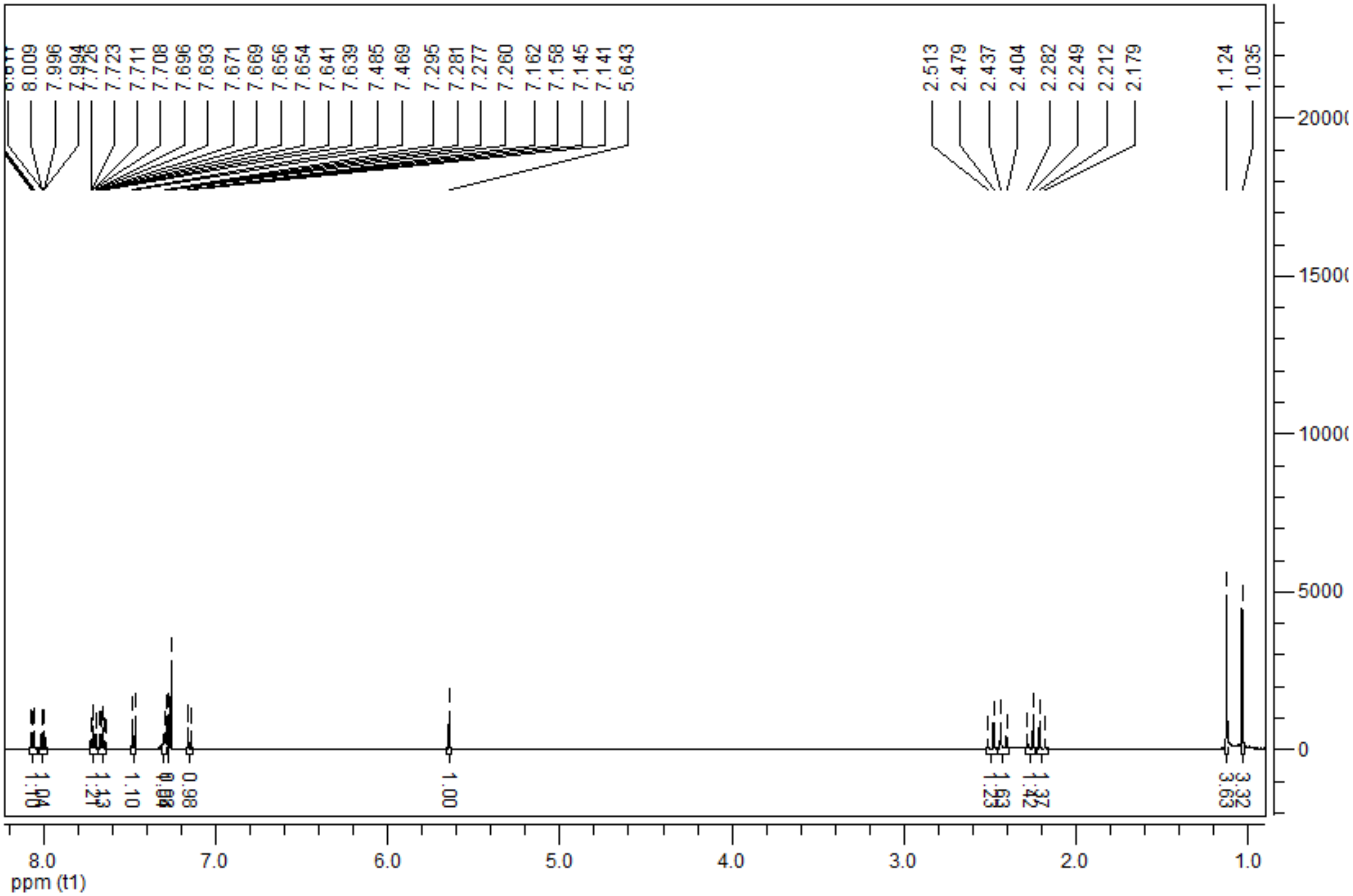

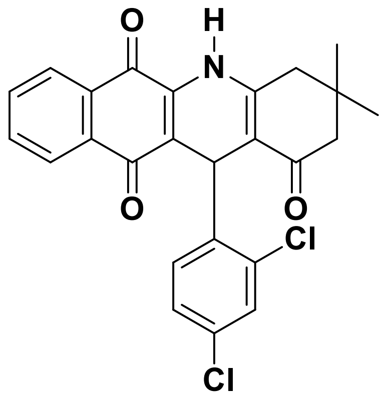

4e

^{1}H NMR spectrum of compound **4e** (500.00 MHz, $CDCl_3$)

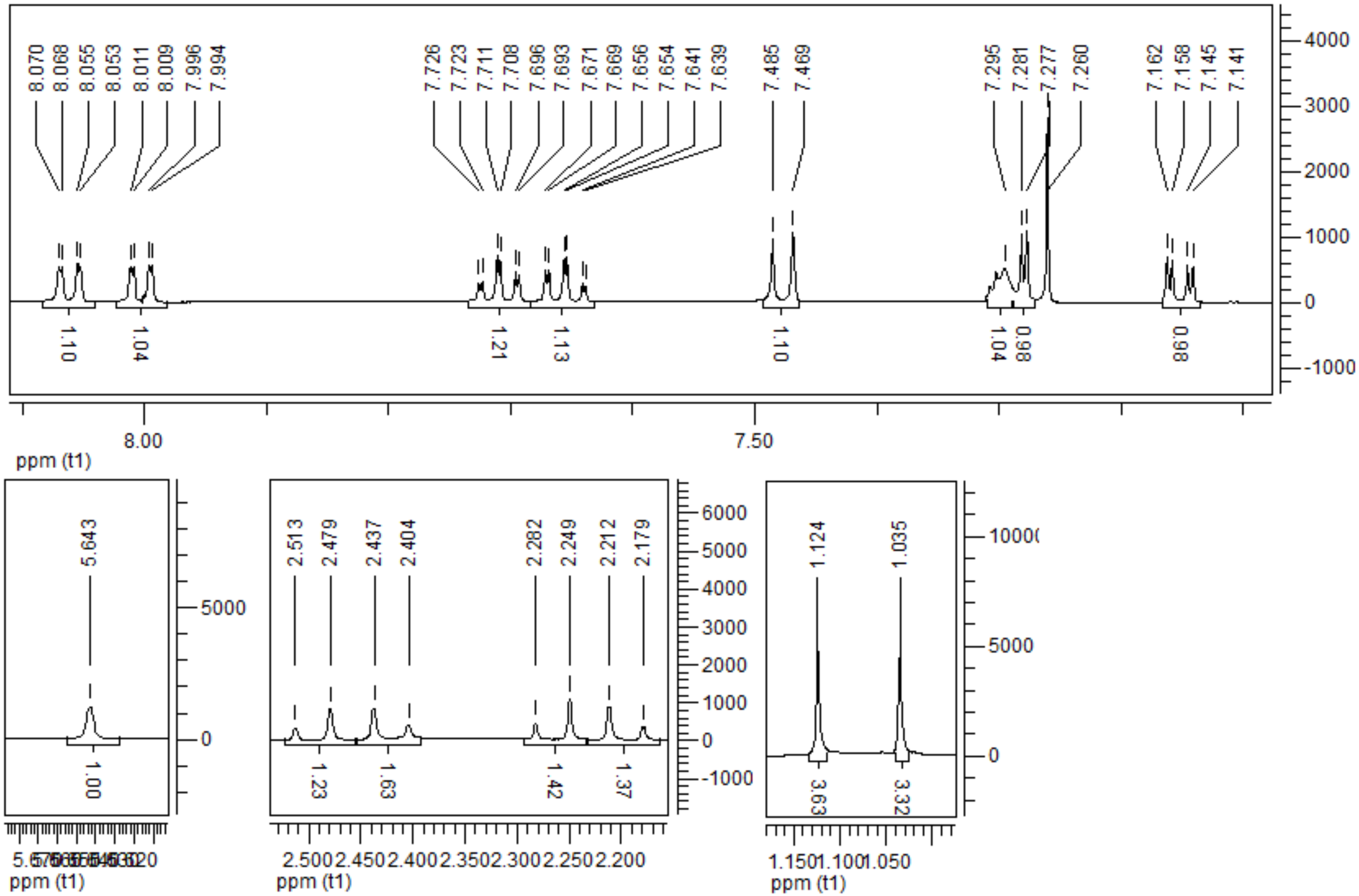

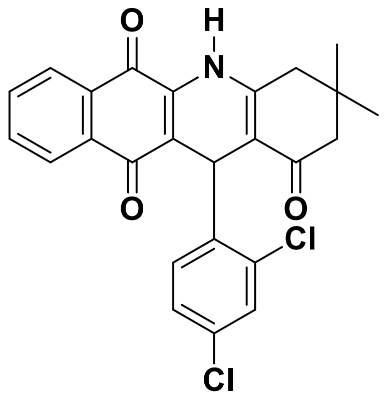

4e

^{1}H NMR spectrum of compound **4e** (500.00 MHz, $CDCl_3$)

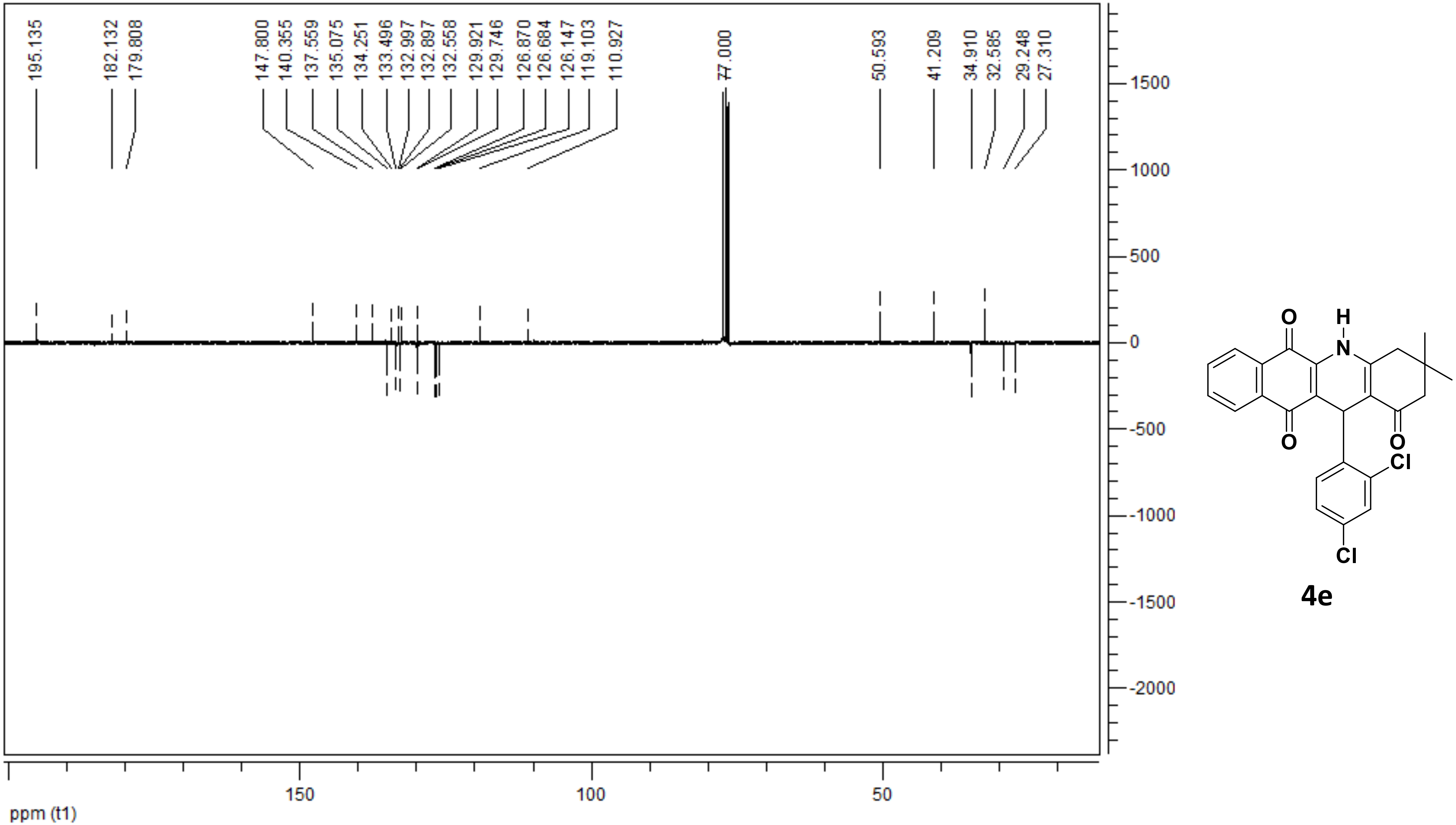

4e

^{13}C NMR spectrum of compound **4e** (75.0 MHz, $CDCl_3$)

+MS, 0.1-0.3min #8-17

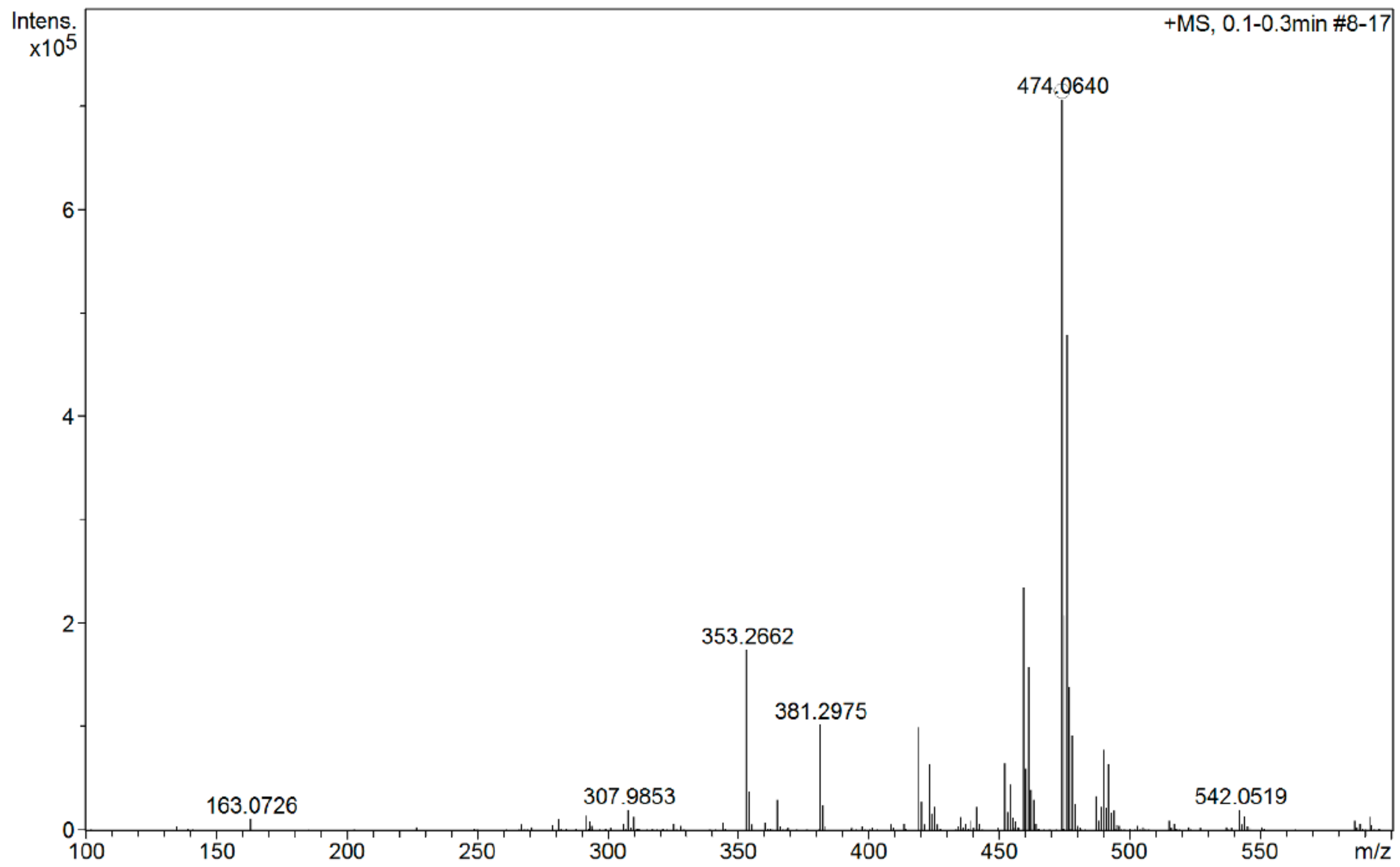

Meas. m/z	#	Ion Formula	m/z	err [ppm]	Mean err [ppm]	rdb	N-Rule	e⁻ Conf	mSigma	Std I	Std Mean m/z	Std I VarNorm	Std m/z Diff	Std Comb Dev
474.063981	1	C25H19Cl2NNaO3	474.063420	-1.2	-2.6	15.5	ok	even	9.4	11.3	n.a.	n.a.	n.a.	n.a.

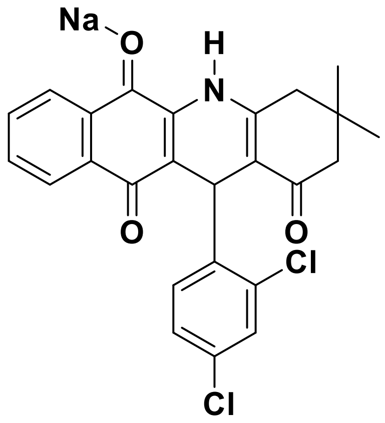

4e

HRMS of compound **4e**

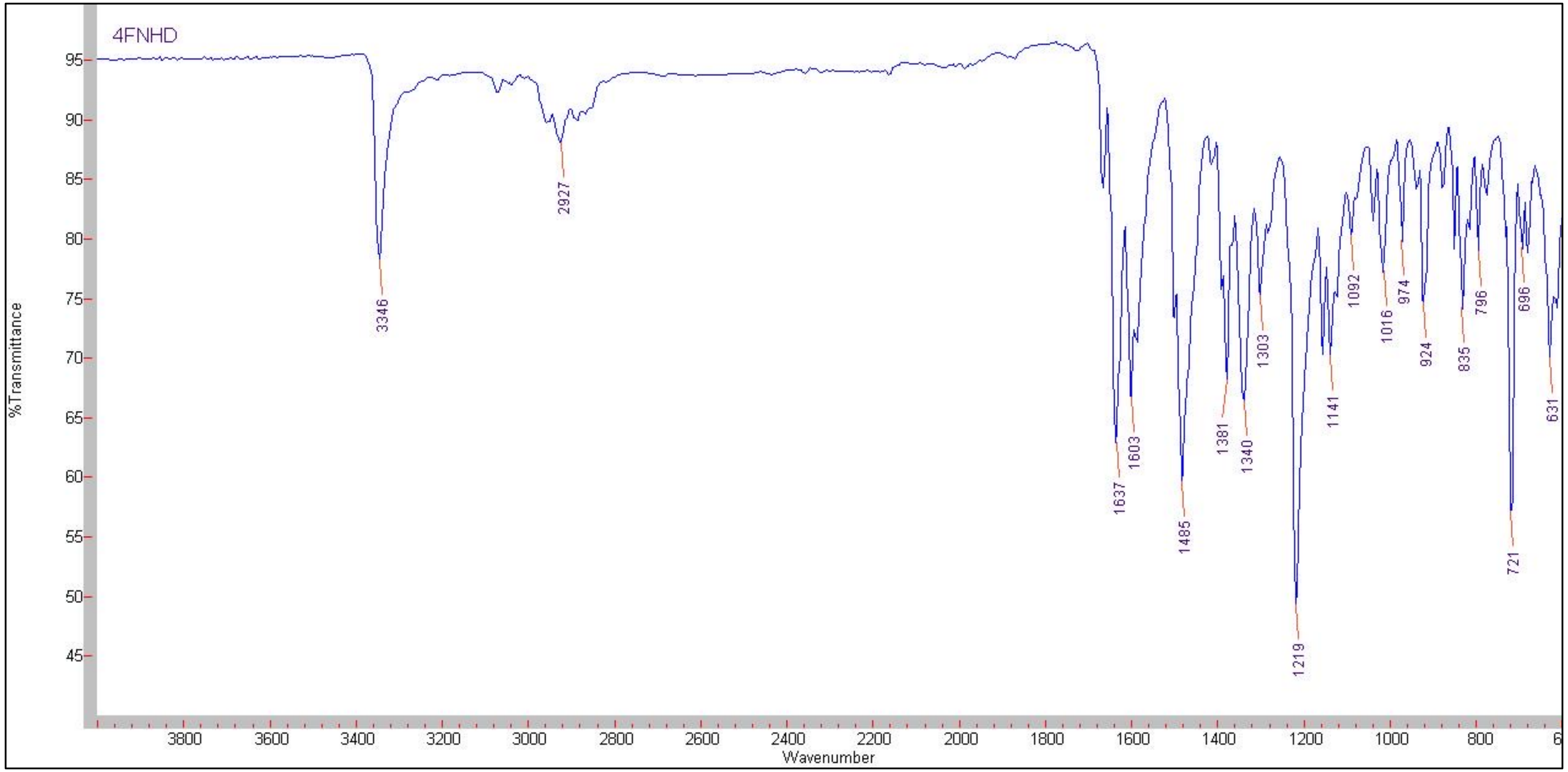

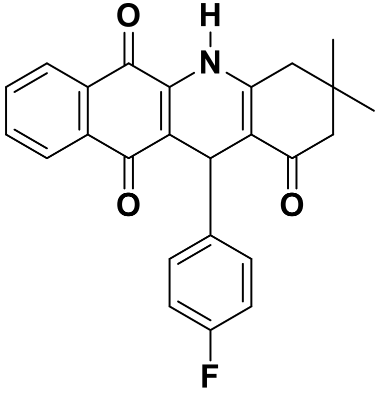

4f

IR spectrum of compound **4f**

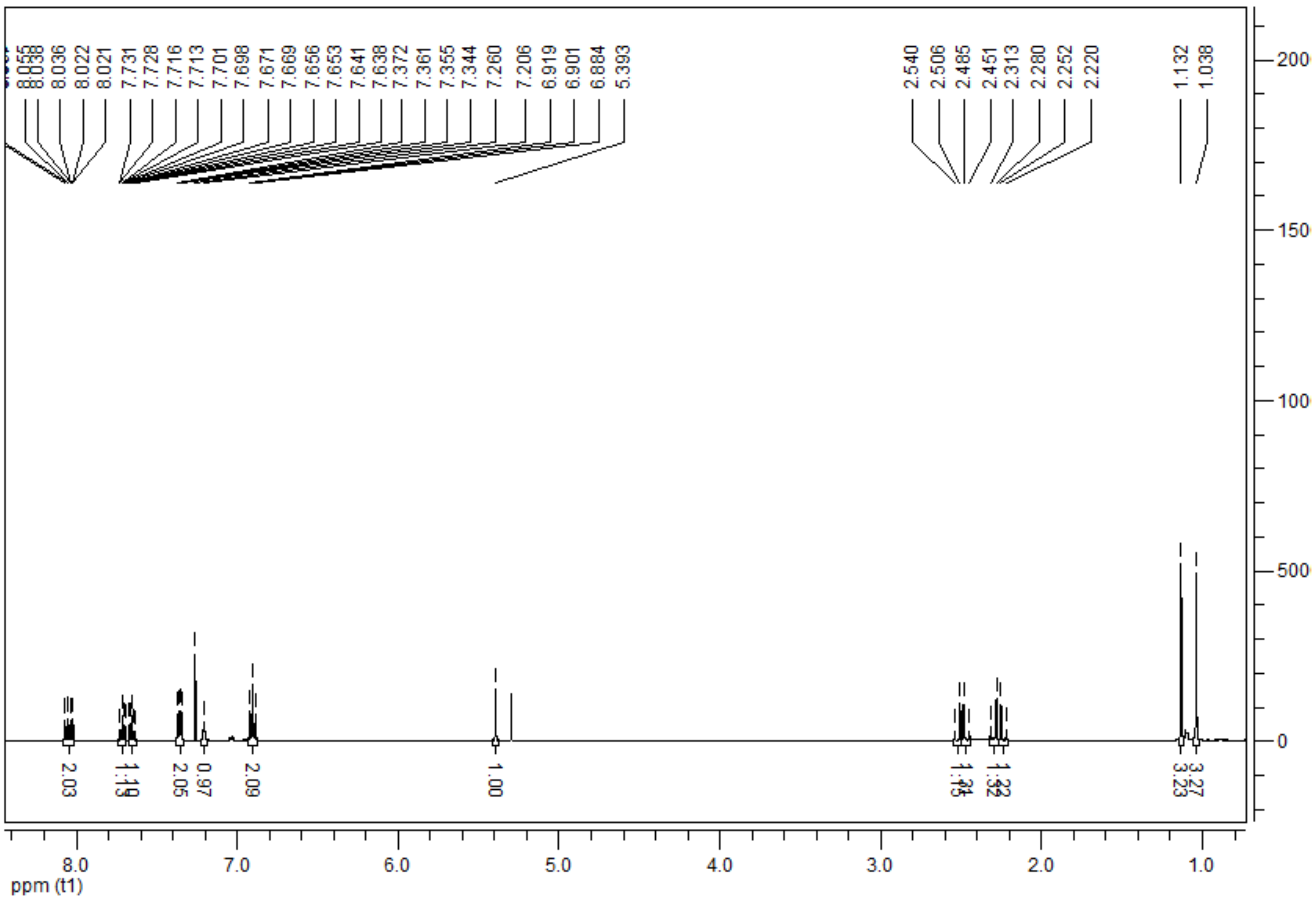

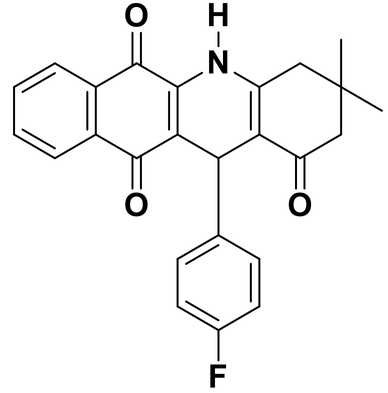

4f

^{1}H NMR spectrum of compound **4f** (500.00 MHz, $CDCl_3$)

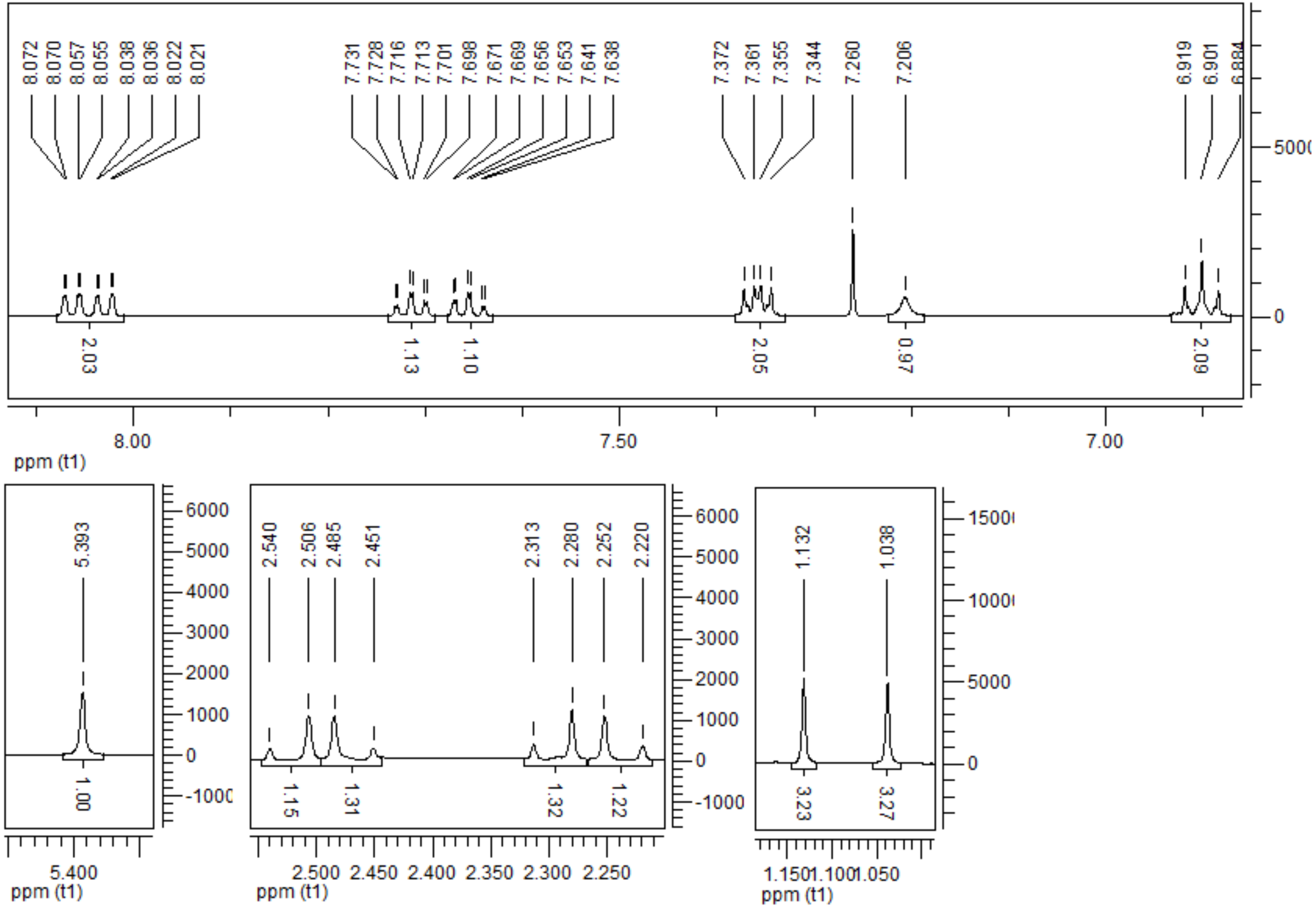

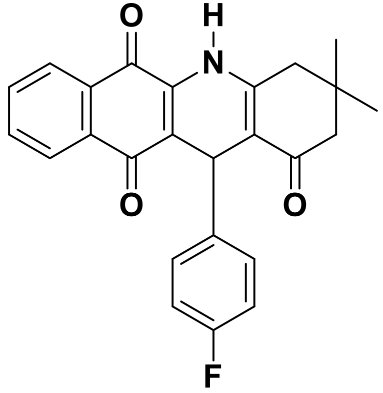

4f

^{1}H NMR spectrum of compound **4f** (500.00 MHz, $CDCl_3$)

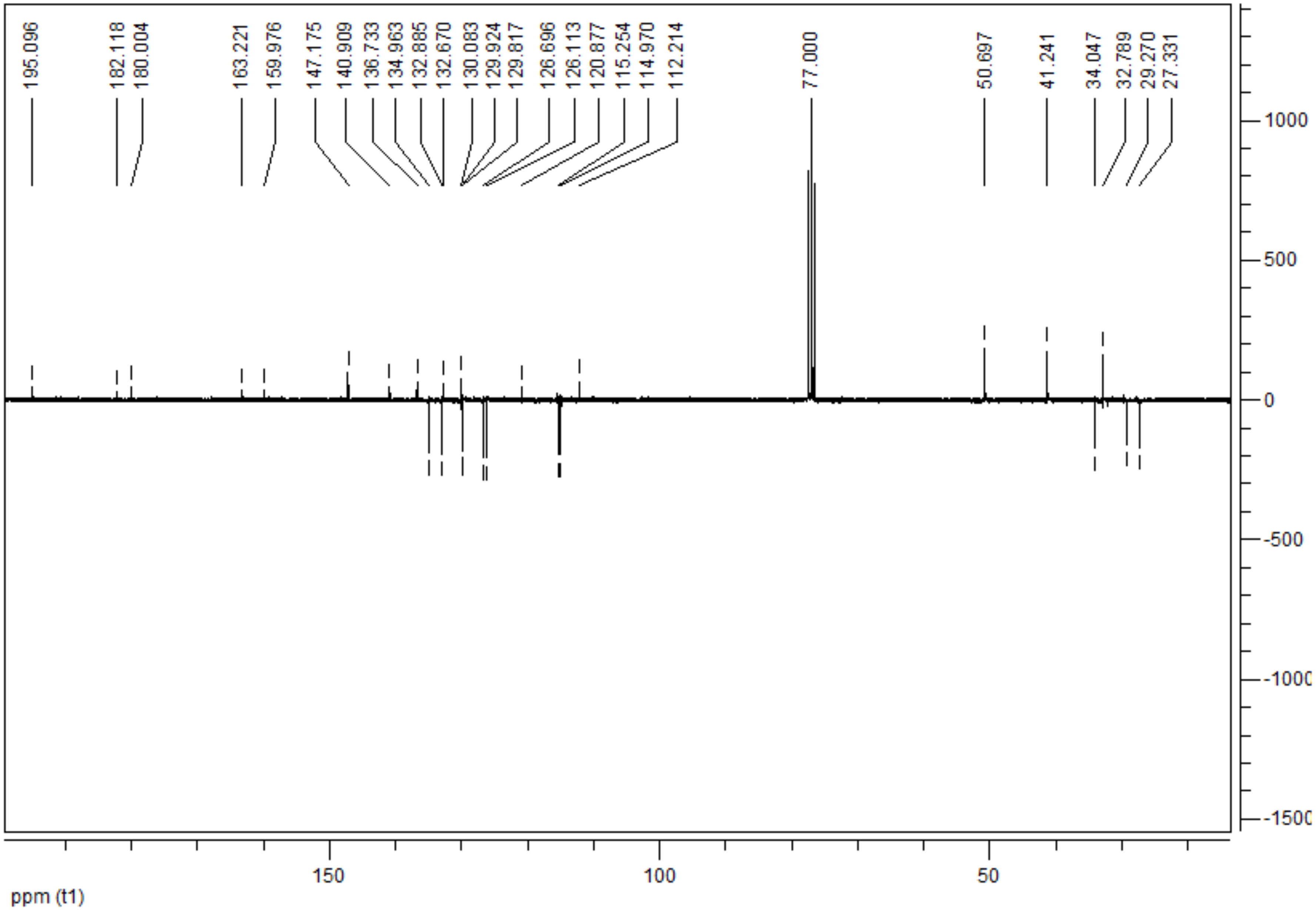

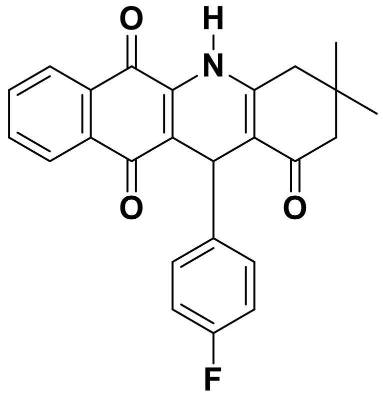

4f

^{13}C NMR spectrum of compound **4f** (75.0 MHz, $CDCl_3$)

+MS, 0.5min #27

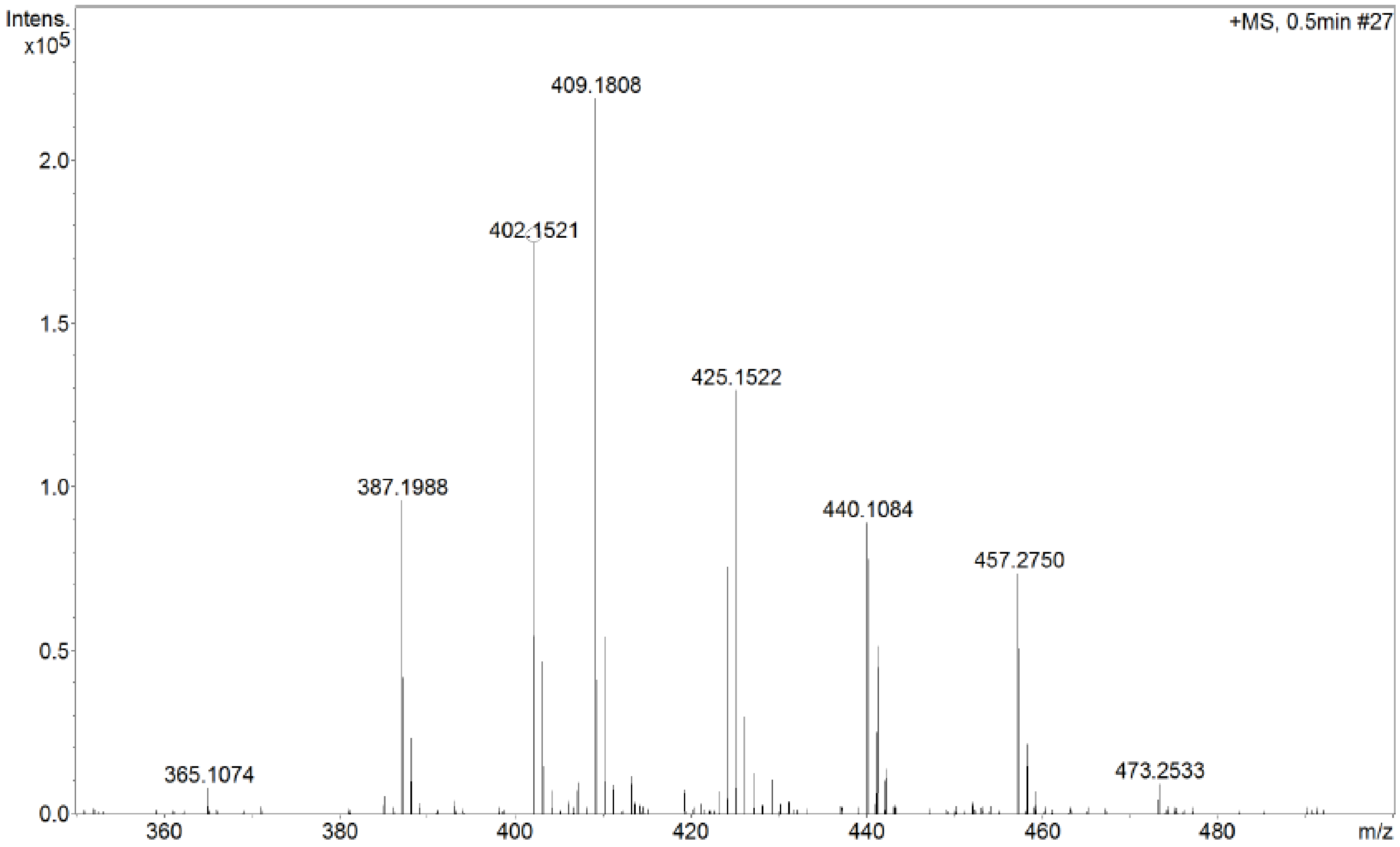

Meas. m/z	#	Ion Formula	m/z	err [ppm]	Mean err [ppm]	rdb	N-Rule	e⁻ Conf	mSigma	Std I	Std Mean m/z	Std I VarNorm	Std m/z Diff	Std Comb Dev
402.152117	1	C25H21FNO3	402.149998	-5.3	-4.4	15.5	ok	even	5.8	8.9	n.a.	n.a.	n.a.	n.a.
	2	C18H25FNO8	402.155871	9.3	9.8	6.5	ok	even	36.7	58.1	n.a.	n.a.	n.a.	n.a.

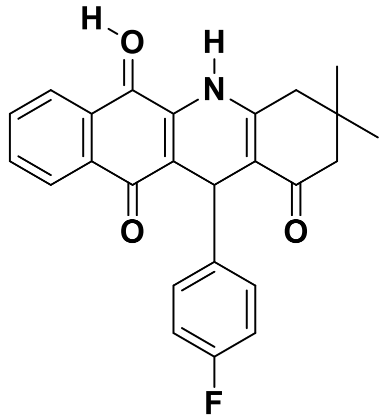

Exact Mass: 402,1505

4f

HRMS of compound **4f**

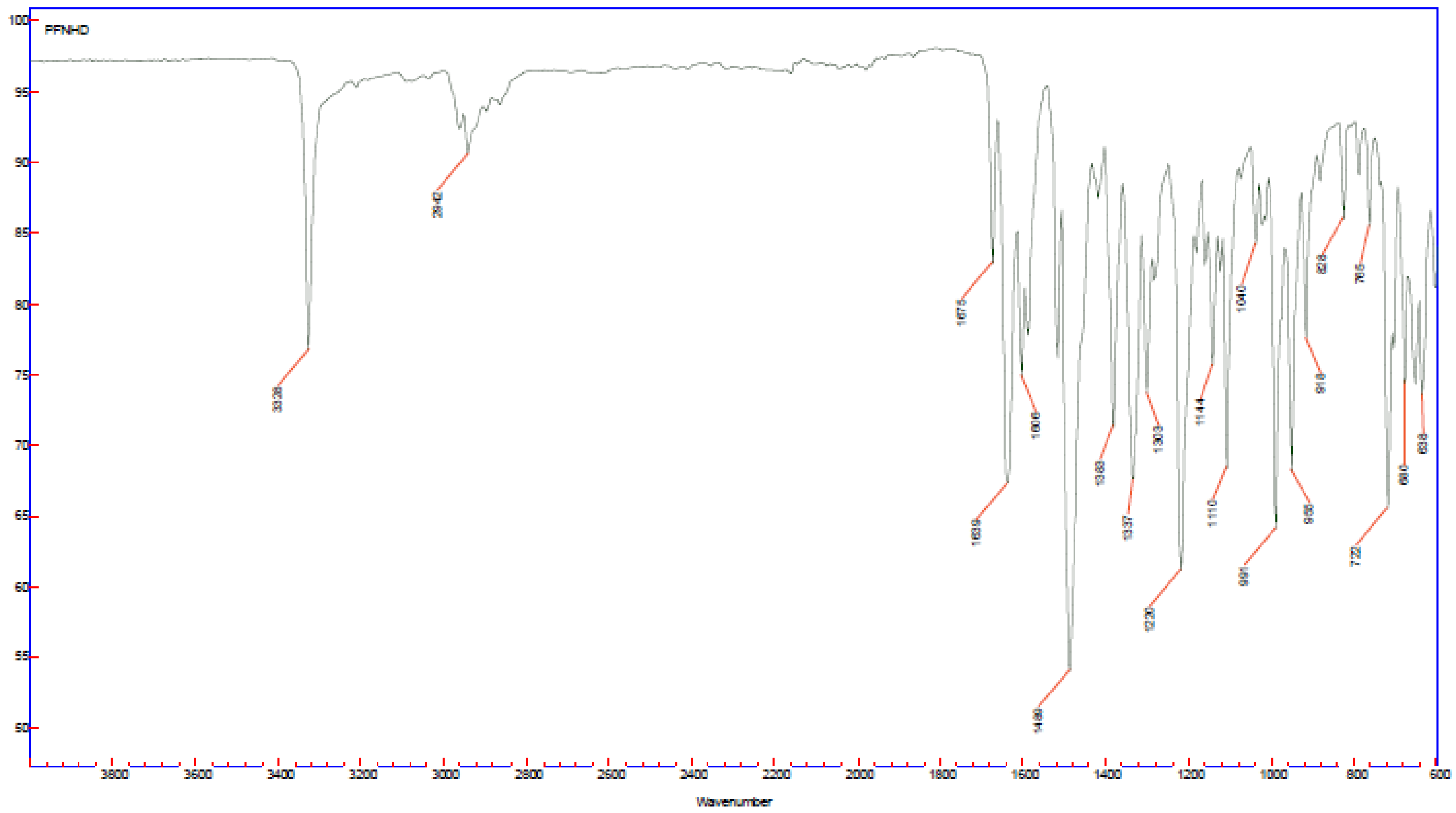

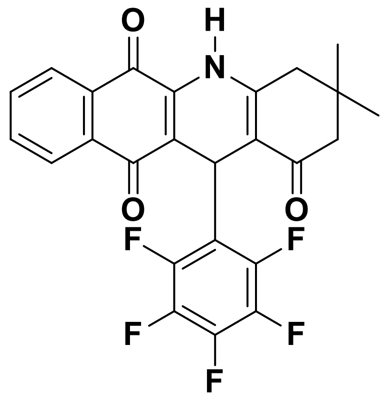

4g

IR spectrum of compound **4g**

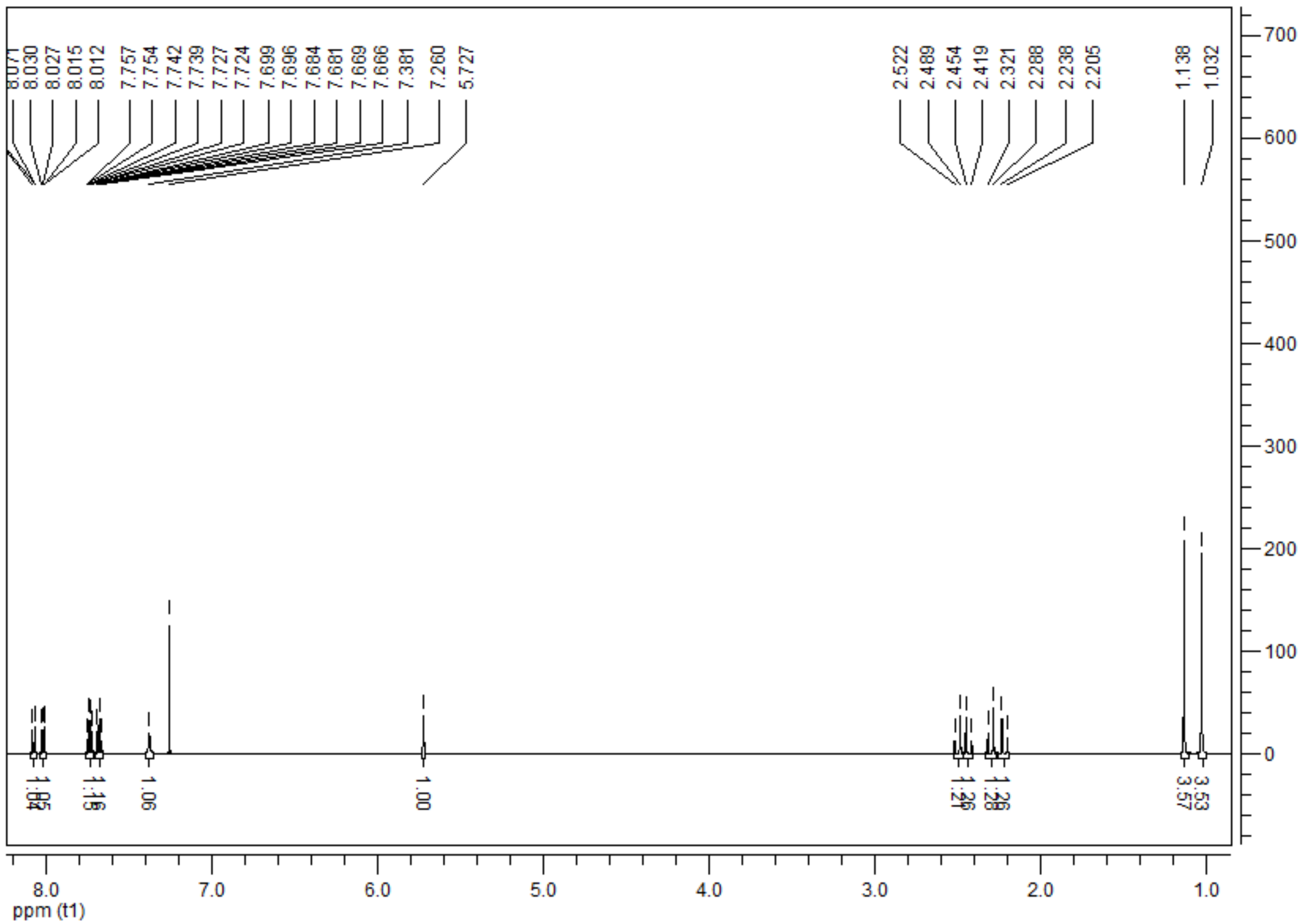

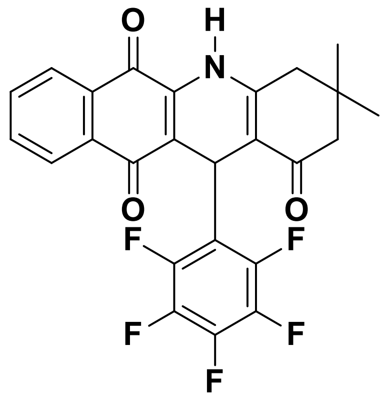
4g

^{1}H NMR spectrum of compound **4g** (500.00 MHz, $CDCl_3$)

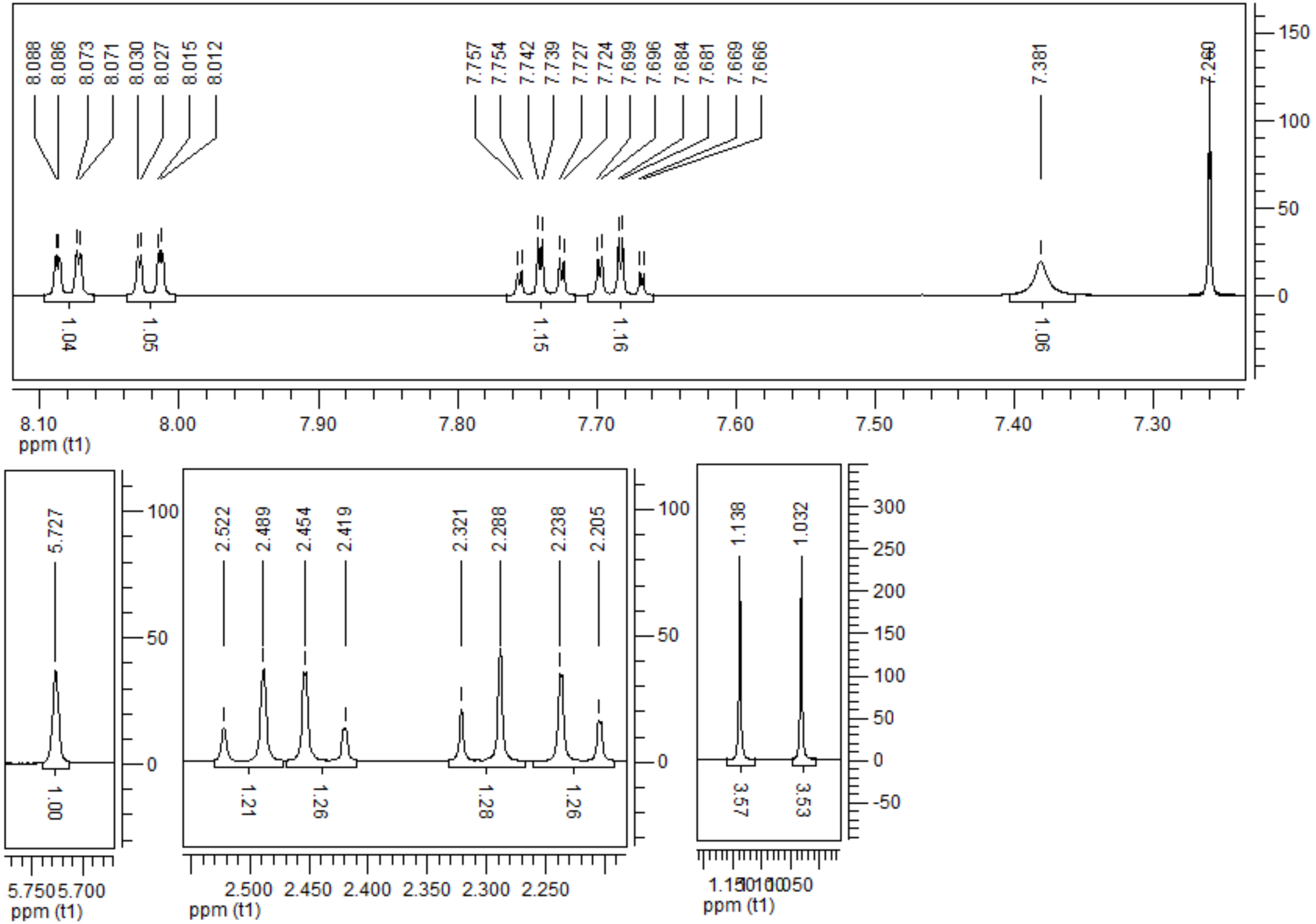

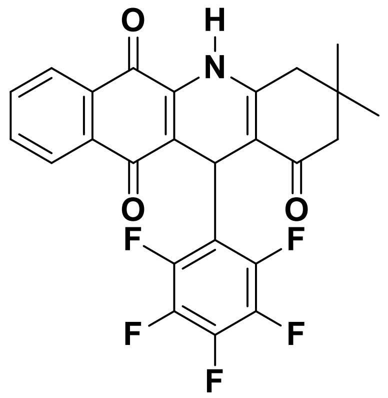

4g

^{1}H NMR spectrum of compound **4g** (500.00 MHz, $CDCl_3$)

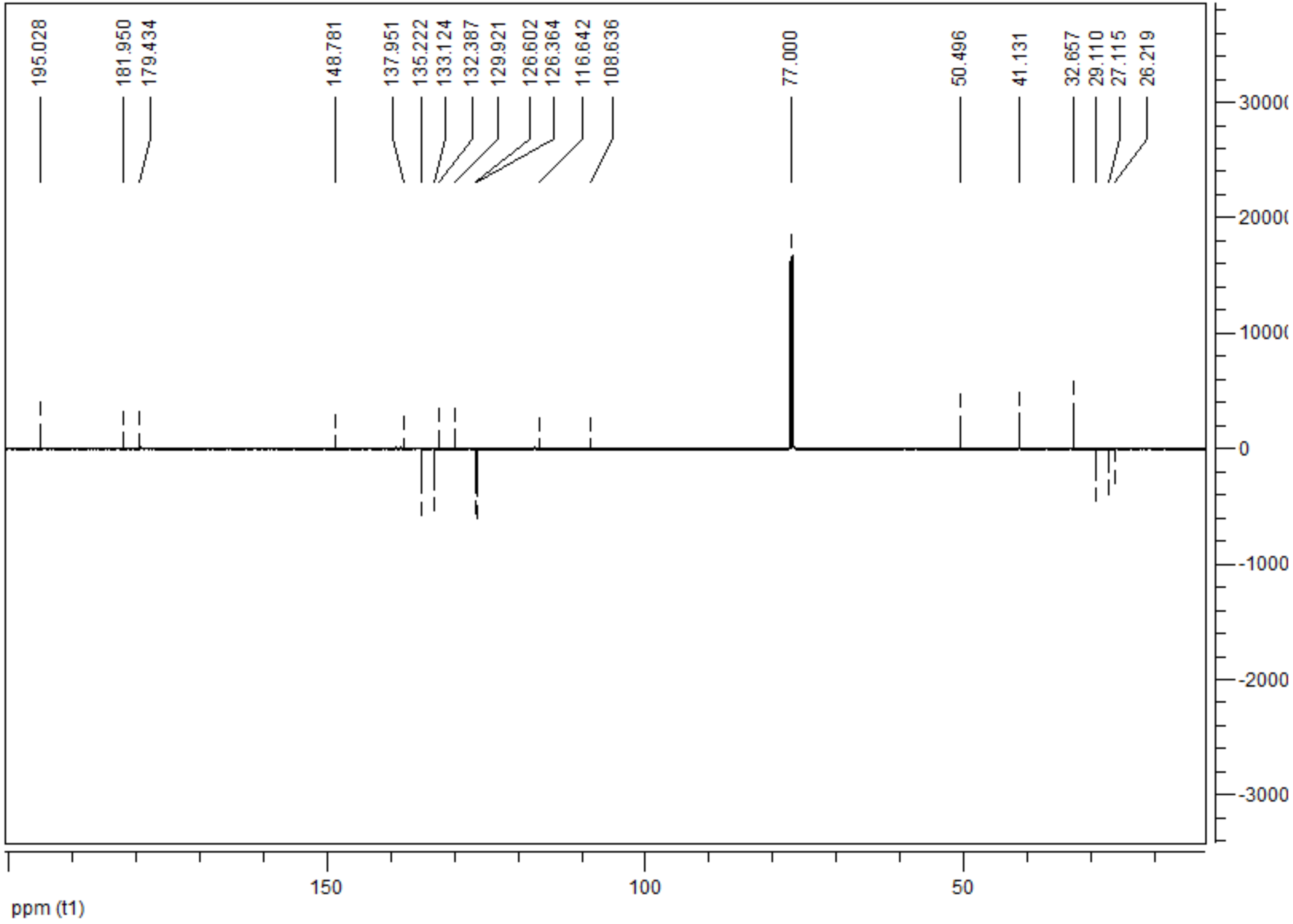

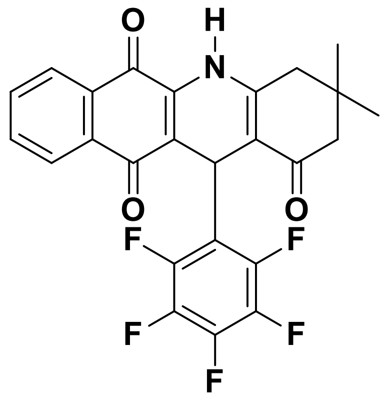

4g

^{13}C NMR spectrum of compound **4g** (125.0 MHz, $CDCl_3$)

+MS, 0.1-0.7min #4-42

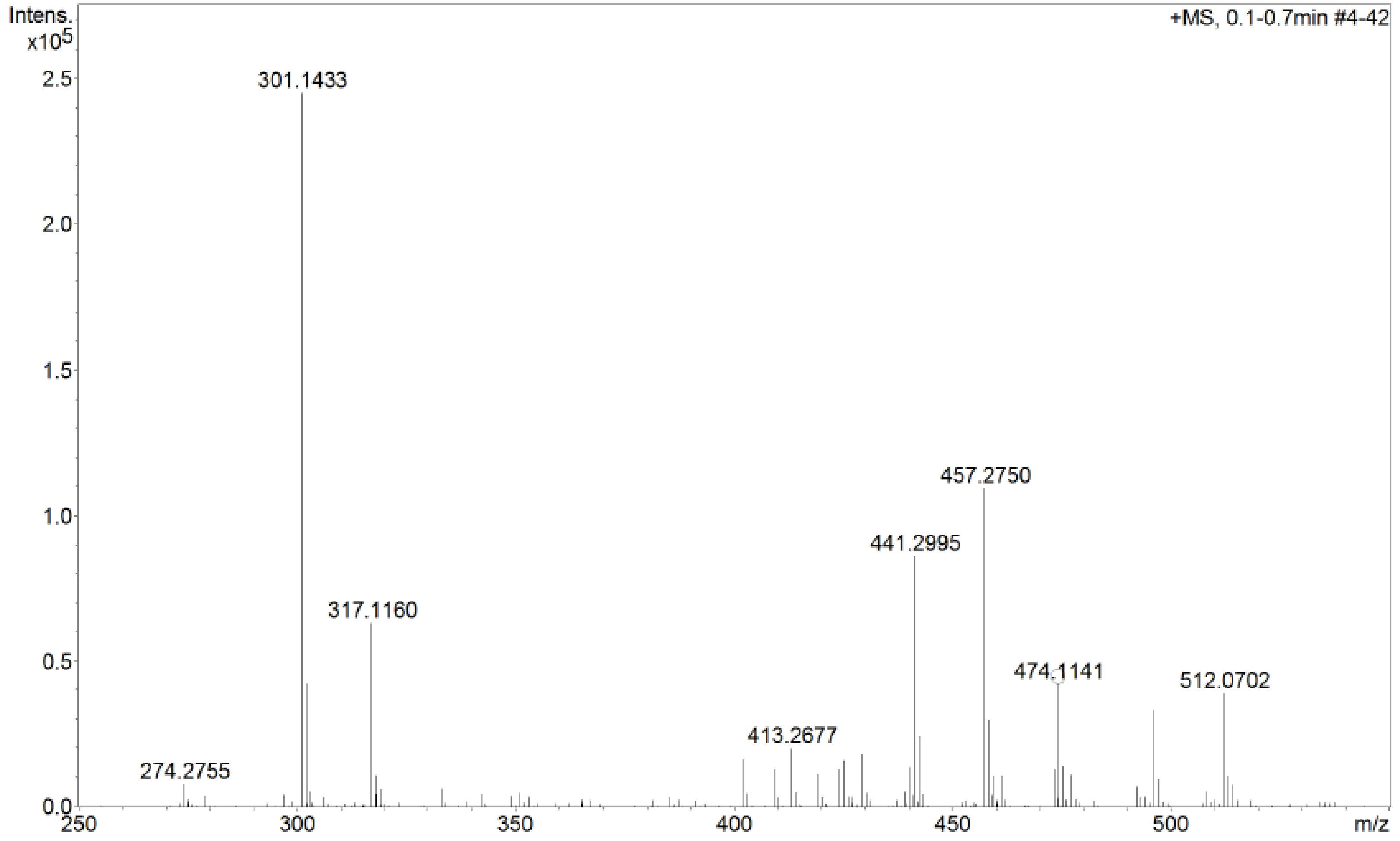

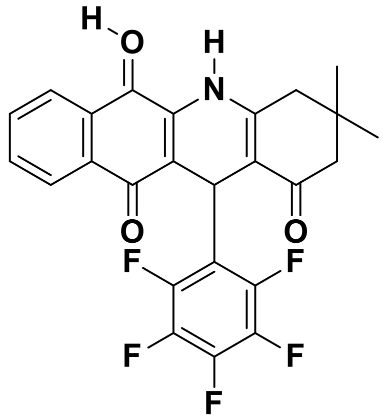

Exact Mass: 474,1129

4g

Meas. m/z	#	Ion Formula	m/z	err [ppm]	Mean err [ppm]	rdb	N-Rule	e⁻ Conf	mSigma	Std I	Std Mean m/z	Std I VarNorm	Std m/z Diff	Std Comb Dev
474.114109	1	$C_{25}H_{17}F_5NO_3$	474.112311	-3.8	-2.1	15.5	ok	even	35.0	52.4	n.a.	n.a.	n.a.	n.a.

HRMS of compound **4g**

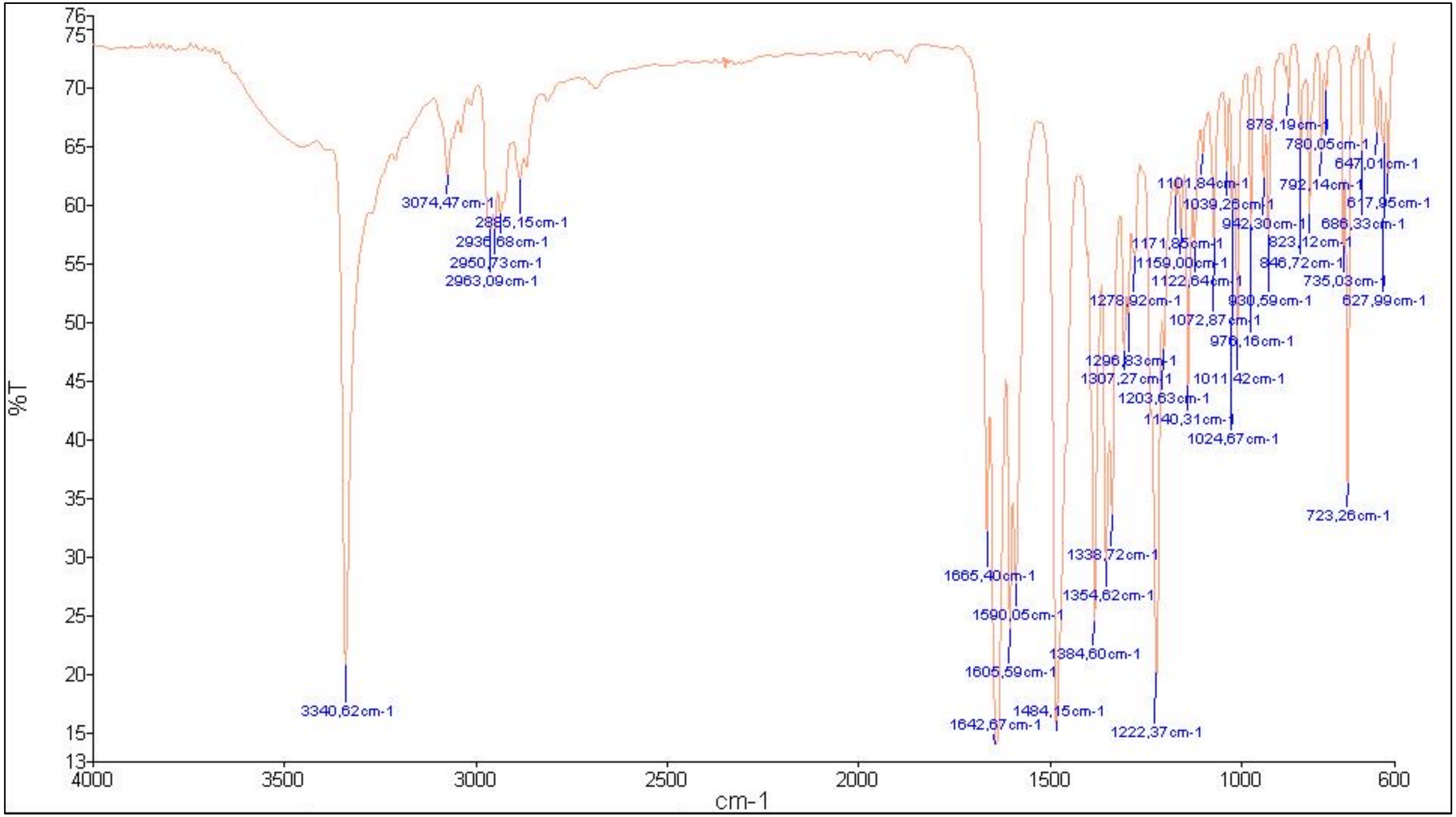

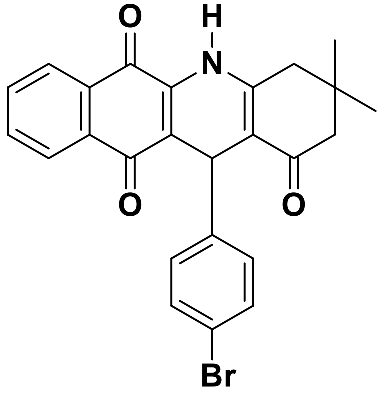

4h

IR spectrum of compound **4h**

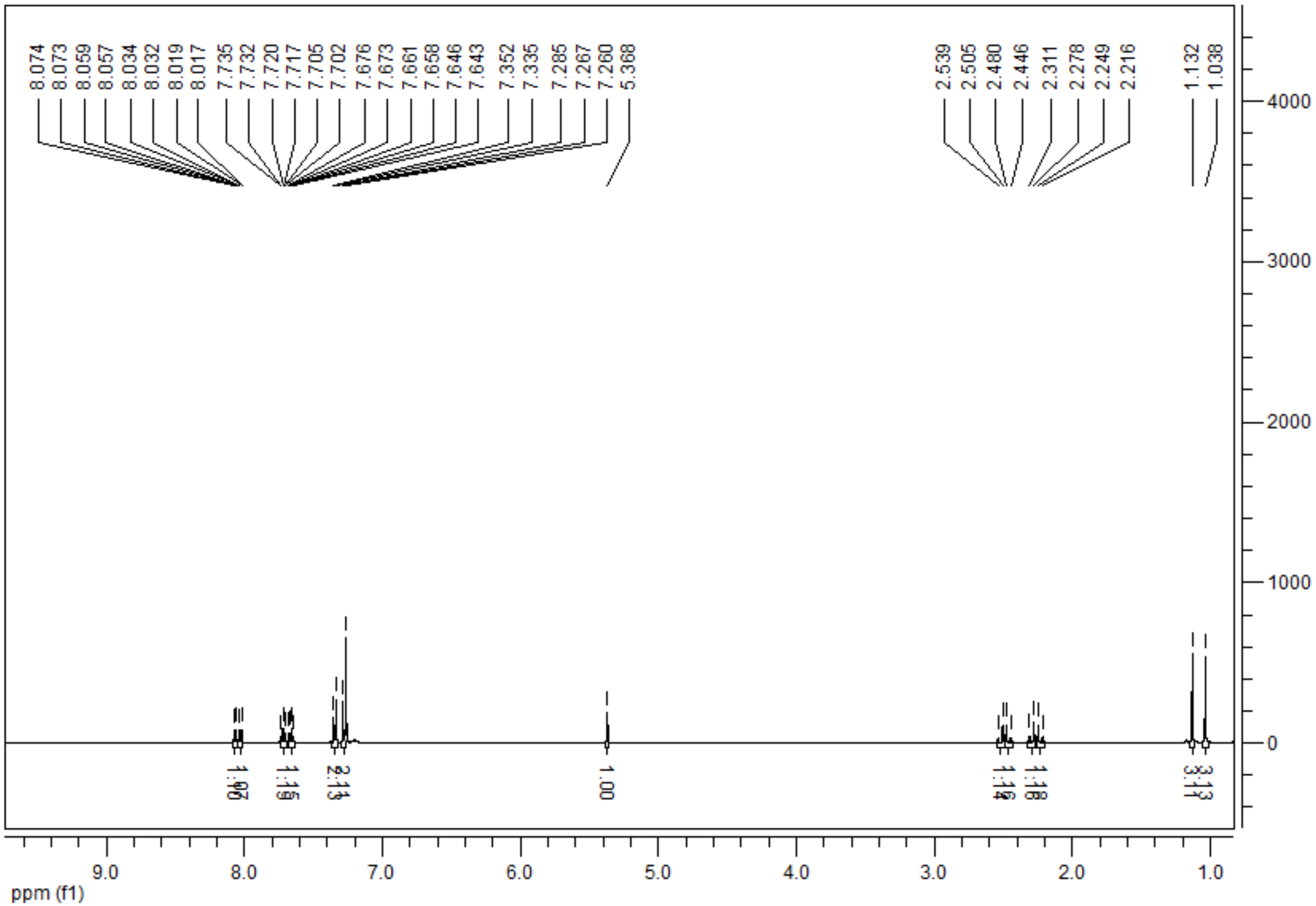

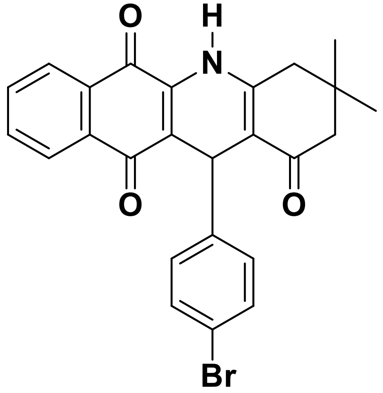

4h

^{1}H NMR spectrum of compound **4h** (500.00 MHz, $CDCl_3$)

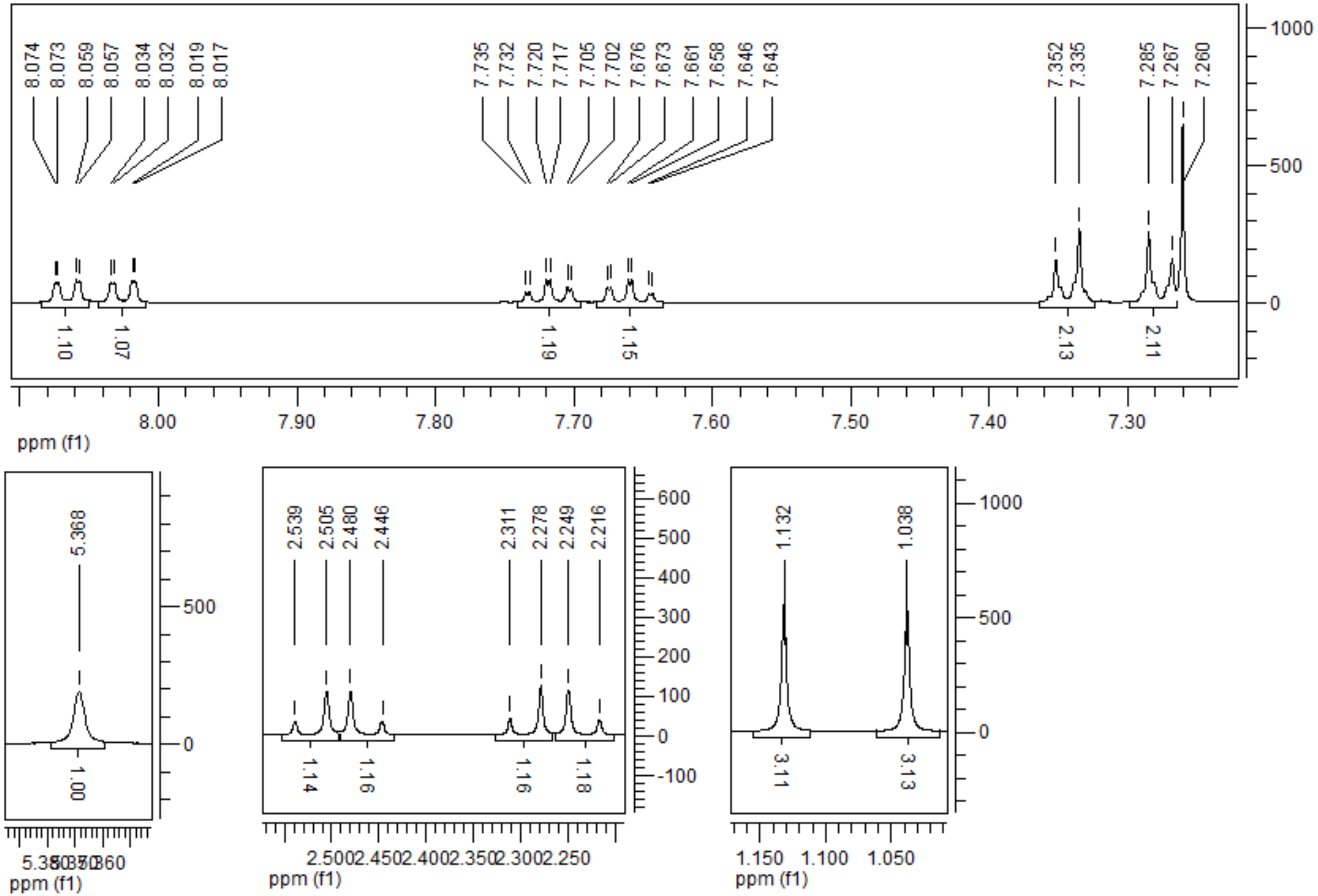

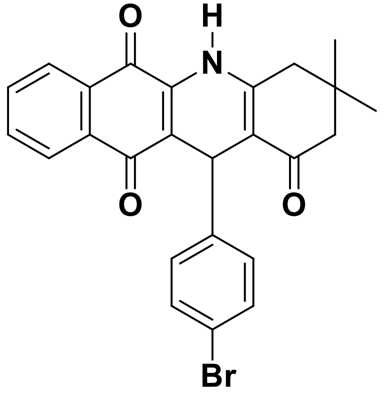

4h

^{1}H NMR spectrum of compound **4h** (500.00 MHz, $CDCl_3$)

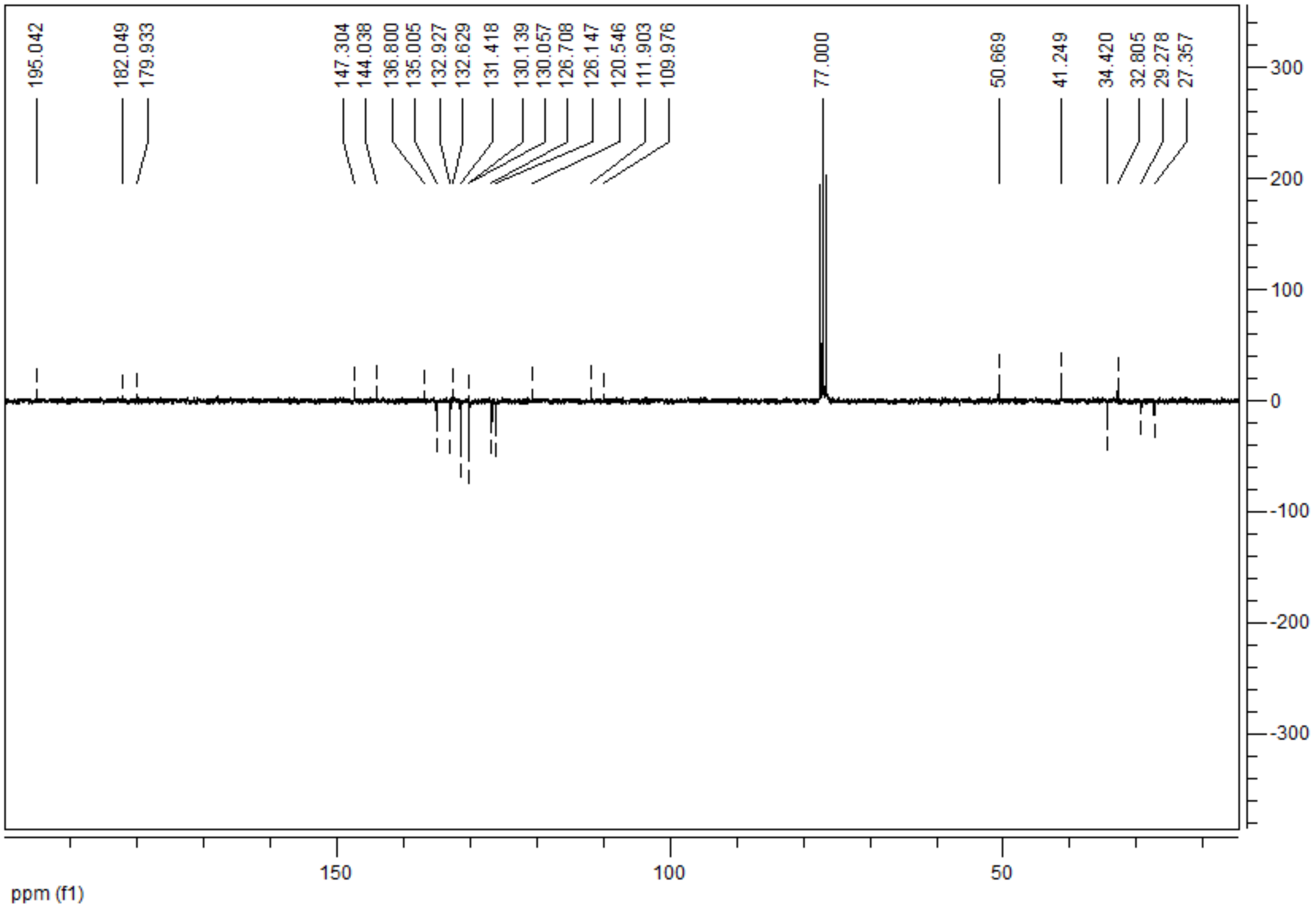

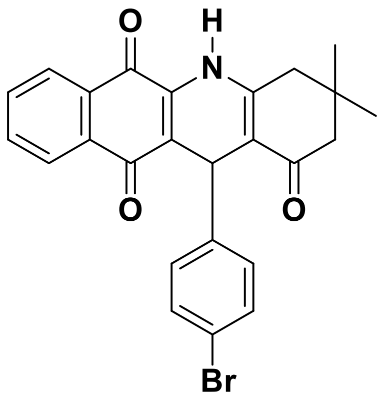

4h

^{13}C NMR spectrum of compound **4h** (75.0 MHz, $CDCl_3$)

+MS, 0.2-0.5min #10-28

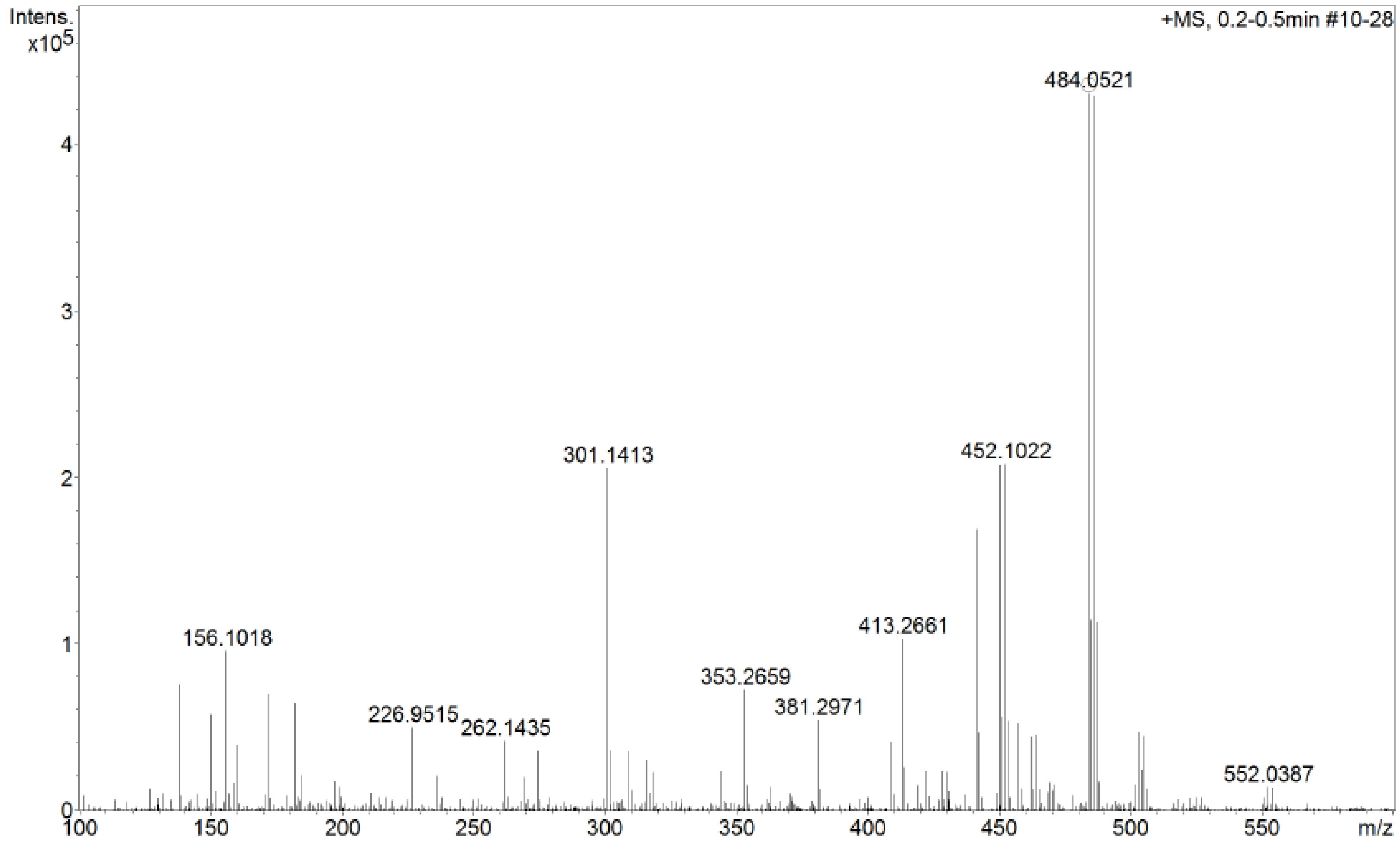

Meas. m/z	#	Ion Formula	m/z	err [ppm]	Mean err [ppm]	rdb	N-Rule	e⁻ Conf	mSigma	Std I	Std Mean m/z	Std I VarNorm	Std m/z Diff	Std Comb Dev
484.052074	1	C25H20BrNNaO3	484.051876	-0.4	0.2	15.5	ok	even	8.3	7.4	n.a.	n.a.	n.a.	n.a.
	2	C21H16BrN7NaO	484.049191	-6.0	-6.0	16.5	ok	even	10.2	10.5	n.a.	n.a.	n.a.	n.a.

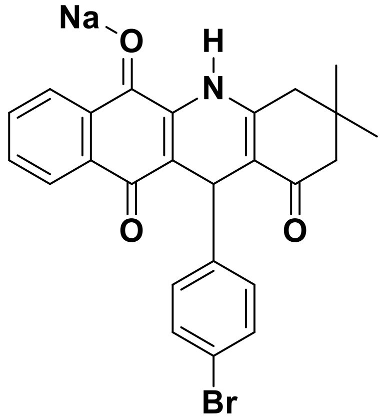

Exact Mass: 484,0524

4h

HRMS of compound **4h**

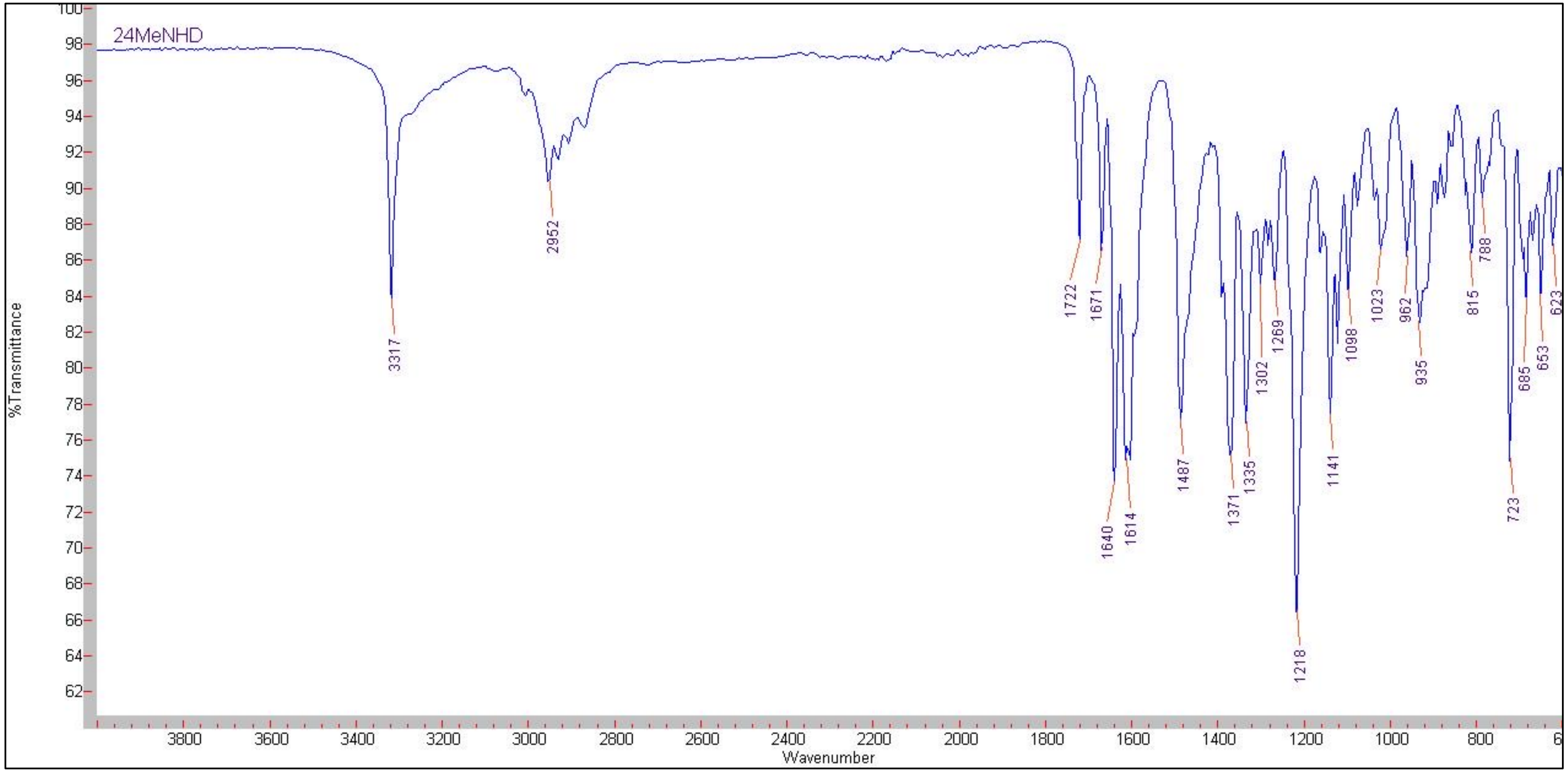

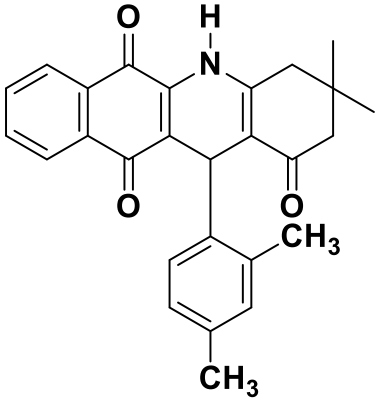

4i

IR spectrum of compound **4i**

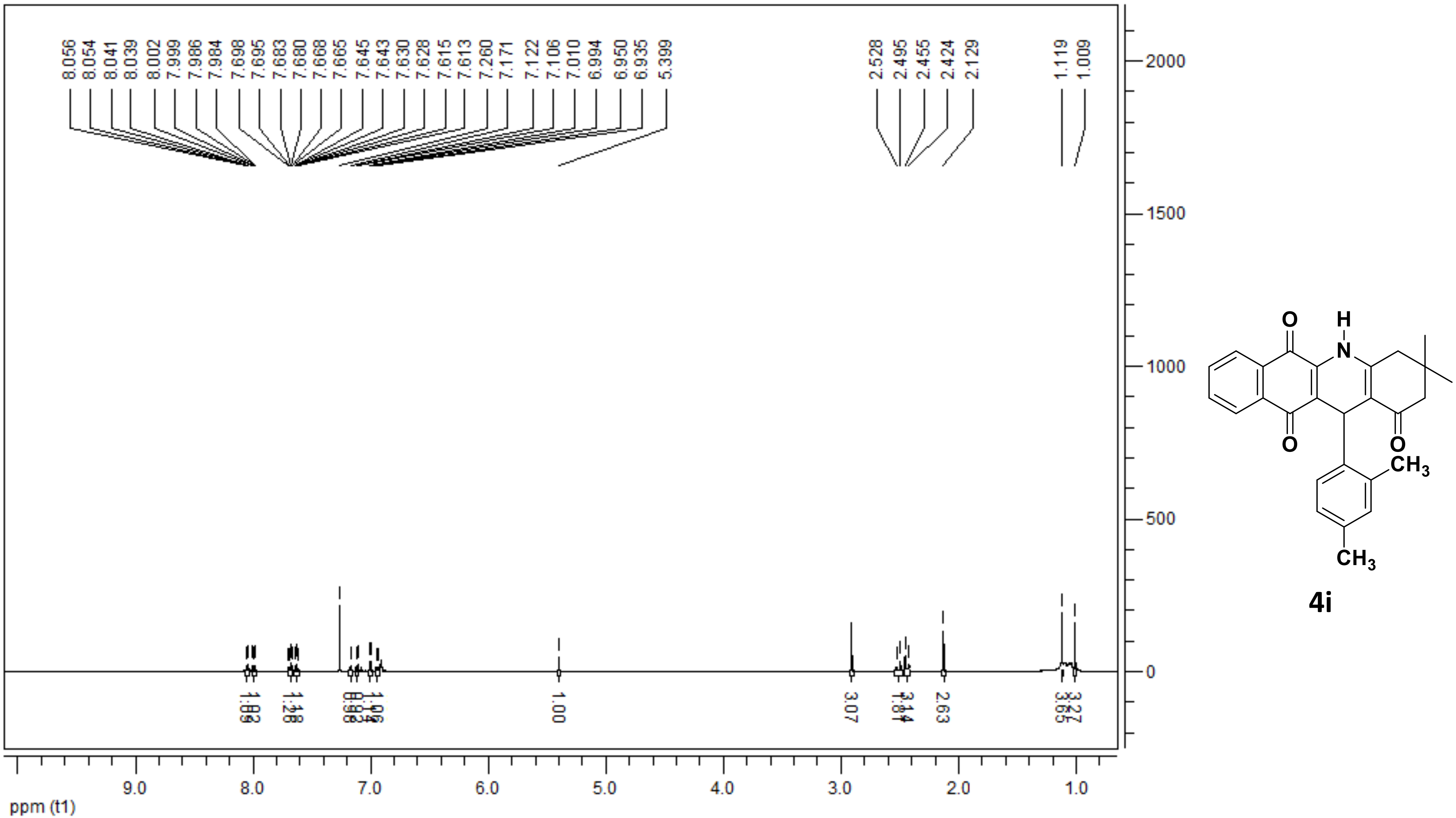

4i

^1H NMR spectrum of compound **4i** (500.00 MHz, $CDCl_3$)

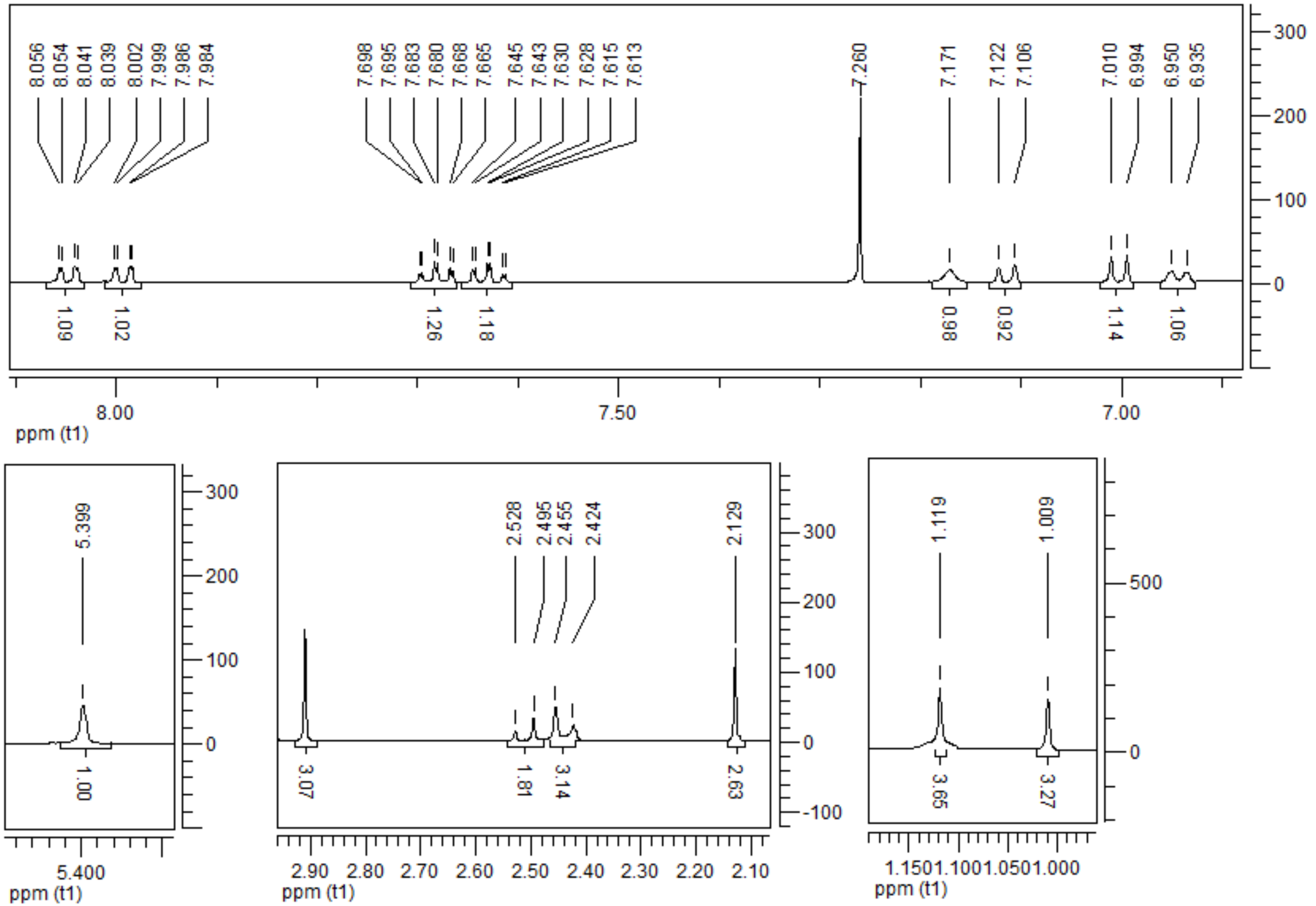

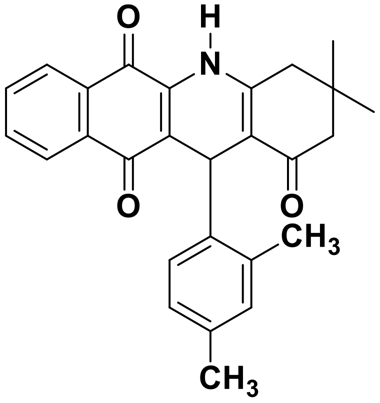

4i

^{1}H NMR spectrum of compound **4i** (500.00 MHz, $CDCl_3$)

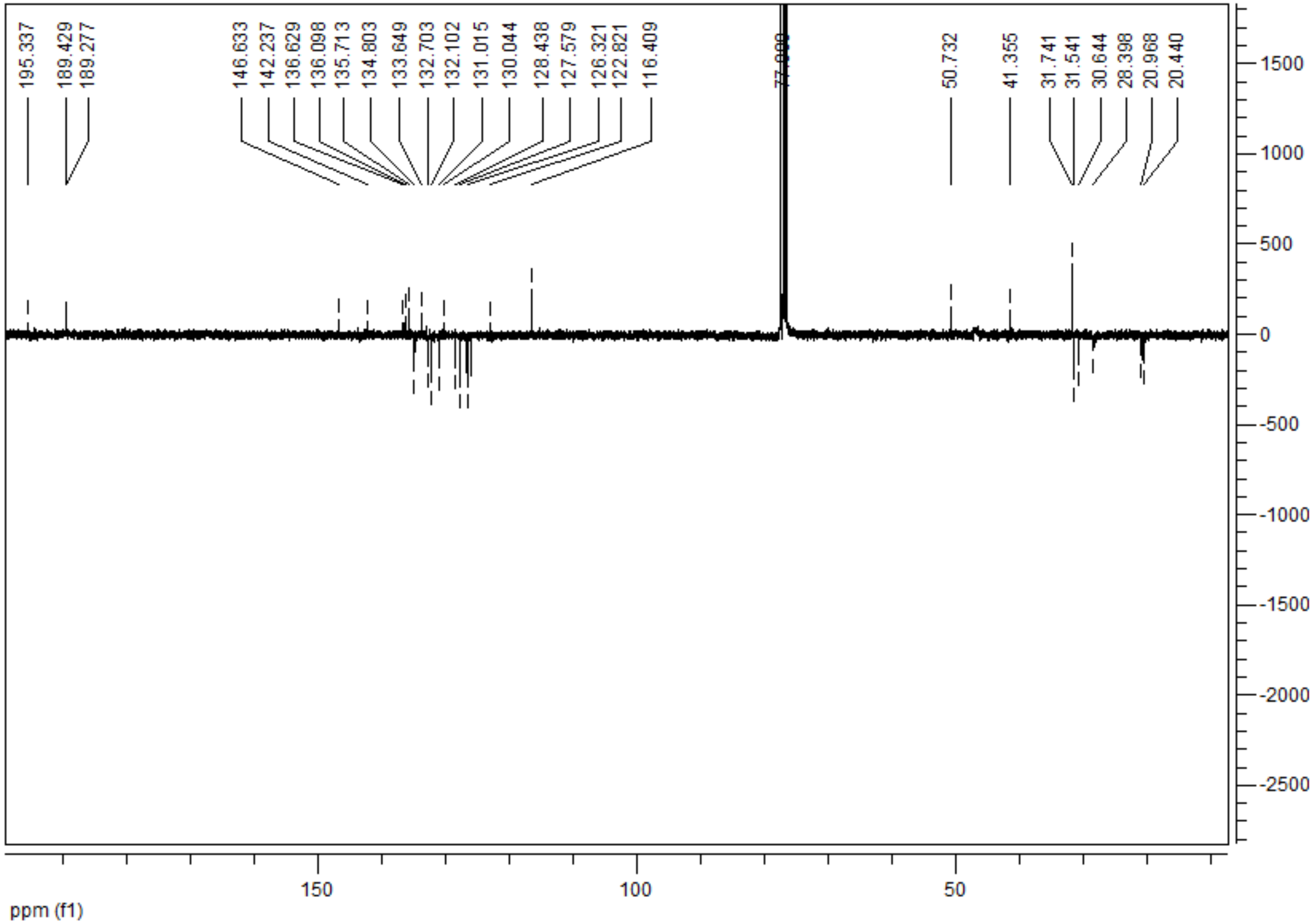

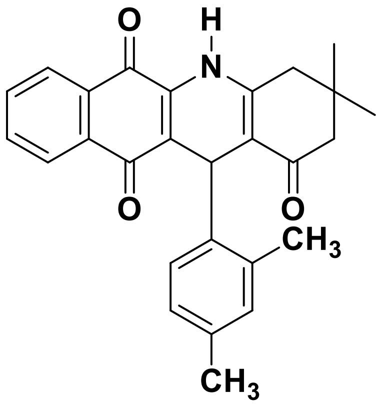

4i

^{13}C NMR spectrum of compound **4i** (75.0 MHz, $CDCl_3$)

+MS, 0.3-0.8min #15-44

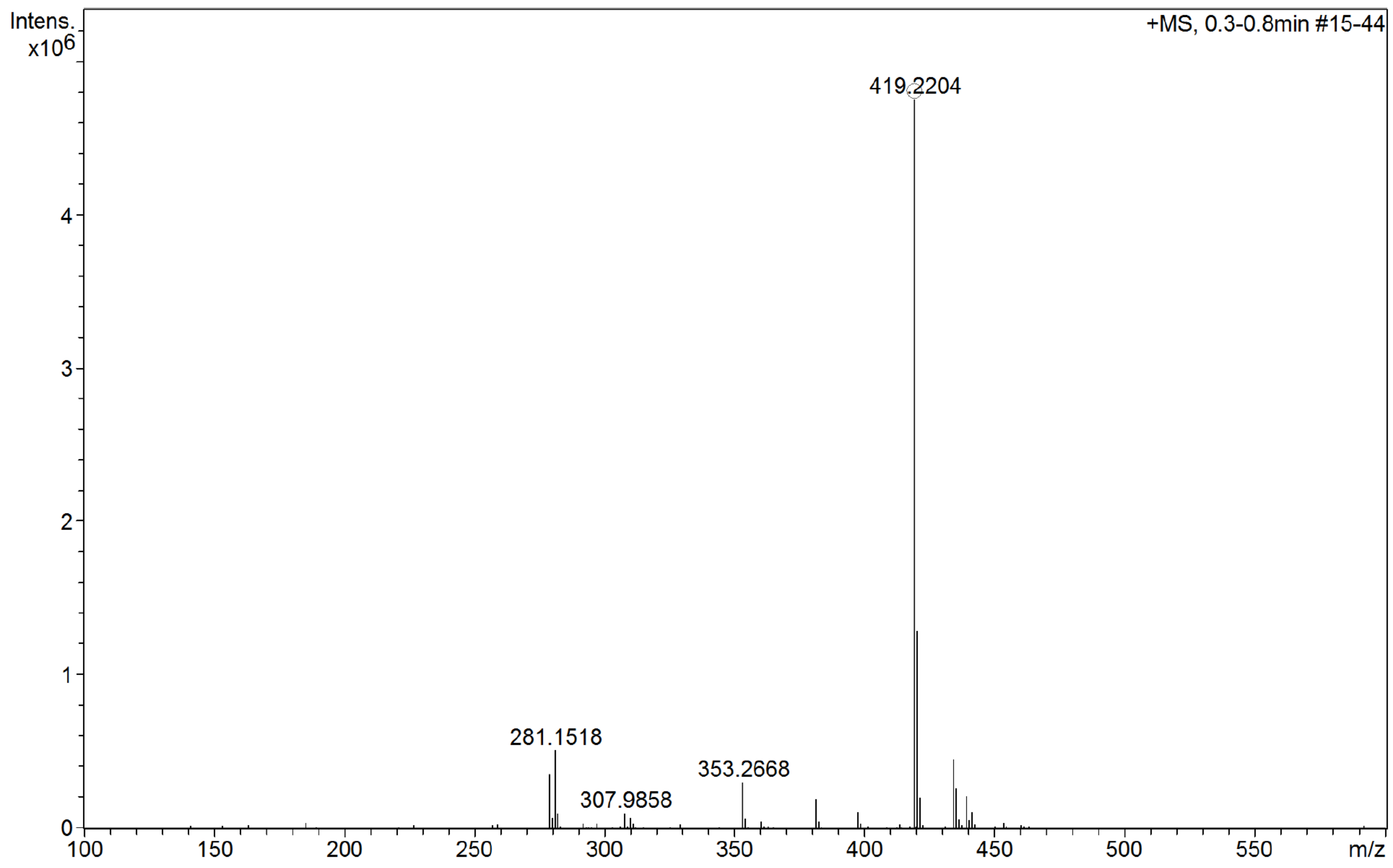

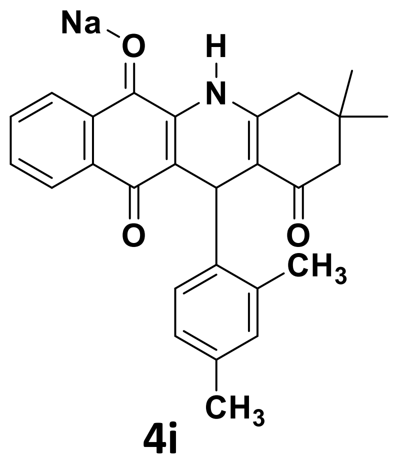

4i

Meas. m/z	#	Ion Formula	m/z	err [ppm]	Mean err [ppm]	rdb	N-Rule	e¯ Conf	mSigma	Std I	Std Mean m/z	Std I VarNorm	Std m/z Diff	Std Comb Dev
419.220360	1	C23H27N6O2	419.219001	-3.2	-4.0	13.5	ok	even	2.0	2.5	n.a.	n.a.	n.a.	n.a.
	2	C27H31O4	419.221686	3.2	3.1	12.5	ok	even	15.7	24.5	n.a.	n.a.	n.a.	n.a.
	1	C25H32NaO4	419.219280	-2.6	-2.6	9.5	ok	even	2.9	4.9	n.a.	n.a.	n.a.	n.a.
	2	C26H28N4Na	419.220618	0.6	0.3	14.5	ok	even	15.9	22.9	n.a.	n.a.	n.a.	n.a.

HRMS of compound **4i**

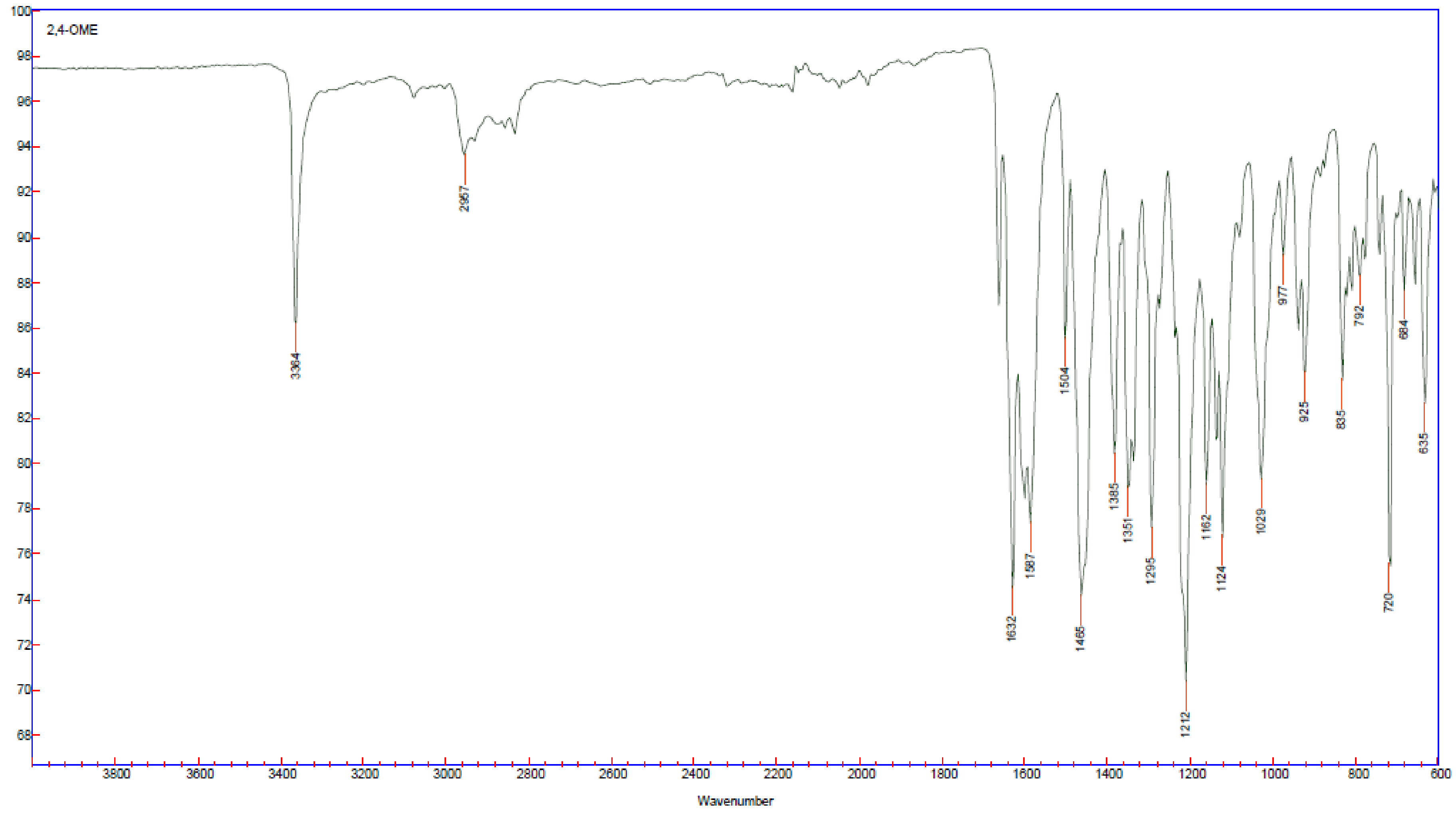

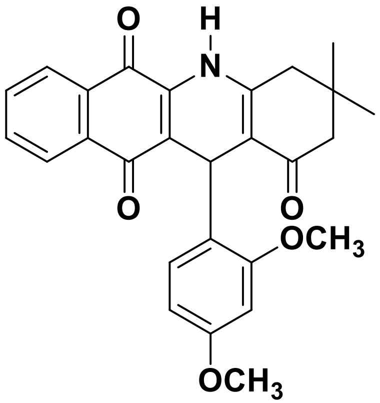

4j

IR spectrum of compound **4j**

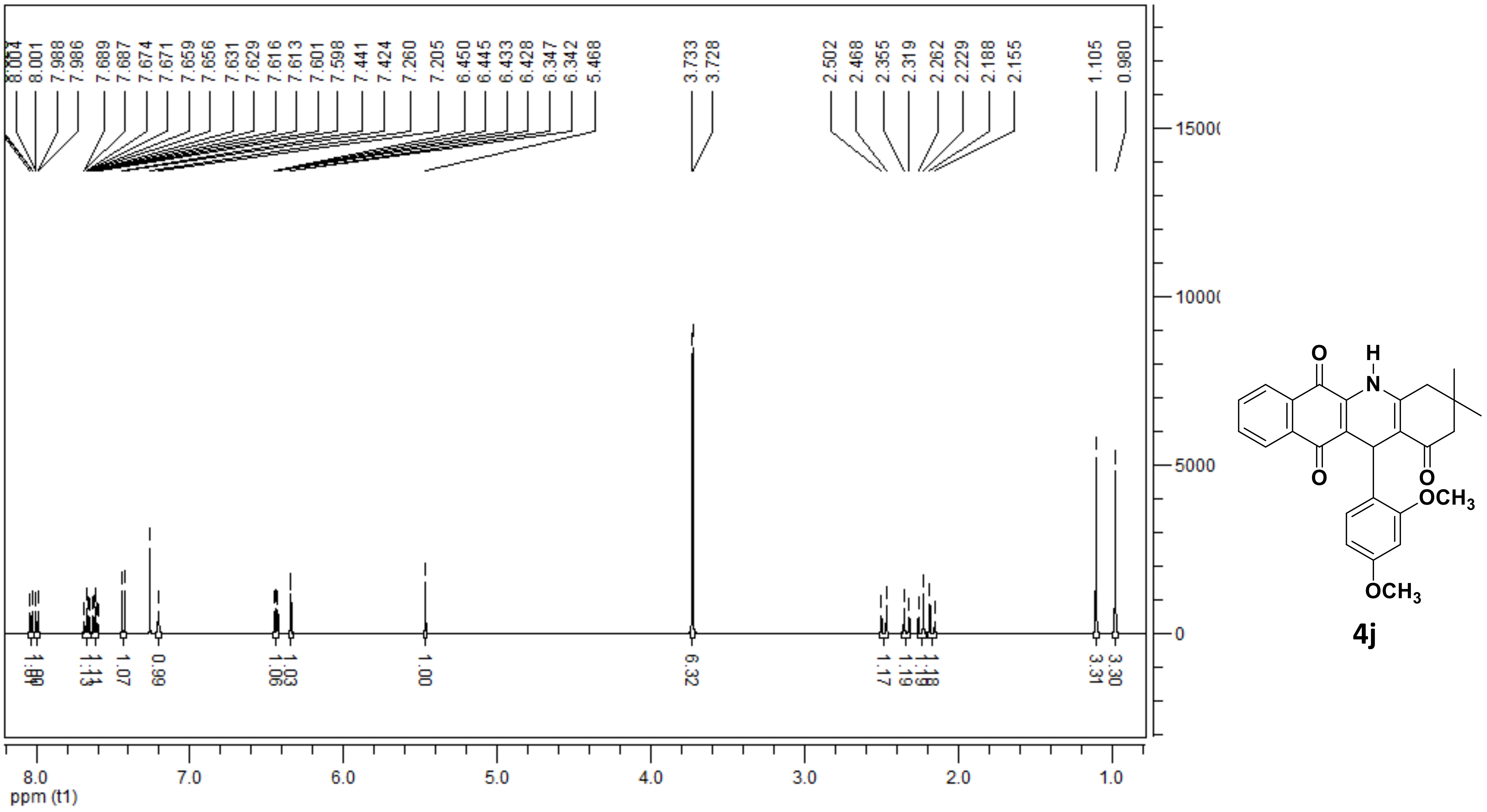

4j

^{1}H NMR spectrum of compound **4j** (500.00 MHz, $CDCl_3$)

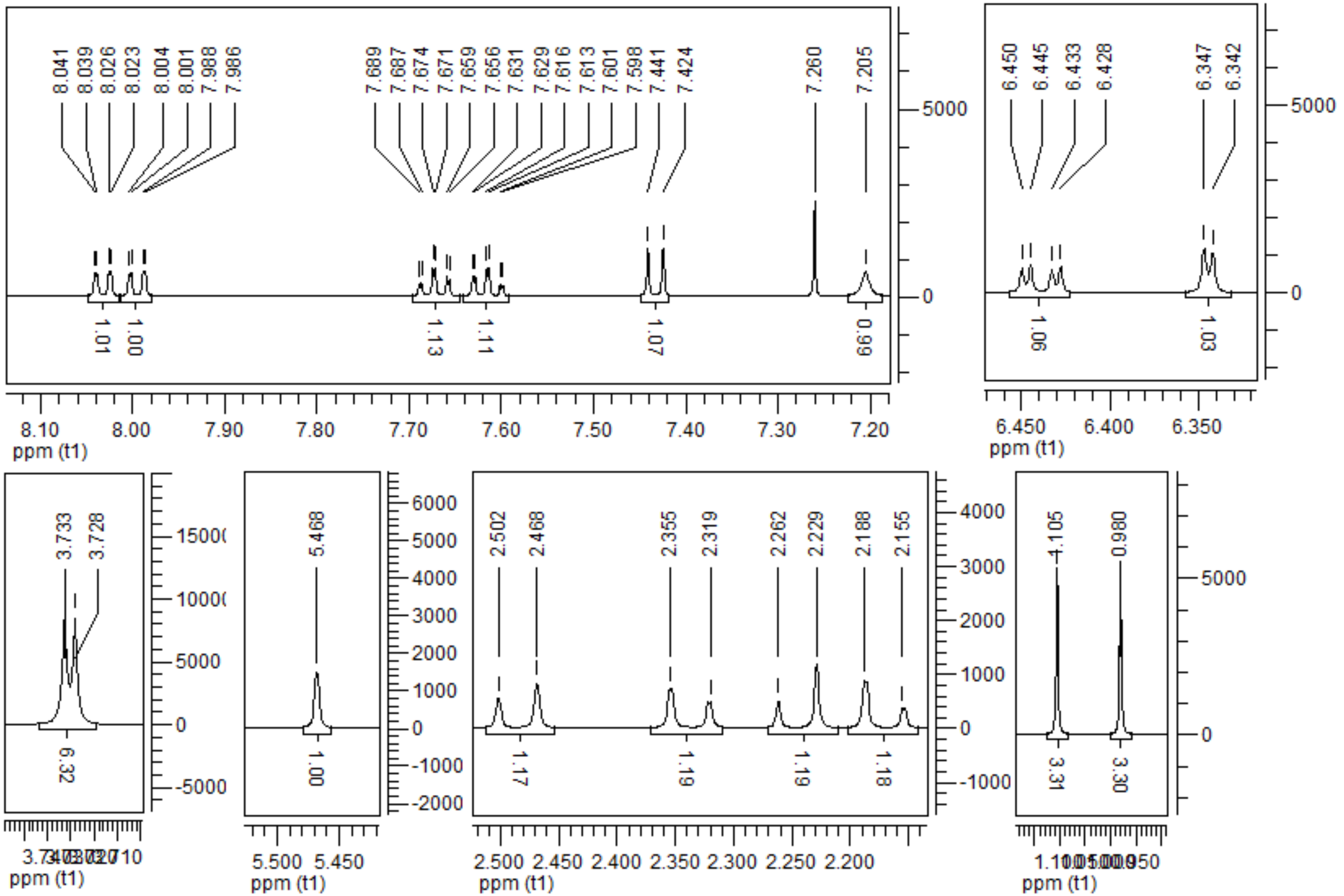

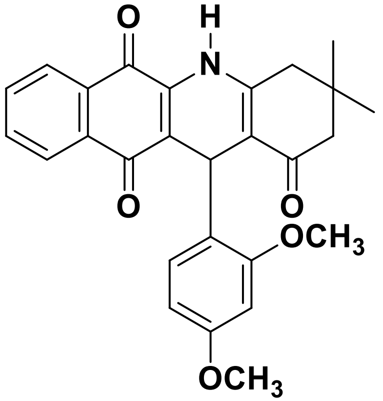

4j

^{1}H NMR spectrum of compound **4j** (500.00 MHz, $CDCl_3$)

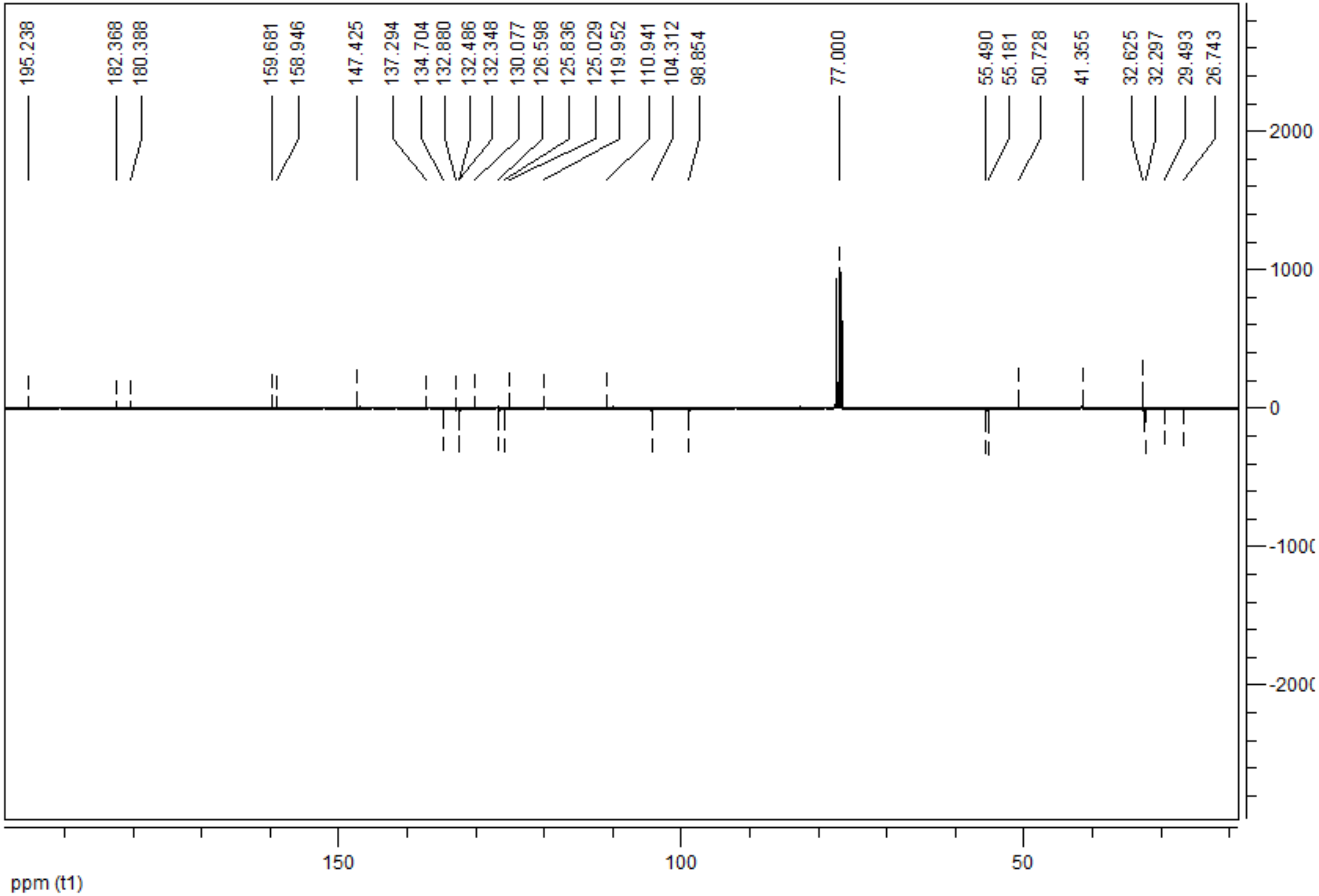

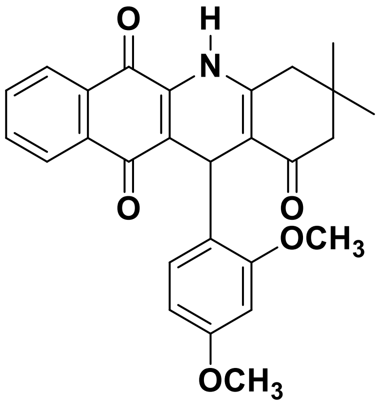

4j

^{13}C NMR spectrum of compound **4j** (75.0 MHz, $CDCl_3$)

+MS, 0.2-0.6min #10-36

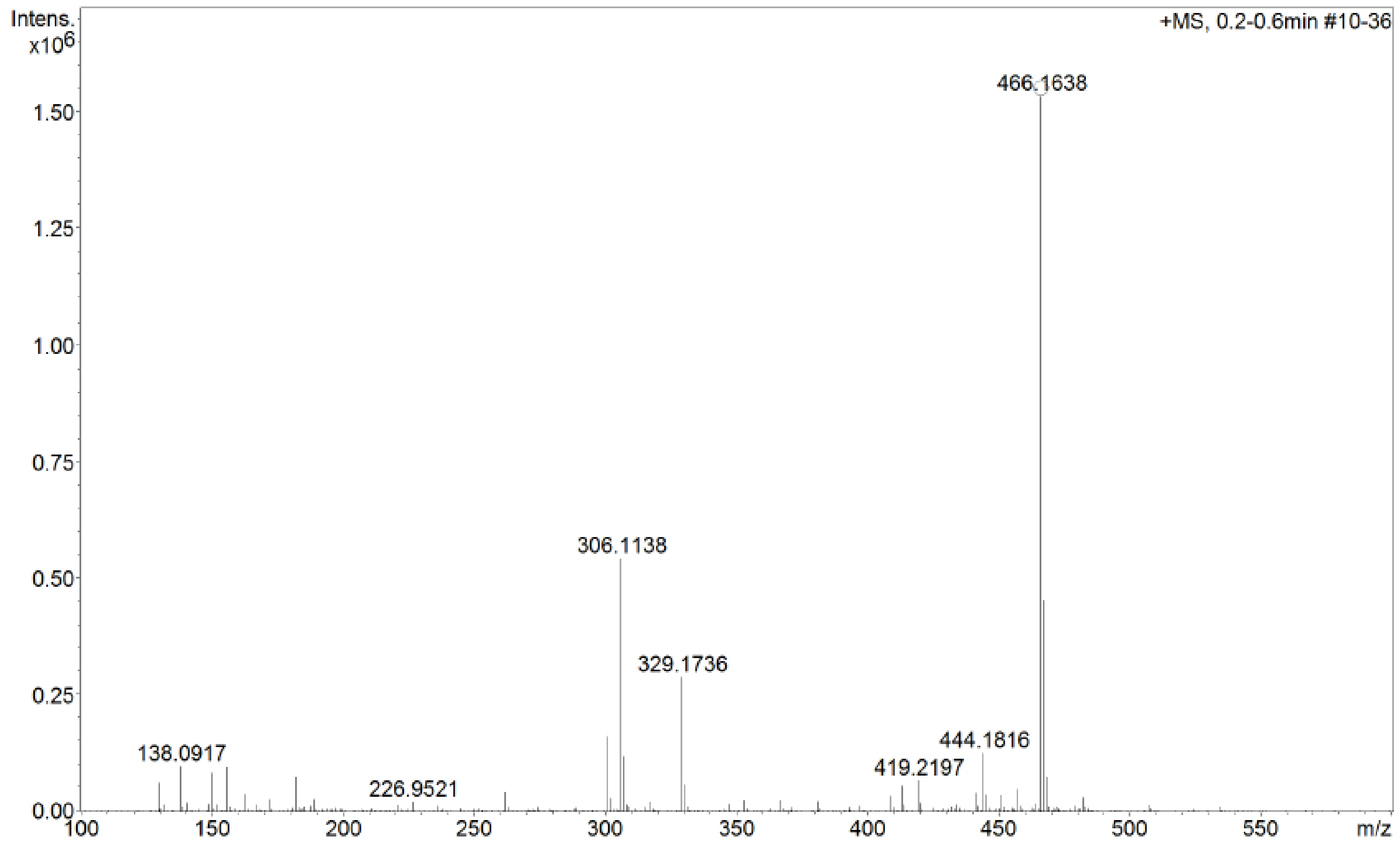

Meas. m/z	#	Ion Formula	m/z	err [ppm]	Mean err [ppm]	rdb	N-Rule	e⁻ Conf	mSigma	Std I	Std Mean m/z	Std I VarNorm	Std m/z Diff	Std Comb Dev
466.163781	1	C27H25NNaO5	466.162494	-2.8	-2.4	15.5	ok	even	3.4	6.8	n.a.	n.a.	n.a.	n.a.
	2	C28H21N5NaO	466.163831	0.1	-0.2	20.5	ok	even	15.8	22.6	n.a.	n.a.	n.a.	n.a.

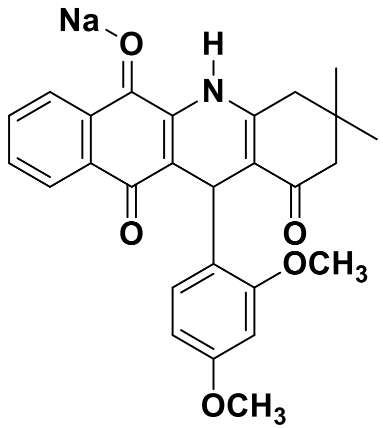

Exact Mass: 466,1630

4j

HRMS of compound **4j**

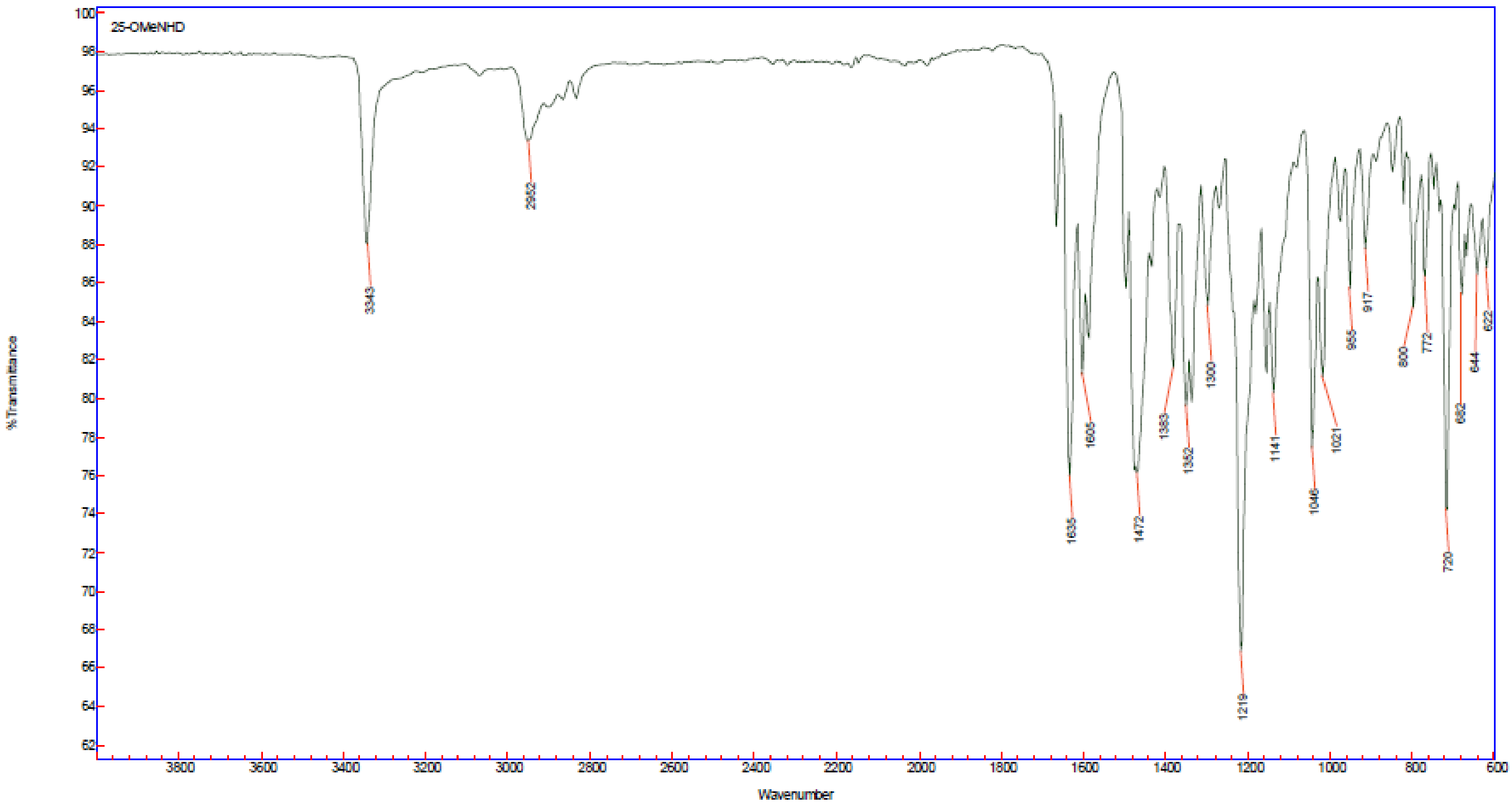

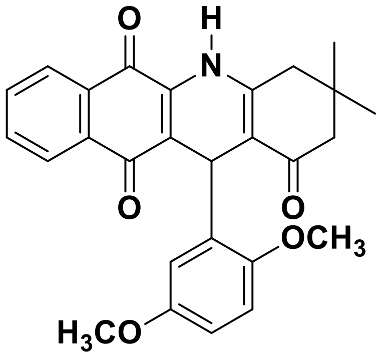

4k

IR spectrum of compound **4k**

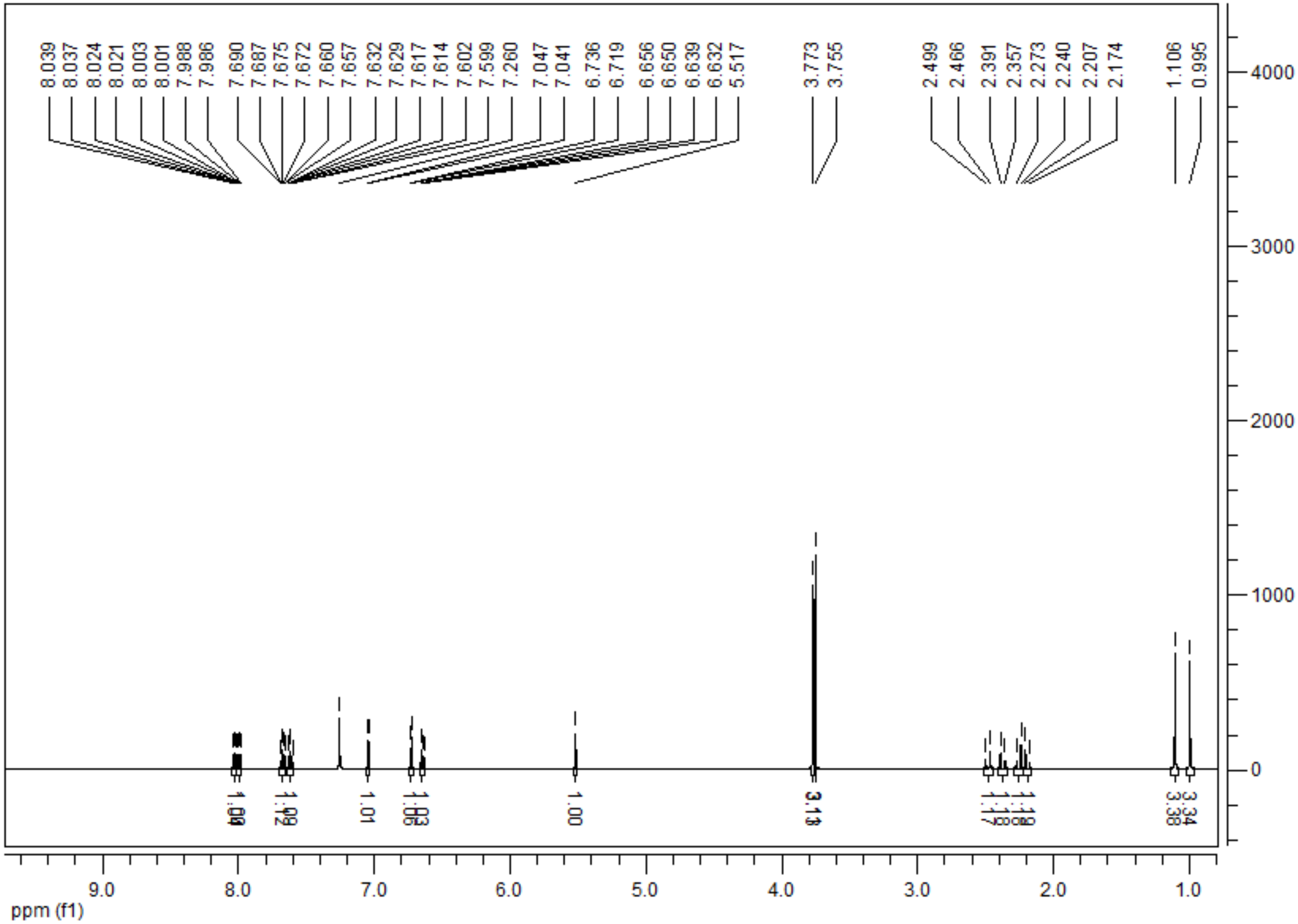

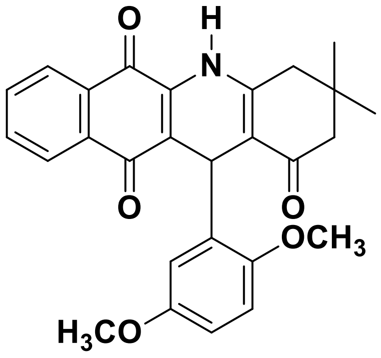

4k

^{1}H NMR spectrum of compound **4k** (500.00 MHz, $CDCl_3$)

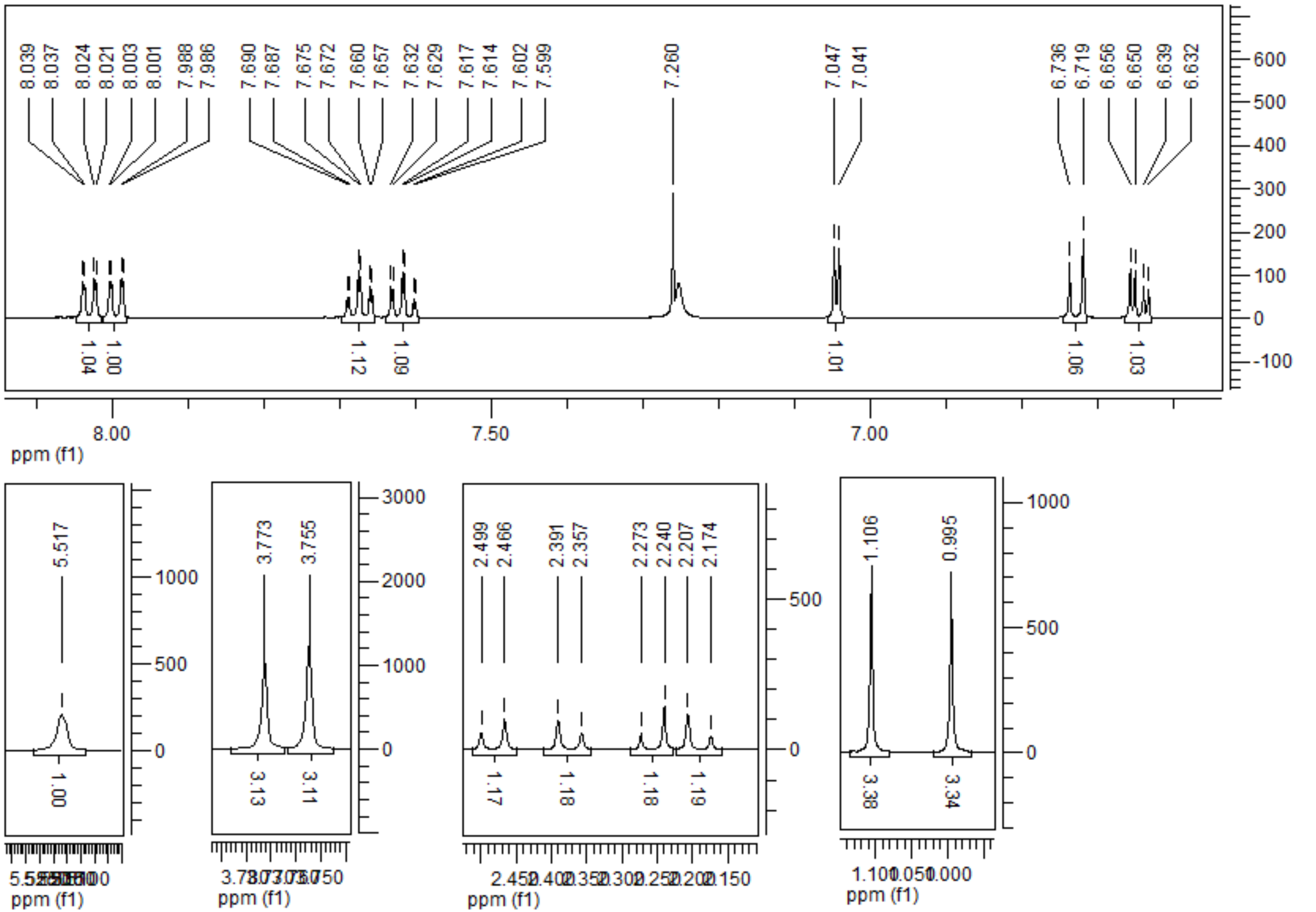

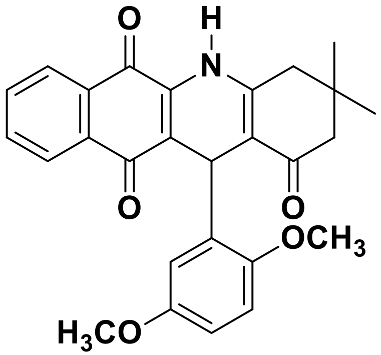

4k

^{1}H NMR spectrum of compound **4k** (500.00 MHz, $CDCl_3$)

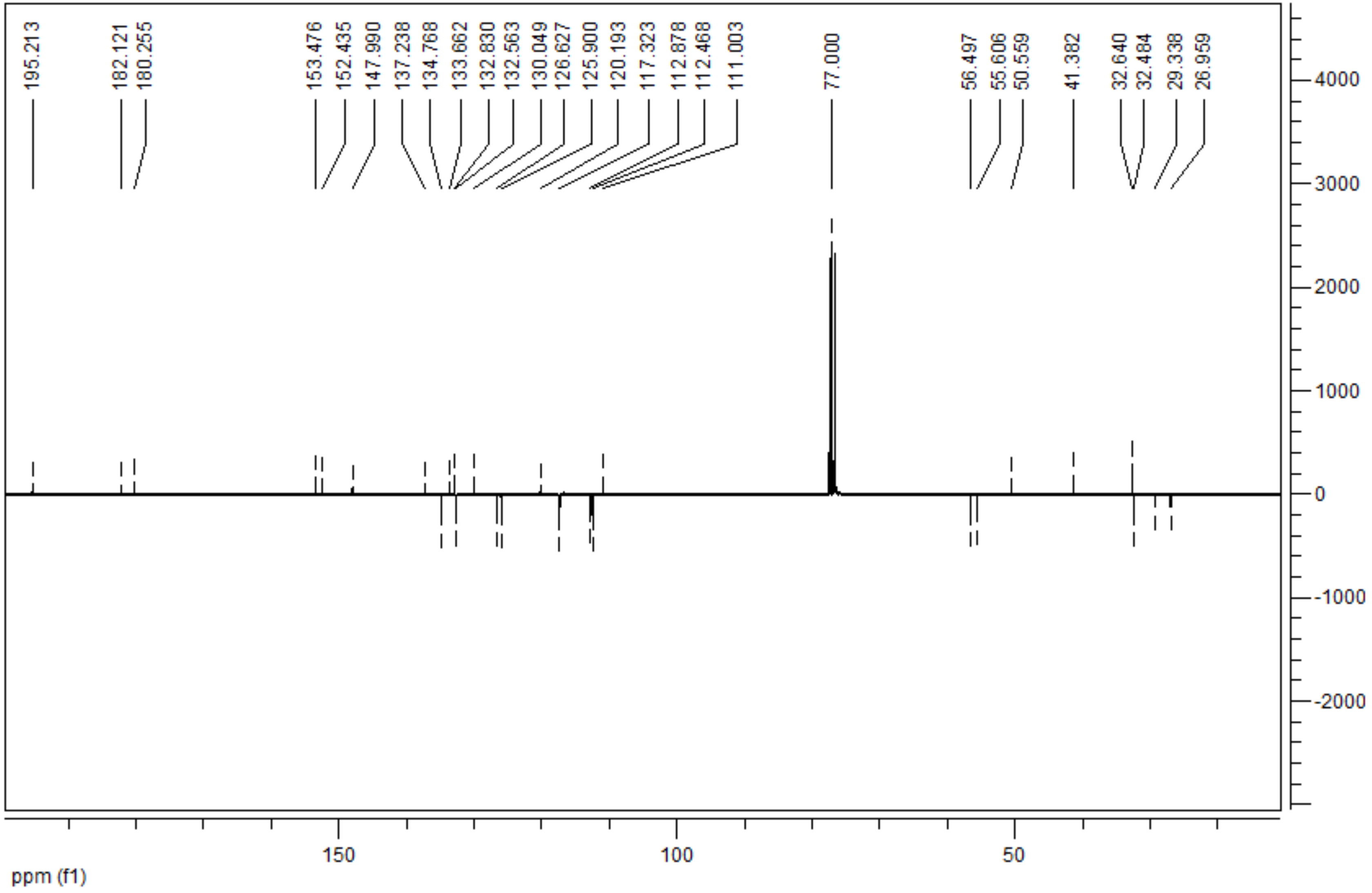

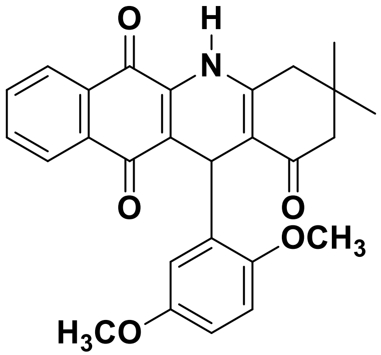

4k

^{13}C NMR spectrum of compound **4k** (75.0 MHz, $CDCl_3$)

+MS, 0.1-0.6min #3-34

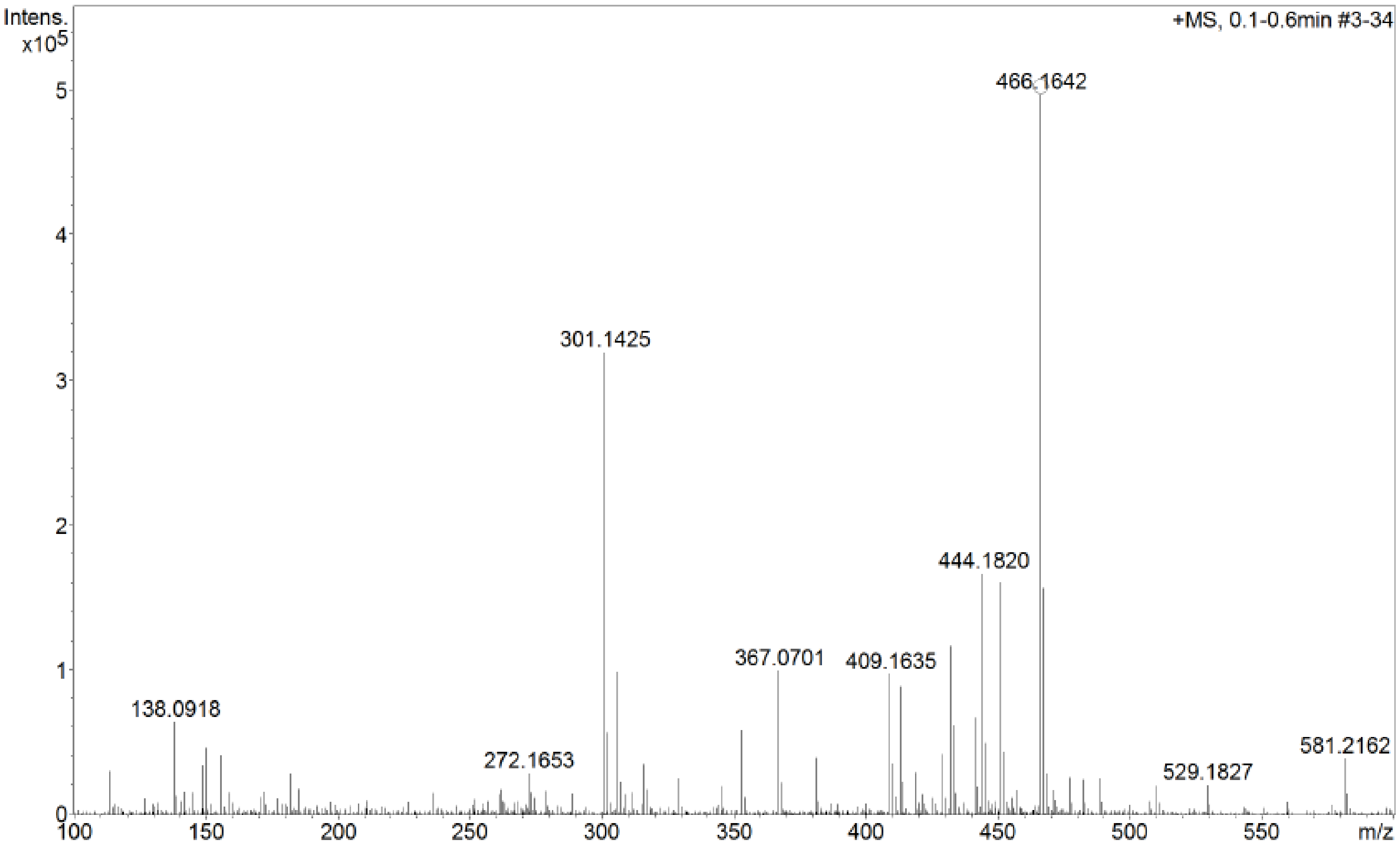

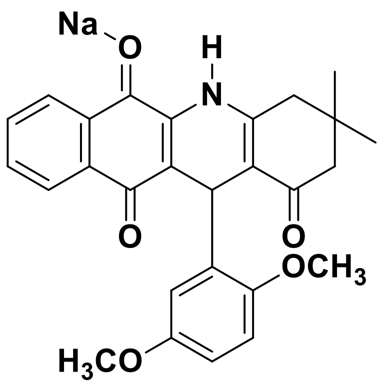

Exact Mass: 466,1630

4k

Meas. m/z	#	Ion Formula	m/z	err [ppm]	Mean err [ppm]	rdb	N-Rule	e⁻ Conf	mSigma	Std I	Std Mean m/z	Std I VarNorm	Std m/z Diff	Std Comb Dev
466.164222	1	C28H21N5NaO	466.163831	-0.8	-0.6	20.5	ok	even	4.9	6.0	n.a.	n.a.	n.a.	n.a.
	2	C27H25NNaO5	466.162494	-3.7	-4.4	15.5	ok	even	8.4	15.0	n.a.	n.a.	n.a.	n.a.

HRMS of compound **4k**

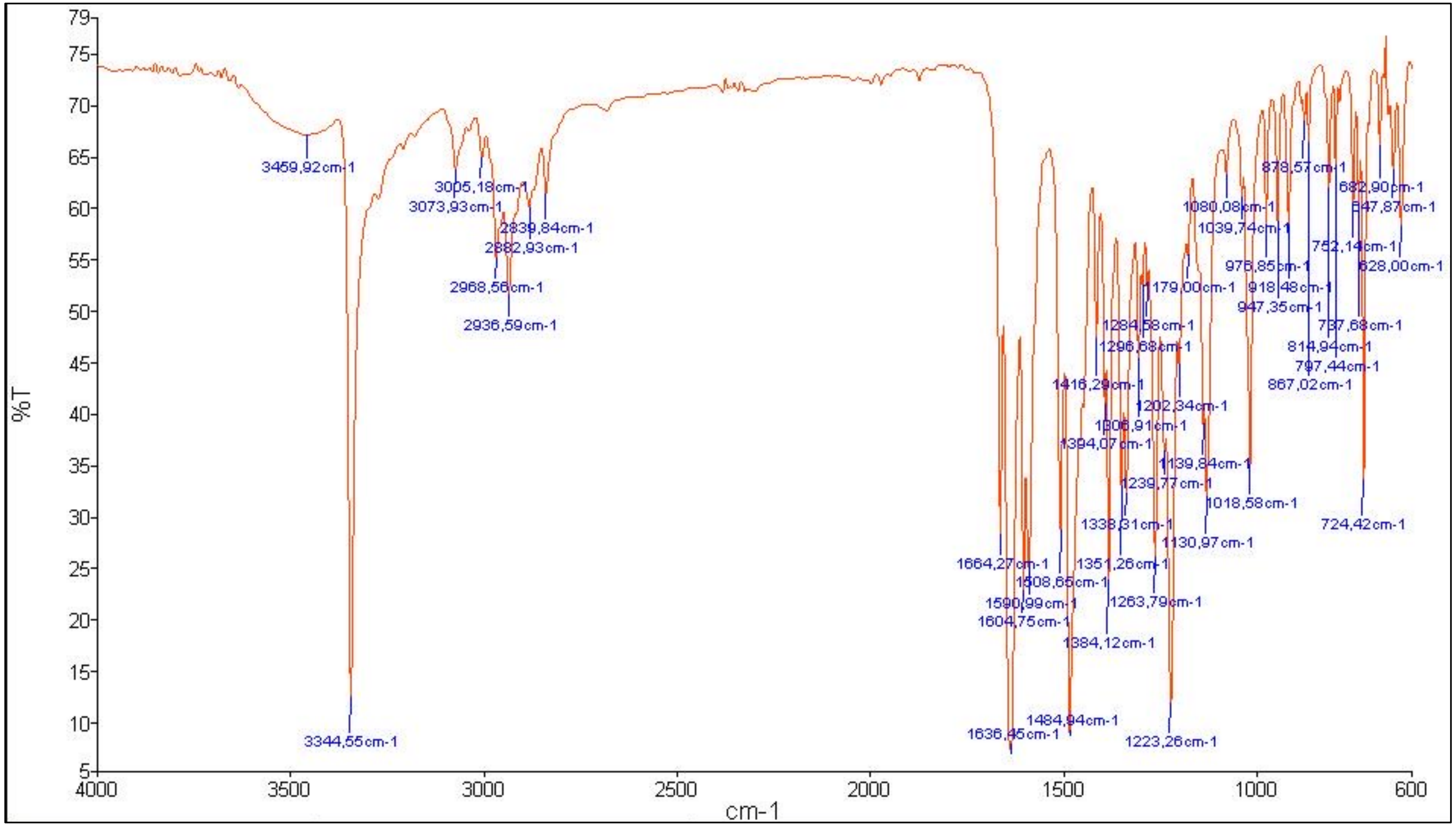

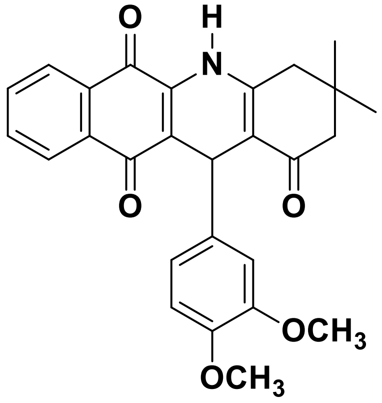

4l

IR spectrum of compound **4l**

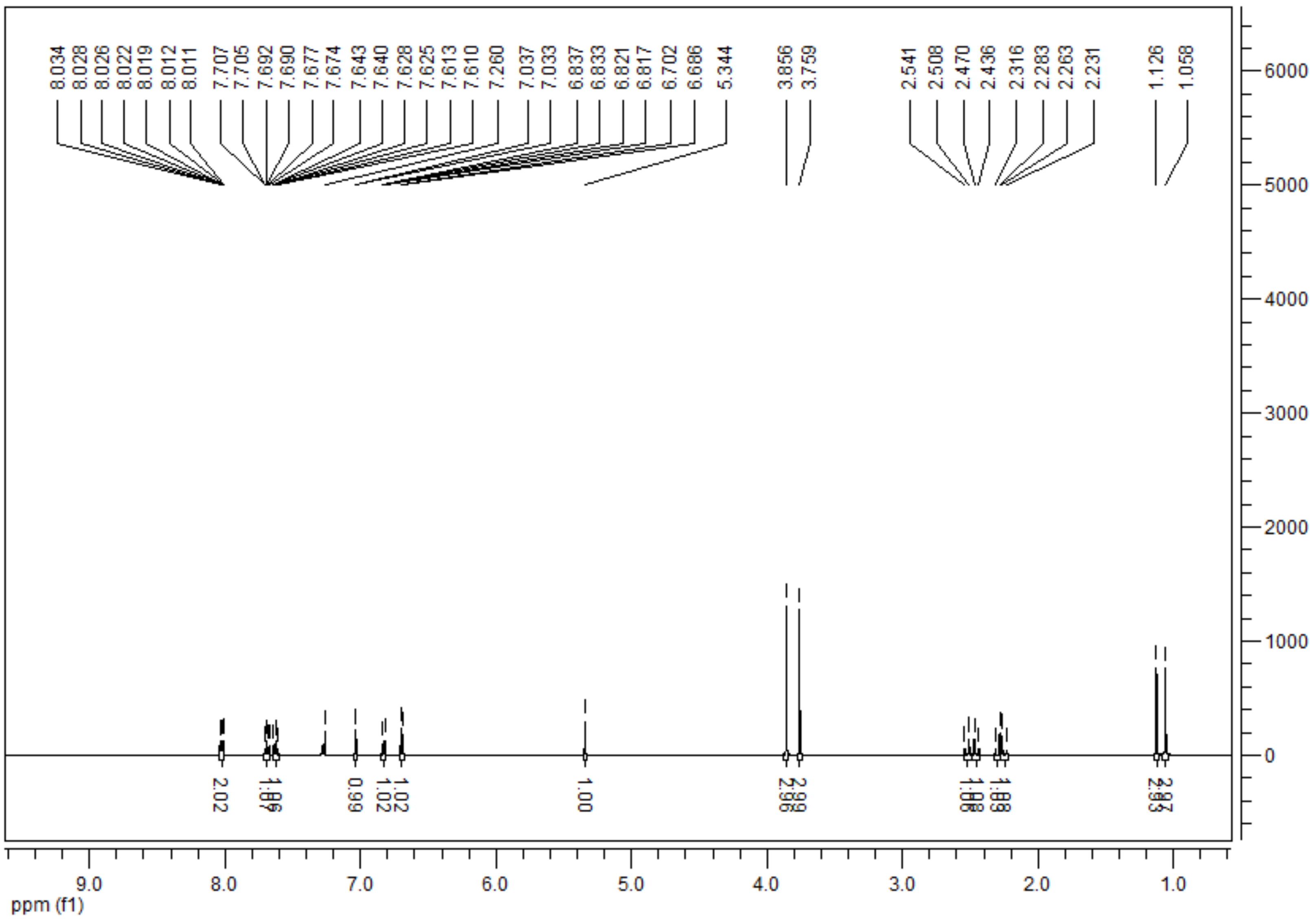

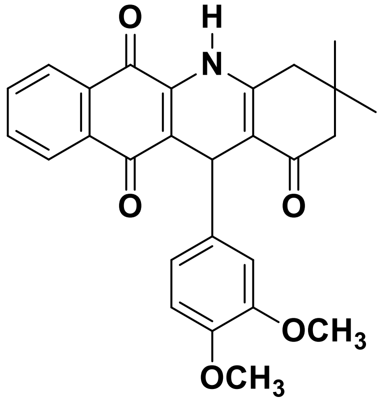

4l

^{1}H NMR spectrum of compound **4l** (500.00 MHz, $CDCl_3$)

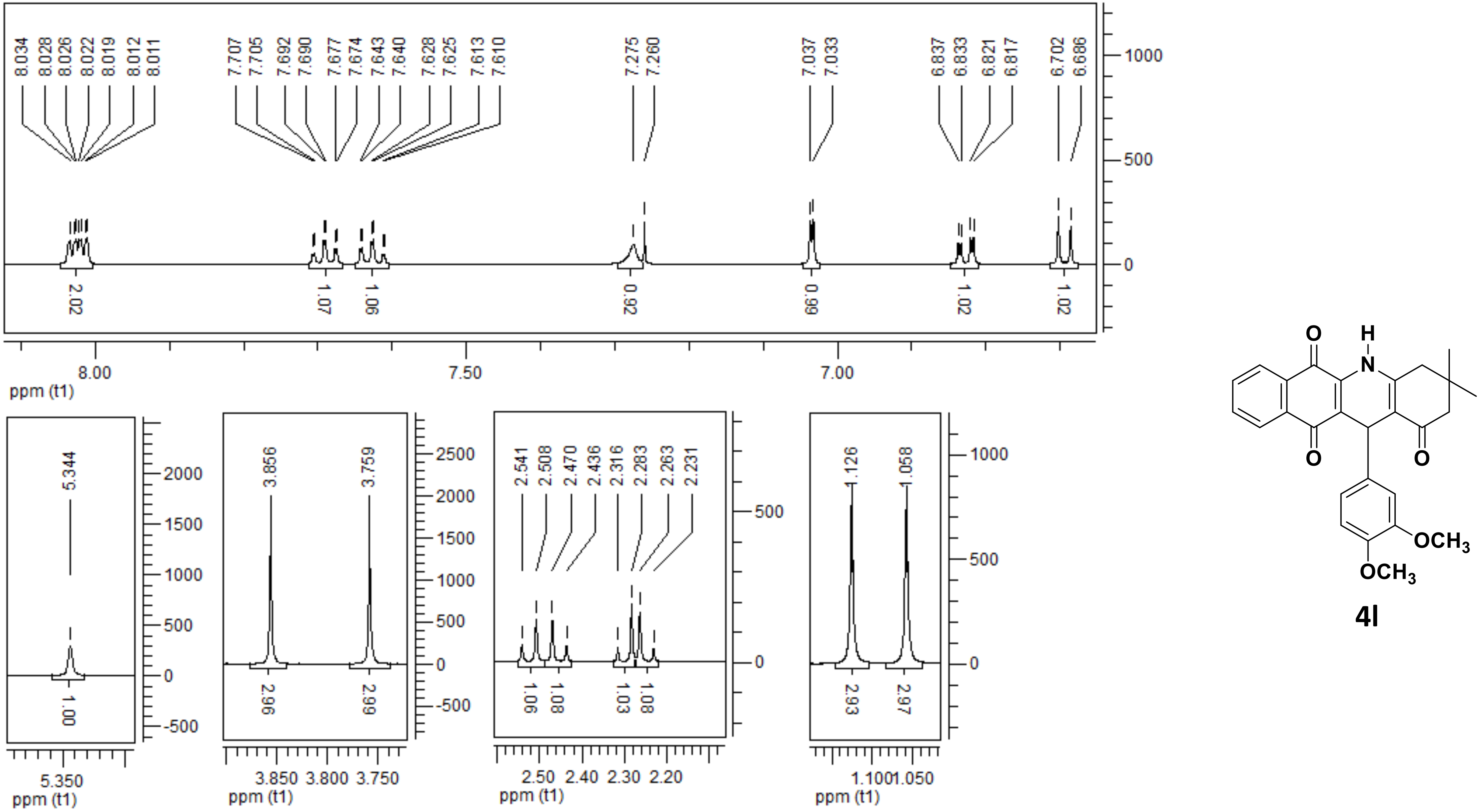

4l

^{1}H NMR spectrum of compound **4l** (500.00 MHz, $CDCl_3$)

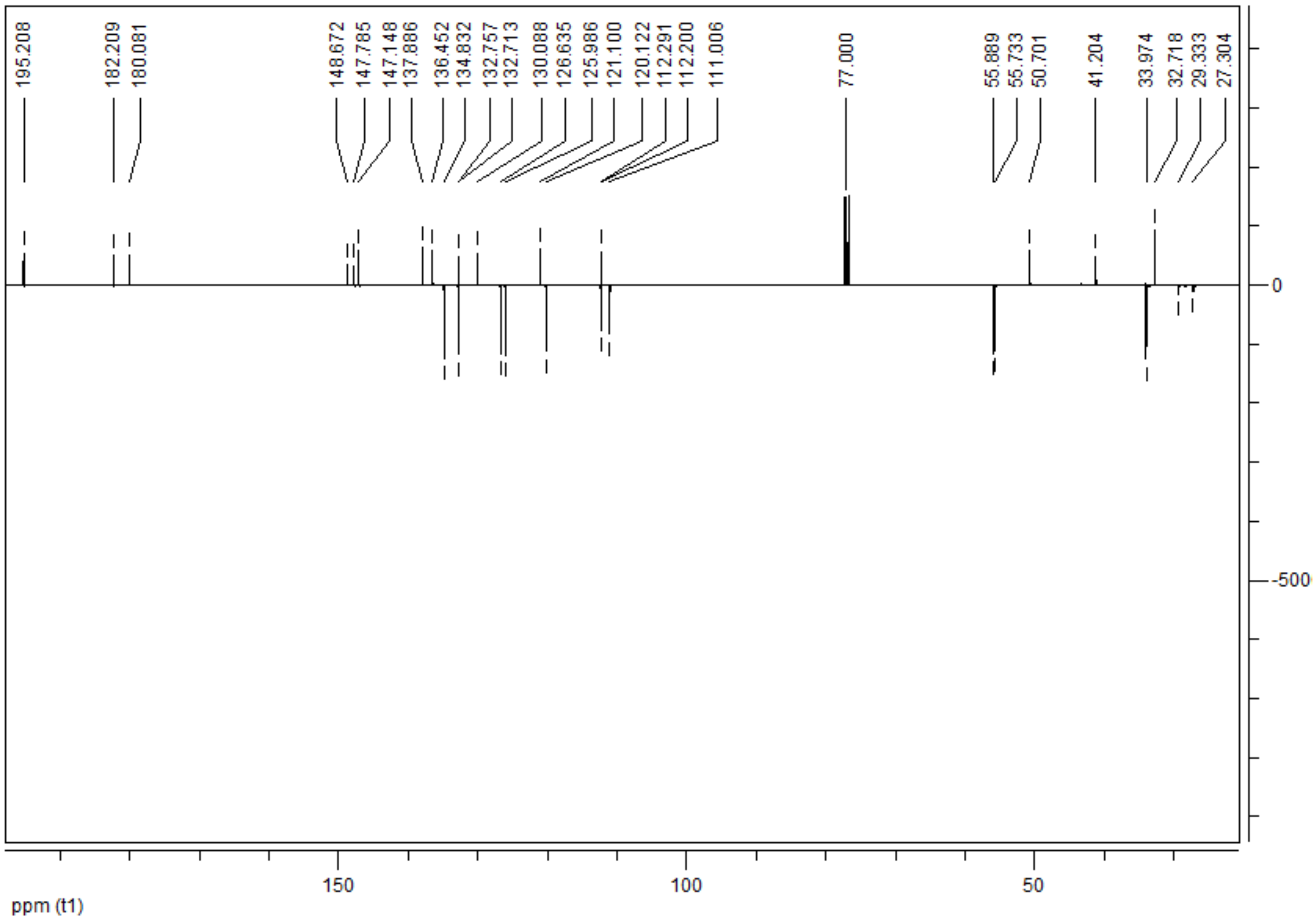

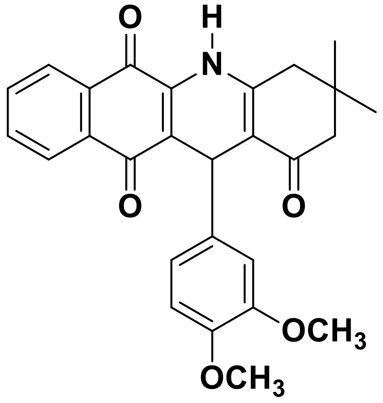

4l

^{13}C NMR spectrum of compound **4l** (125.0 MHz, $CDCl_3$)

+MS, 0.2-0.8min #13-45

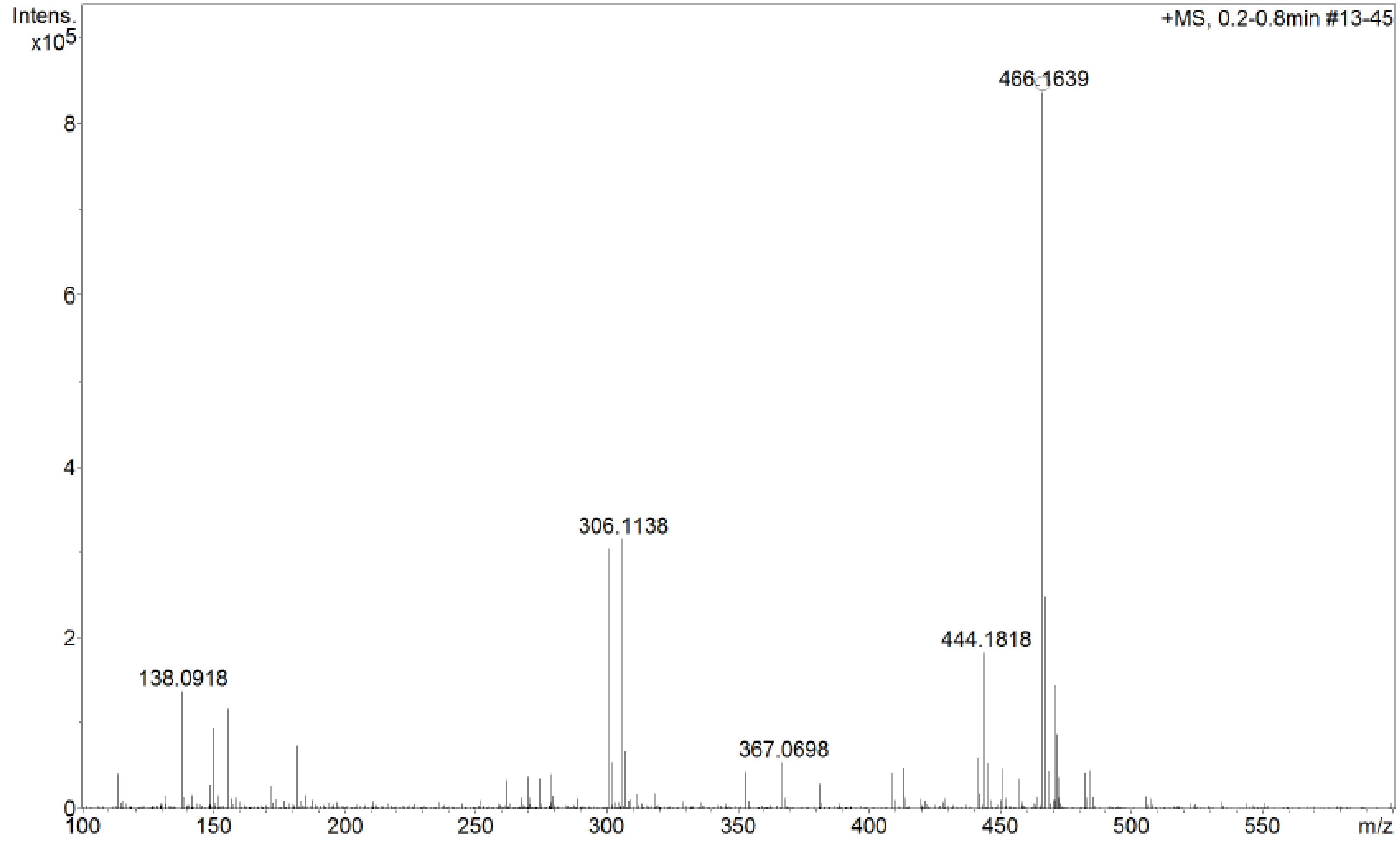

Meas. m/z	#	Ion Formula	m/z	err [ppm]	Mean err [ppm]	rdb	N-Rule	e¯ Conf	mSigma	Std I	Std Mean m/z	Std I VarNorm	Std m/z Diff	Std Comb Dev
466.163935	1	C27H25NNaO5	466.162494	-3.1	-4.5	15.5	ok	even	1.8	3.0	n.a.	n.a.	n.a.	n.a.
	2	C28H21N5NaO	466.163831	-0.2	-0.9	20.5	ok	even	15.3	20.9	n.a.	n.a.	n.a.	n.a.

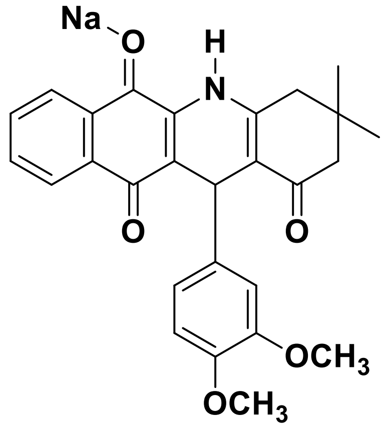

Exact Mass: 466,1630

4l

HRMS of compound **4l**

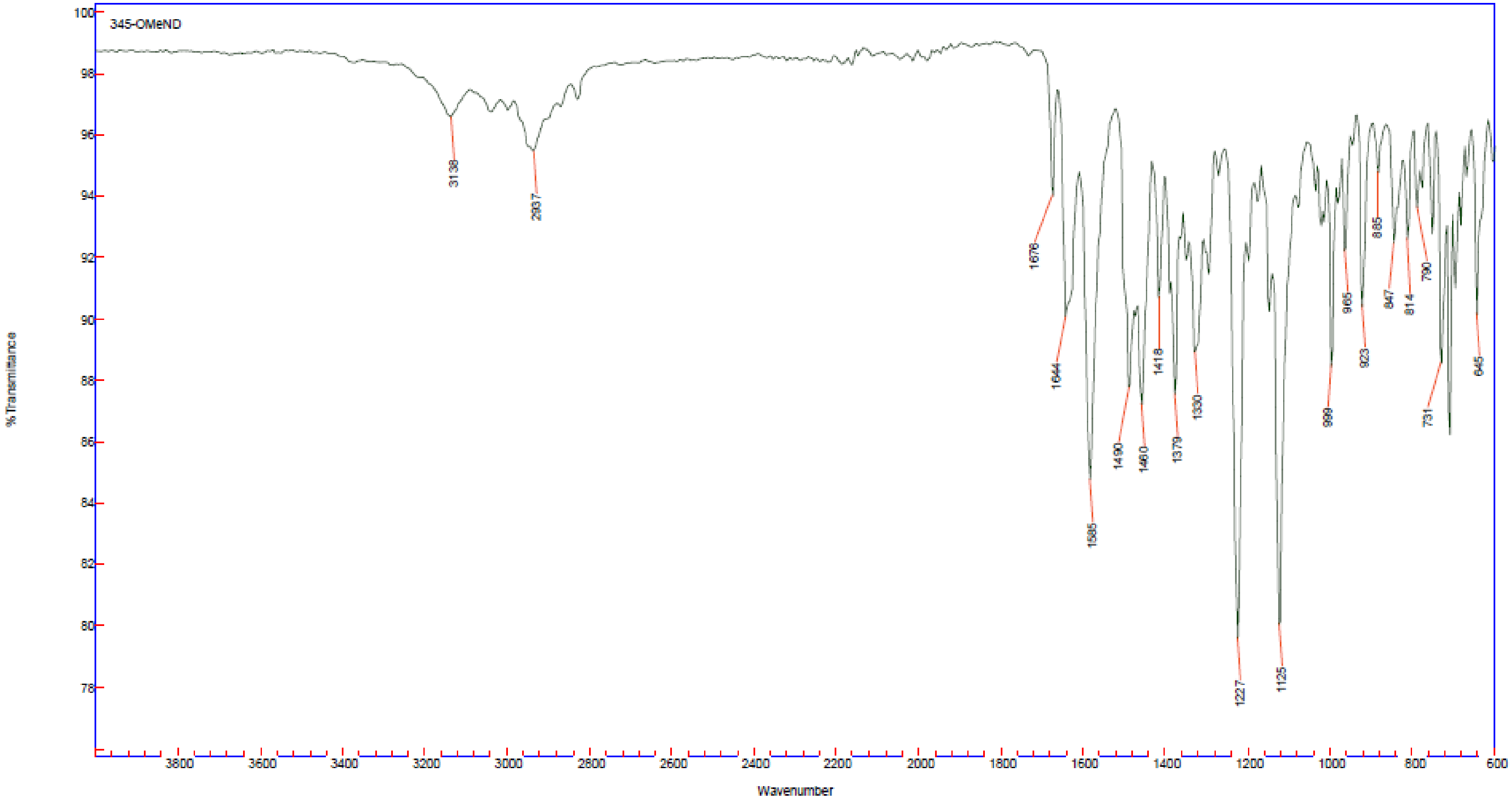

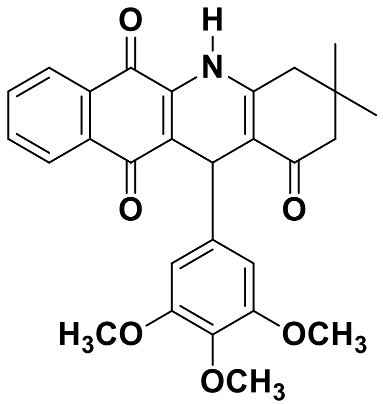

4m

IR spectrum of compound **4m**

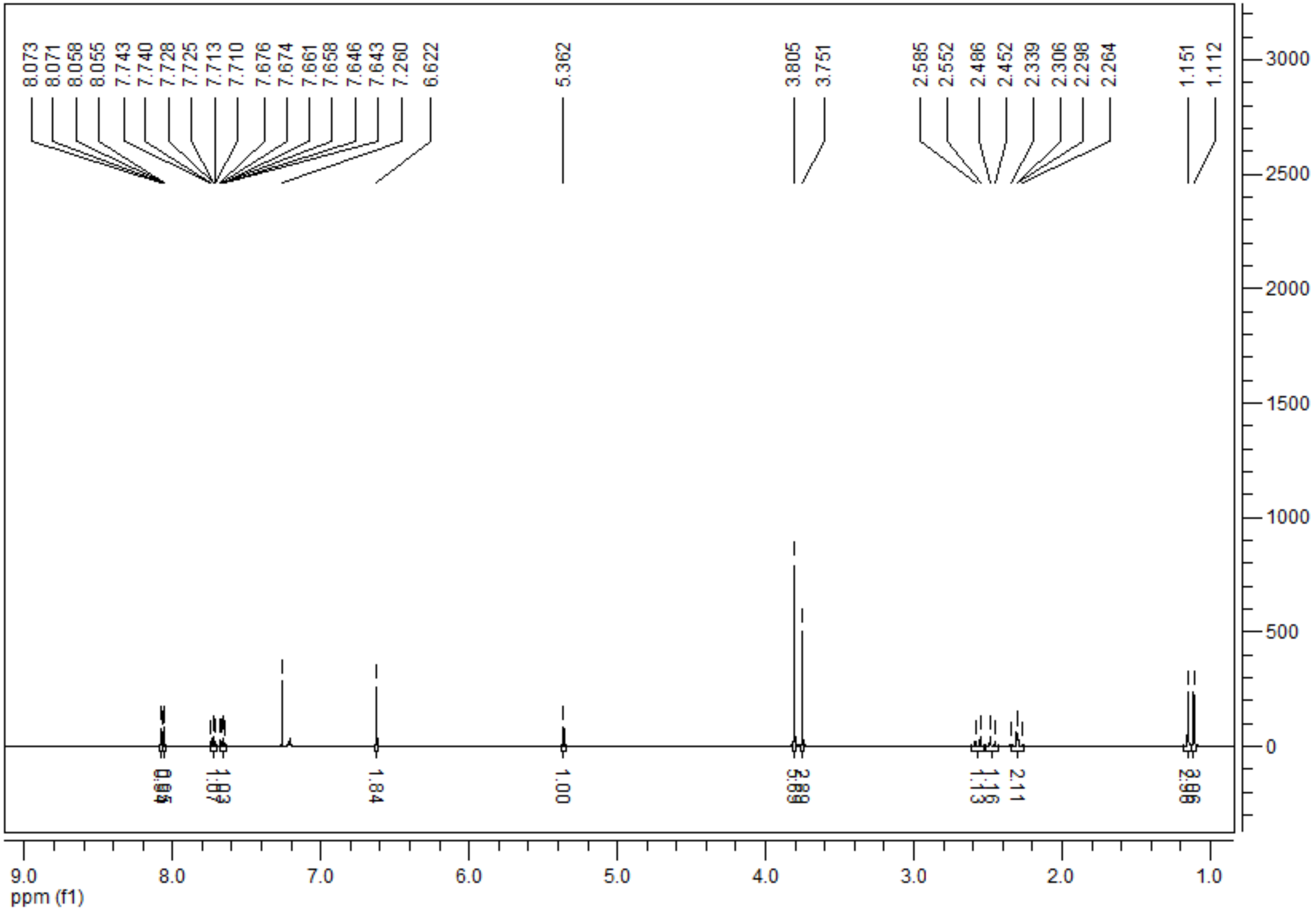

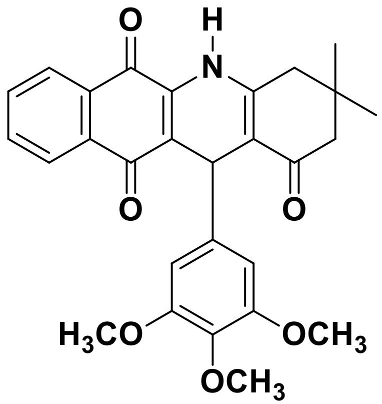

4m

^{1}H NMR spectrum of compound **4m** (500.00 MHz, $CDCl_3$)

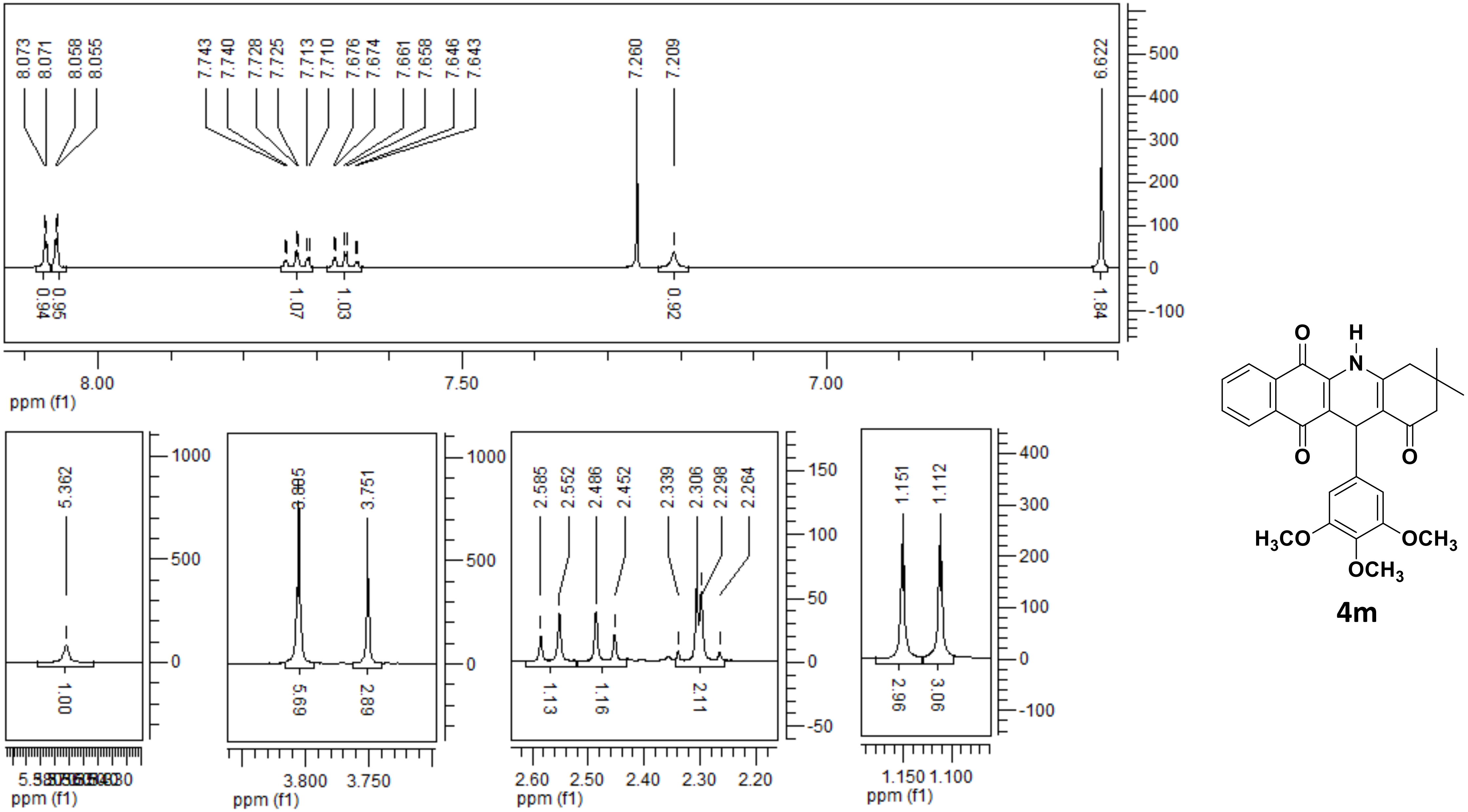

^{1}H NMR spectrum of compound **4m** (500.00 MHz, $CDCl_3$)

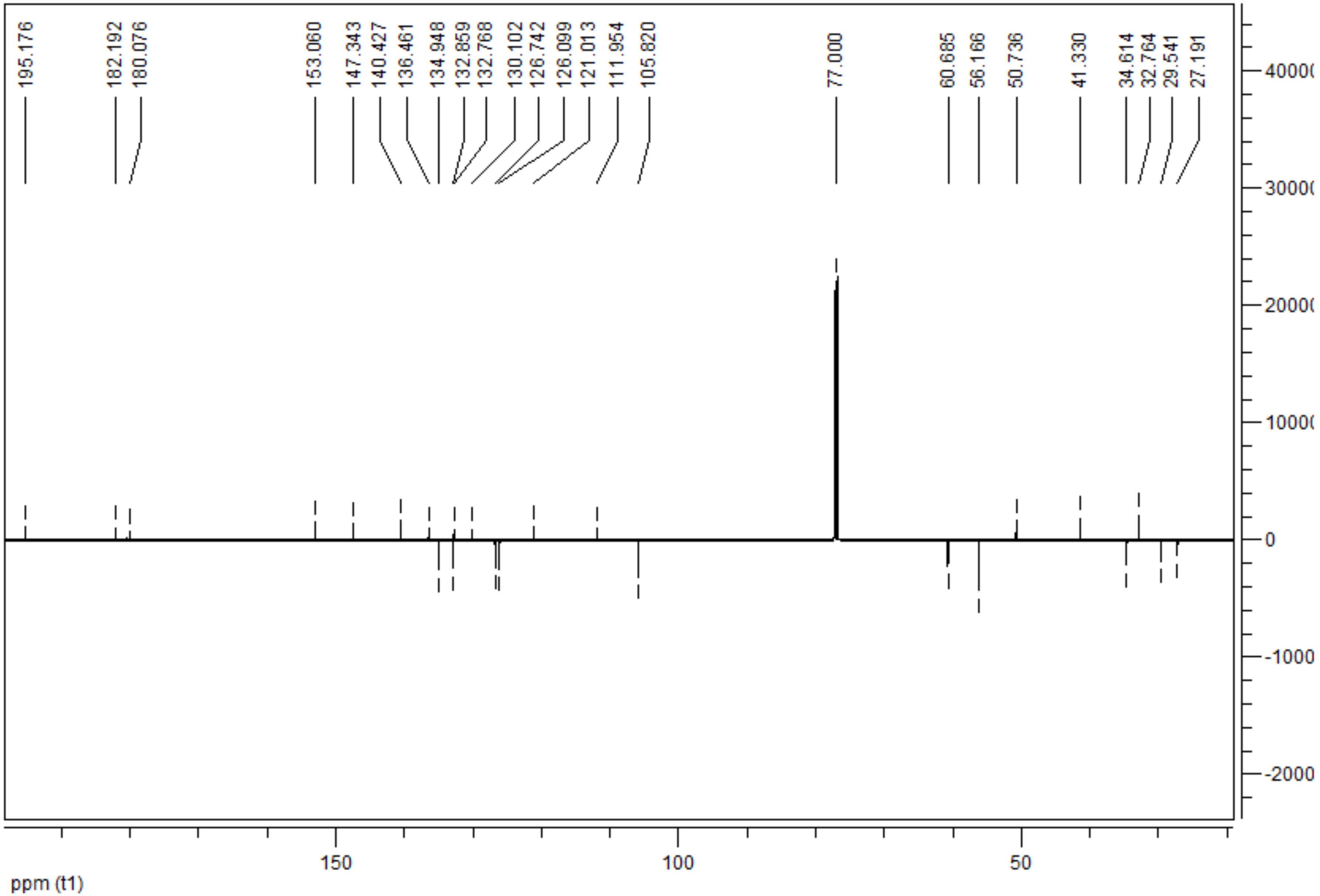

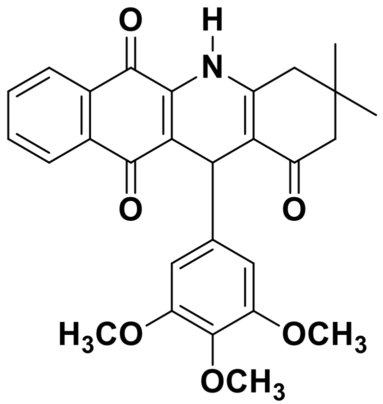

4m

^{13}C NMR spectrum of compound **4m** (125.0 MHz, $CDCl_3$)

+MS, 0.2-0.8min #14-45

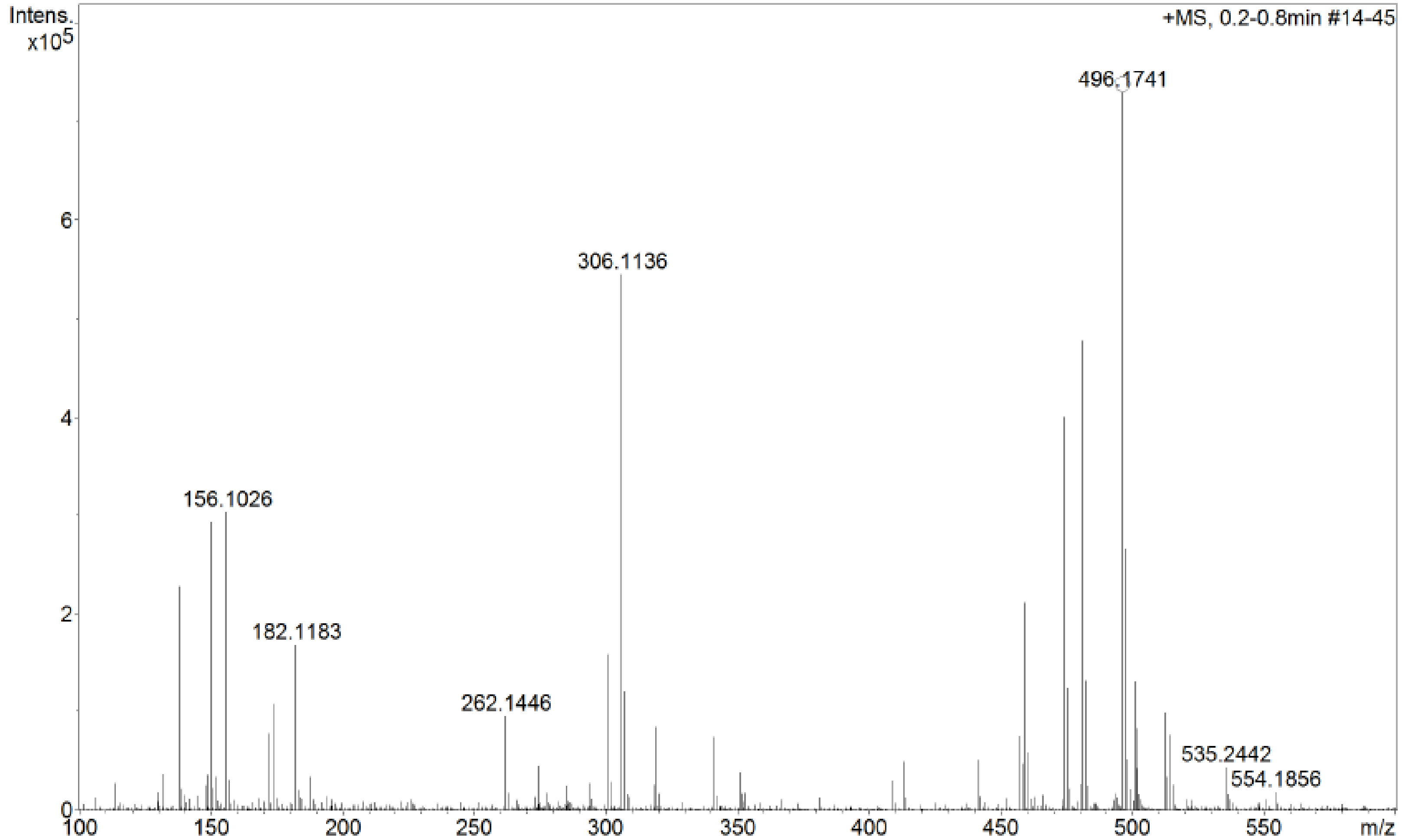

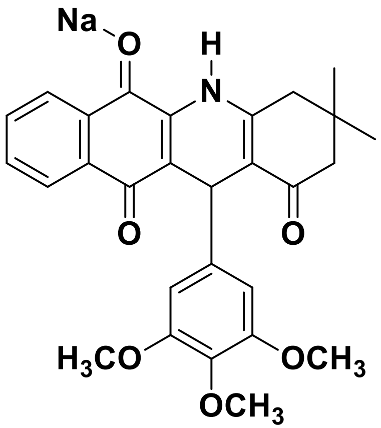

Exact Mass: 496,1736

4m

Meas. m/z	#	Ion Formula	m/z	err [ppm]	Mean err [ppm]	rdb	N-Rule	e⁻ Conf	mSigma	Std I	Std Mean m/z	Std I VarNorm	Std m/z Diff	Std Comb Dev
496.174090	1	C29H23N5NaO2	496.174396	0.6	982.8	20.5	ok	even	16.8	25.1	n.a.	n.a.	n.a.	n.a.
	2	C28H27NNaO6	496.173058	-2.1	986.9	15.5	ok	even	27.9	41.5	n.a.	n.a.	n.a.	n.a.

HRMS of compound **4m**

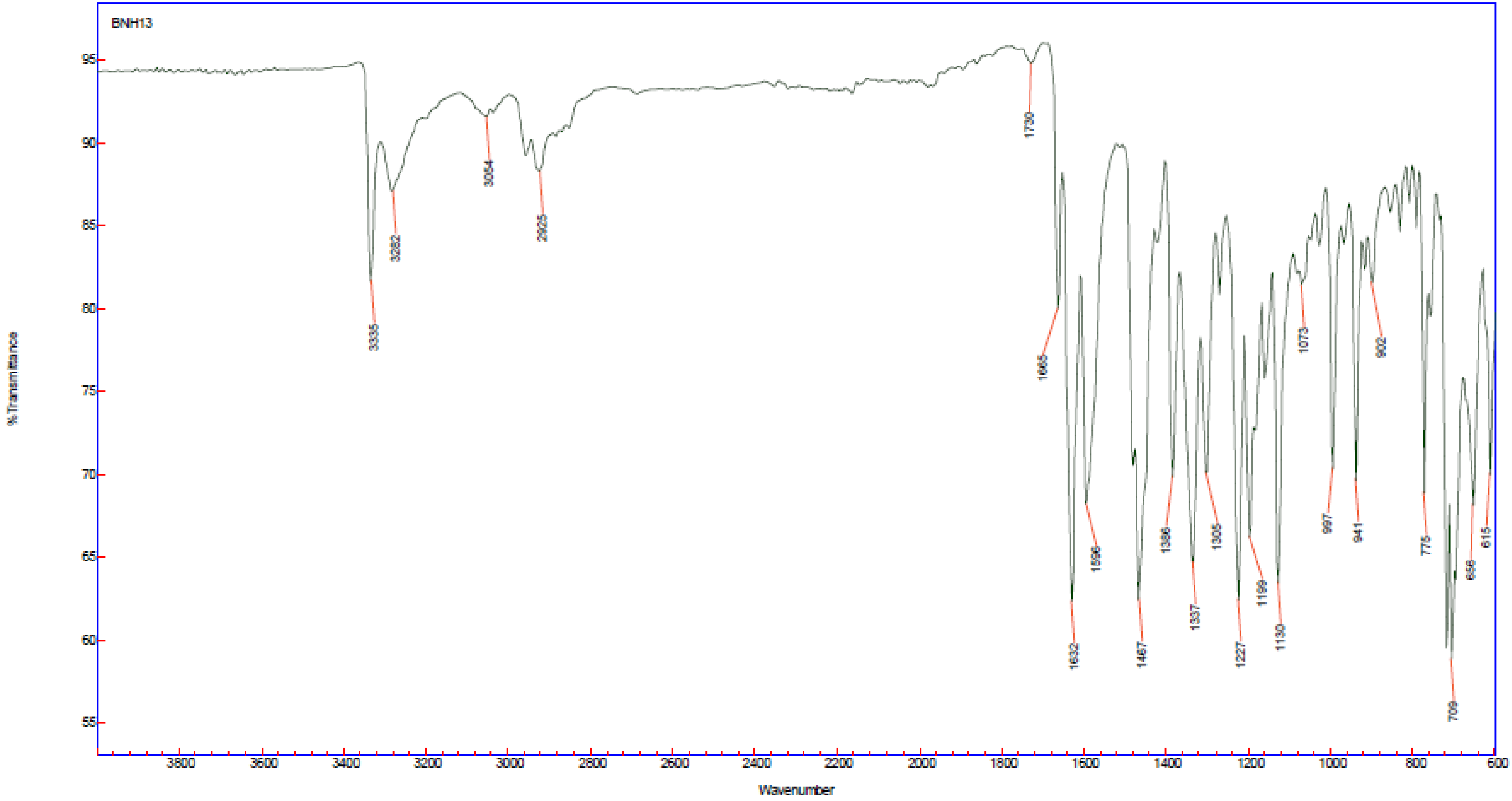

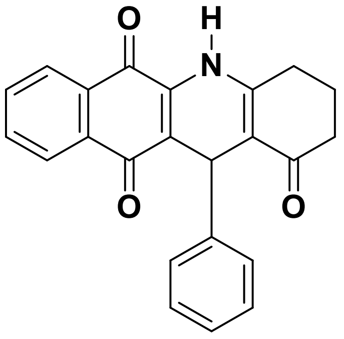

5a

IR spectrum of compound **5a**

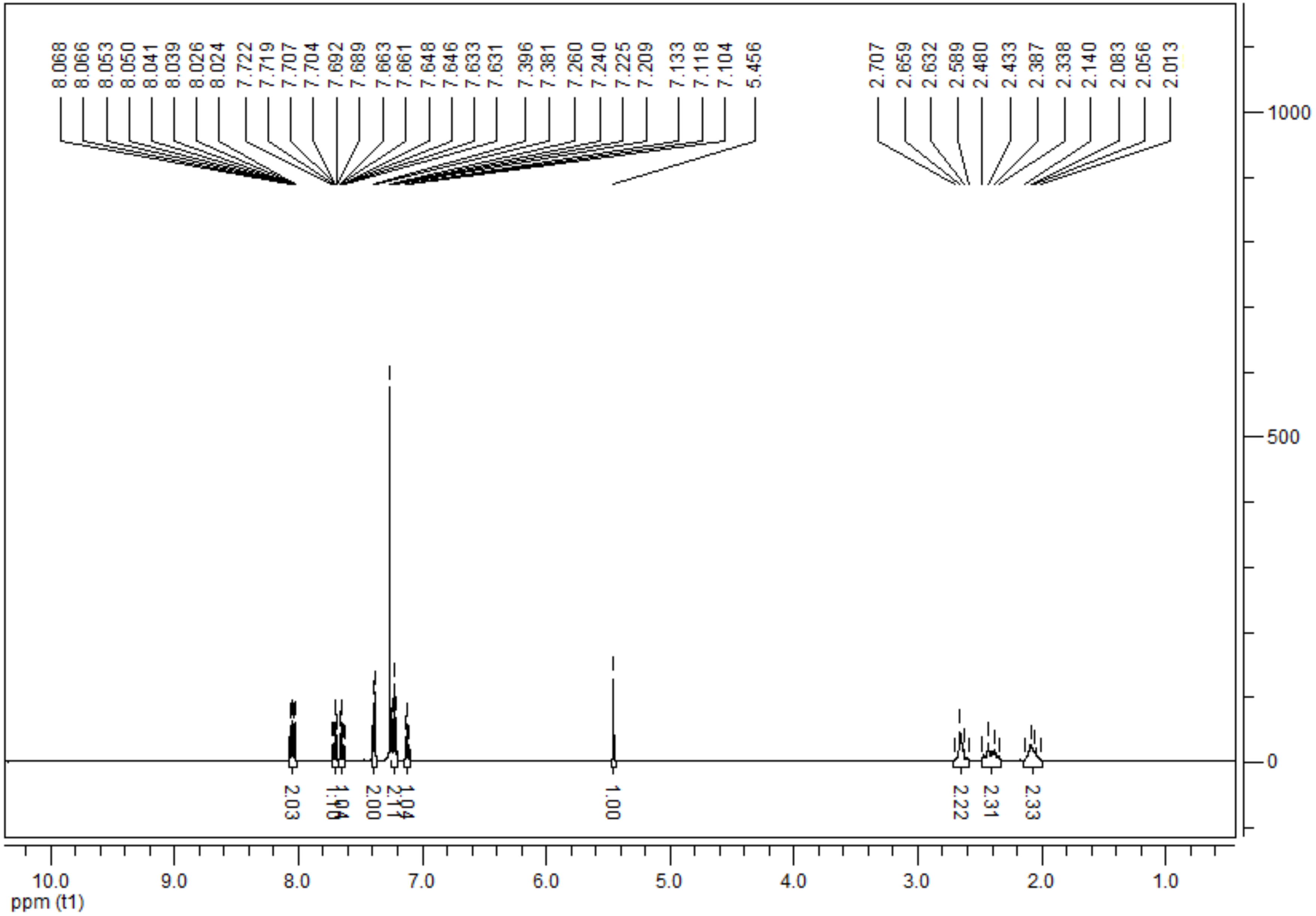

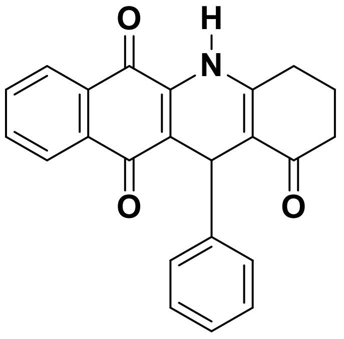

5a

^{1}H NMR spectrum of compound **5a** (500.00 MHz, $CDCl_3$)

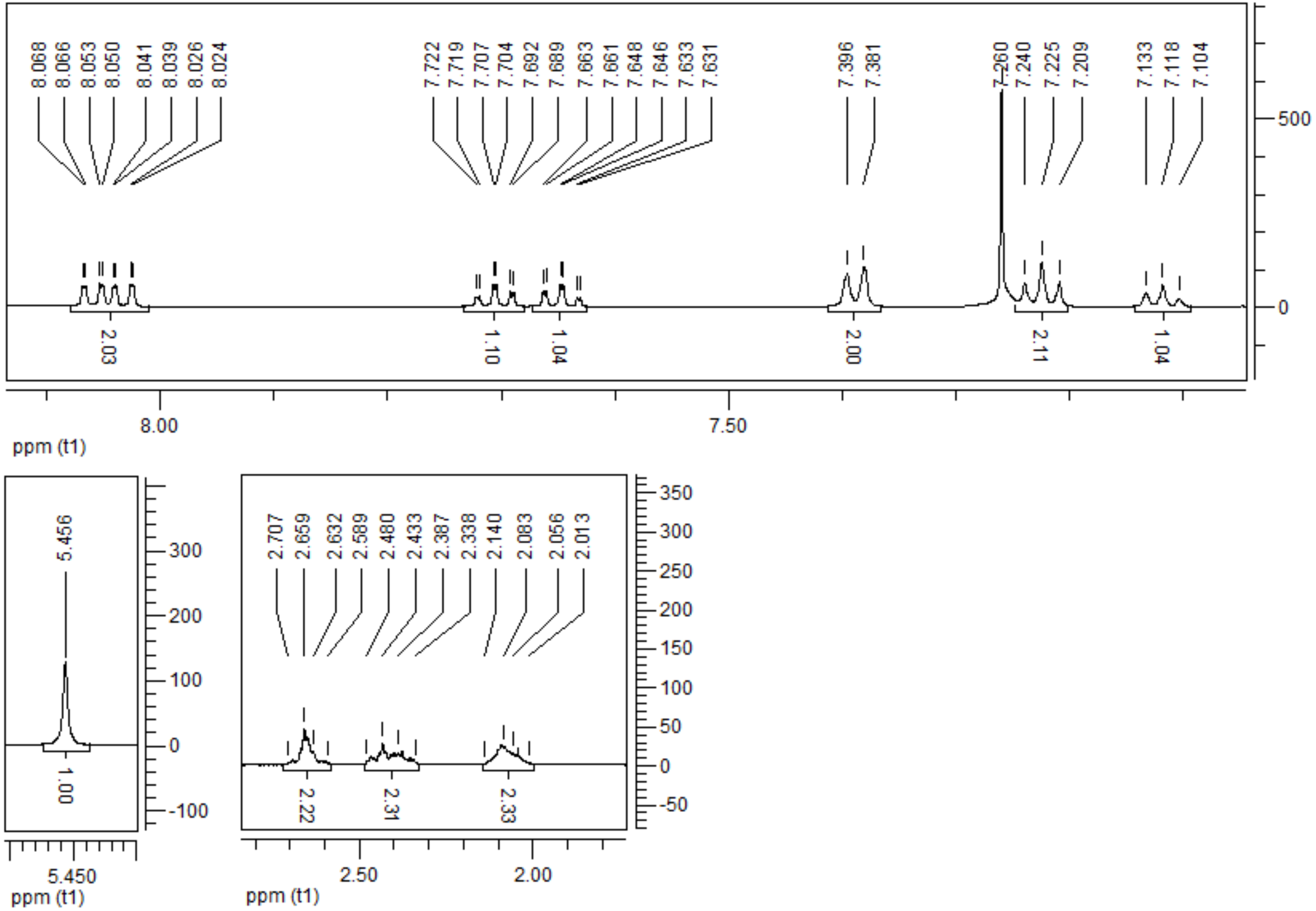

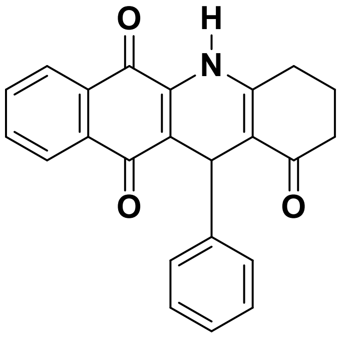

5a

^{1}H NMR spectrum of compound **5a** (500.00 MHz, $CDCl_3$)

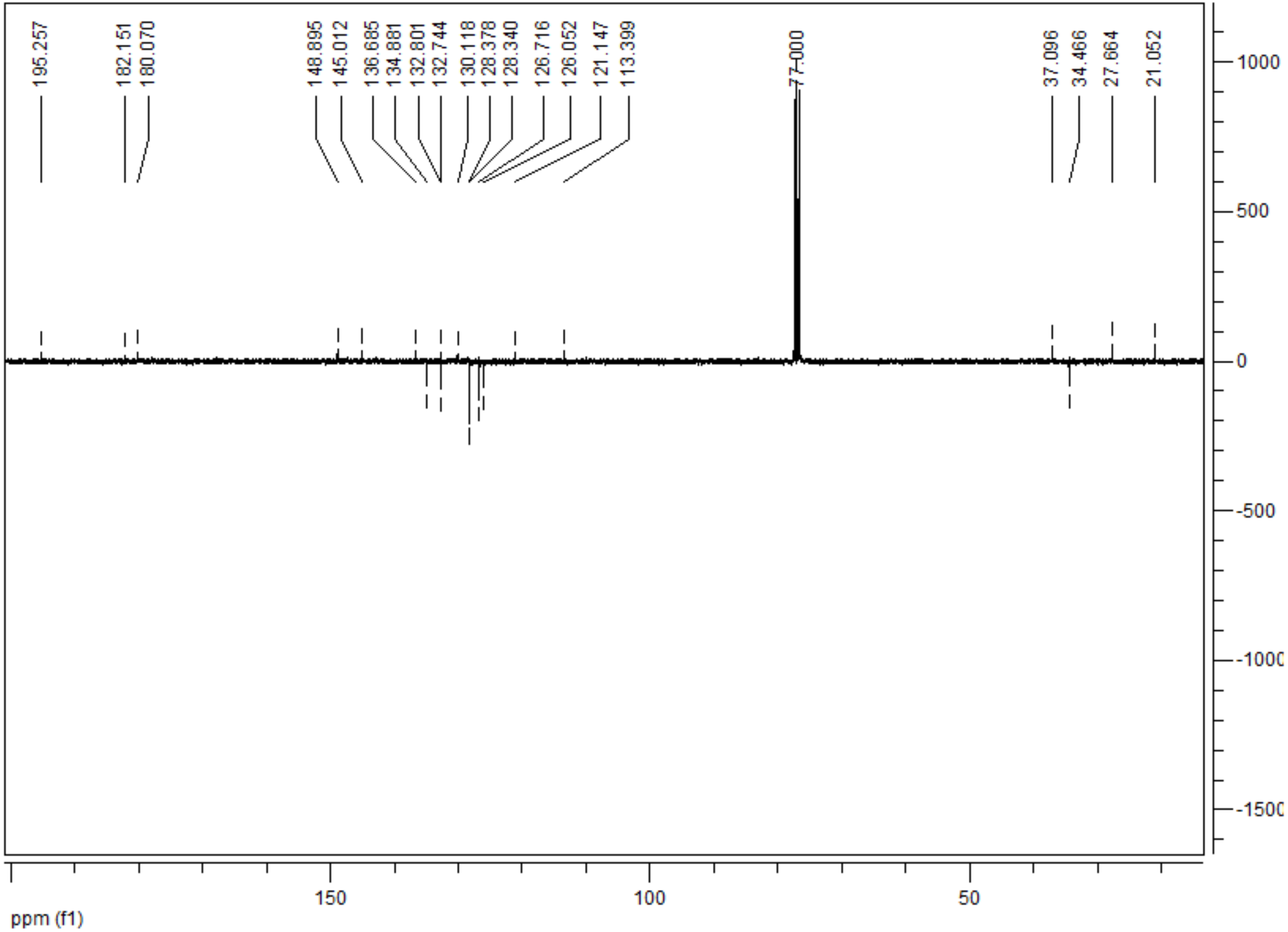

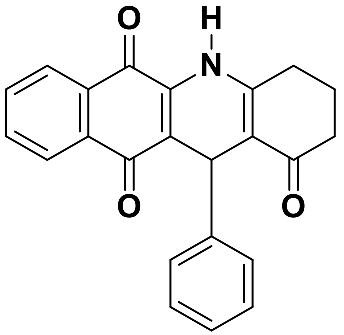

5a

^{13}C NMR spectrum of compound **5a** (75.0 MHz, $CDCl_3$)

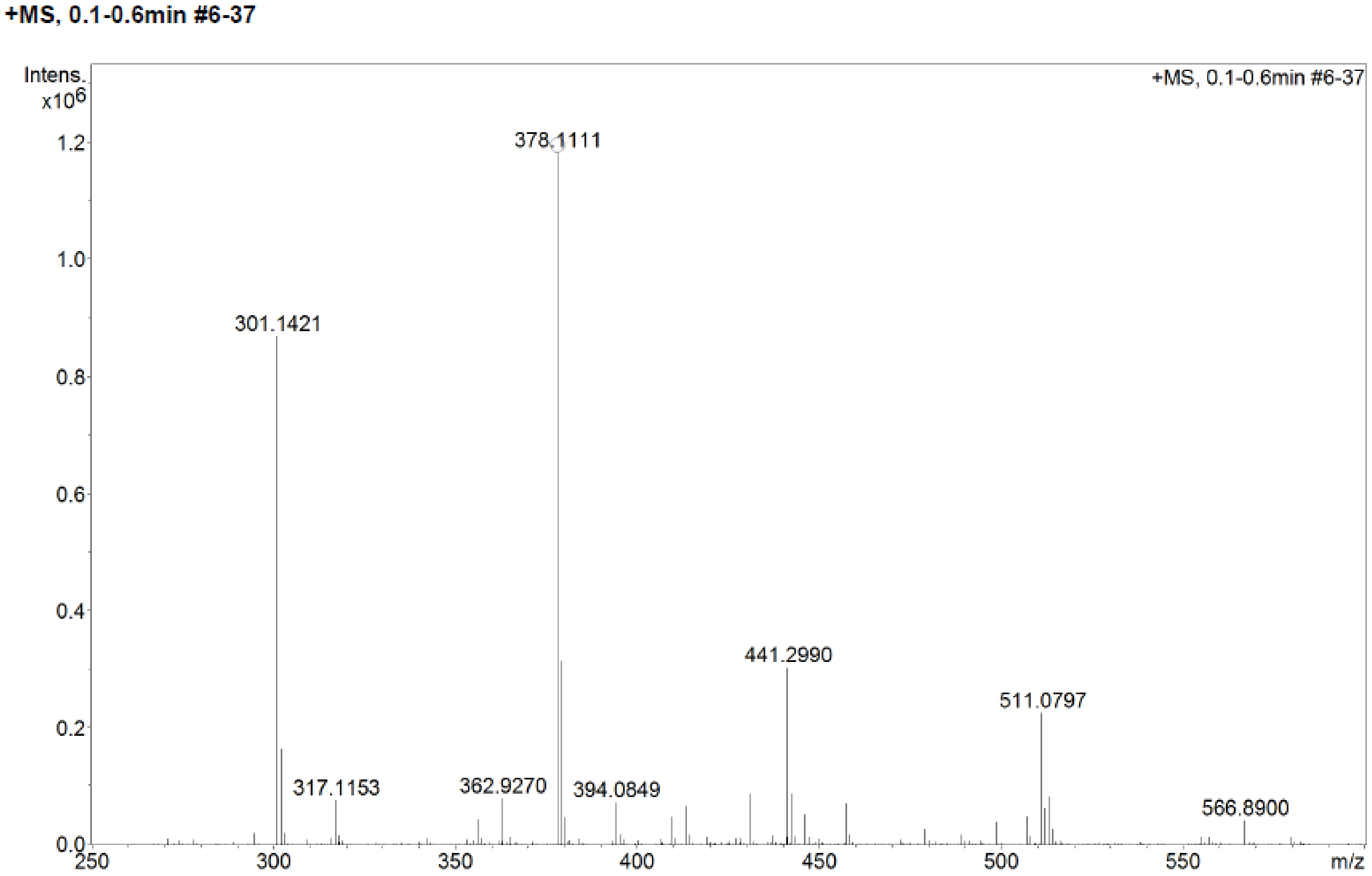

Meas. m/z	#	Ion Formula	m/z	err [ppm]	Mean err [ppm]	rdb	N-Rule	e^- Conf	mSigma	Std I	Std Mean m/z	Std I VarNorm	Std m/z Diff	Std Comb Dev
378.111144	1	C23H17NNaO3	378.110064	-2.9	-3.4	15.5	ok	even	6.8	10.7	n.a.	n.a.	n.a.	n.a.
	2	C19H13N7NaO	378.107379	-10.0	-11.5	16.5	ok	even	20.9	35.0	n.a.	n.a.	n.a.	n.a.

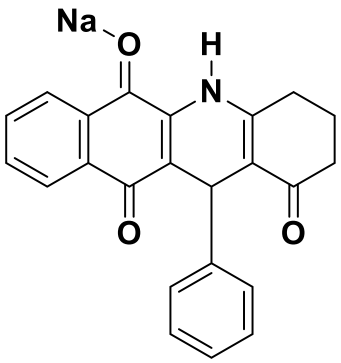

Exact Mass: 378,1106

5a

HRMS of compound **5a**

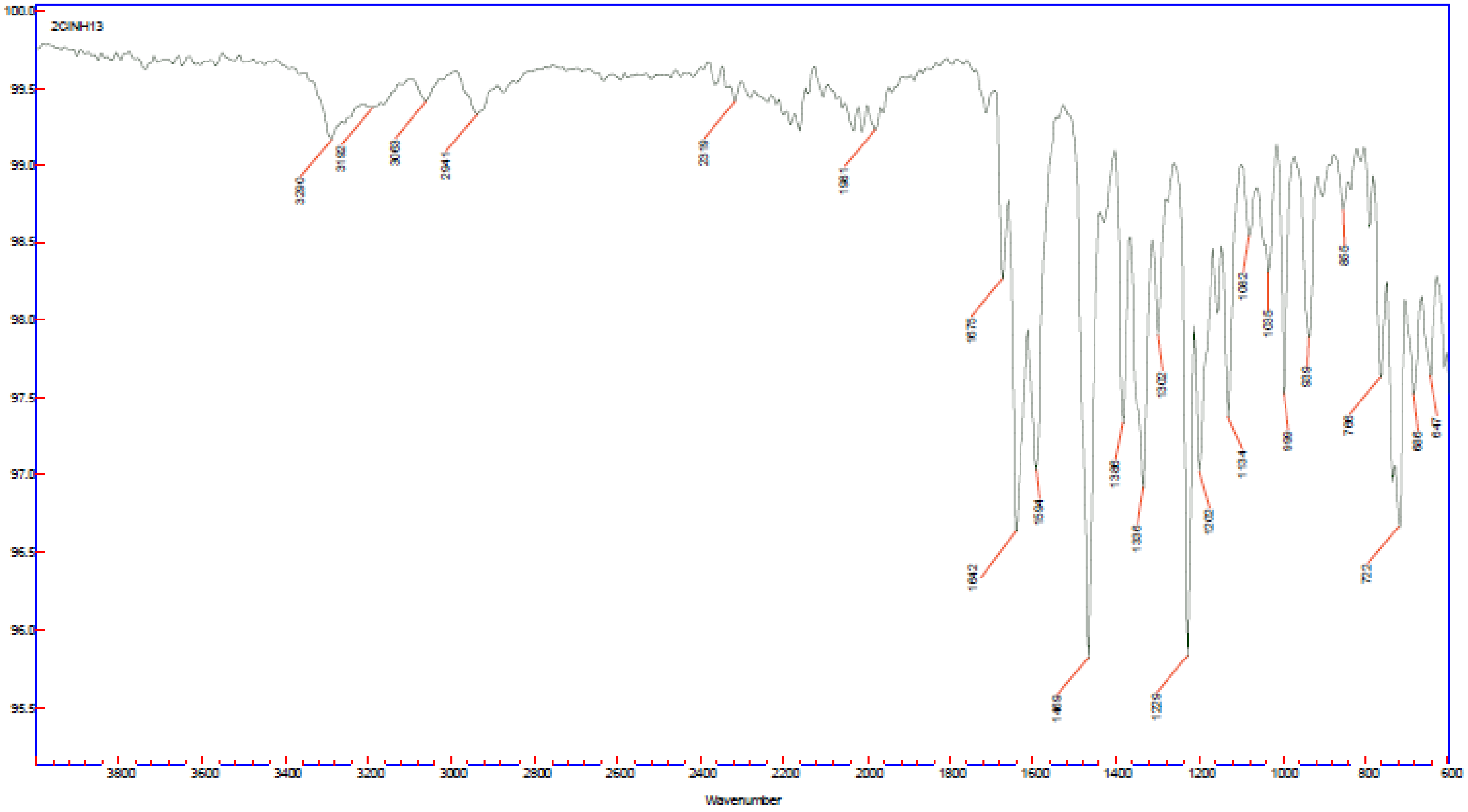

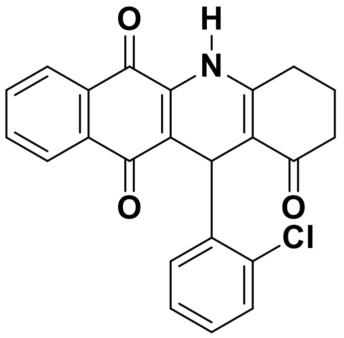

5b

IR spectrum of compound **5b**

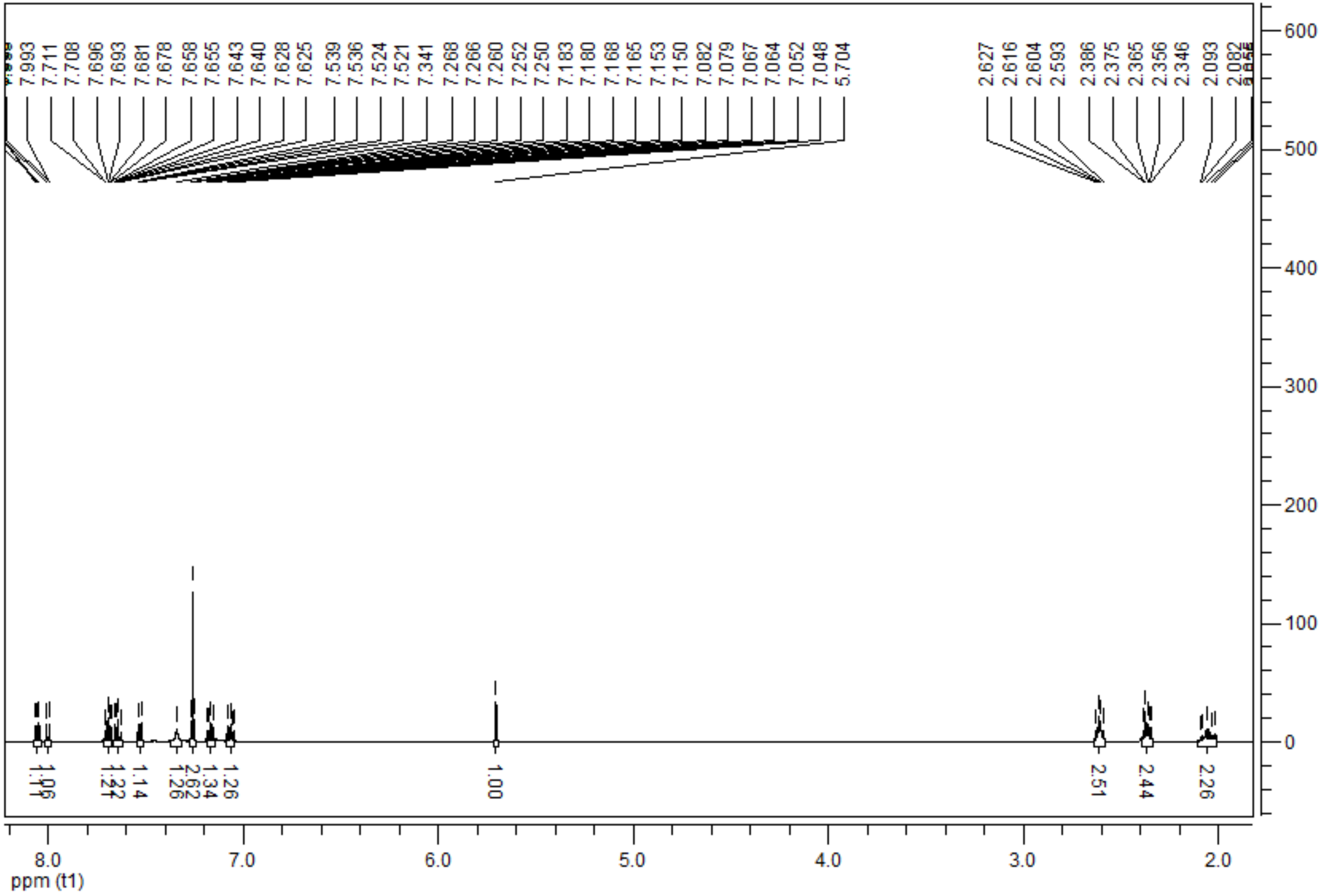

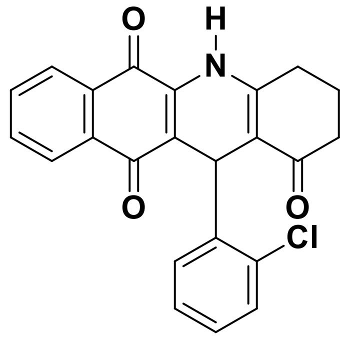

5b

^1H NMR spectrum of compound **5b** (500.00 MHz, $CDCl_3$)

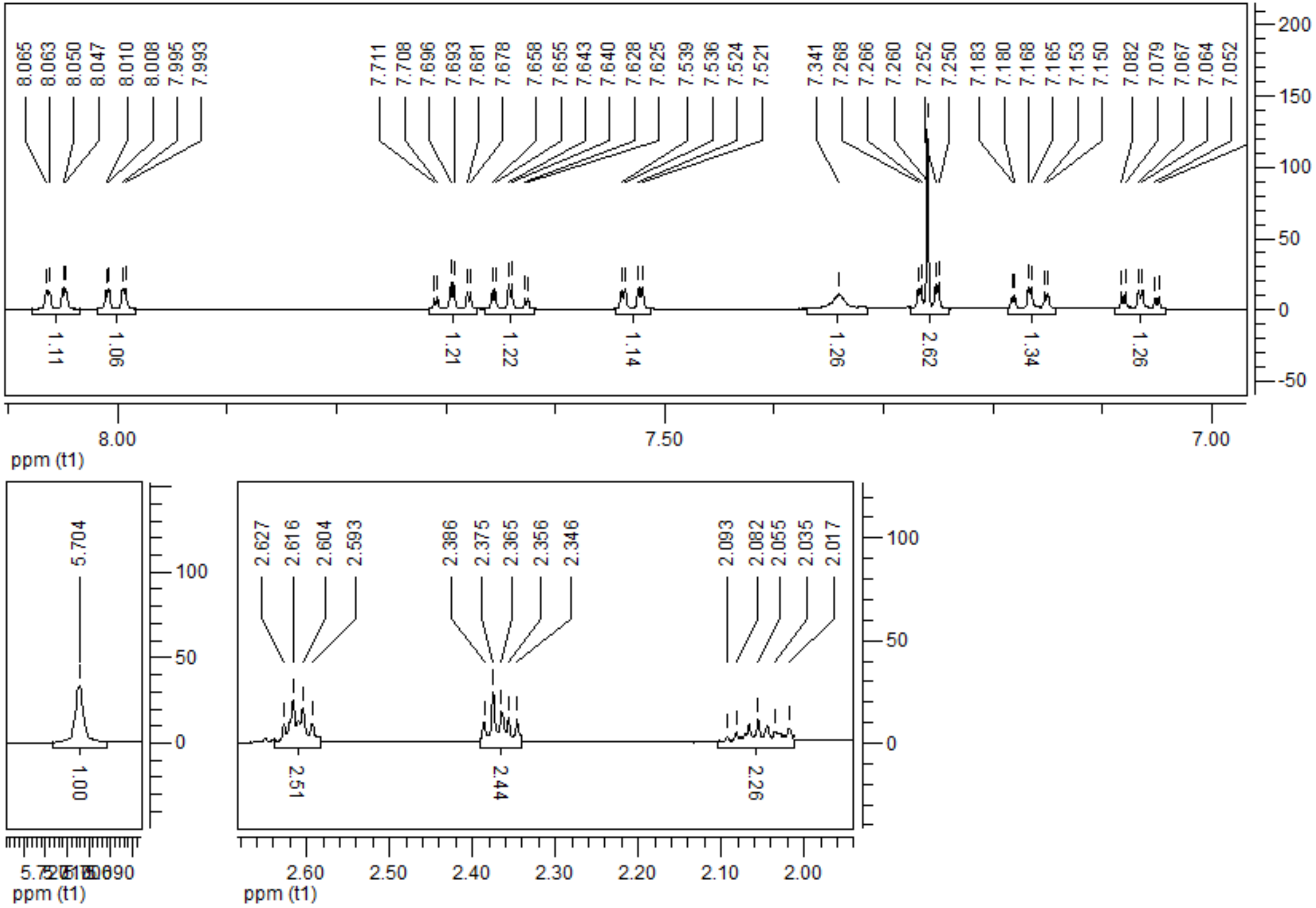

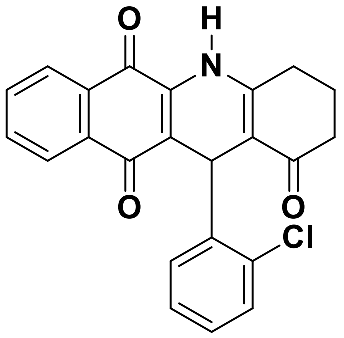

5b

^{1}H NMR spectrum of compound **5b** (500.00 MHz, $CDCl_3$)

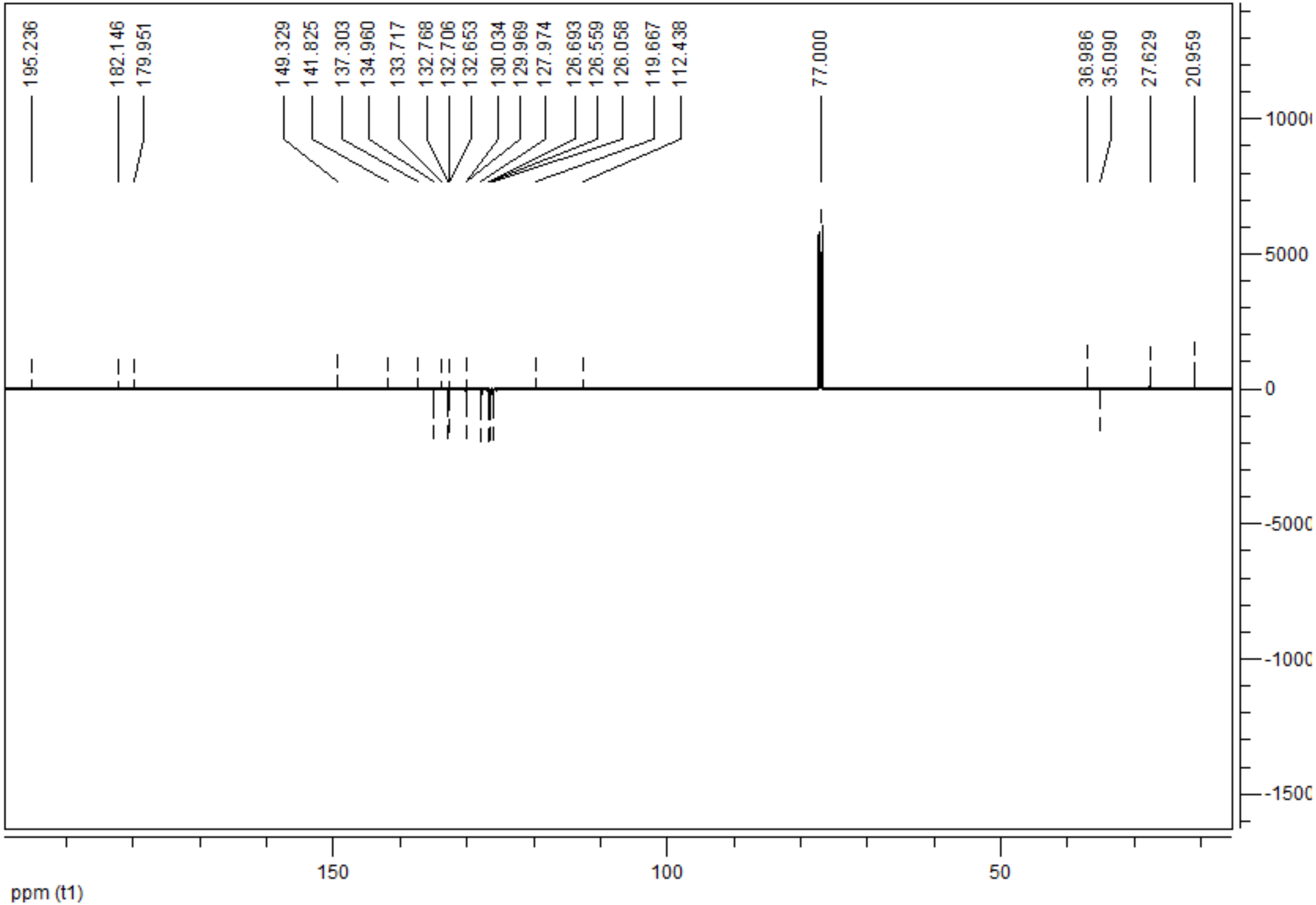

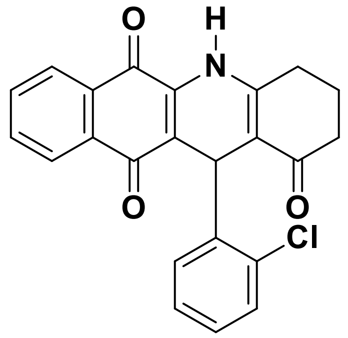

5b

^{13}C NMR spectrum of compound **5b** (125.0 MHz, $CDCl_3$)

+MS, 0.5-0.5min #27-30

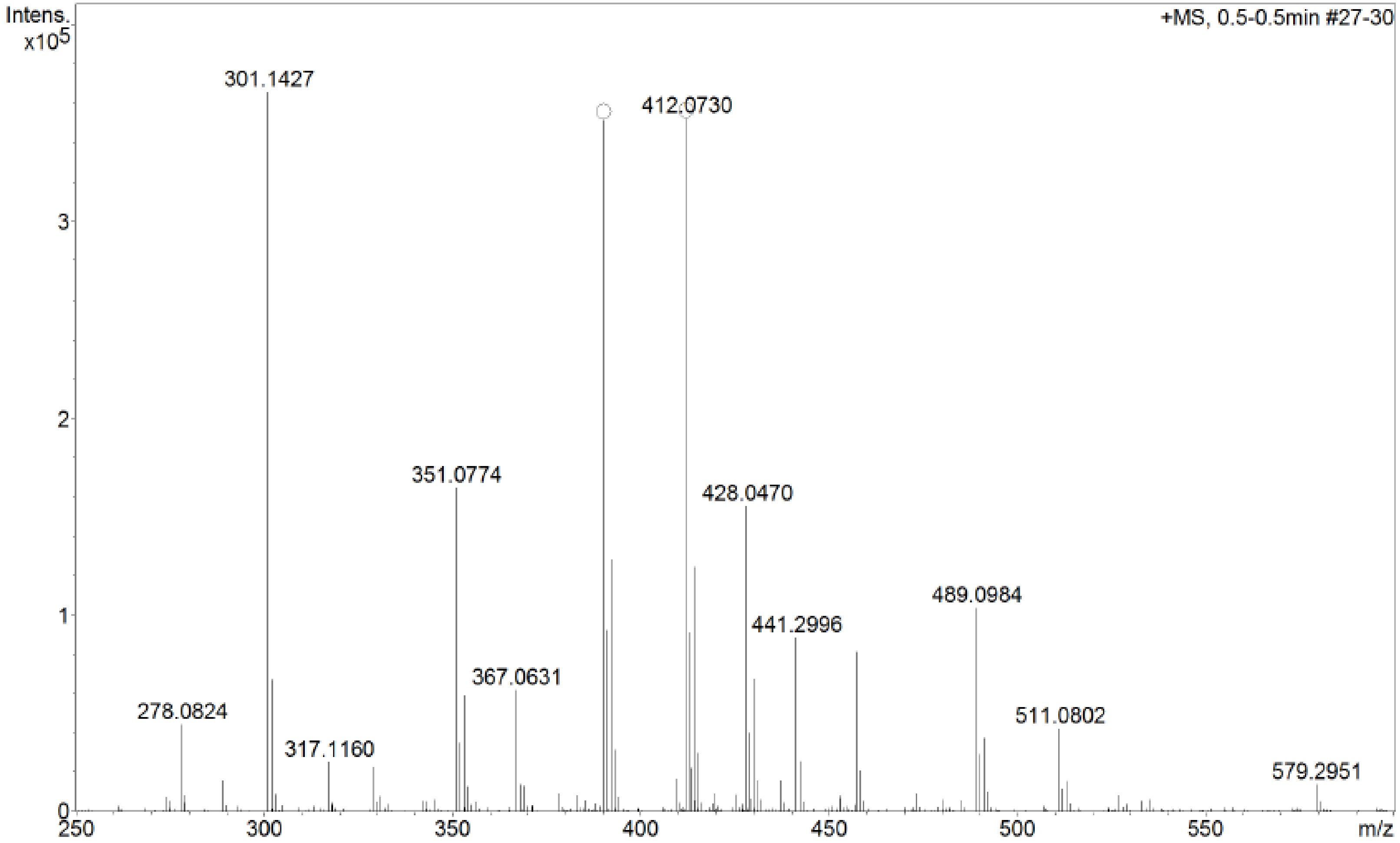

Meas. m/z	#	Ion Formula	m/z	err [ppm]	Mean err [ppm]	rdb	N-Rule	e⁻ Conf	mSigma	Std I	Std Mean m/z	Std I VarNorm	Std m/z Diff	Std Comb Dev
390.090698	1	C23H17ClNO3	390.089148	-4.0	-3.3	15.5	ok	even	7.0	11.8	n.a.	n.a.	n.a.	n.a.
412.073029	1	C23H16ClNNaO3	412.071092	-4.7	-4.2	15.5	ok	even	2.5	3.3	n.a.	n.a.	n.a.	n.a.

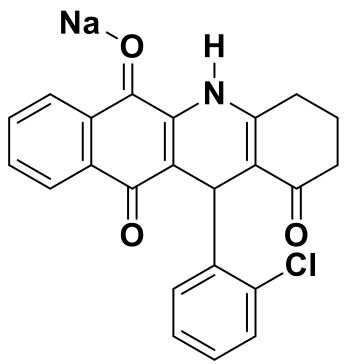

Exact Mass: 412,0716

5b

HRMS of compound **5b**

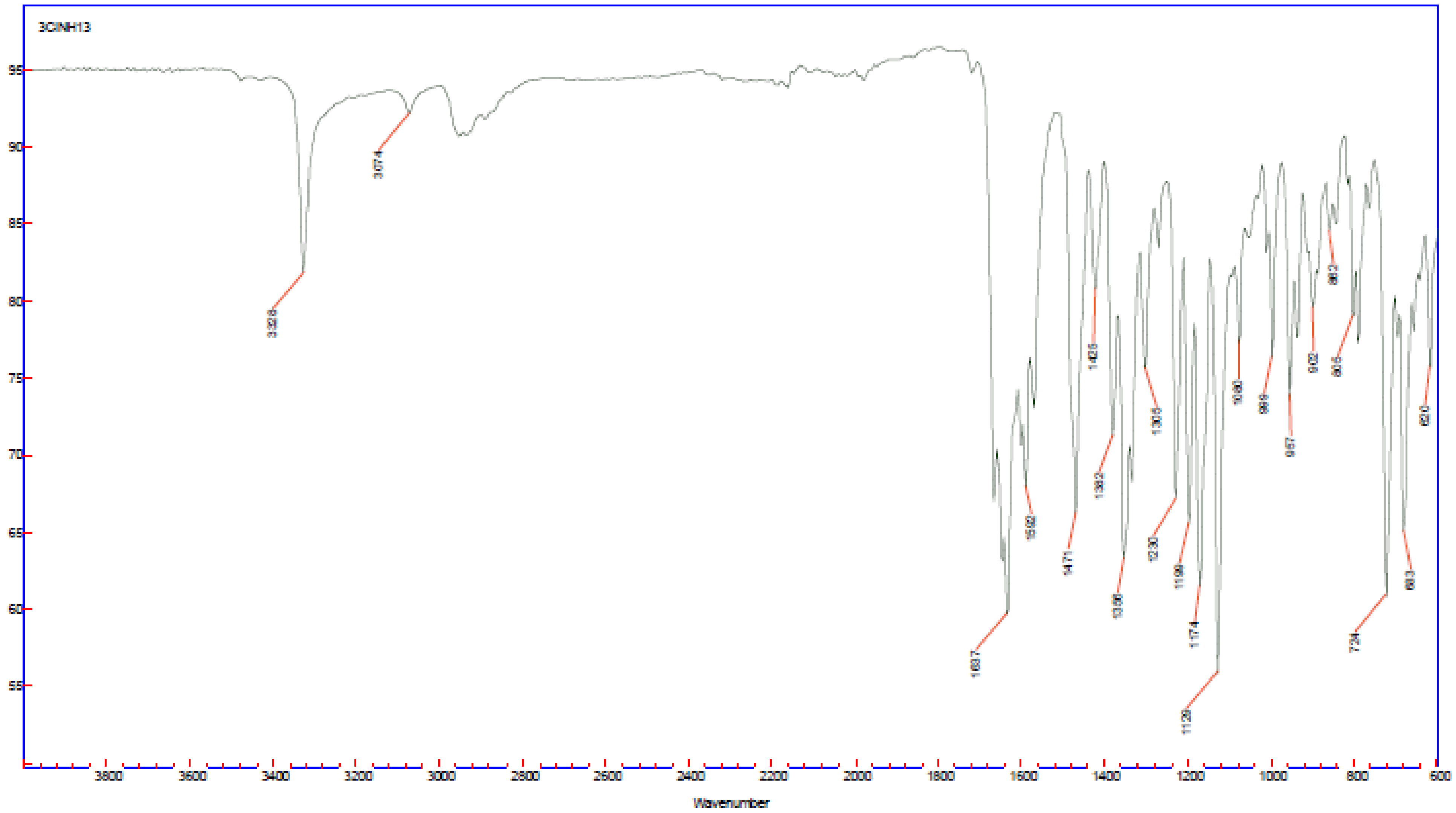

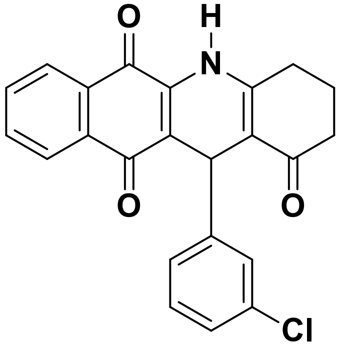

5c

IR spectrum of compound **5c**

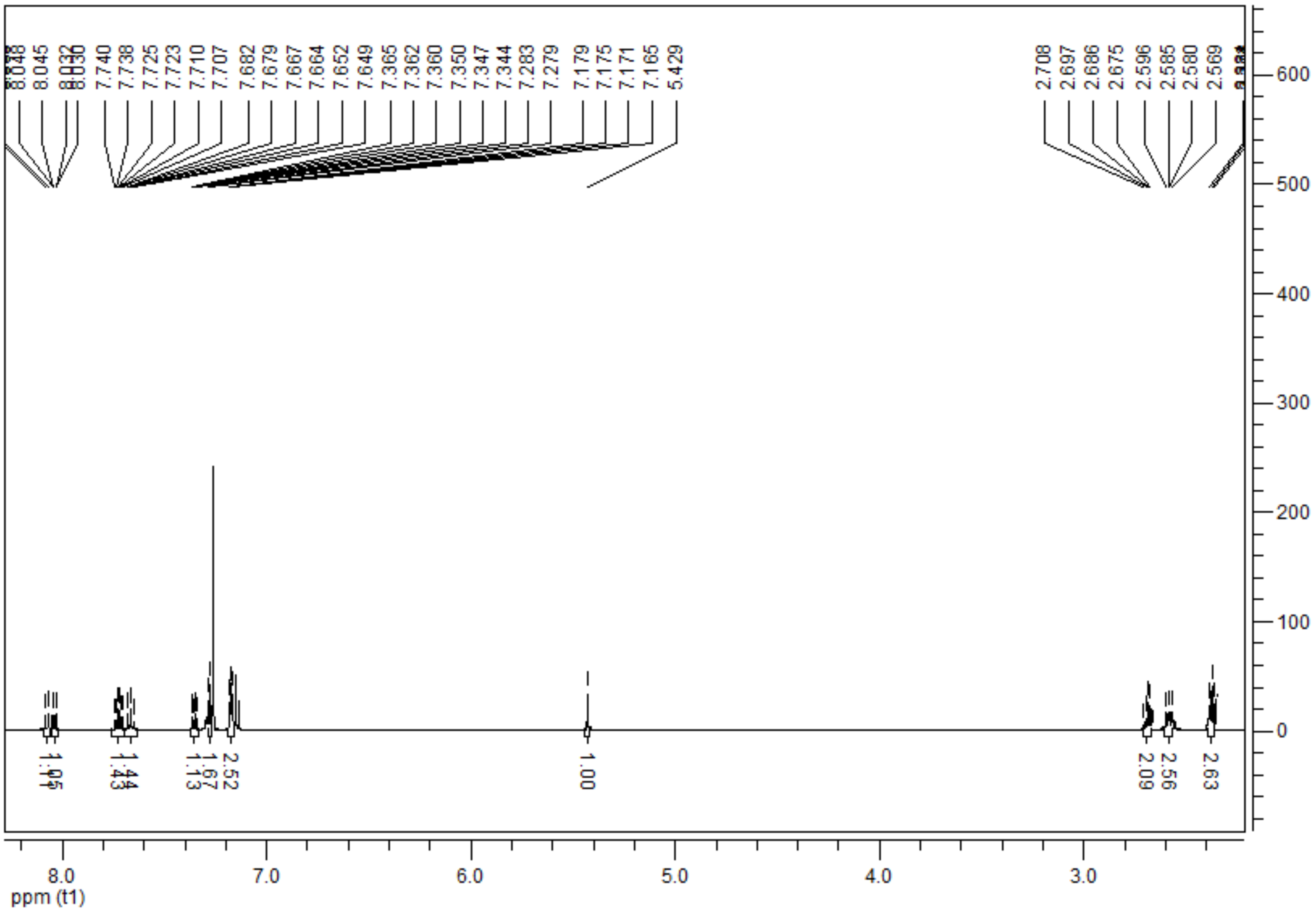

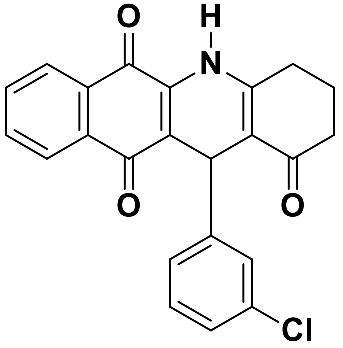

5c

^{1}H NMR spectrum of compound **5c** (500.00 MHz, $CDCl_3$)

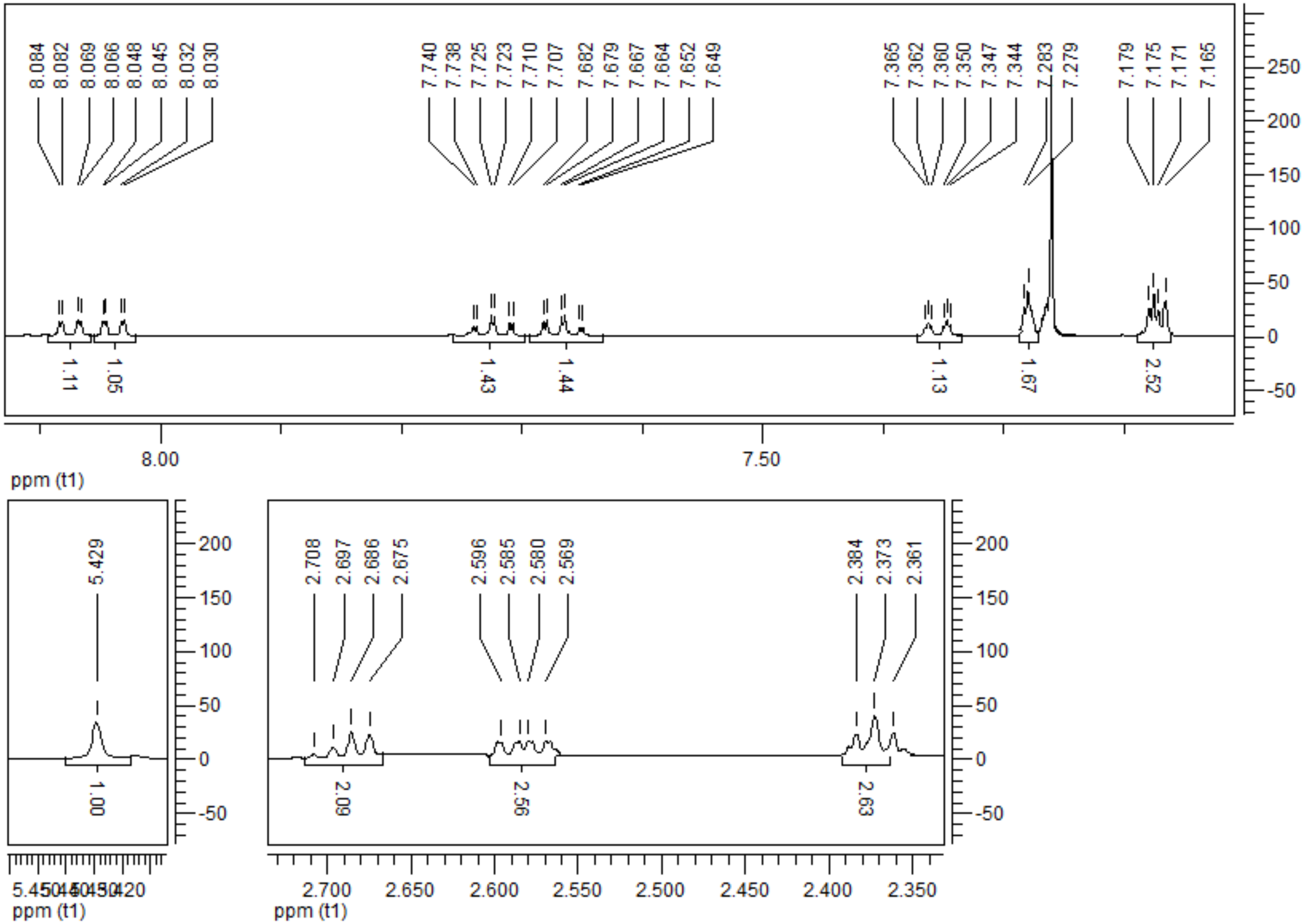

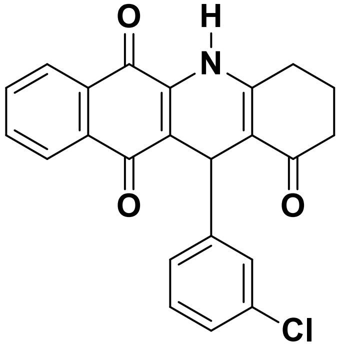

5c

^{1}H NMR spectrum of compound **5c** (500.00 MHz, $CDCl_3$)

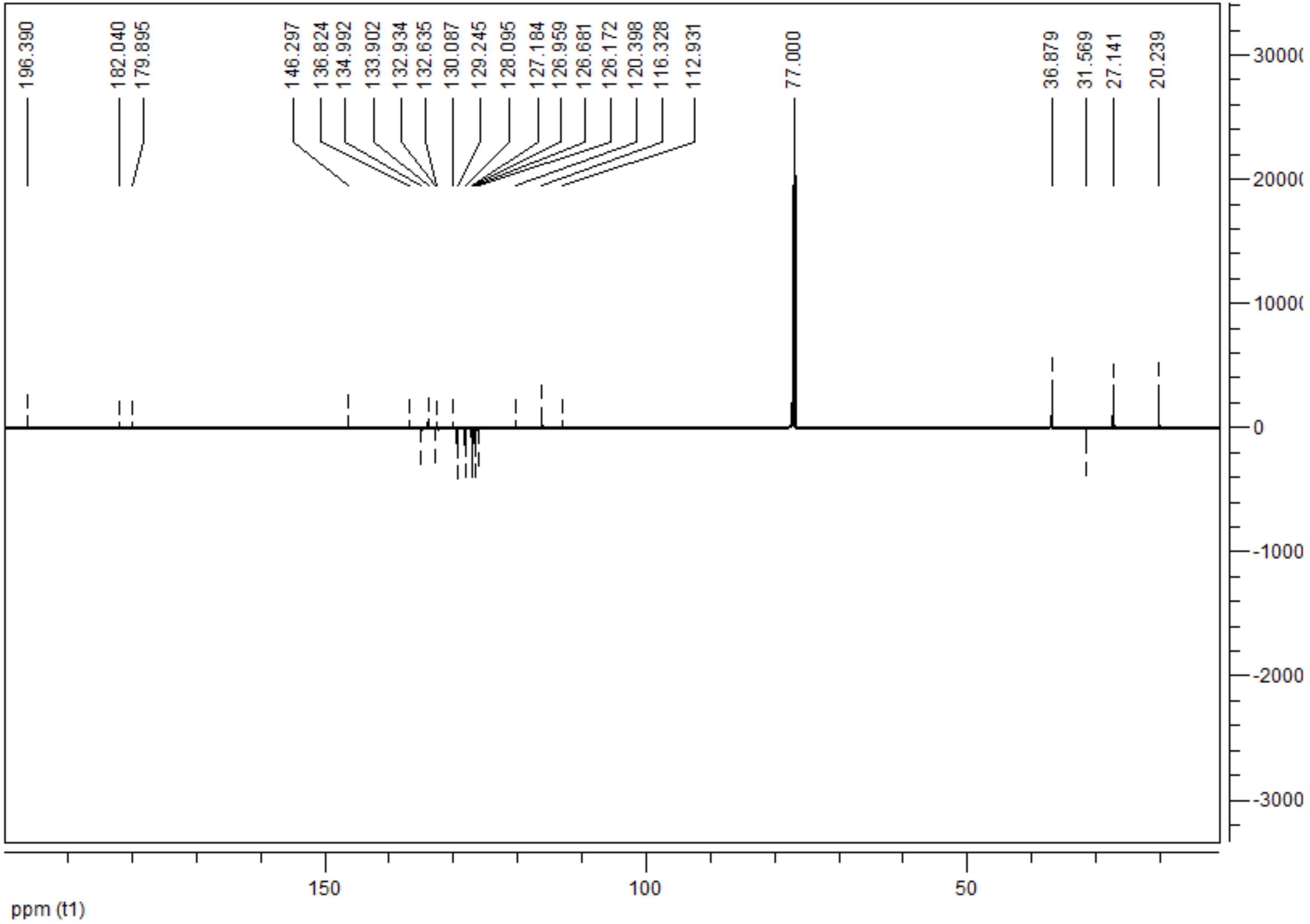

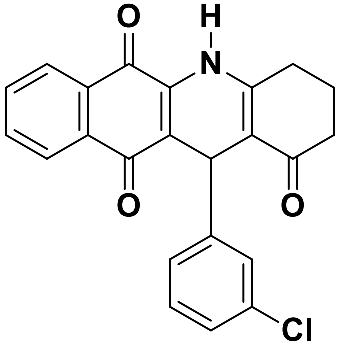

5c

^{13}C NMR spectrum of compound **5c** (125.0 MHz, $CDCl_3$)

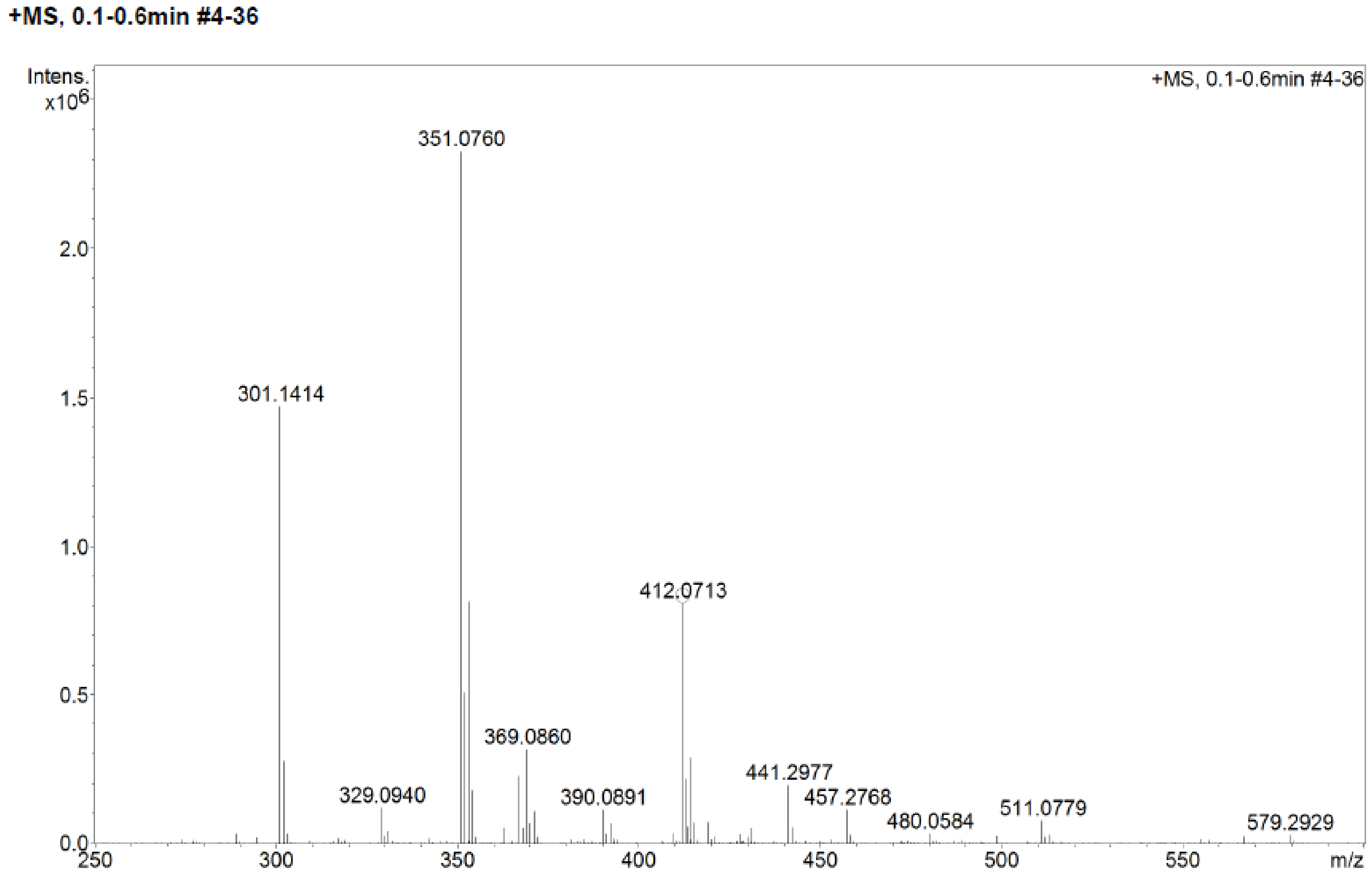

Meas. m/z	#	Ion Formula	m/z	err [ppm]	Mean err [ppm]	rdb	N-Rule	e⁻ Conf	mSigma	Std I	Std Mean m/z	Std I VarNorm	Std m/z Diff	Std Comb Dev
412.071268	1	C23H16ClNNaO3	412.071092	-0.4	0.3	15.5	ok	even	5.1	7.1	n.a.	n.a.	n.a.	n.a.
	2	C19H12ClN7NaO	412.068406	-6.9	-7.2	16.5	ok	even	16.6	25.5	n.a.	n.a.	n.a.	n.a.

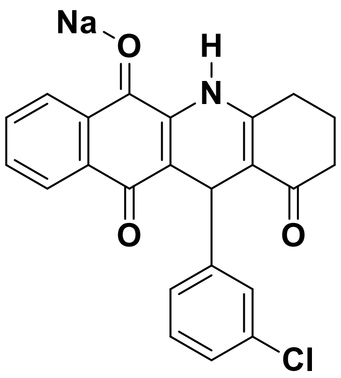

Exact Mass: 412,0716

5c

HRMS of compound **5c**

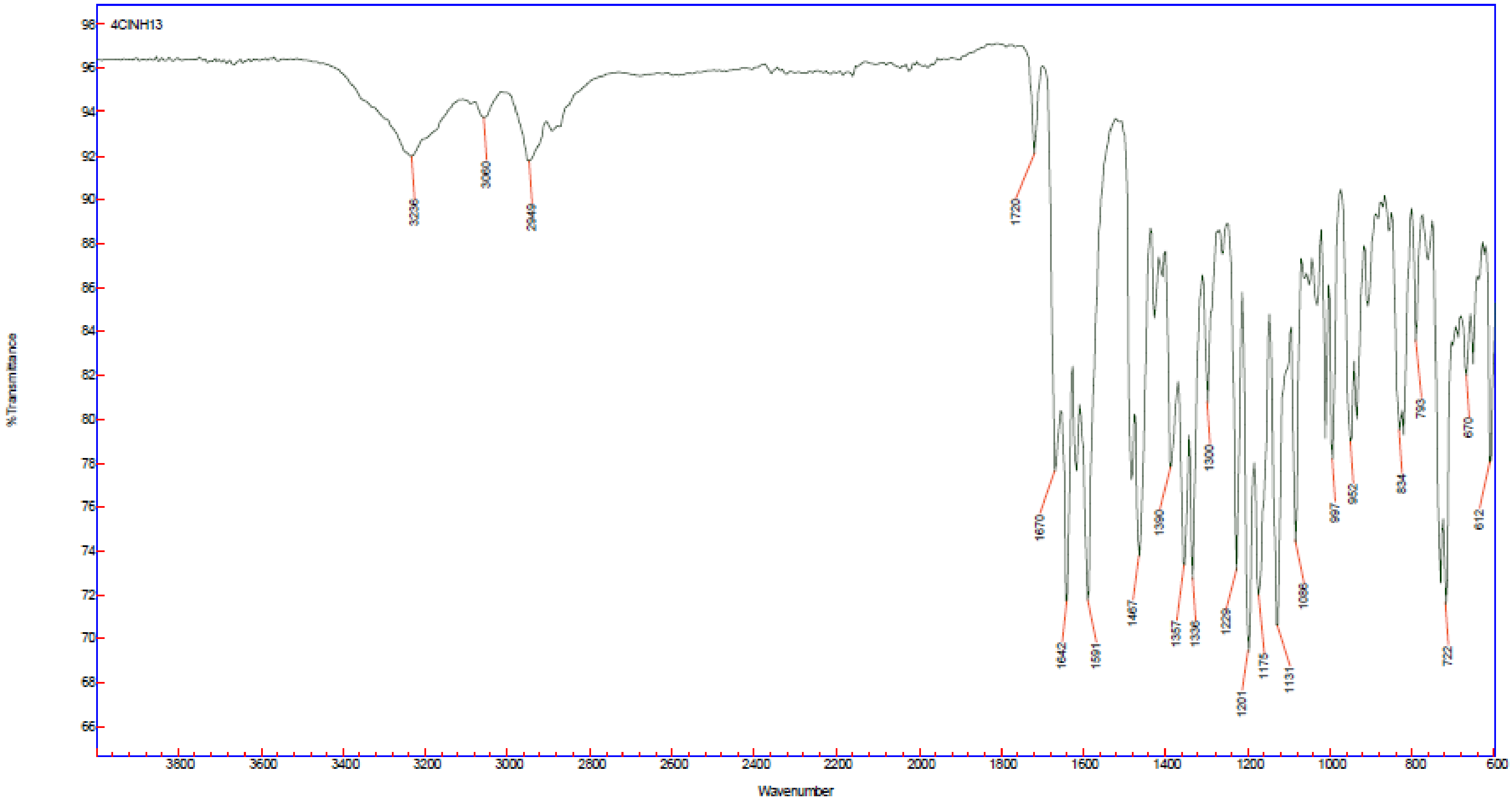

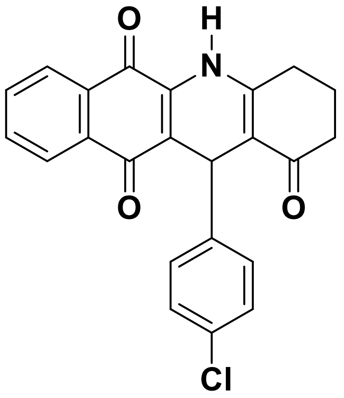

5d

IR spectrum of compound **5d**

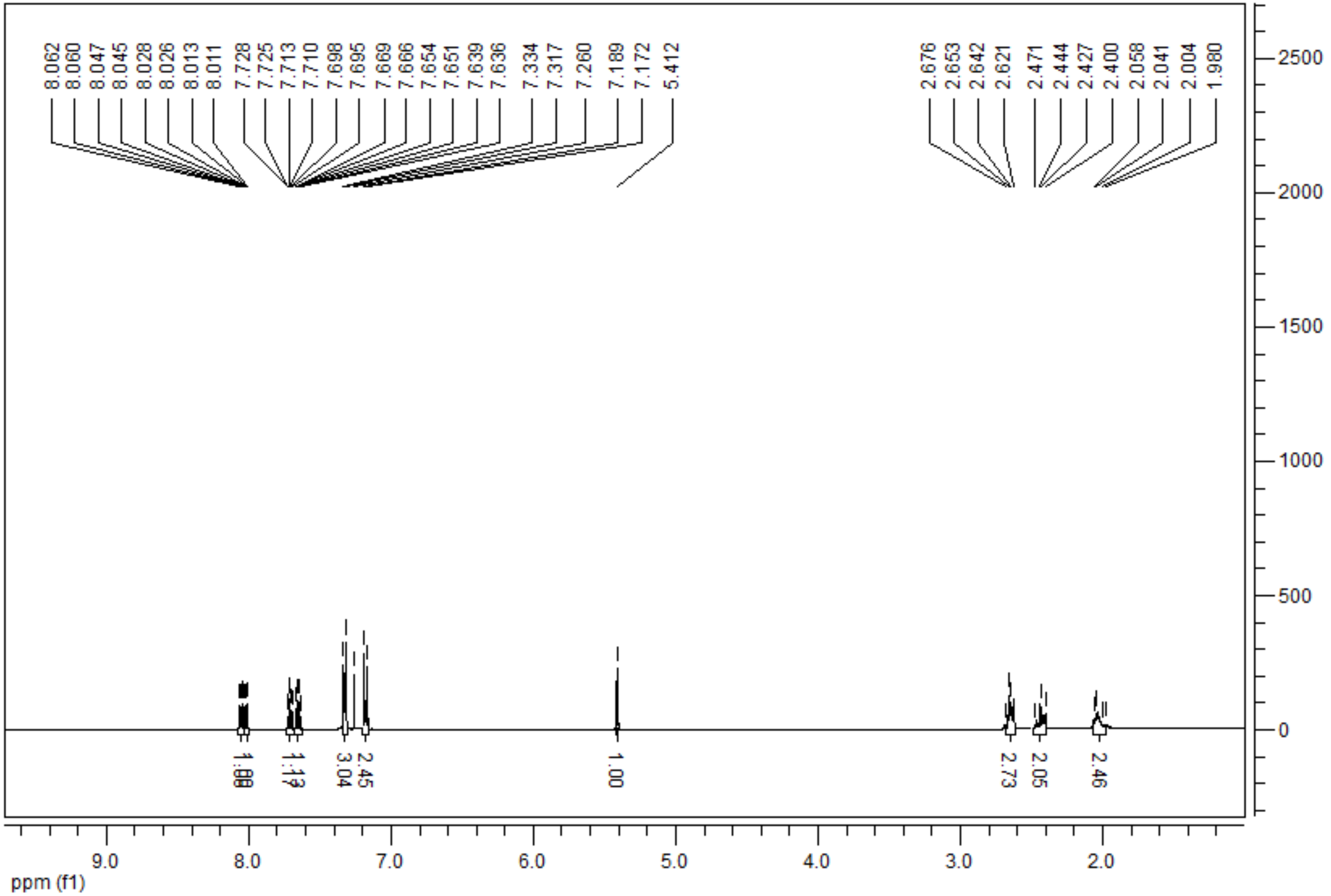

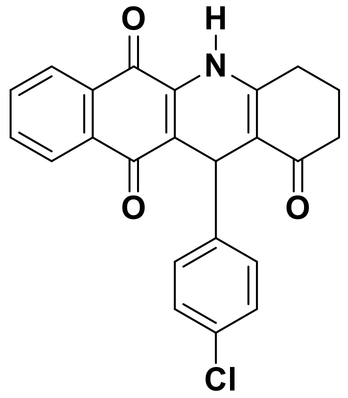

5d

^{1}H NMR spectrum of compound **5d** (500.00 MHz, $CDCl_3$)

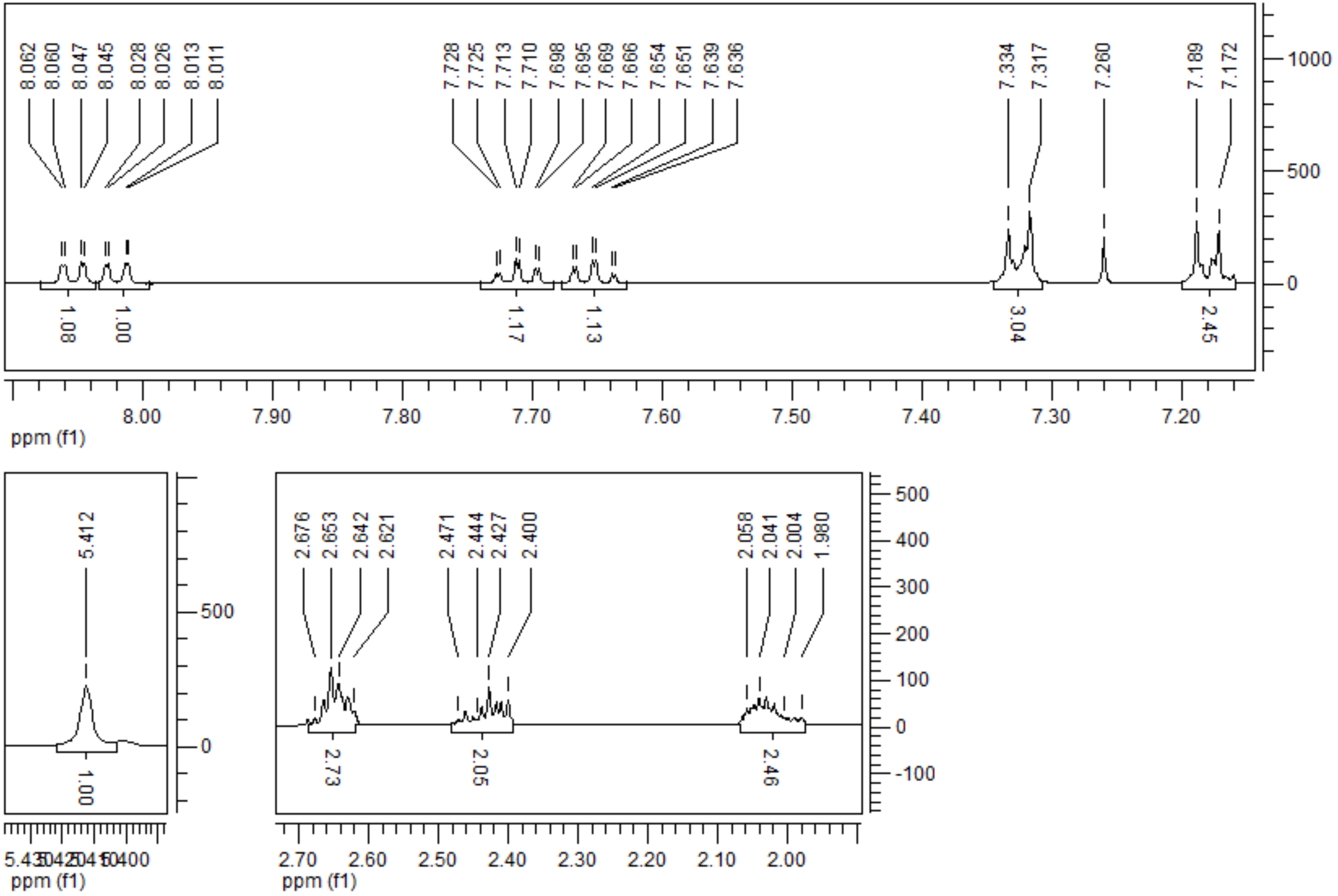

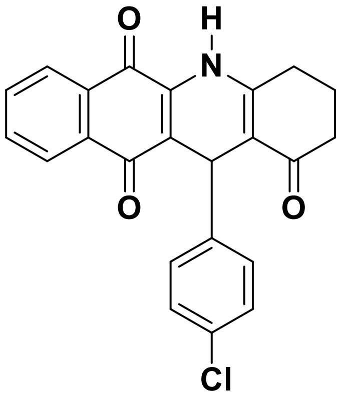

5d

^{1}H NMR spectrum of compound **5d** (500.00 MHz, $CDCl_3$)

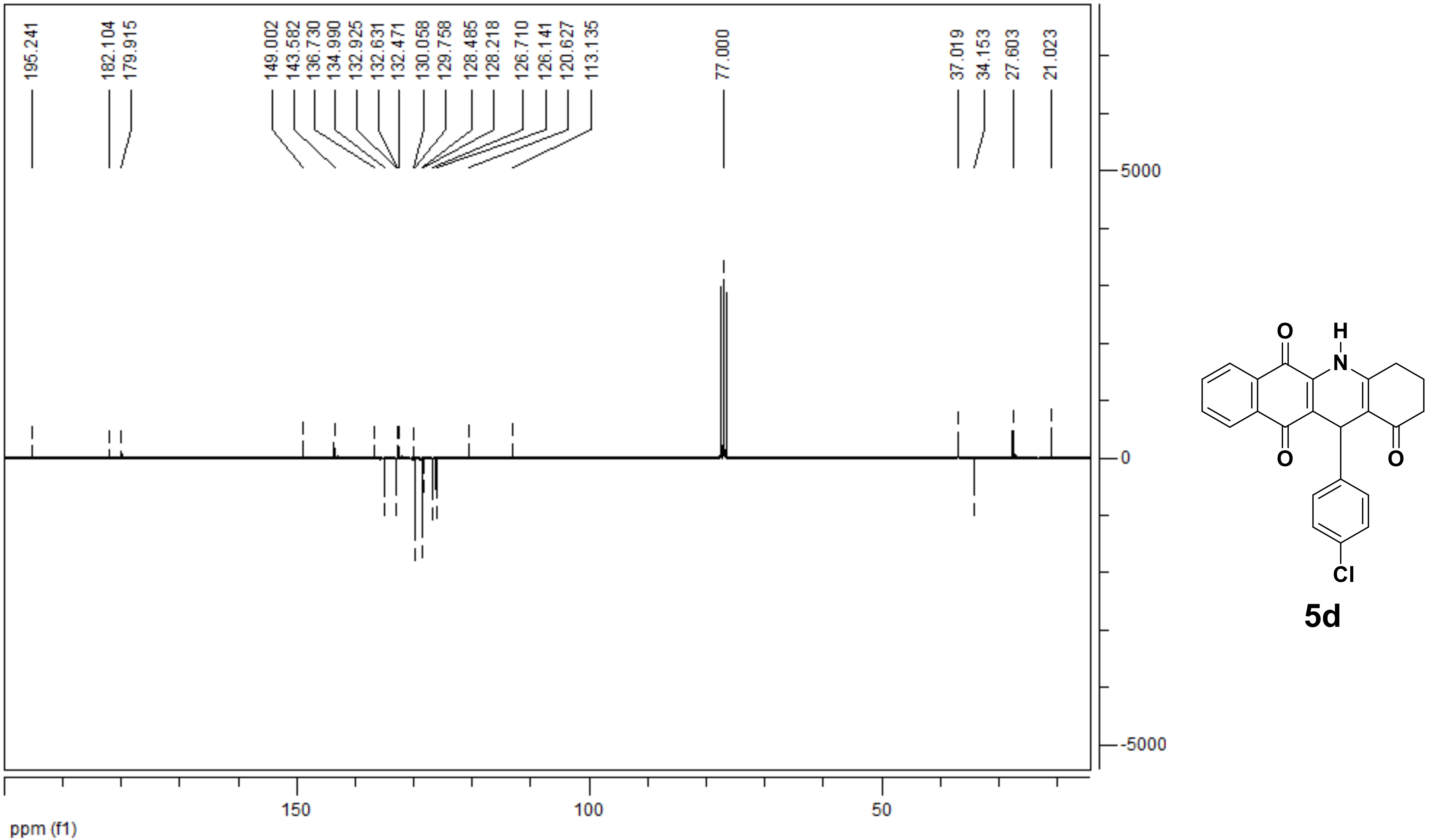

^{13}C NMR spectrum of compound **5d** (75.0 MHz, $CDCl_3$)

+MS, 0.3-0.7min #17-39

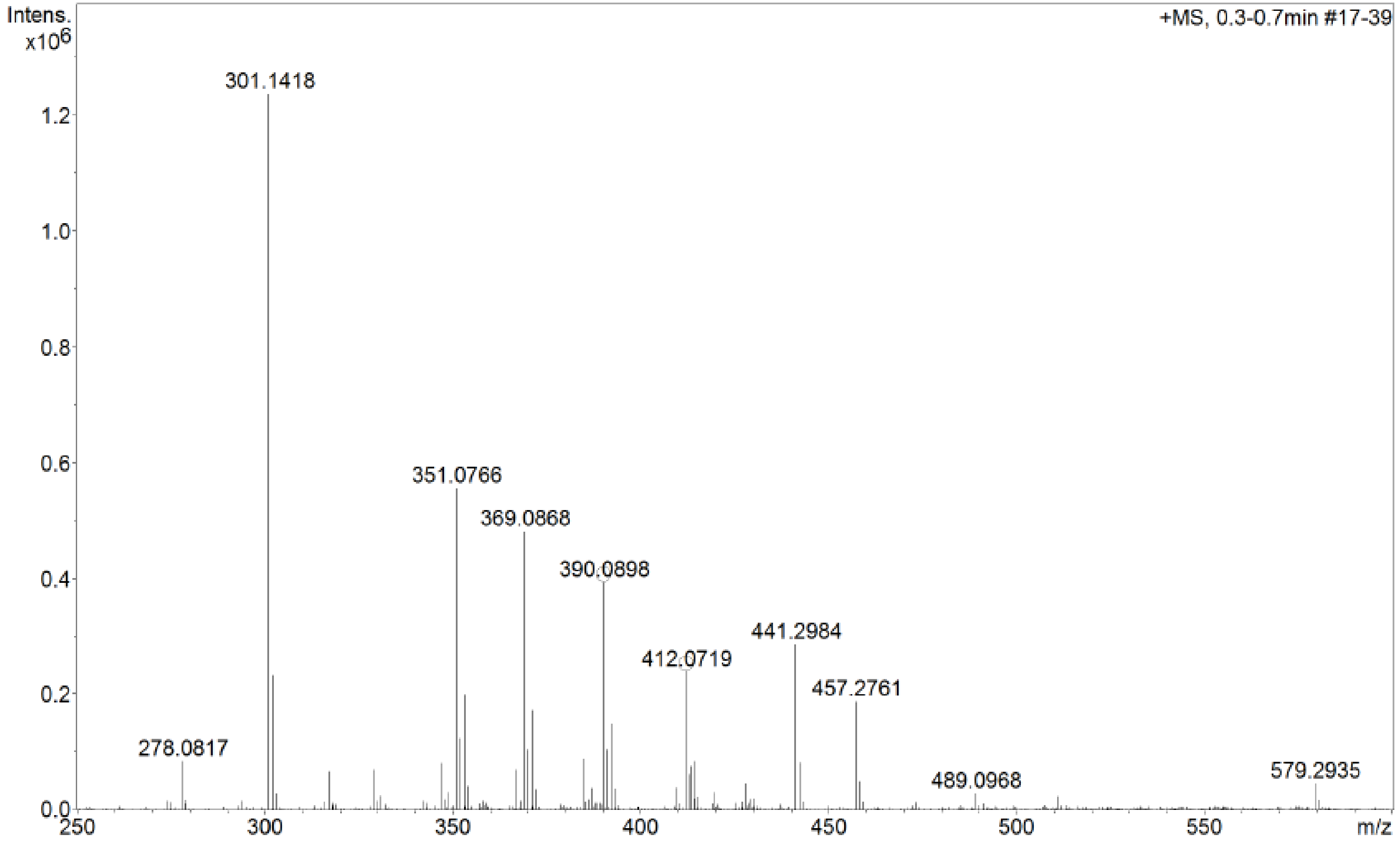

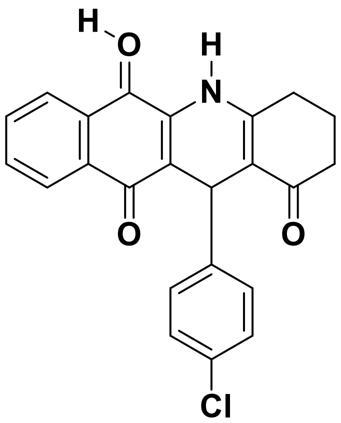

Exact Mass: 390,0897

5d

Meas. m/z	#	Ion Formula	m/z	err [ppm]	Mean err [ppm]	rdb	N-Rule	e⁻ Conf	mSigma	Std I	Std Mean m/z	Std I VarNorm	Std m/z Diff	Std Comb Dev
390.089822	1	C23H17ClNO3	390.089148	-1.7	-2.0	15.5	ok	even	11.0	17.0	n.a.	n.a.	n.a.	n.a.
	2	C19H13ClN7O	390.086462	-8.6	-9.9	16.5	ok	even	21.4	34.3	n.a.	n.a.	n.a.	n.a.
412.071891	1	C23H16ClNNaO3	412.071092	-1.9	-2.1	15.5	ok	even	4.9	6.6	n.a.	n.a.	n.a.	n.a.
	2	C19H12ClN7NaO	412.068406	-8.5	-9.7	16.5	ok	even	13.0	21.6	n.a.	n.a.	n.a.	n.a.

HRMS of compound **5d**

IR (KBr)

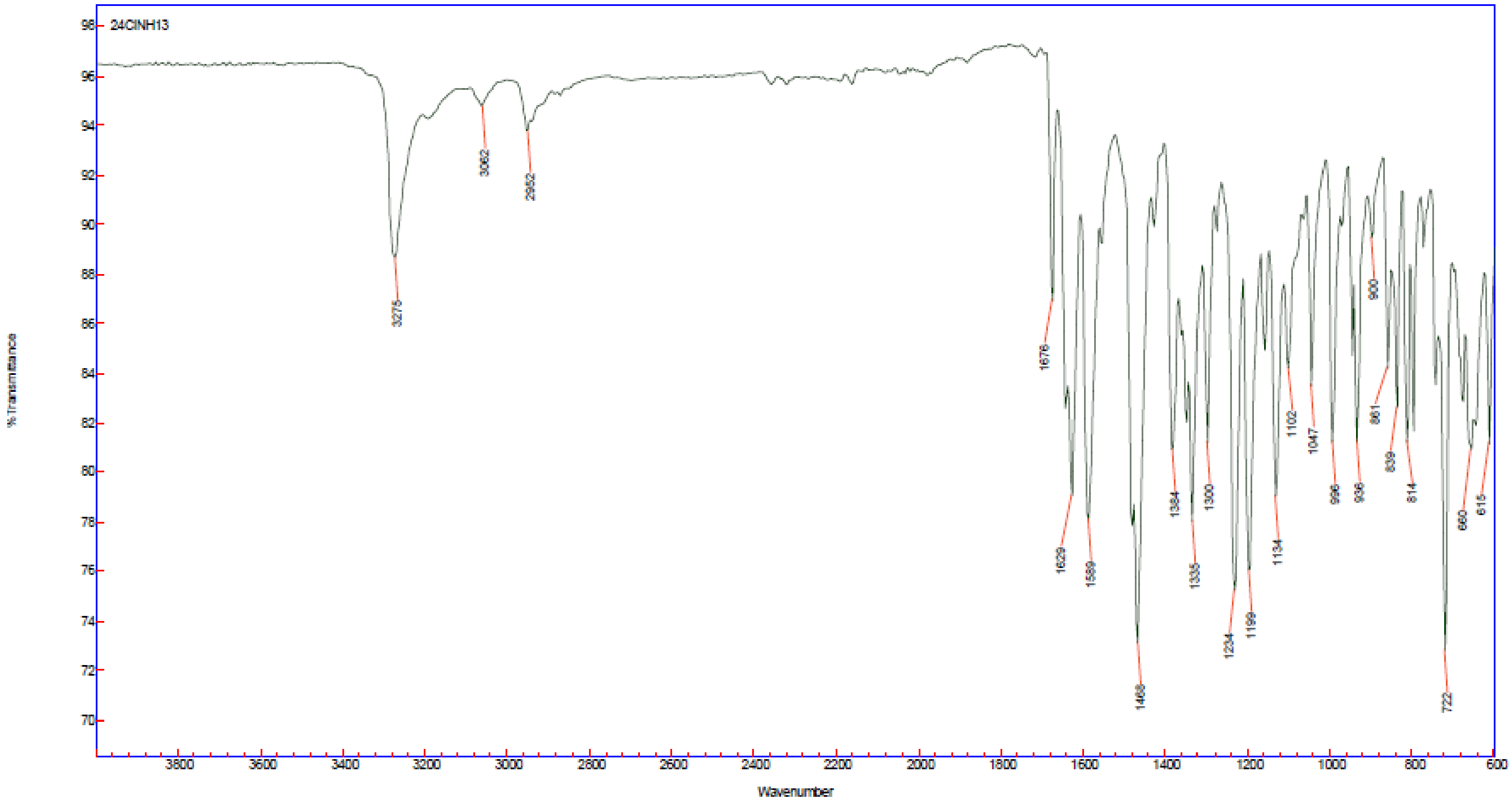

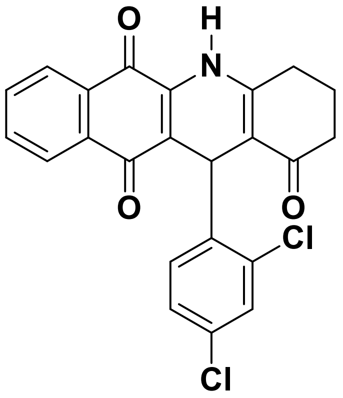

5e

IR spectrum of compound **5e**

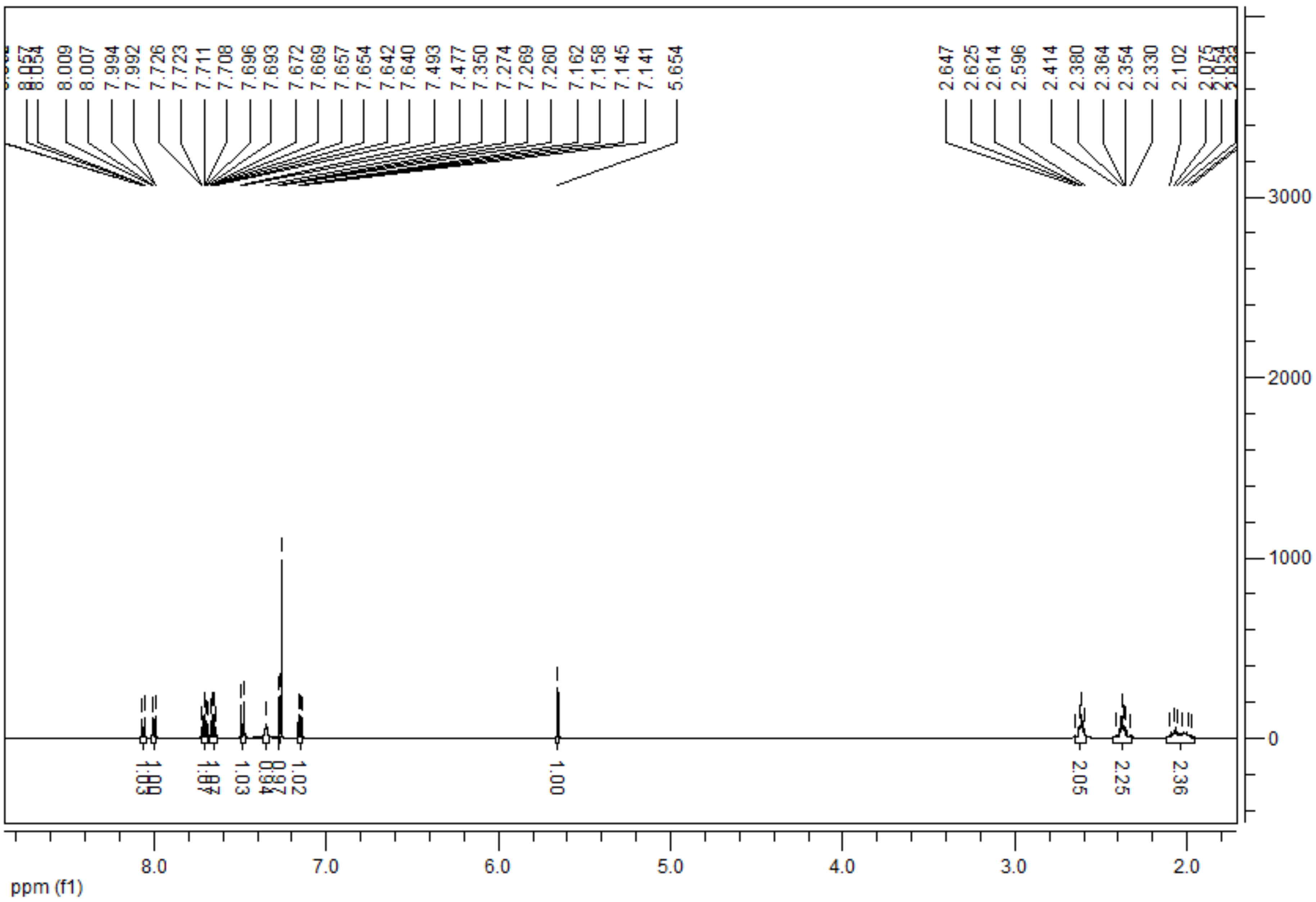

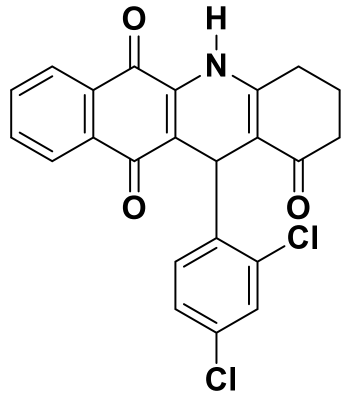

5e

^1H NMR spectrum of compound **5e** (500.00 MHz, $CDCl_3$)

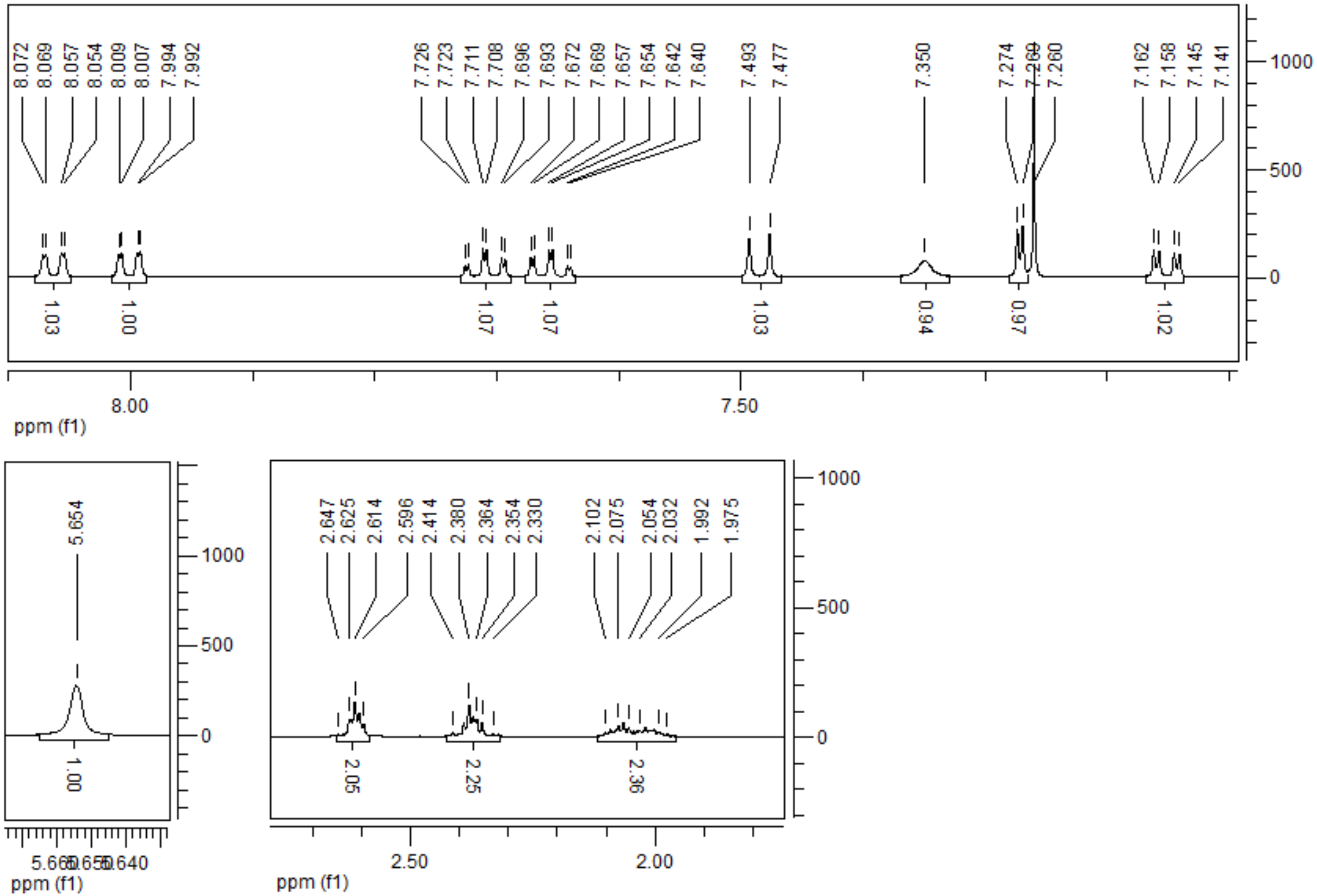

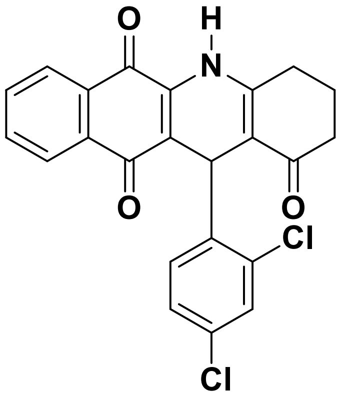

5e

^{1}H NMR spectrum of compound **5e** (500.00 MHz, $CDCl_3$)

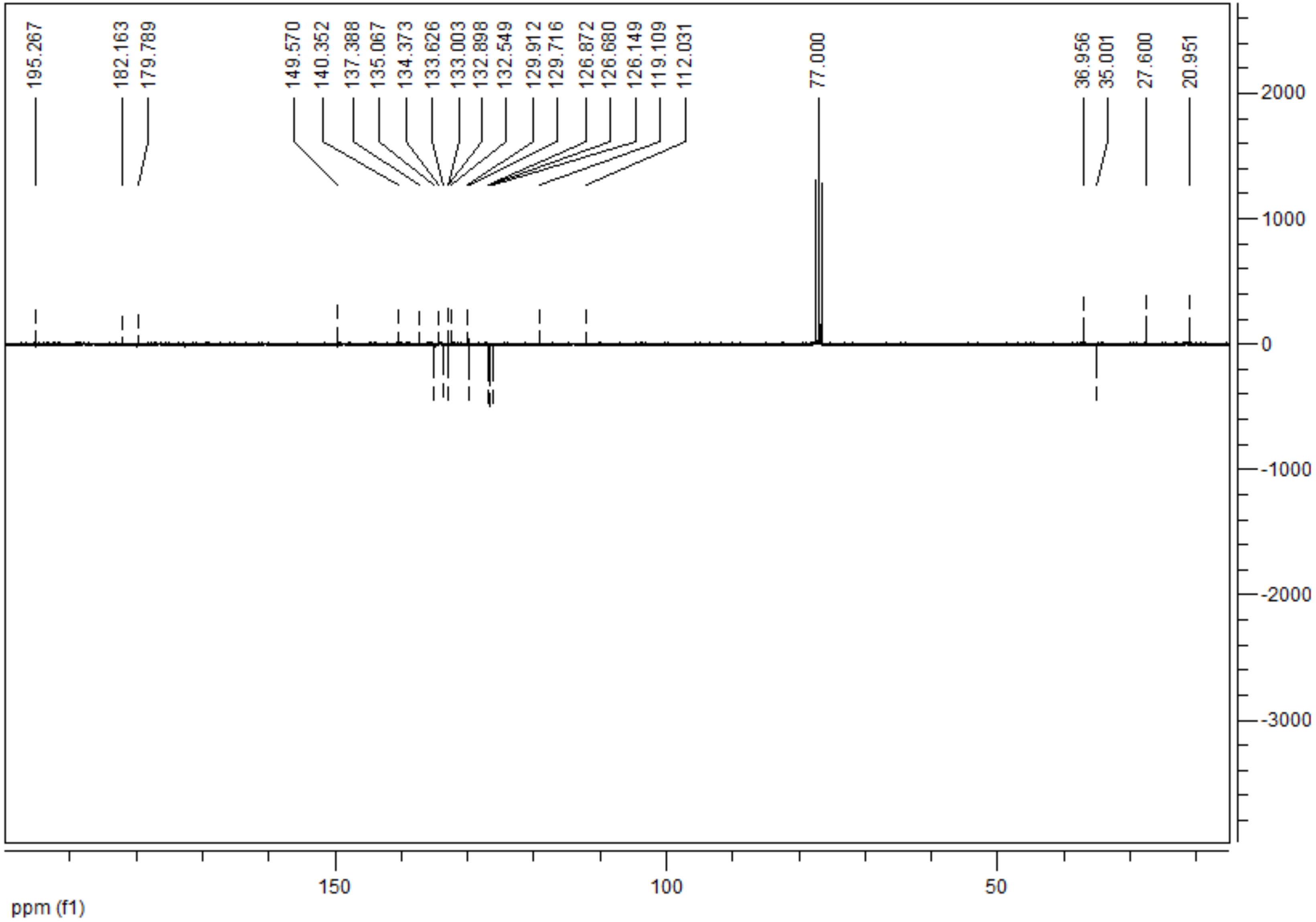

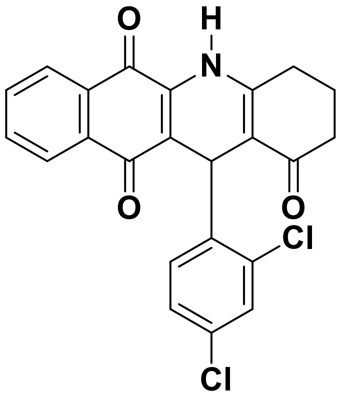

5e

^{13}C NMR spectrum of compound **5e** (75.0 MHz, $CDCl_3$)

+MS, 0.3-0.8min #16-46

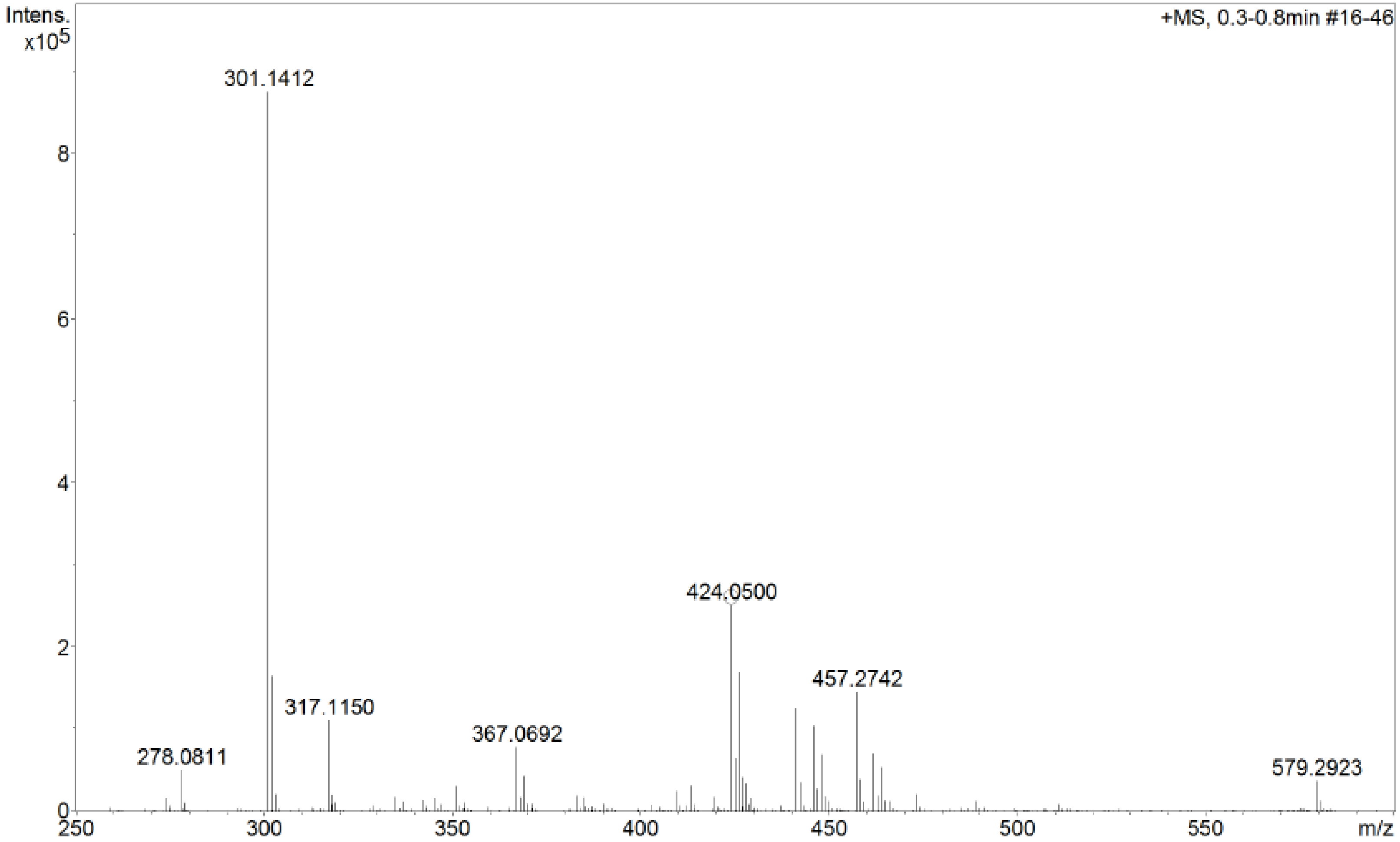

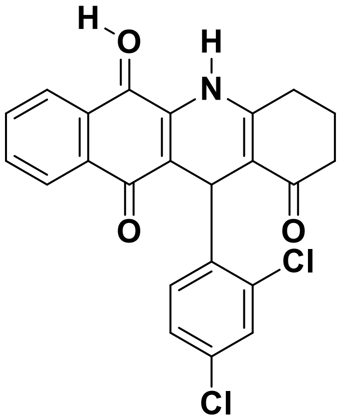

Exact Mass: 424,0507

5e

Meas. m/z	#	Ion Formula	m/z	err [ppm]	Mean err [ppm]	rdb	N-Rule	e⁻ Conf	mSigma	Std I	Std Mean m/z	Std I VarNorm	Std m/z Diff	Std Comb Dev
424.049984	1	C23H16Cl2NO3	424.050175	0.5	0.8	15.5	ok	even	4.2	4.6	n.a.	n.a.	n.a.	n.a.
	2	C19H12Cl2N7O	424.047490	-5.9	-6.4	16.5	ok	even	12.0	16.3	n.a.	n.a.	n.a.	n.a.
	3	C18H16Cl2N3O5	424.046152	-9.0	-9.1	11.5	ok	even	22.1	29.4	n.a.	n.a.	n.a.	n.a.

HRMS of compound **5e**

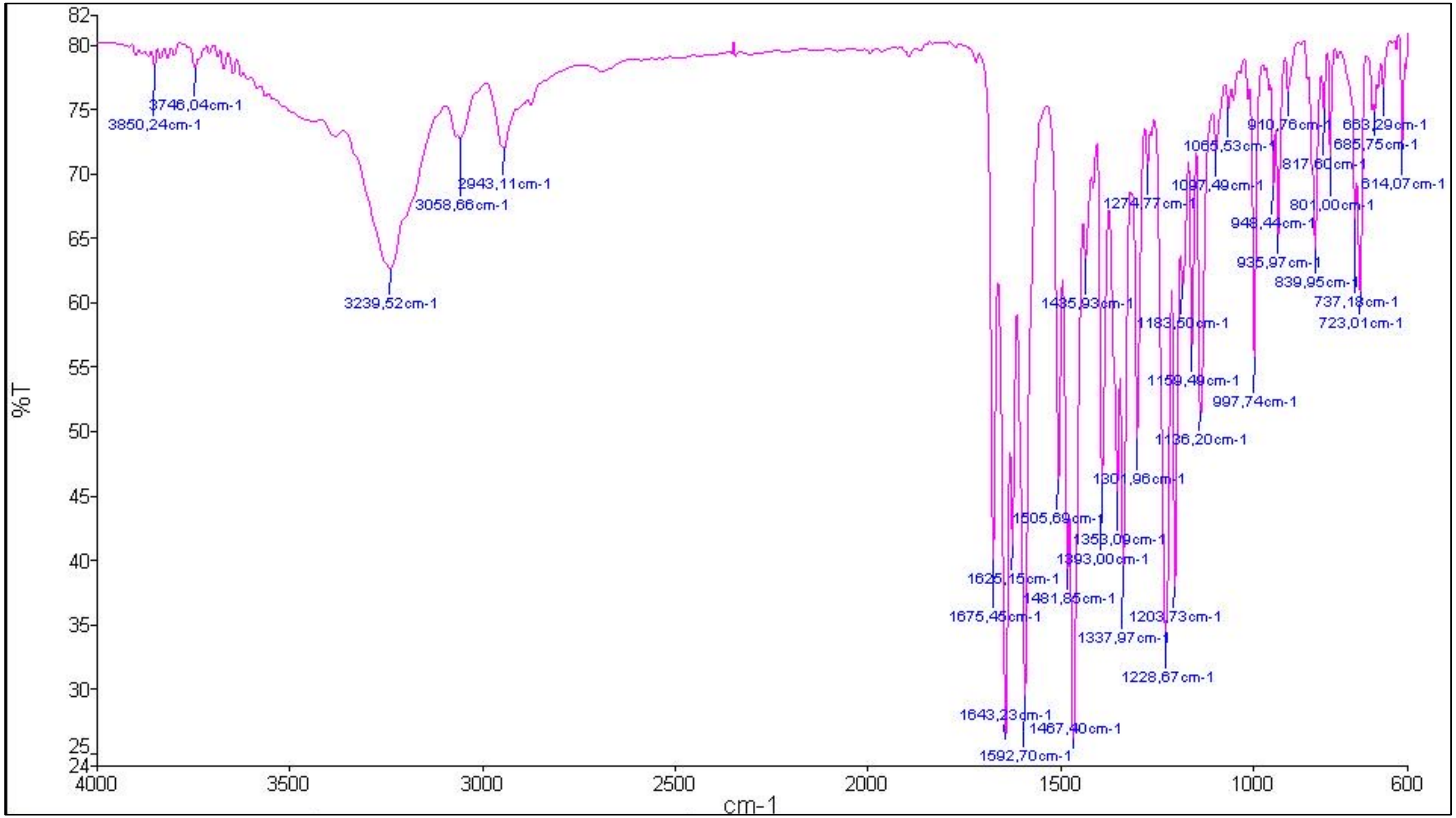

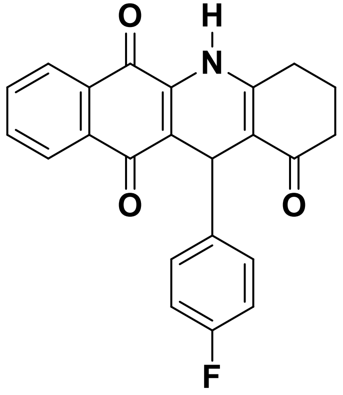

5f

IR spectrum of compound **5f**

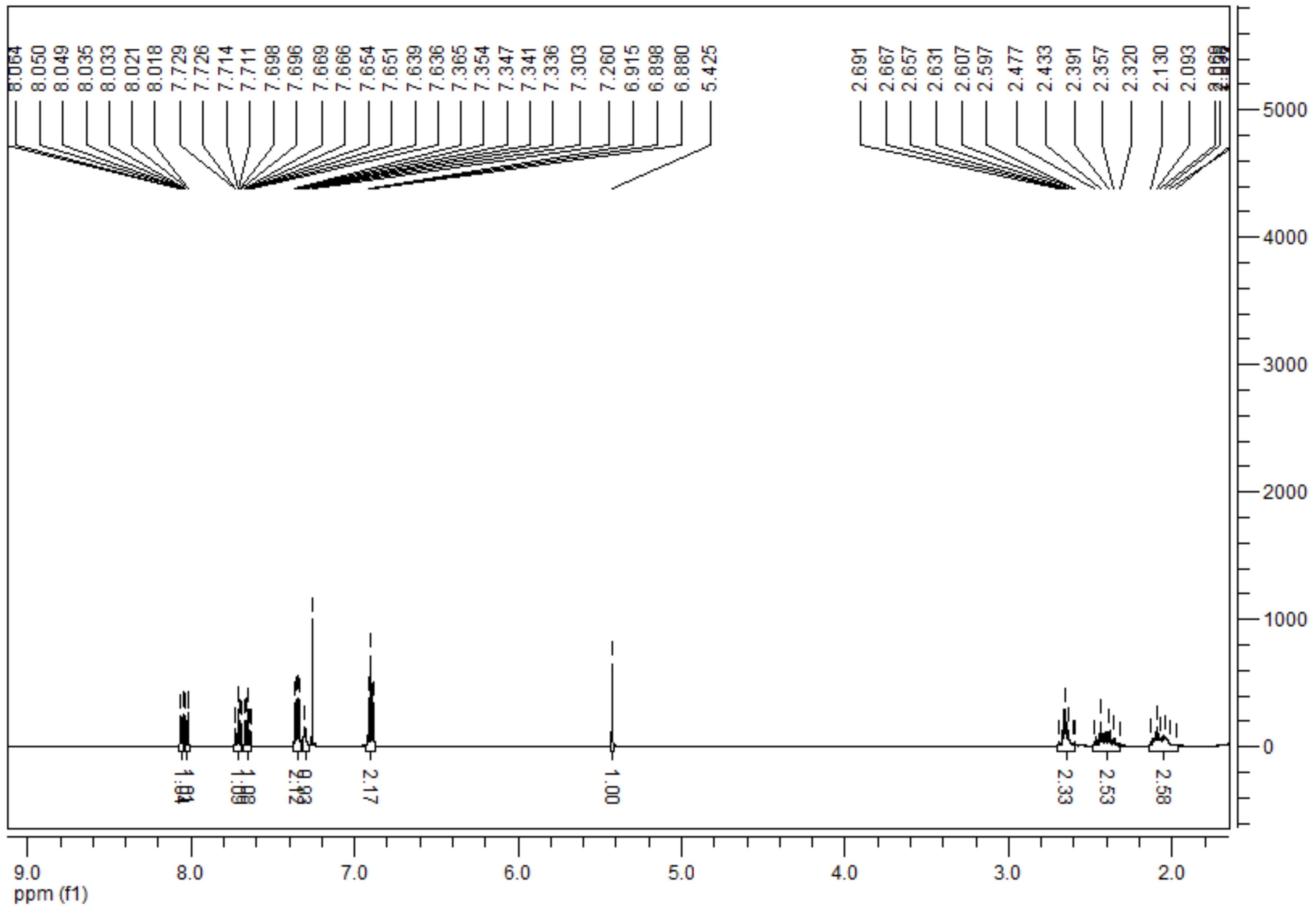

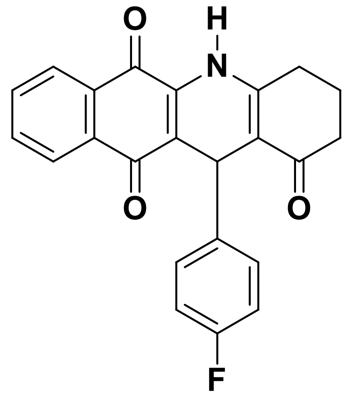

5f

^{1}H NMR spectrum of compound **5f** (500.00 MHz, $CDCl_3$)

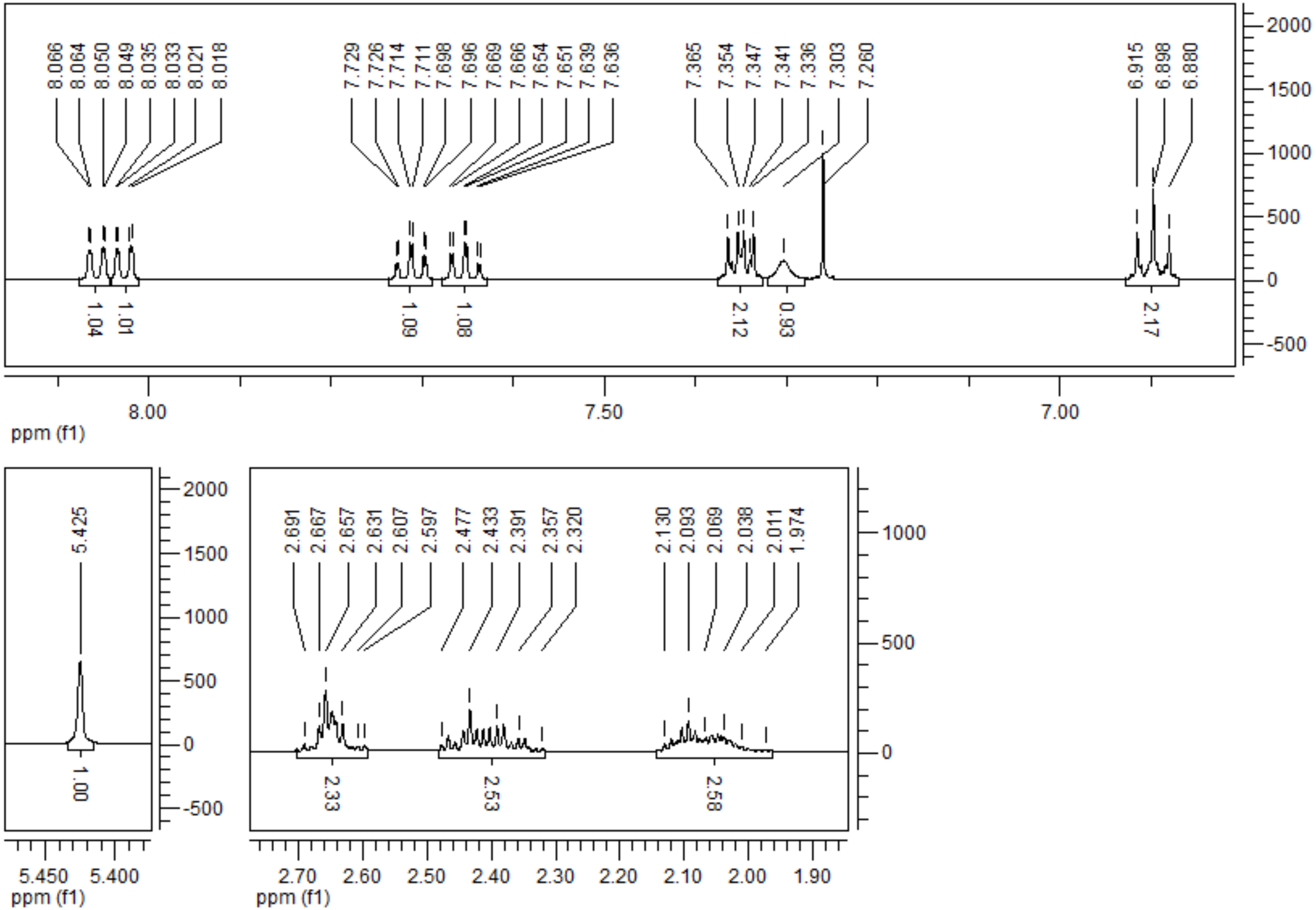

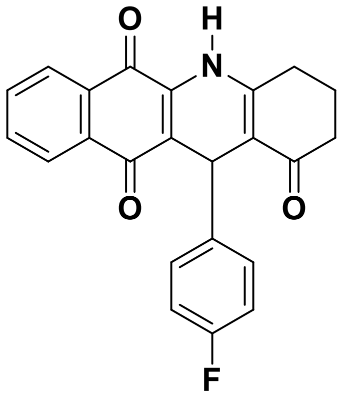

5f

^{1}H NMR spectrum of compound **5f** (500.00 MHz, $CDCl_3$)

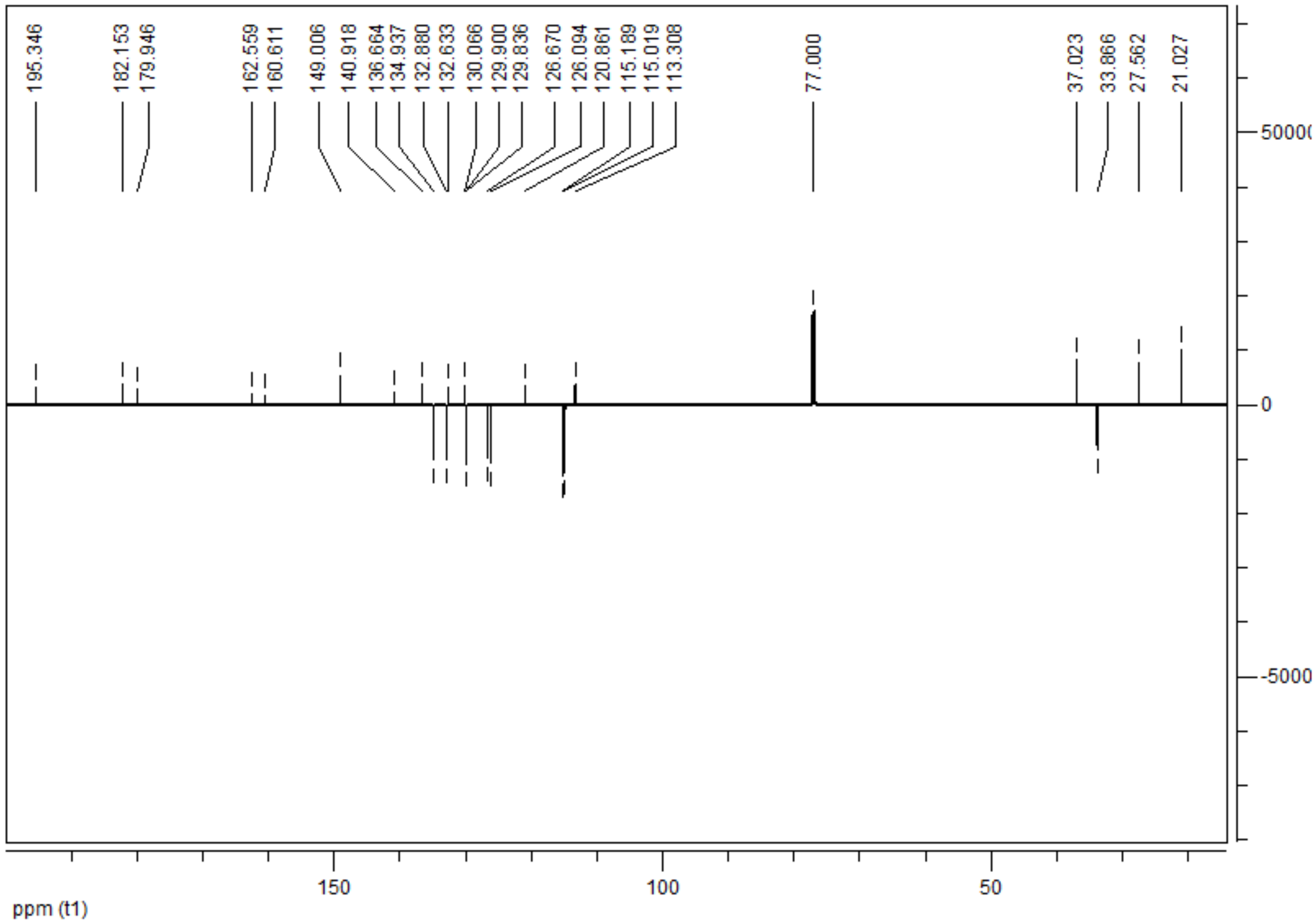

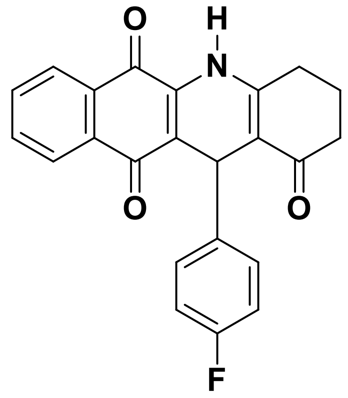

5f

^{13}C NMR spectrum of compound **5f** (75.0 MHz, $CDCl_3$)

+MS, 0.2-0.7min #11-42

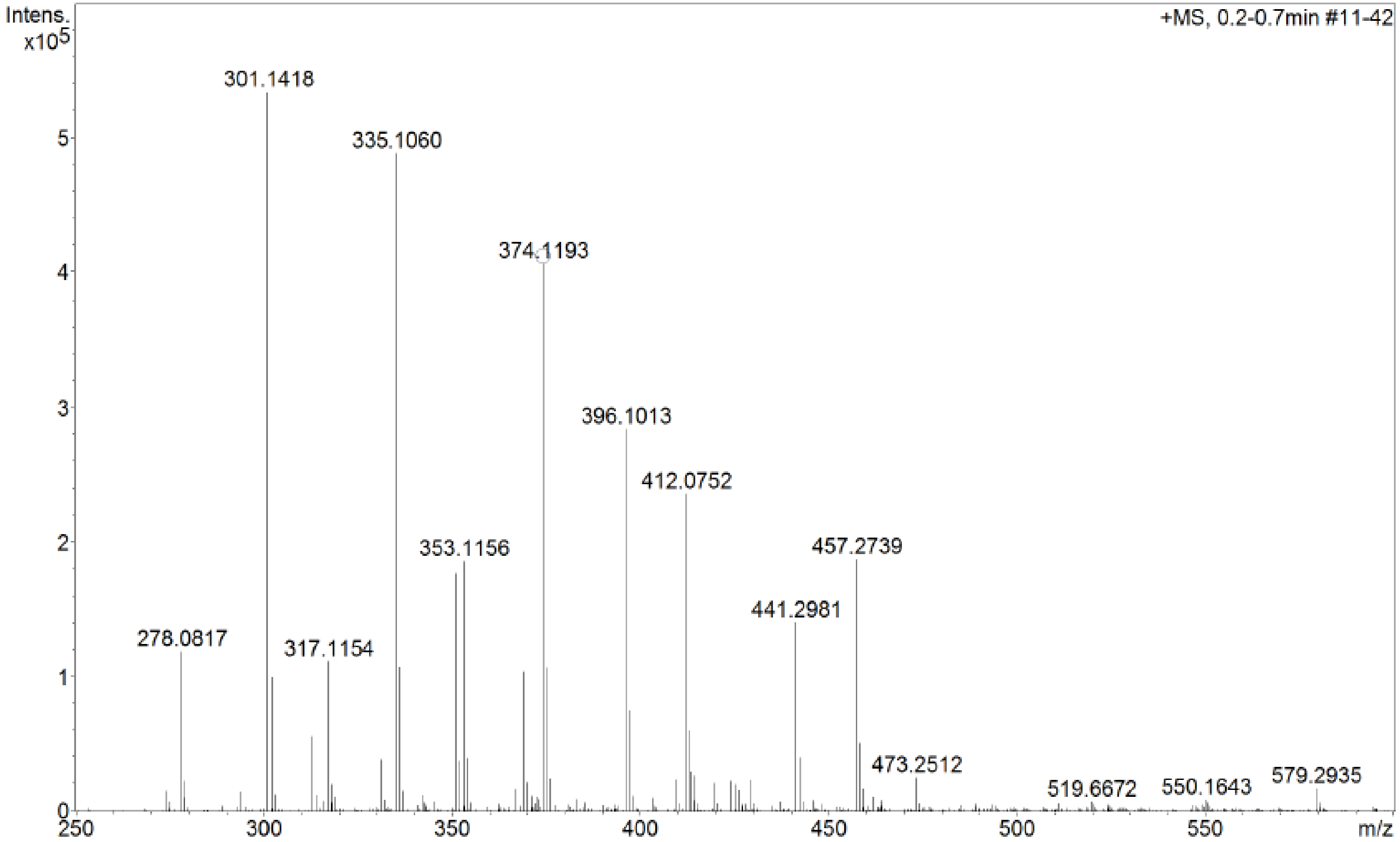

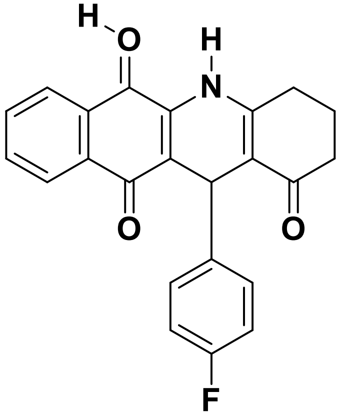

Exact Mass: 374,1192

5f

Meas. m/z	#	Ion Formula	m/z	err [ppm]	Mean err [ppm]	rdb	N-Rule	e⁻ Conf	mSigma	Std I	Std Mean m/z	Std I VarNorm	Std m/z Diff	Std Comb Dev
374.119336	1	C23H17FNO3	374.118698	-1.7	-2.8	15.5	ok	even	13.8	24.2	n.a.	n.a.	n.a.	n.a.
	2	C19H13FN7O	374.116013	-8.9	-10.9	16.5	ok	even	25.3	46.0	n.a.	n.a.	n.a.	n.a.

HRMS of compound **5f**

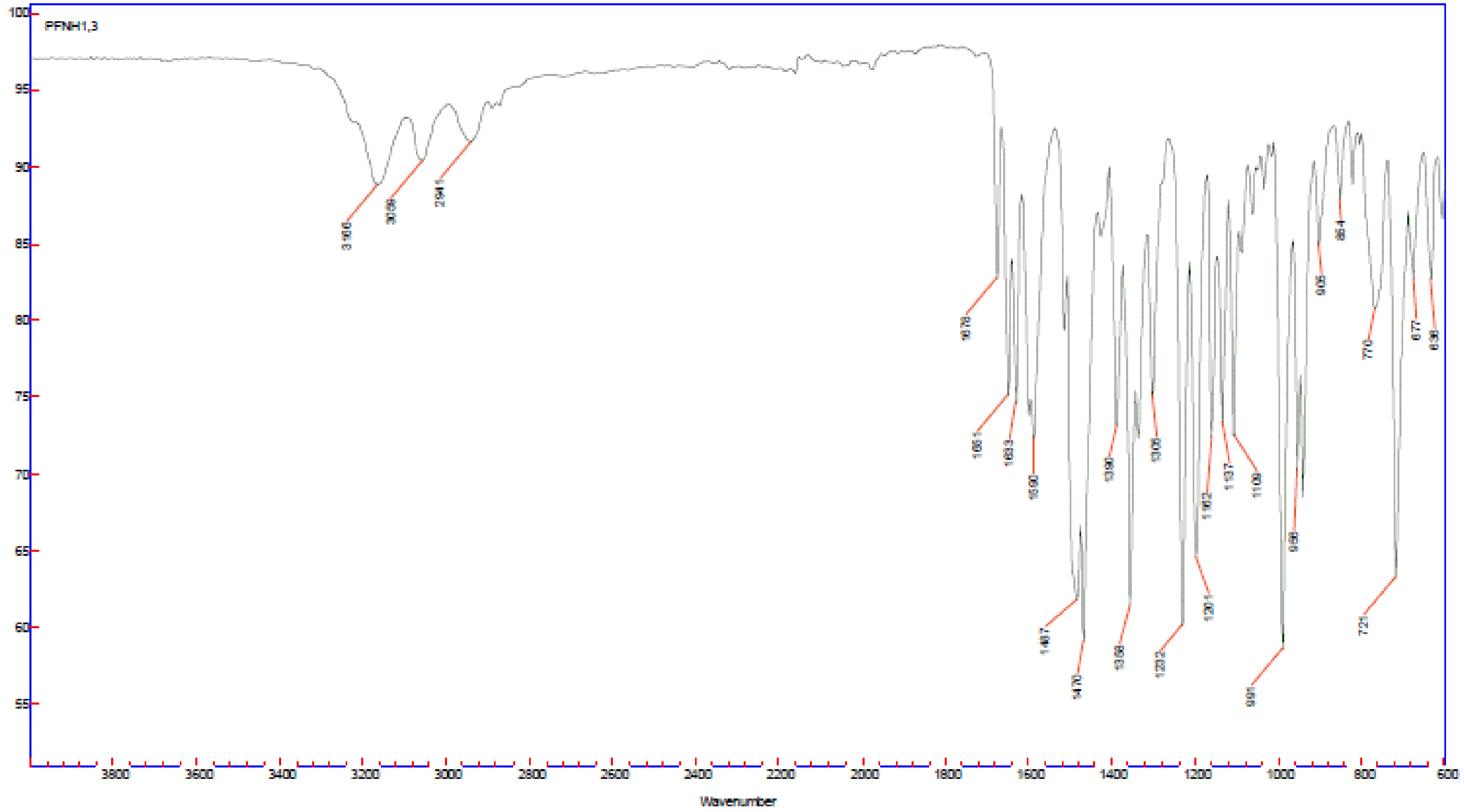

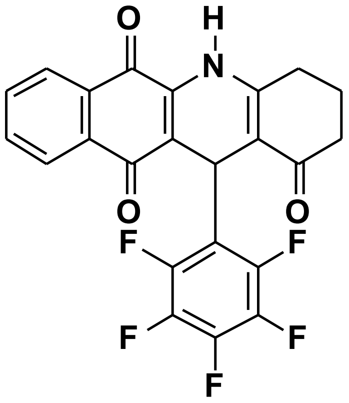
5g

IR spectrum of compound **5g**

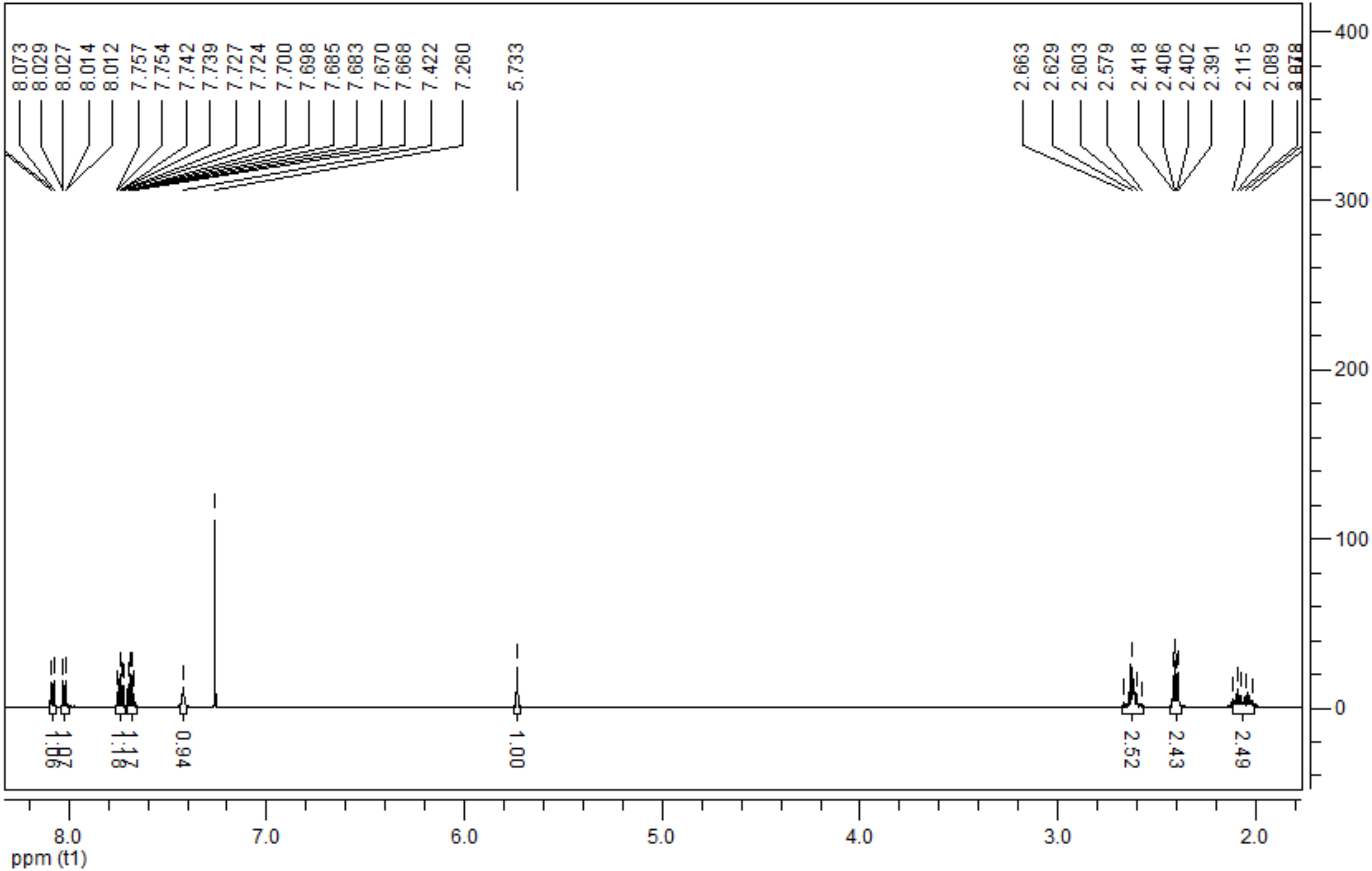

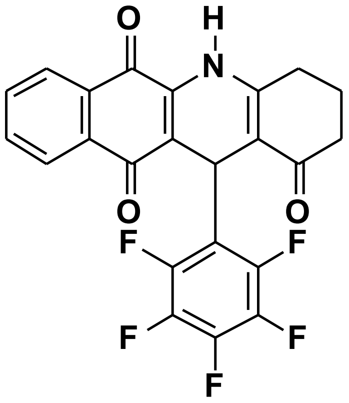

5g

^{1}H NMR spectrum of compound **5g** (500.00 MHz, $CDCl_3$)

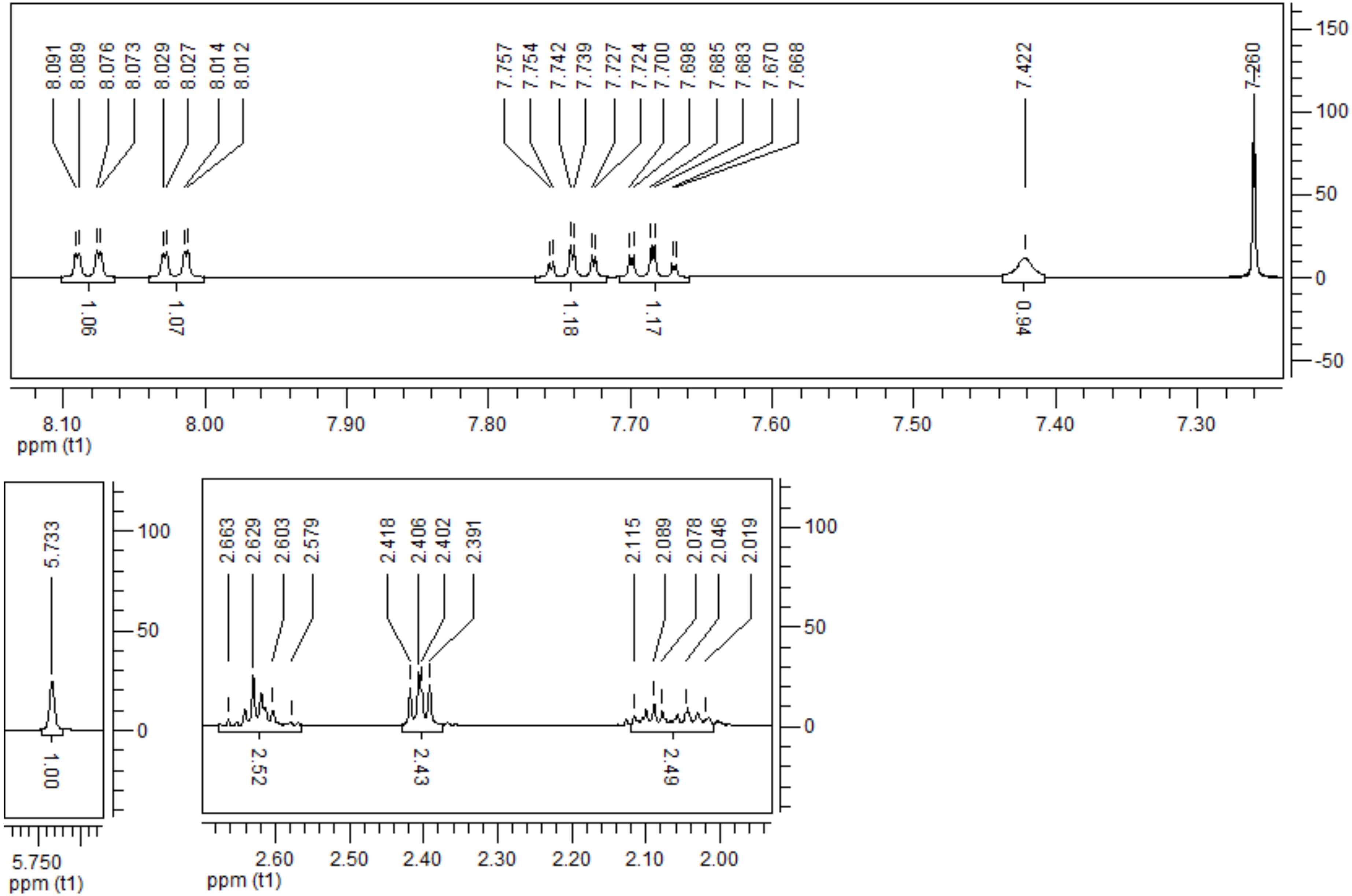

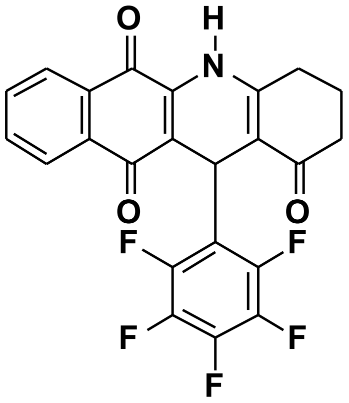

5g

^{1}H NMR spectrum of compound **5g** (500.00 MHz, $CDCl_3$)

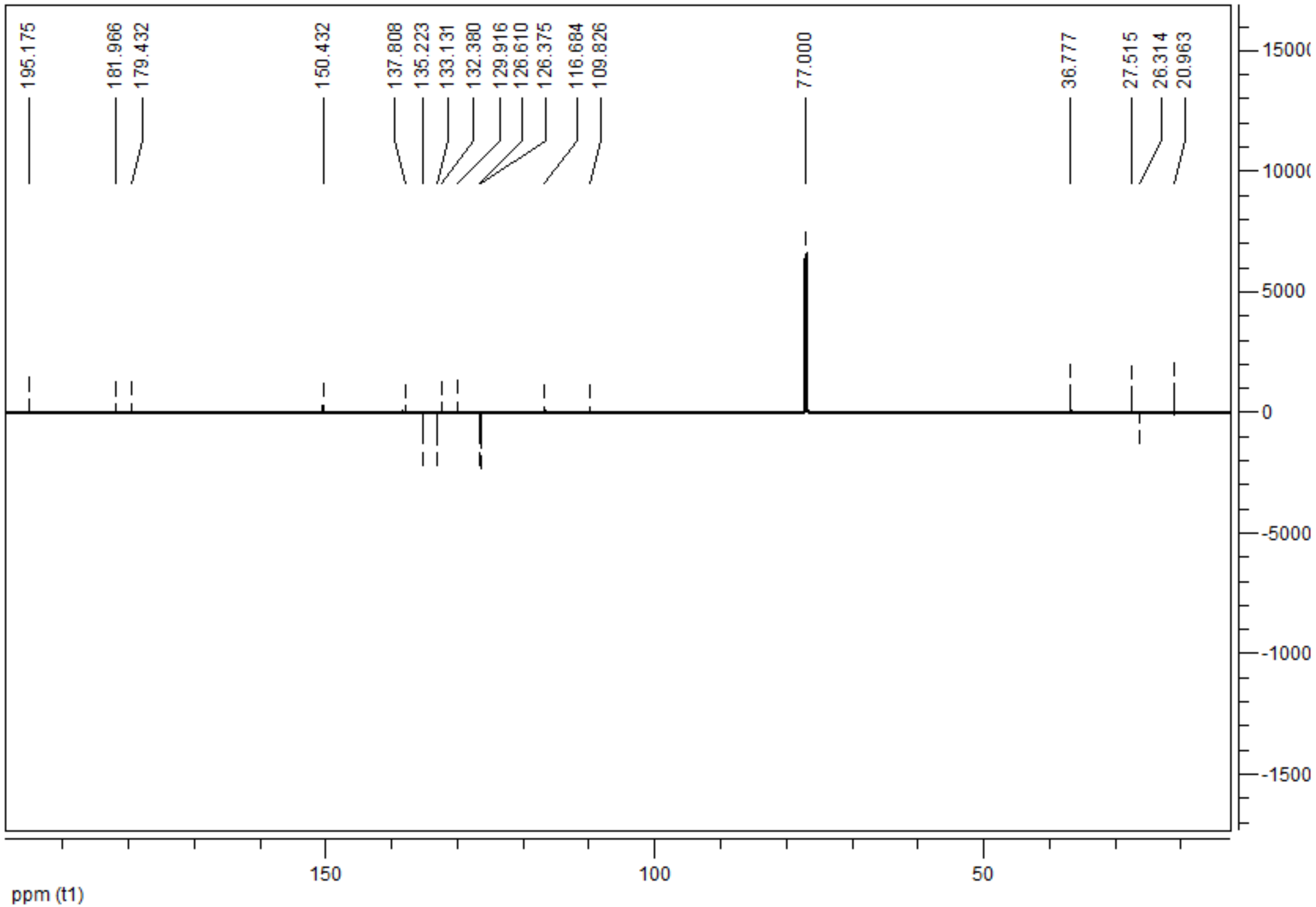

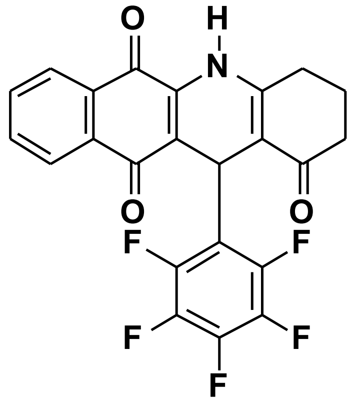

5g

^{13}C NMR spectrum of compound **5g** (125.0 MHz, $CDCl_3$)

+MS, 0.4-0.9min #21-54

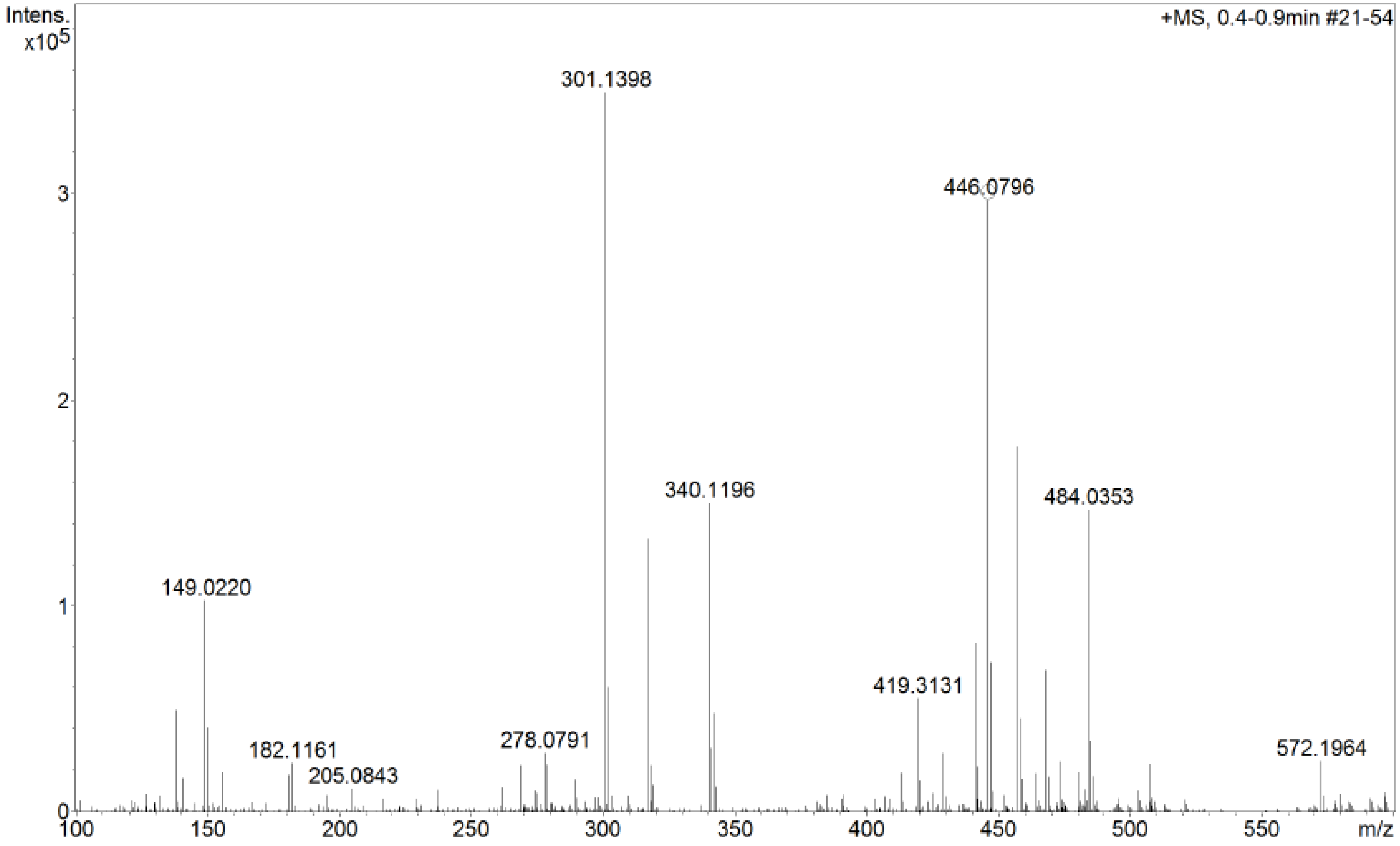

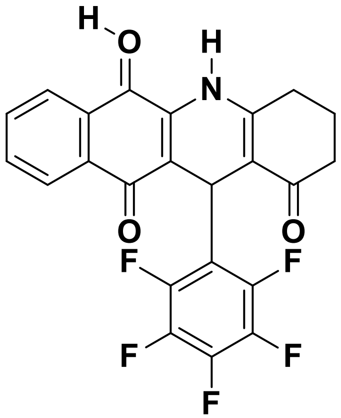

Exact Mass: 446,0816

5g

Meas. m/z	#	Ion Formula	m/z	err [ppm]	Mean err [ppm]	rdb	N-Rule	e⁻ Conf	mSigma	Std I	Std Mean m/z	Std I VarNorm	Std m/z Diff	Std Comb Dev
446.079562	1	C23H13F5NO3	446.081011	3.2	4.4	15.5	ok	even	6.3	10.8	n.a.	n.a.	n.a.	n.a.
	2	C19H9F5N7O	446.078325	-2.8	-2.4	16.5	ok	even	7.8	13.7	n.a.	n.a.	n.a.	n.a.
	3	C18H13F5N3O5	446.076988	-5.8	-5.1	11.5	ok	even	20.8	33.3	n.a.	n.a.	n.a.	n.a.

HRMS of compound **5g**

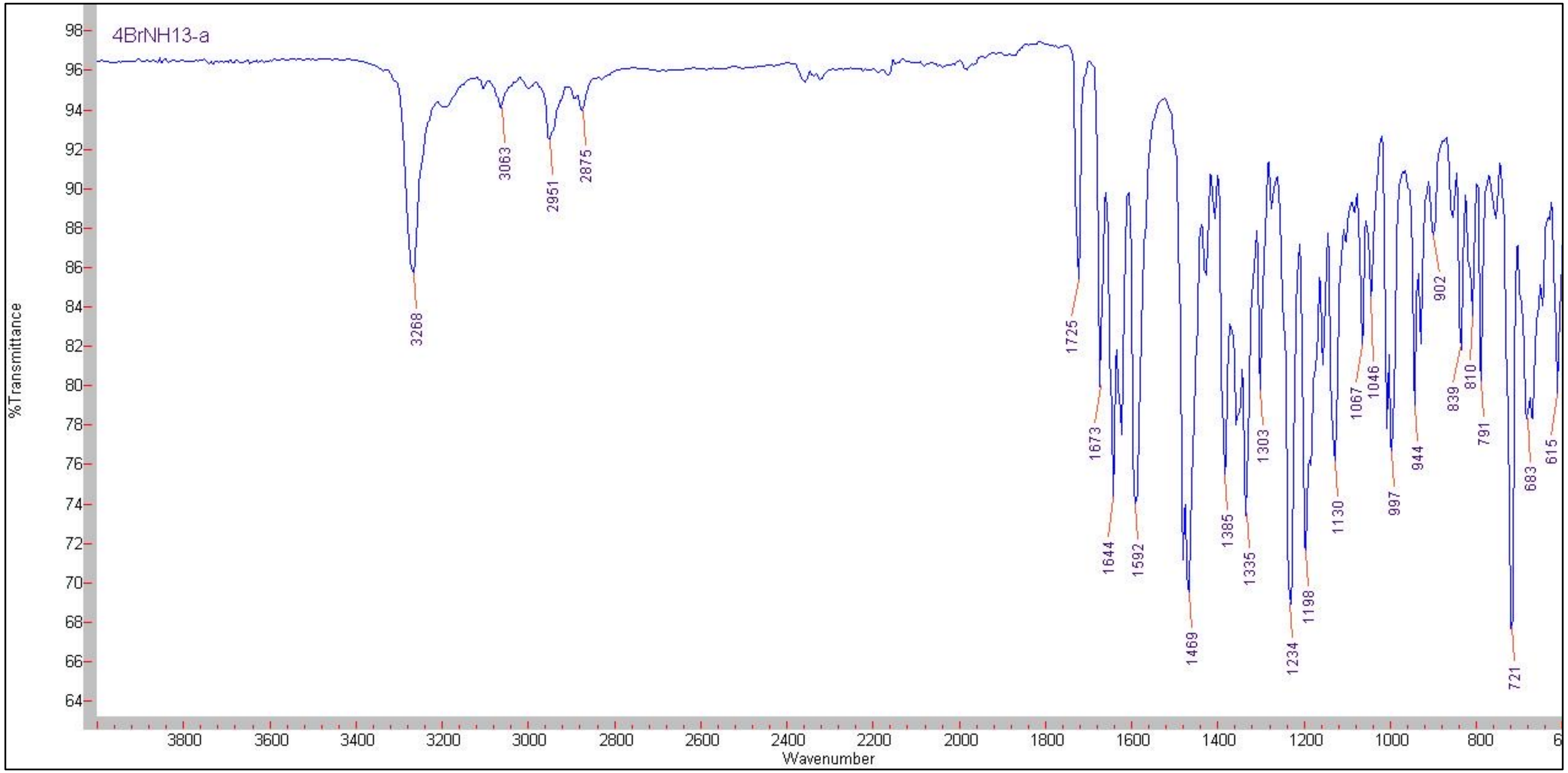

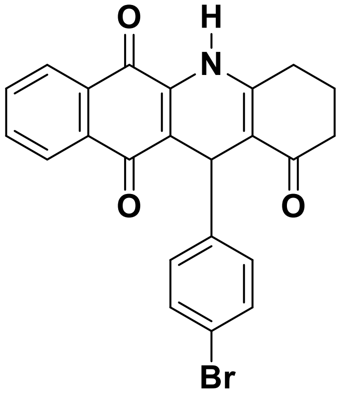

5h

IR spectrum of compound **5h**

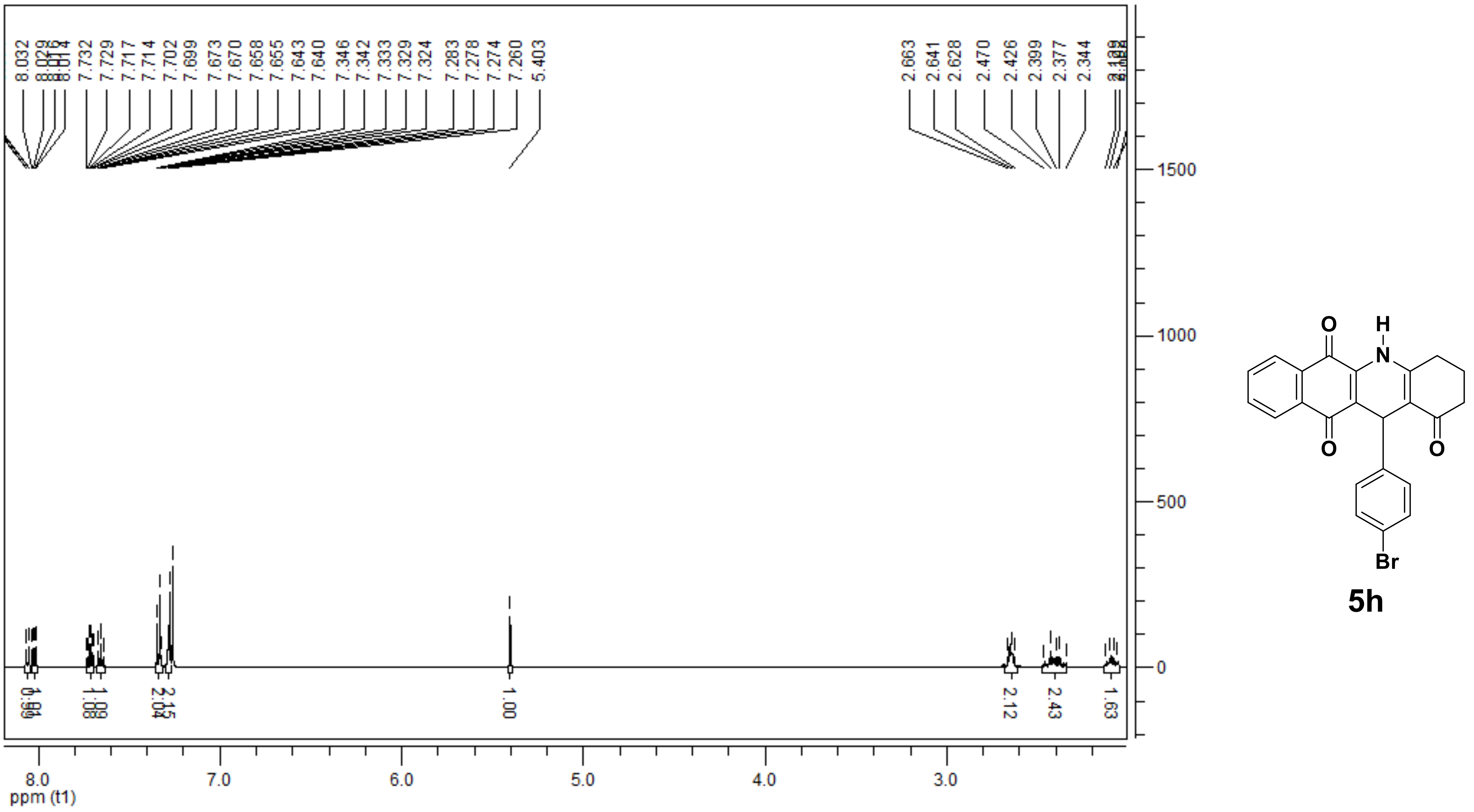

^{1}H NMR spectrum of compound **5h** (500.00 MHz, $CDCl_3$)

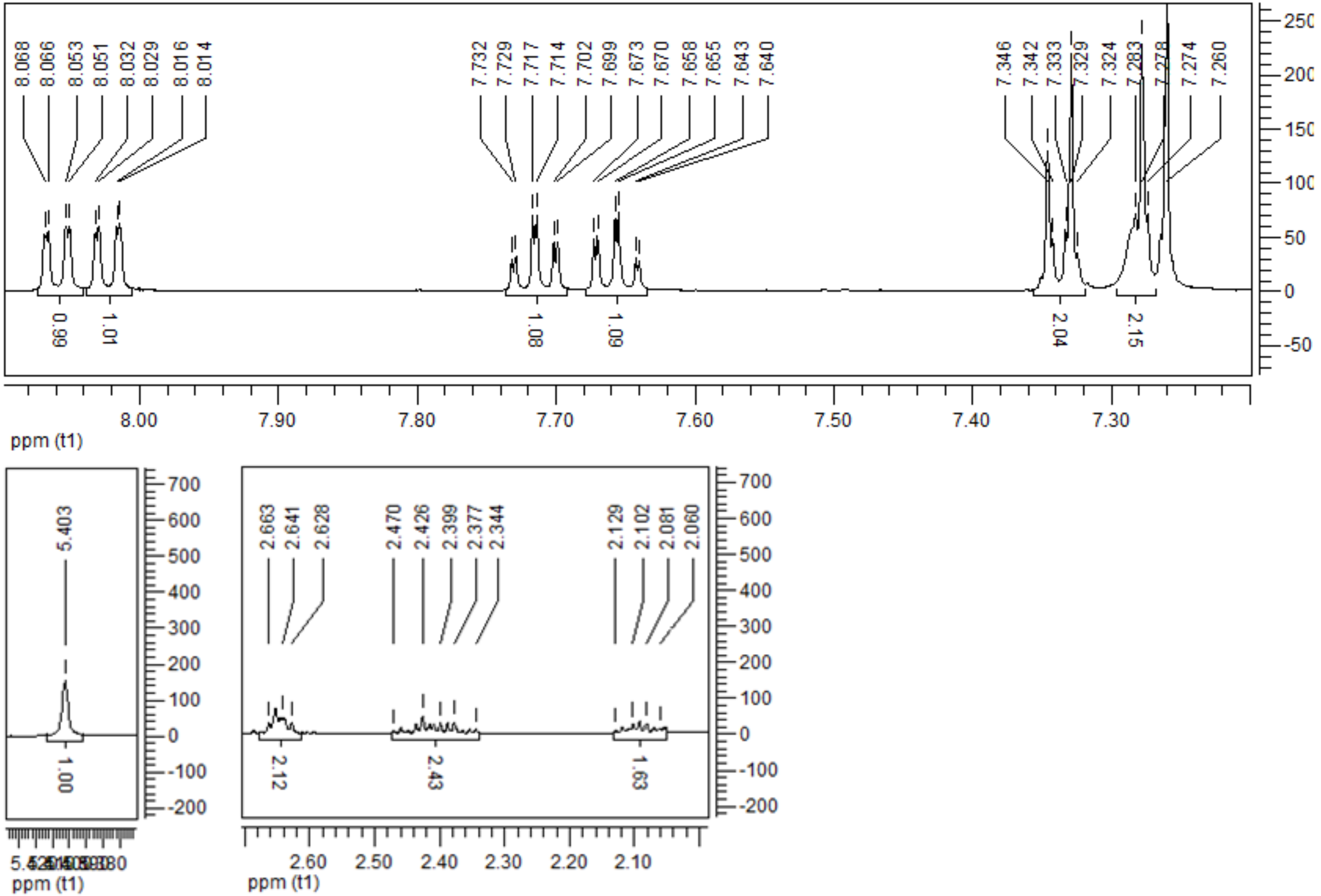

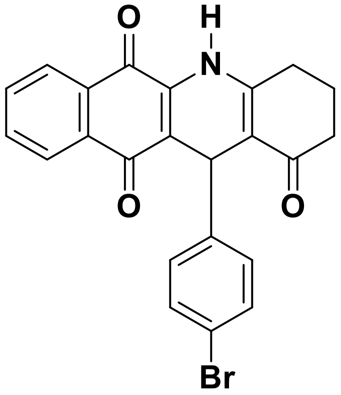

5h

^{1}H NMR spectrum of compound **5h** (500.00 MHz, $CDCl_3$)

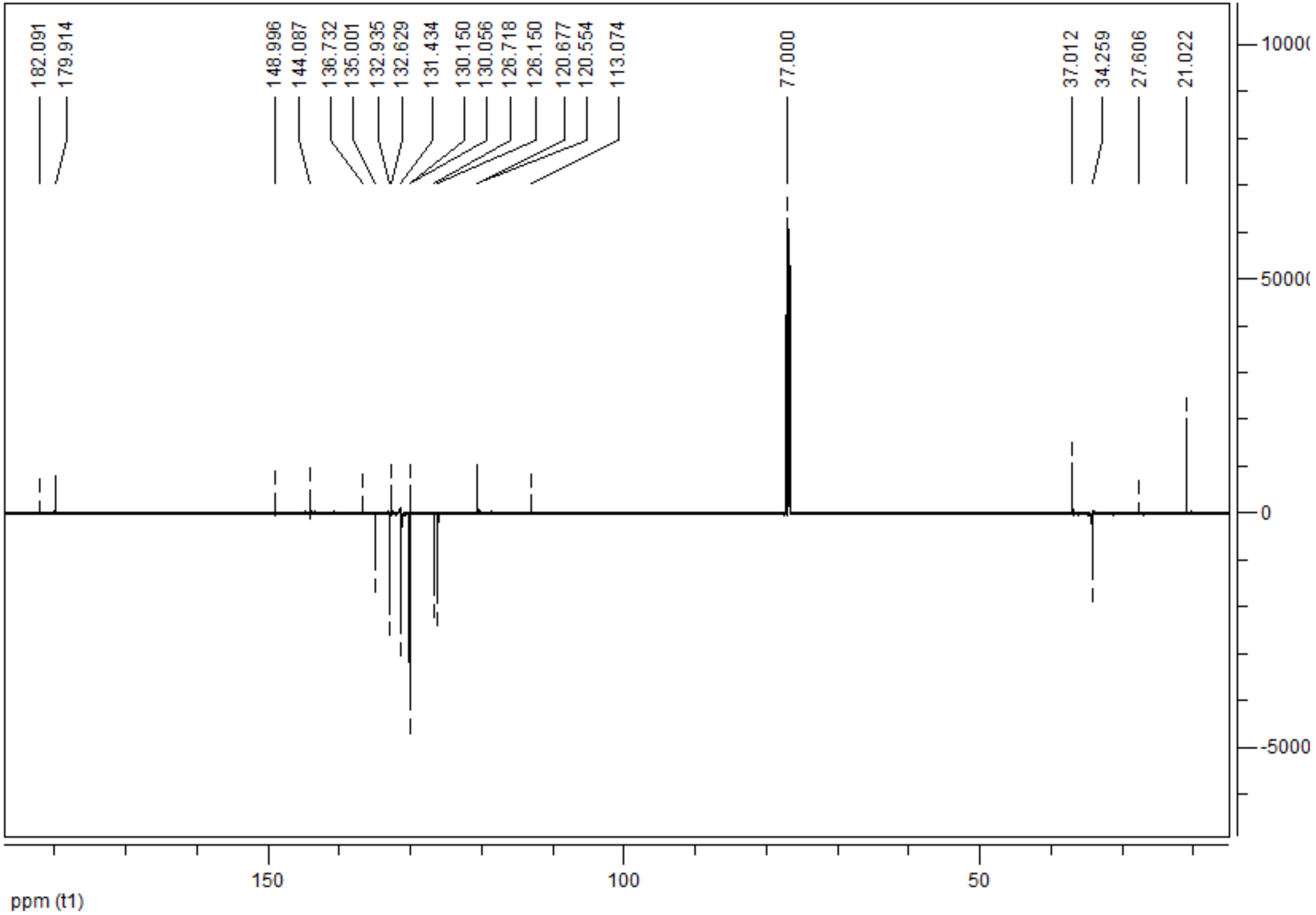

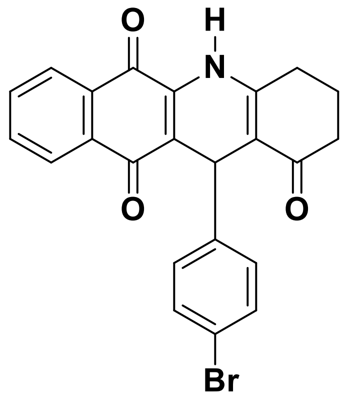

5h

^{13}C NMR spectrum of compound **5h** (125.0 MHz, $CDCl_3$)

+MS, 0.2-0.5min #11-26

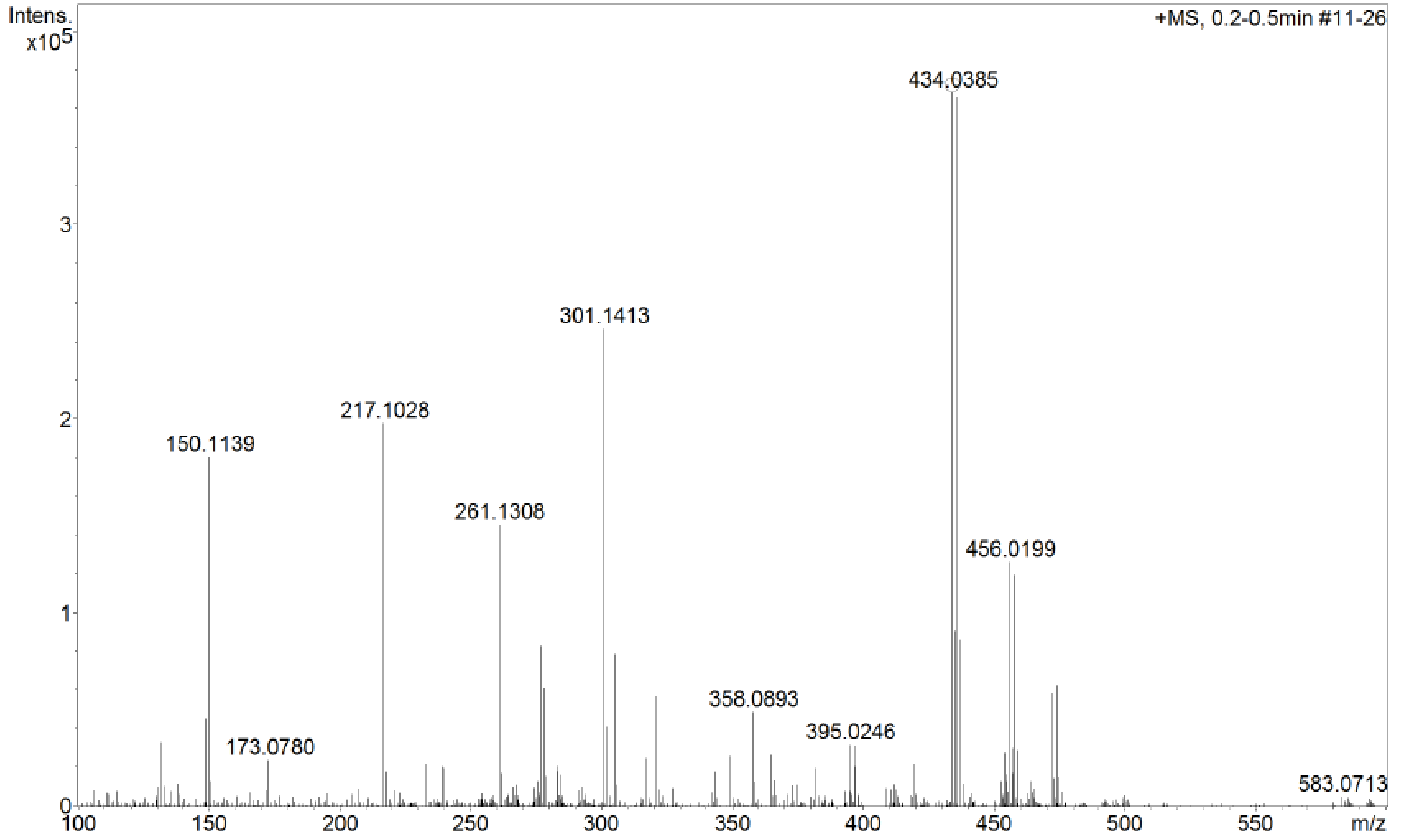

Meas. m/z	#	Ion Formula	m/z	err [ppm]	Mean err [ppm]	rdb	N-Rule	e^- Conf	mSigma	Std I	Std Mean m/z	Std I VarNorm	Std m/z Diff	Std Comb Dev
434.038533	1	C23H17BrNO3	434.038632	0.2	0.7	15.5	ok	even	9.2	9.2	n.a.	n.a.	n.a.	n.a.

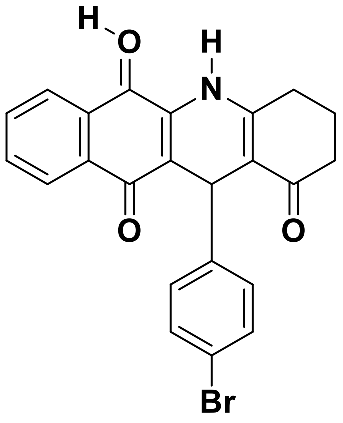

Exact Mass: 434,0392

5h

HRMS of compound **5h**

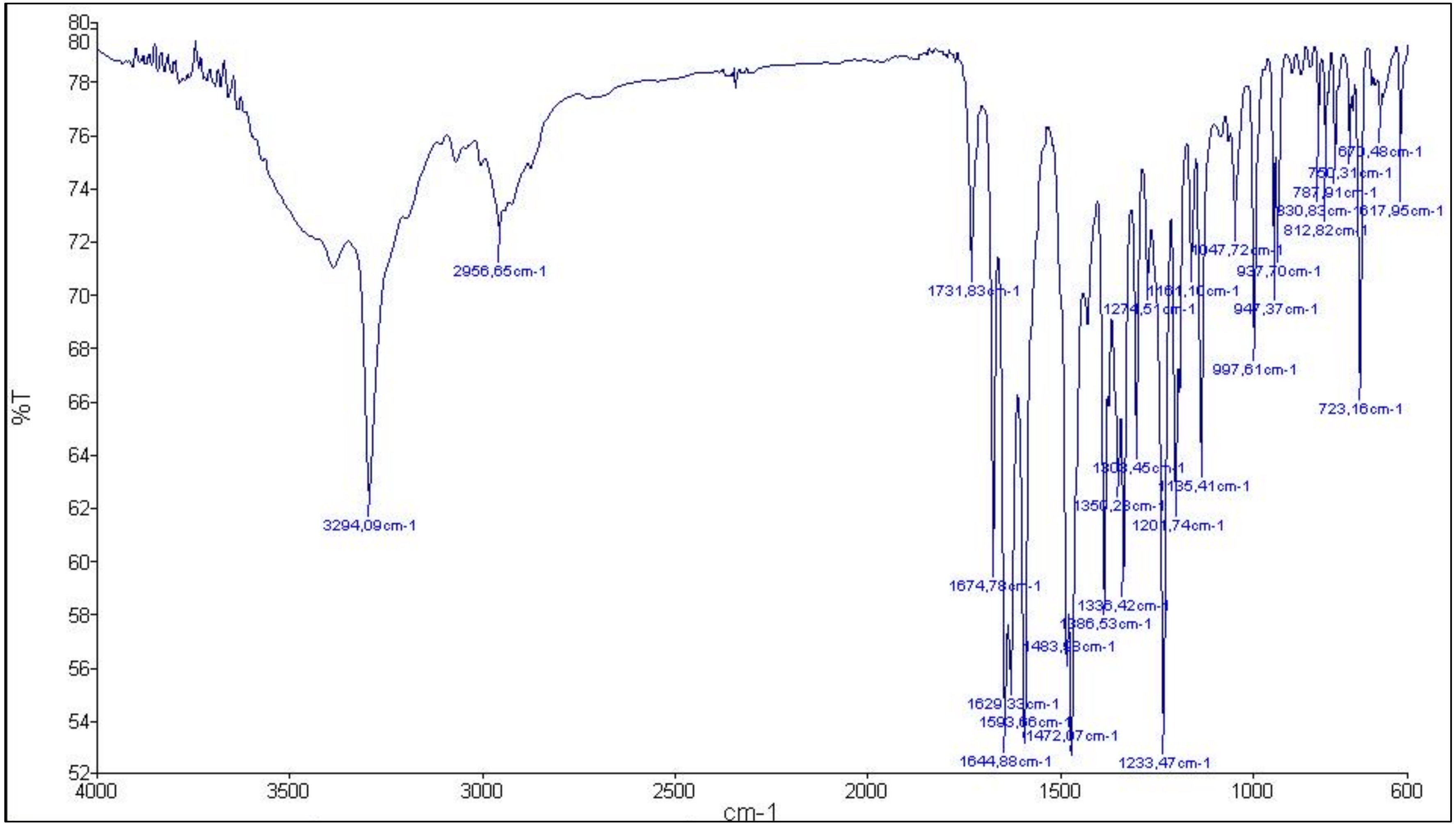

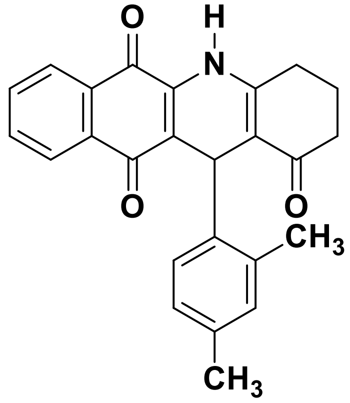

5i

IR spectrum of compound **5i**

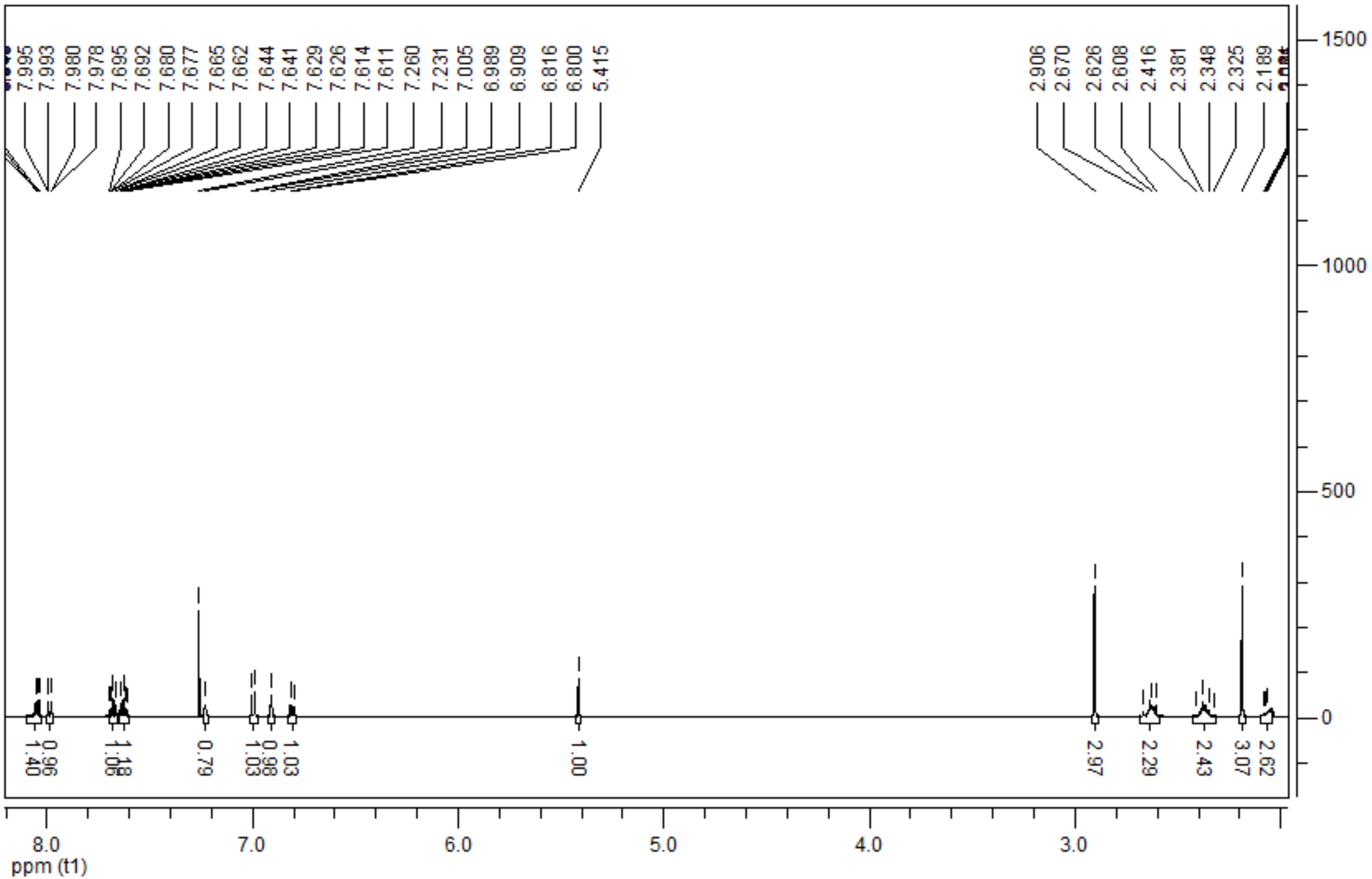

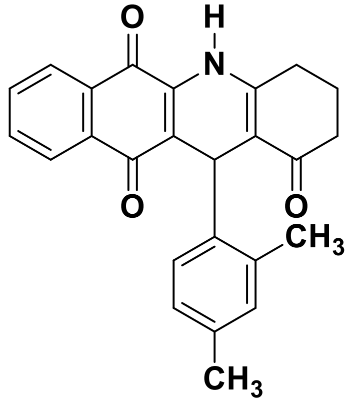

5i

^{1}H NMR spectrum of compound **5i** (500.00 MHz, $CDCl_3$)

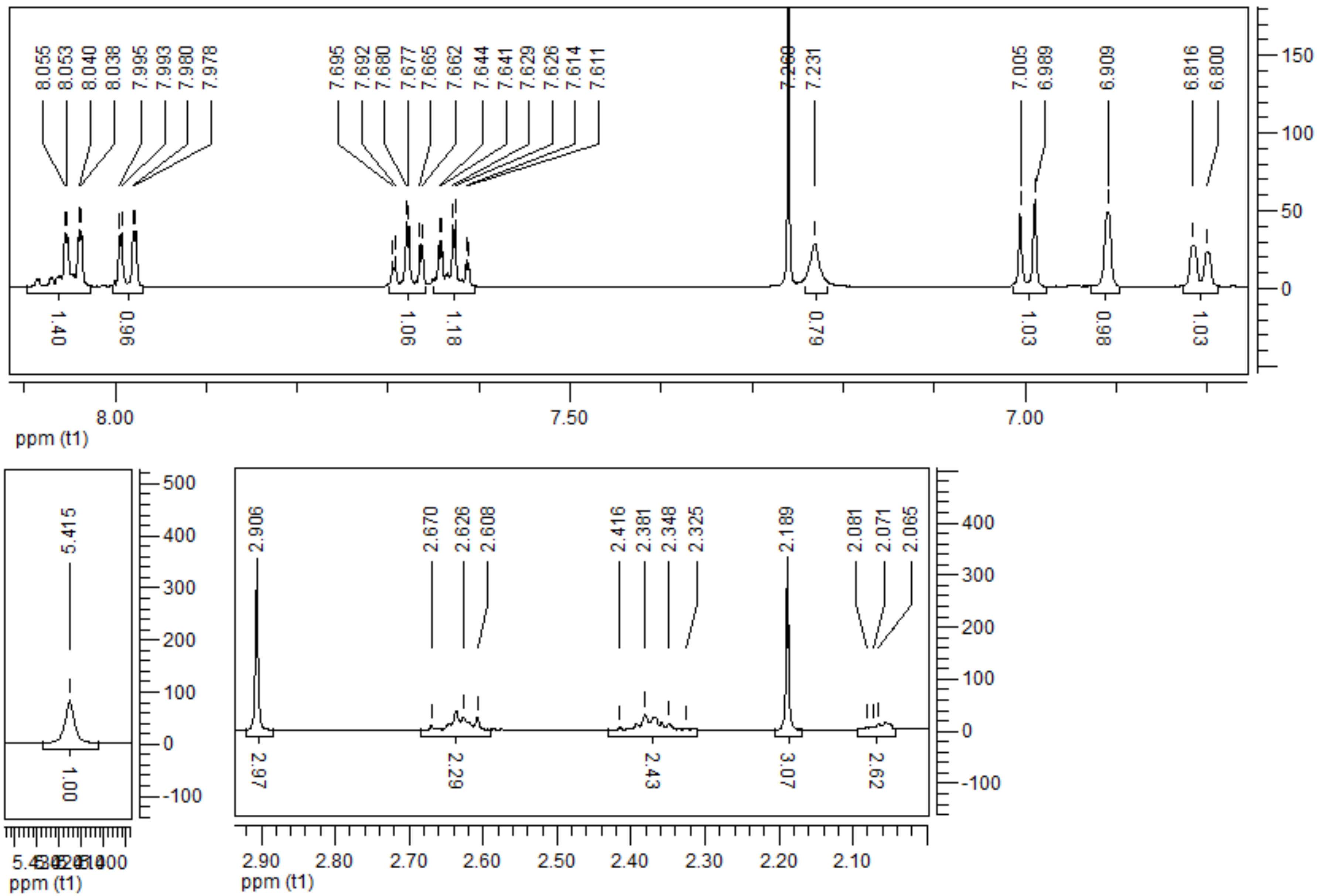

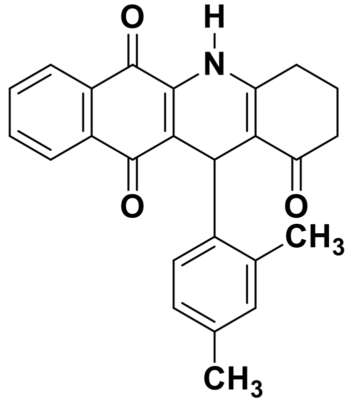

5i

^{1}H NMR spectrum of compound **5i** (500.00 MHz, $CDCl_3$)

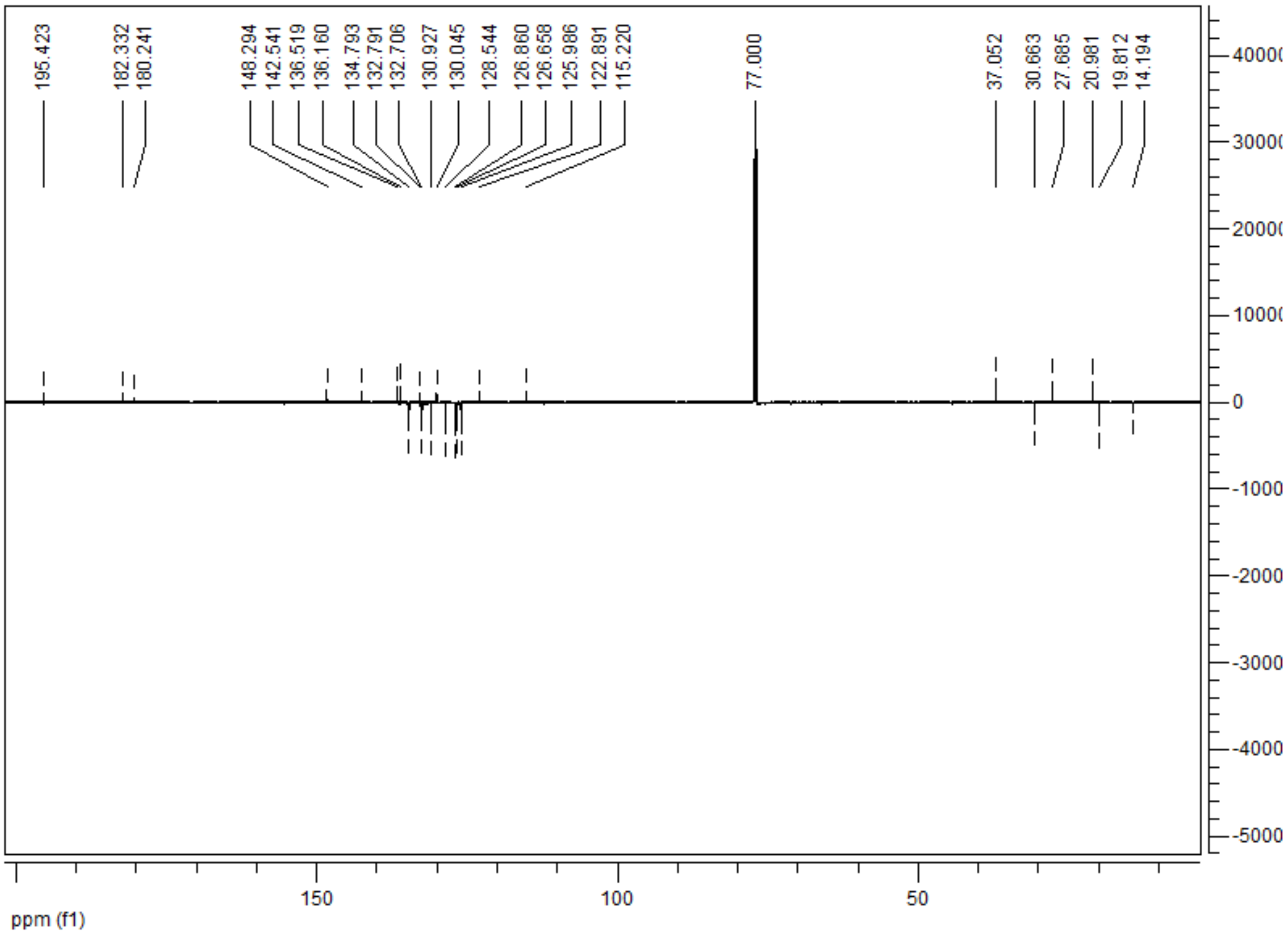

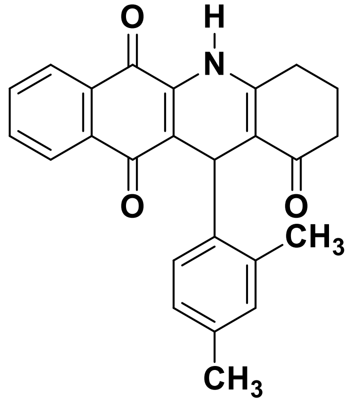

5i

^{13}C NMR spectrum of compound **5i** (75.0 MHz, $CDCl_3$)

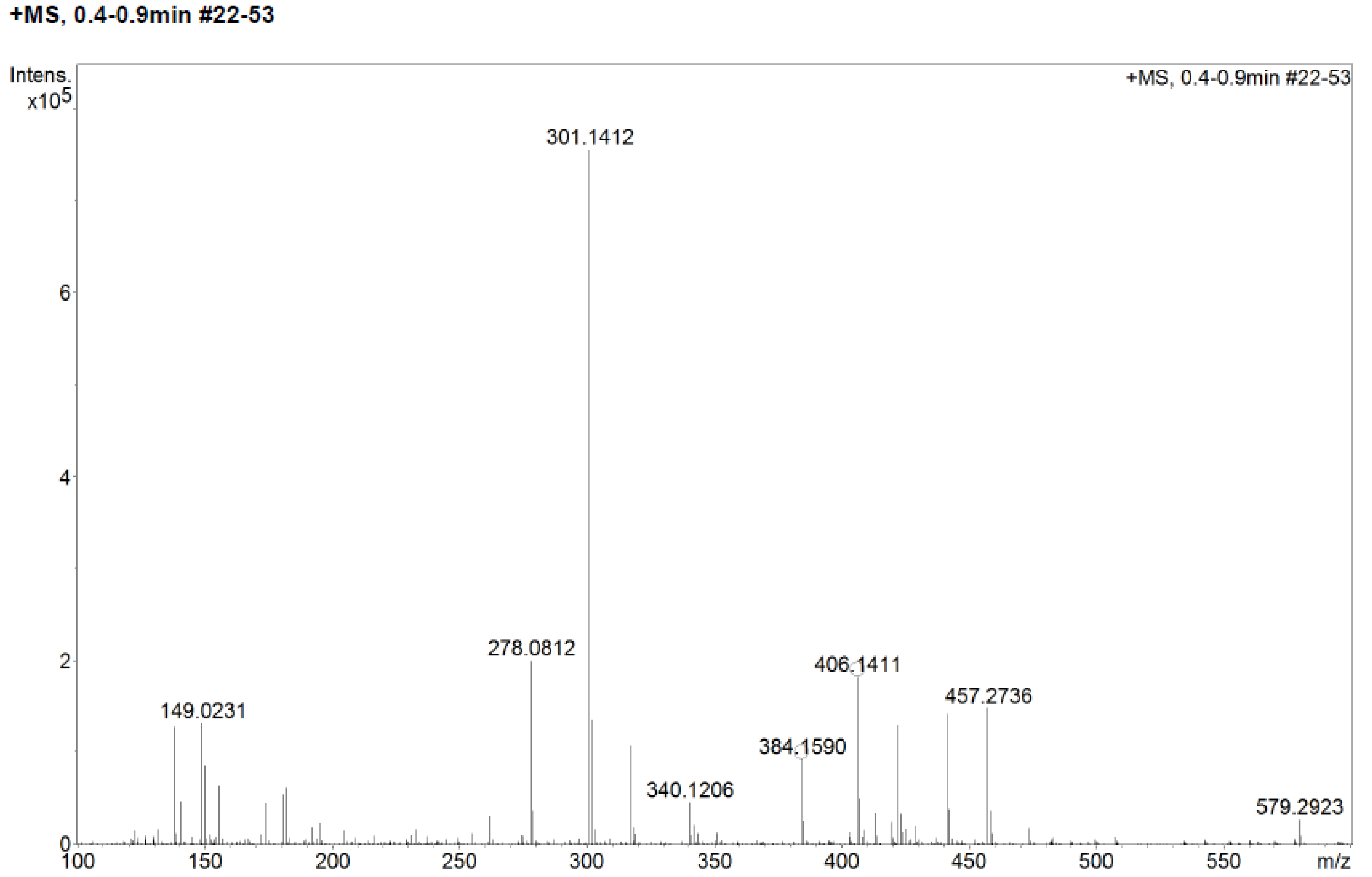

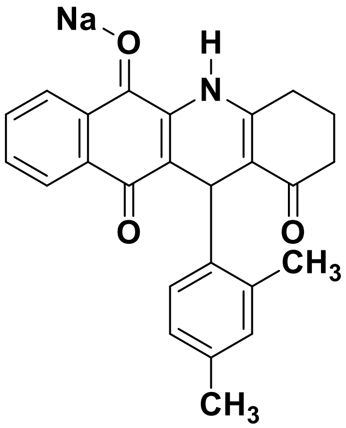

Exact Mass: 406,1419

5i

Meas. m/z	#	Ion Formula	m/z	err [ppm]	Mean err [ppm]	rdb	N-Rule	e⁻ Conf	mSigma	Std I	Std Mean m/z	Std I VarNorm	Std m/z Diff	Std Comb Dev
384.159038	1	C25H22NO3	384.159420	1.0	2.0	15.5	ok	even	3.1	4.5	n.a.	n.a.	n.a.	n.a.
	2	C21H18N7O	384.156735	-6.0	-5.8	16.5	ok	even	11.4	19.5	n.a.	n.a.	n.a.	n.a.
	3	C20H22N3O5	384.155397	-9.5	-9.0	11.5	ok	even	23.8	37.8	n.a.	n.a.	n.a.	n.a.
406.141135	1	C25H21NNaO3	406.141364	0.6	1.3	15.5	ok	even	4.7	7.2	n.a.	n.a.	n.a.	n.a.
	2	C21H17N7NaO	406.138679	-6.0	-6.1	16.5	ok	even	9.7	16.8	n.a.	n.a.	n.a.	n.a.
	3	C20H21N3NaO5	406.137341	-9.3	-9.0	11.5	ok	even	22.2	35.1	n.a.	n.a.	n.a.	n.a.

HRMS of compound **5i**

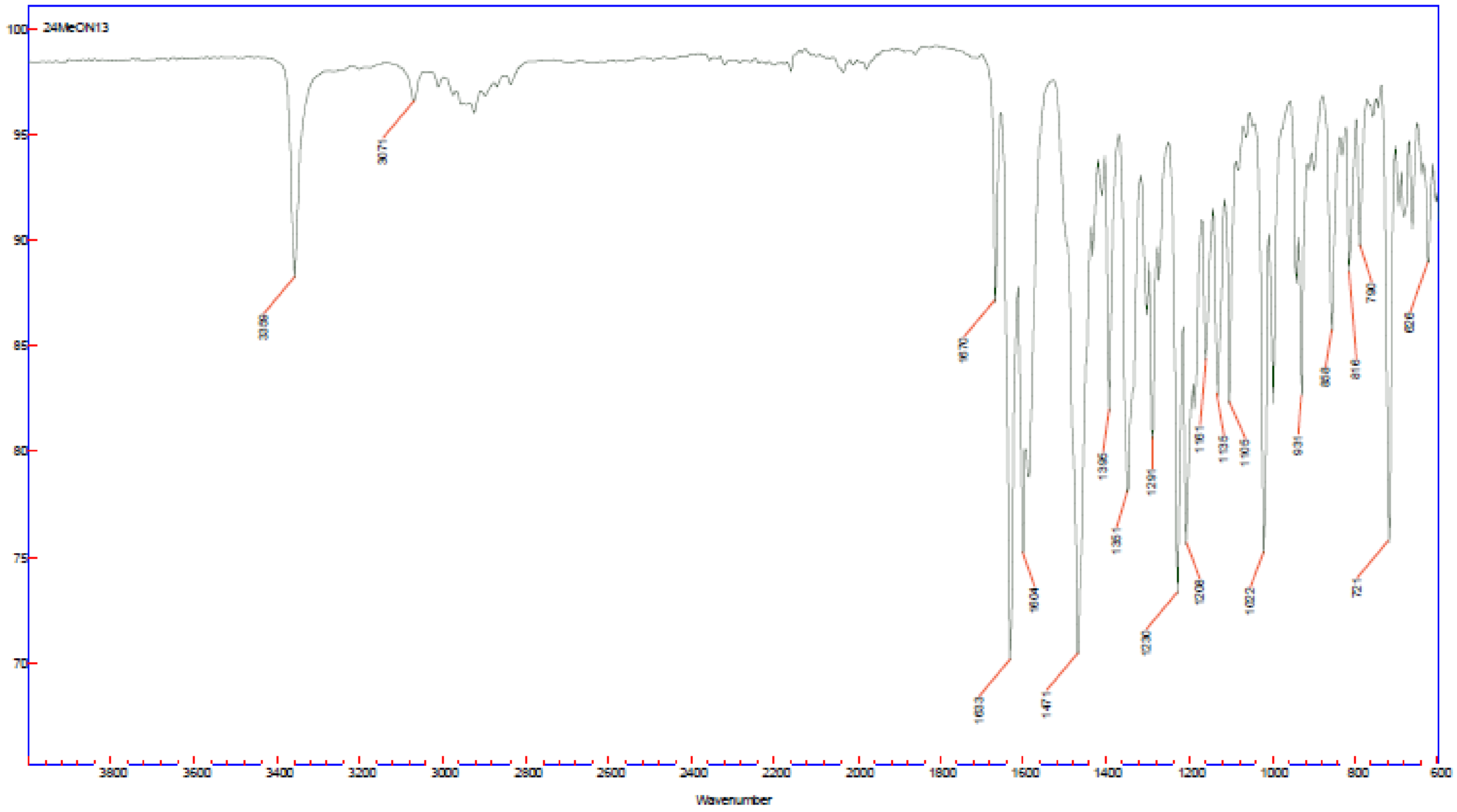

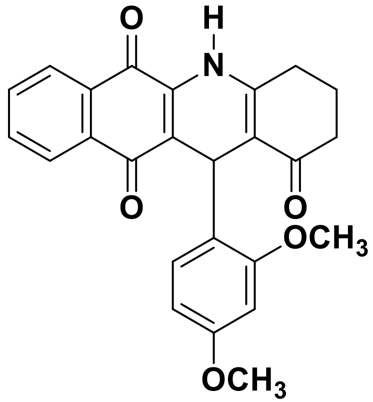

5j

IR spectrum of compound **5j**

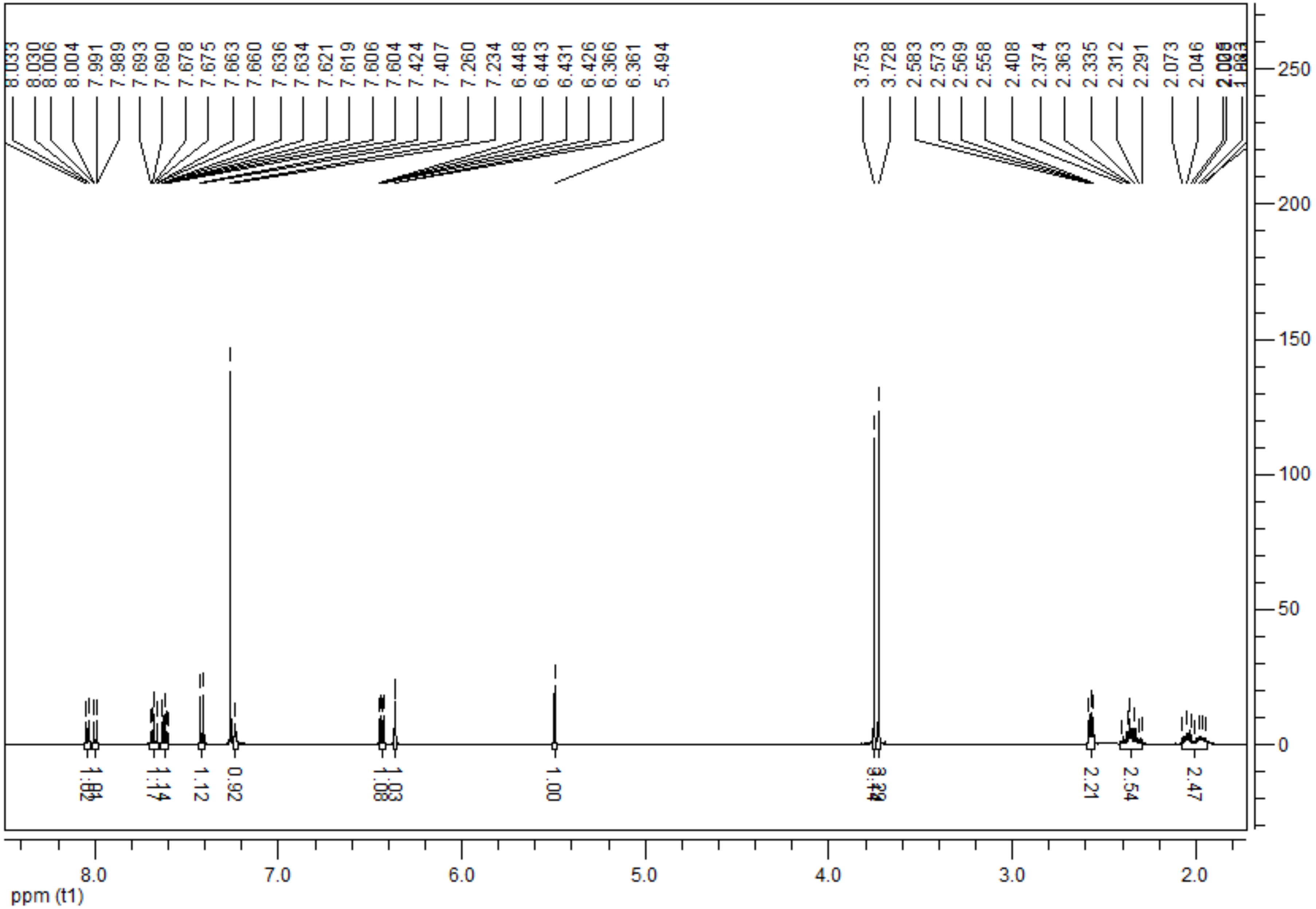

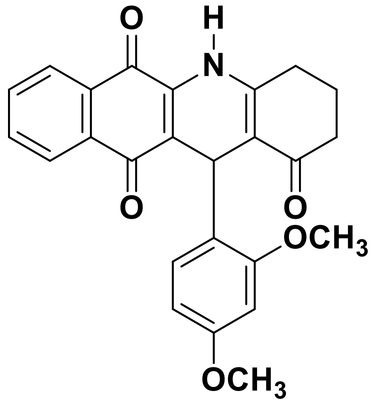

5j

^{1}H NMR spectrum of compound **5j** (500.00 MHz, $CDCl_3$)

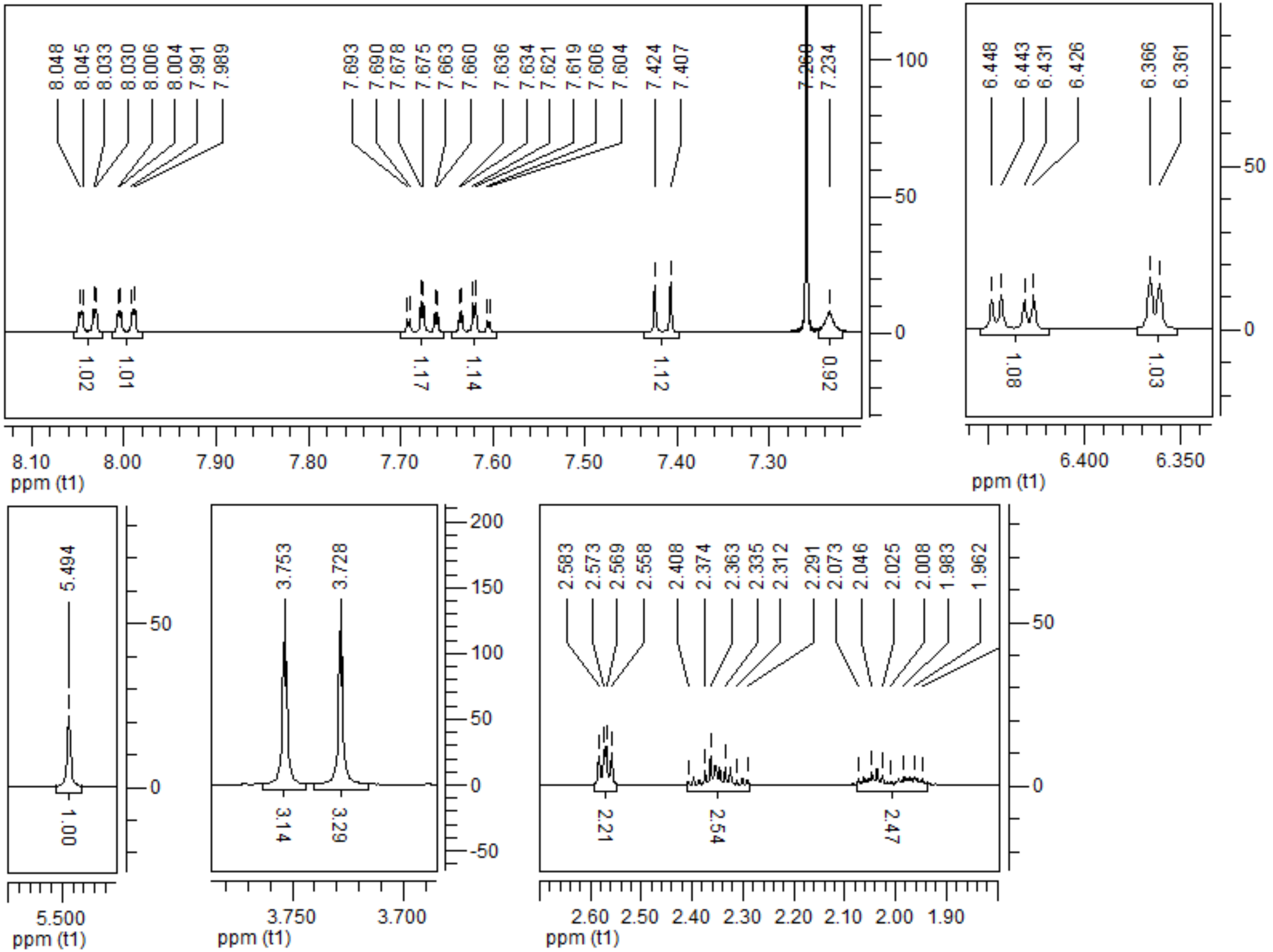

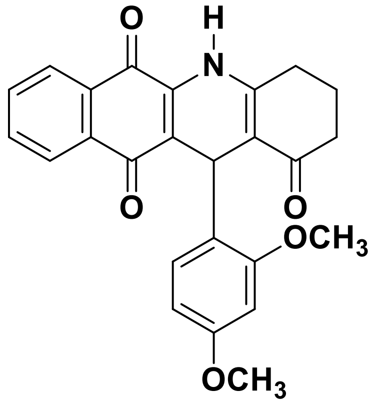

5j

^{1}H NMR spectrum of compound **5j** (500.00 MHz, $CDCl_3$)

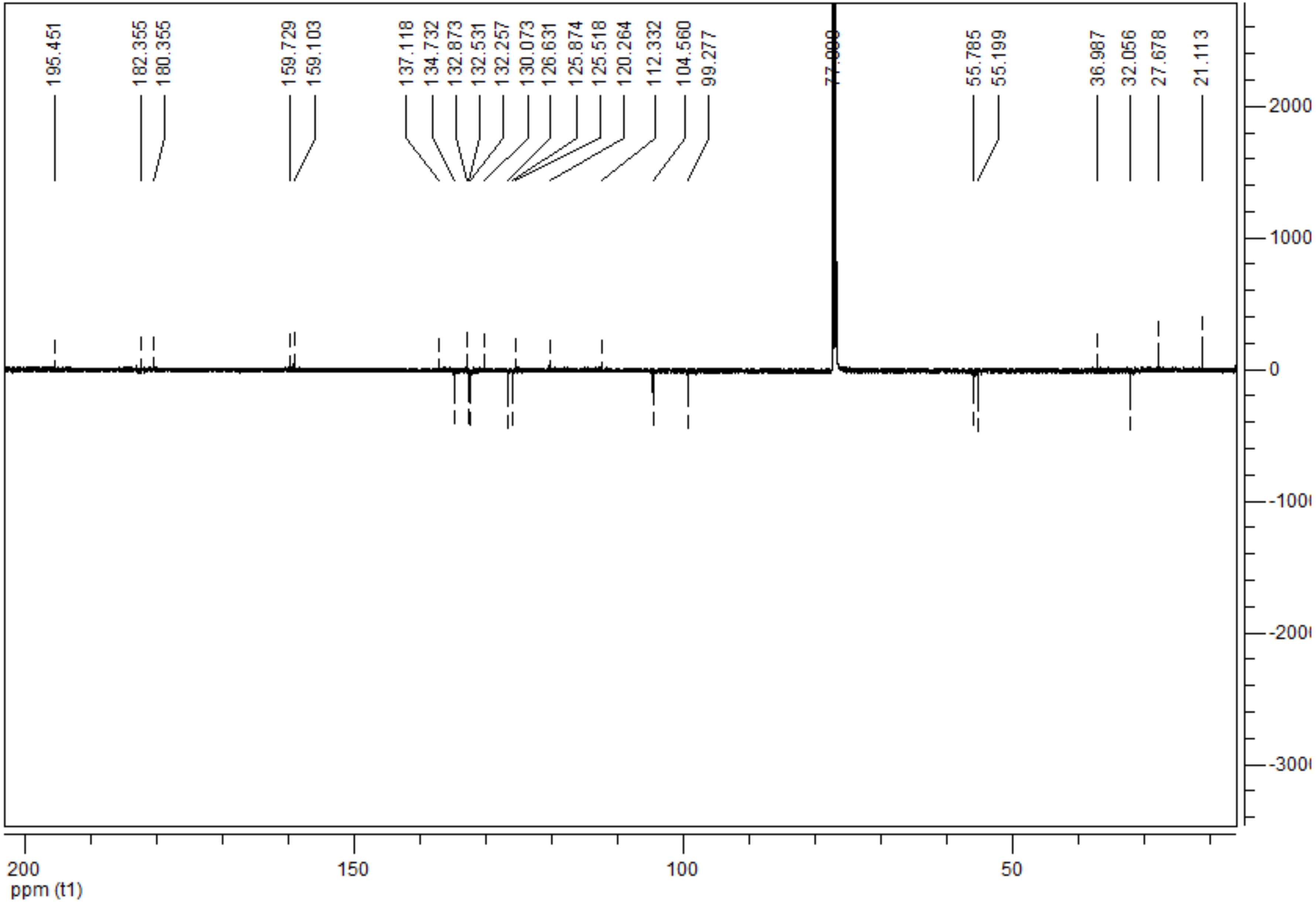

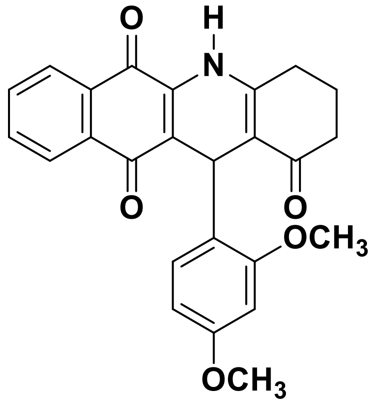

5j

^{13}C NMR spectrum of compound **5j** (75.0 MHz, $CDCl_3$)

+MS, 0.5-0.6min #28-37

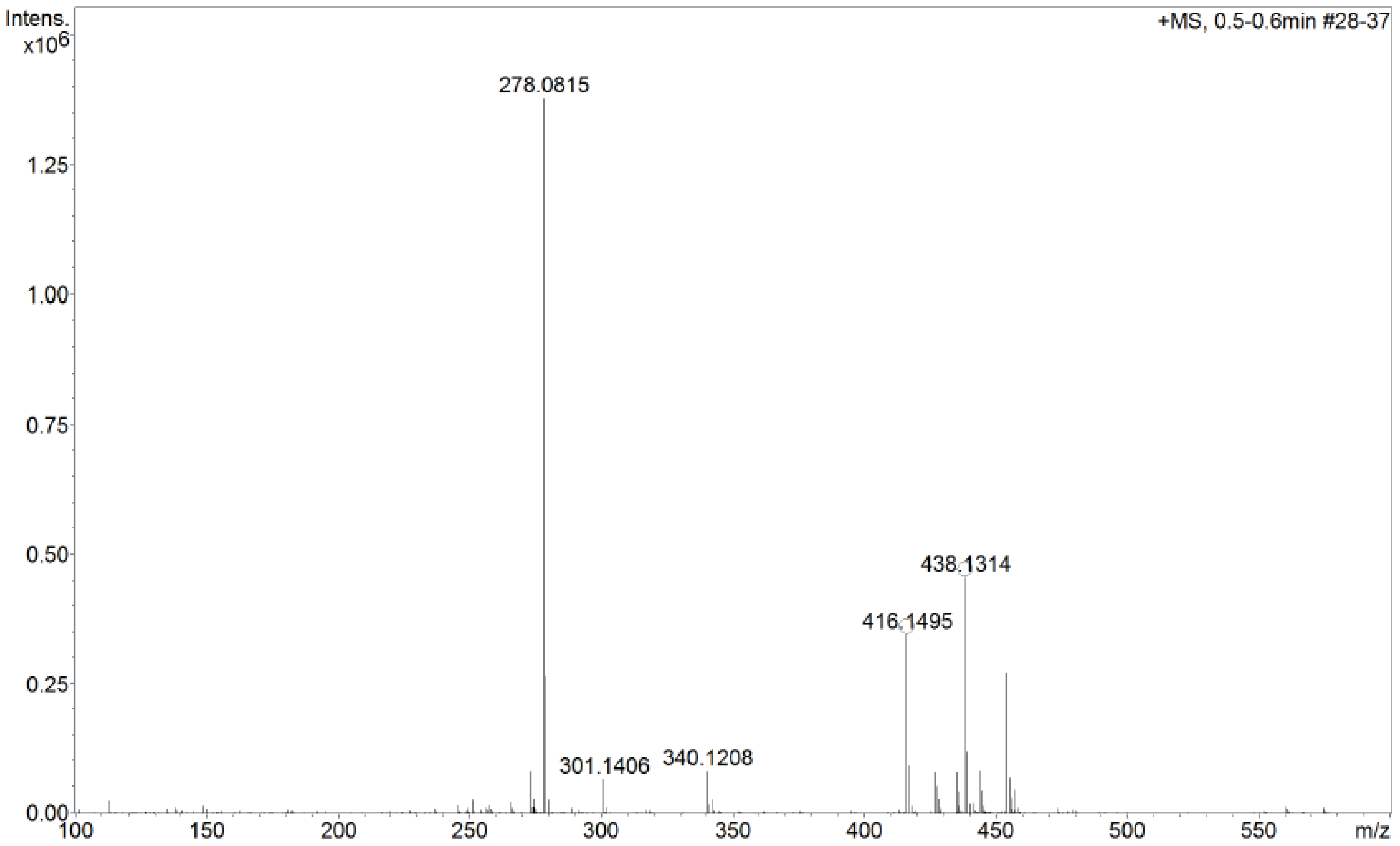

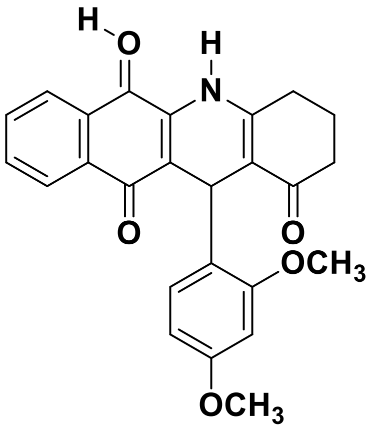

Exact Mass: 416,1498

5j

Meas. m/z	#	Ion Formula	m/z	err [ppm]	Mean err [ppm]	rdb	N-Rule	e⁻ Conf	mSigma	Std I	Std Mean m/z	Std I VarNorm	Std m/z Diff	Std Comb Dev
416.149467	1	C25H22NO5	416.149249	-0.5	0.1	15.5	ok	even	8.4	13.8	n.a.	n.a.	n.a.	n.a.
	2	C26H18N5O	416.150587	2.7	3.1	20.5	ok	even	21.3	31.5	n.a.	n.a.	n.a.	n.a.
438.131436	1	C25H21NNaO5	438.131193	-0.6	0.3	15.5	ok	even	11.7	19.3	n.a.	n.a.	n.a.	n.a.
	2	C26H17N5NaO	438.132531	2.5	3.1	20.5	ok	even	24.4	36.9	n.a.	n.a.	n.a.	n.a.

HRMS of compound **5j**

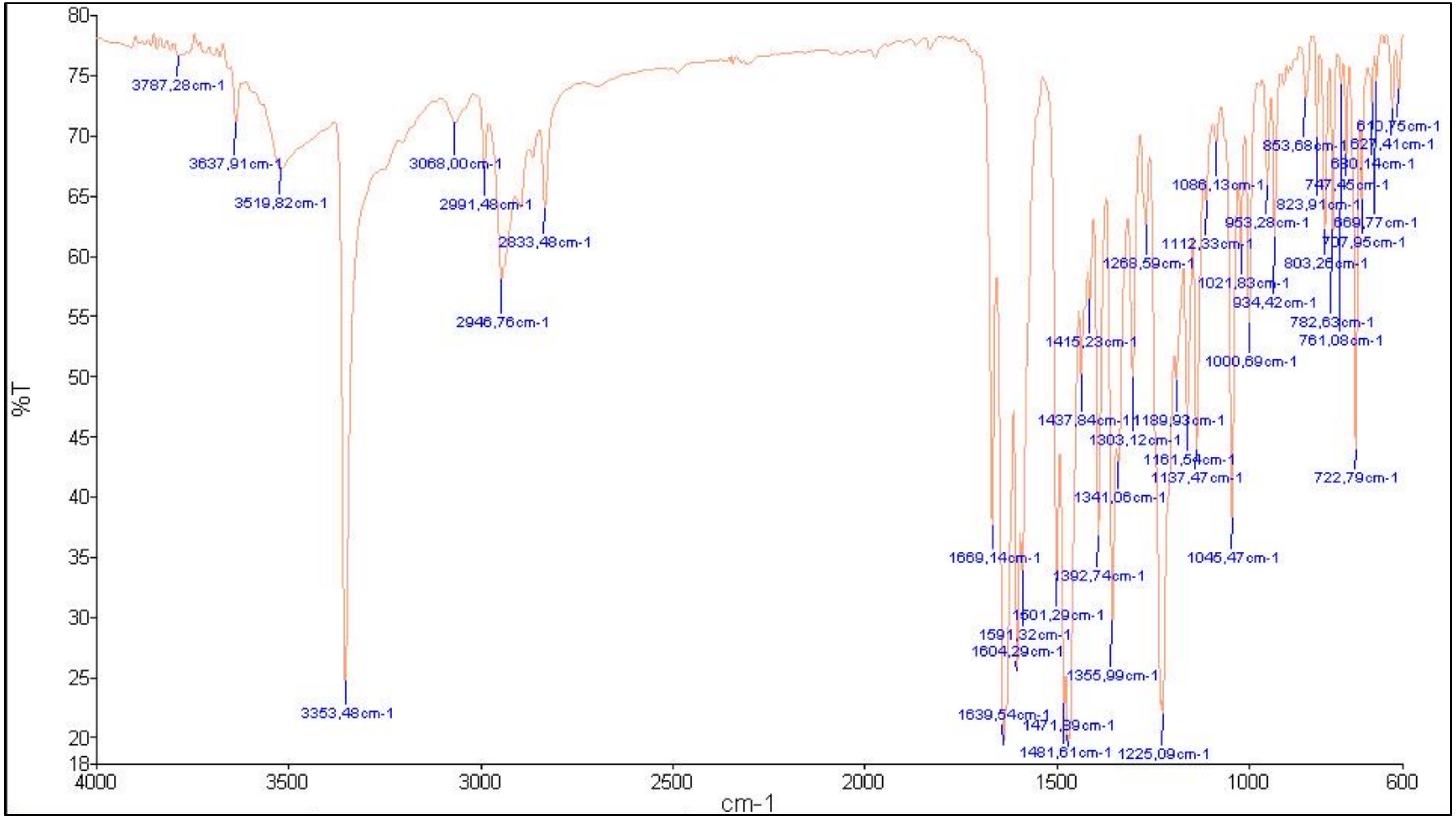

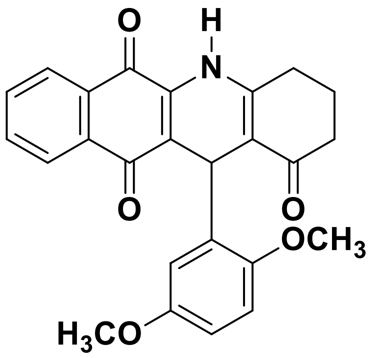

IR spectrum of compound **5k**

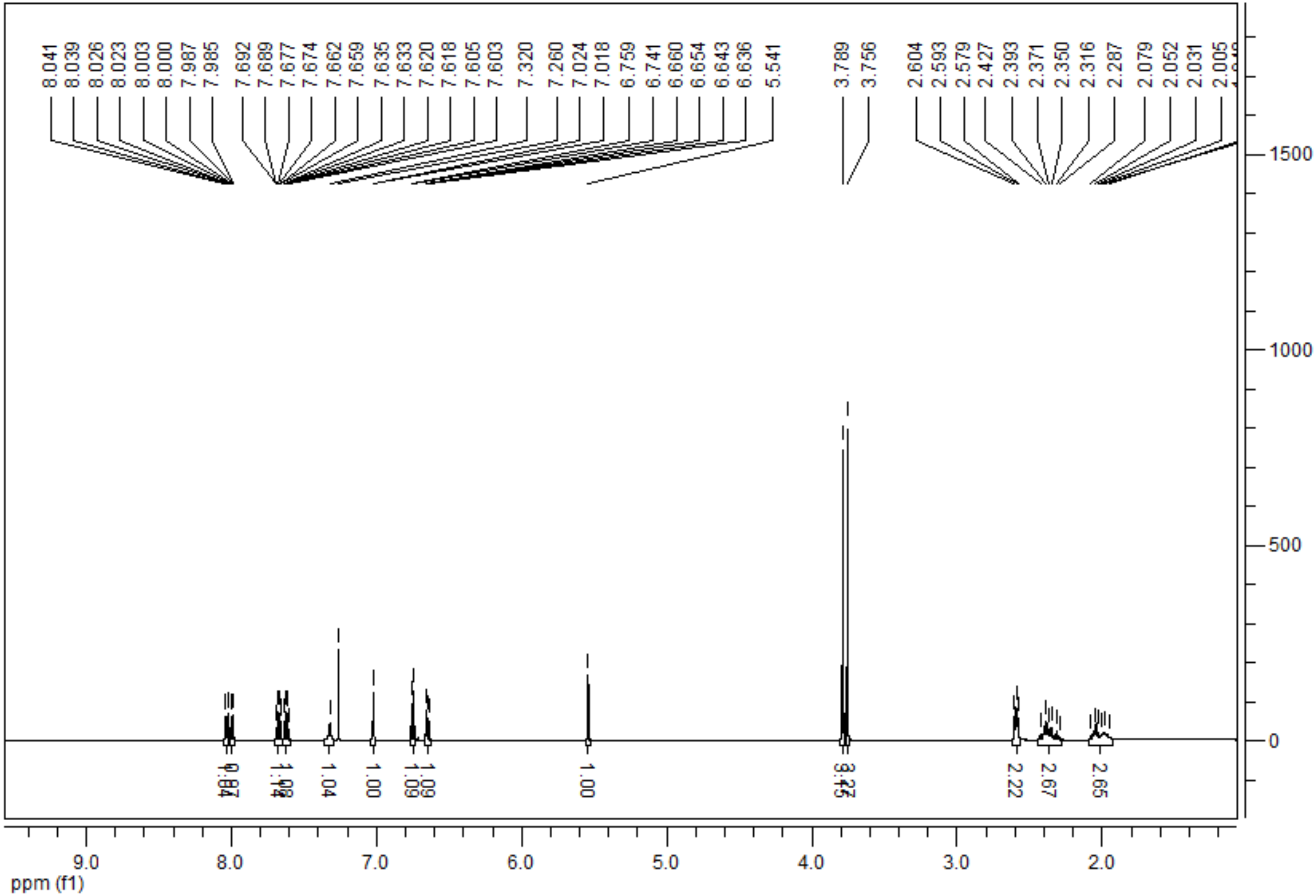

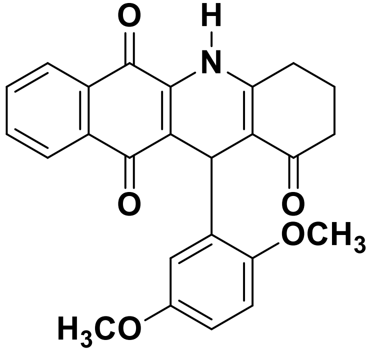

5k

^{1}H NMR spectrum of compound **5k** (500.00 MHz, $CDCl_3$)

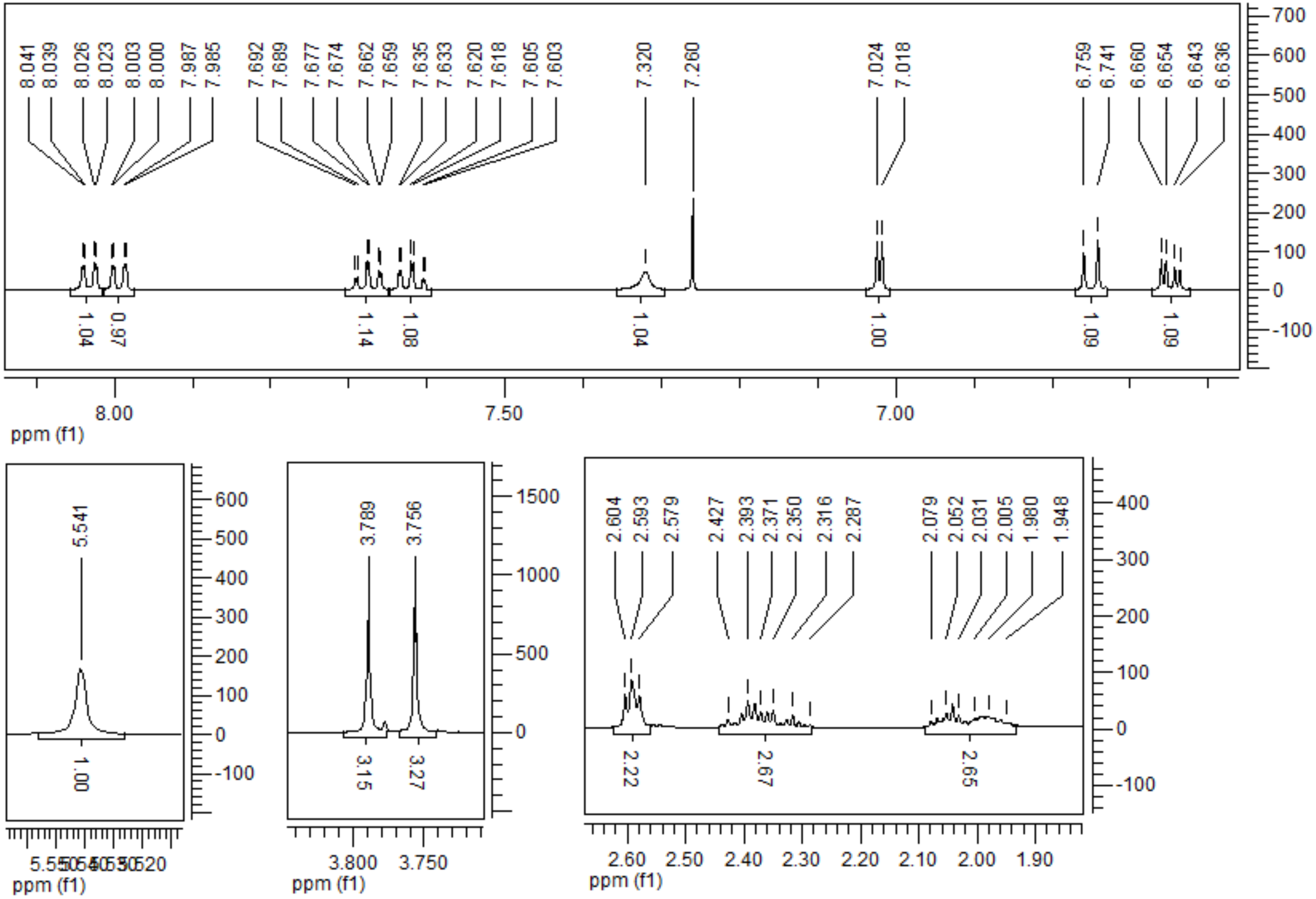

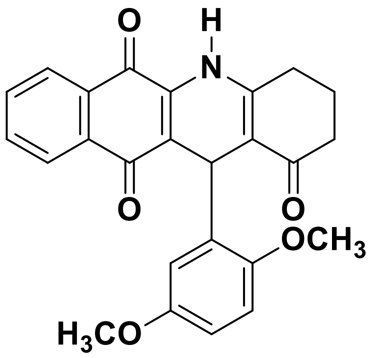

5k

^{1}H NMR spectrum of compound **5k** (500.00 MHz, $CDCl_3$)

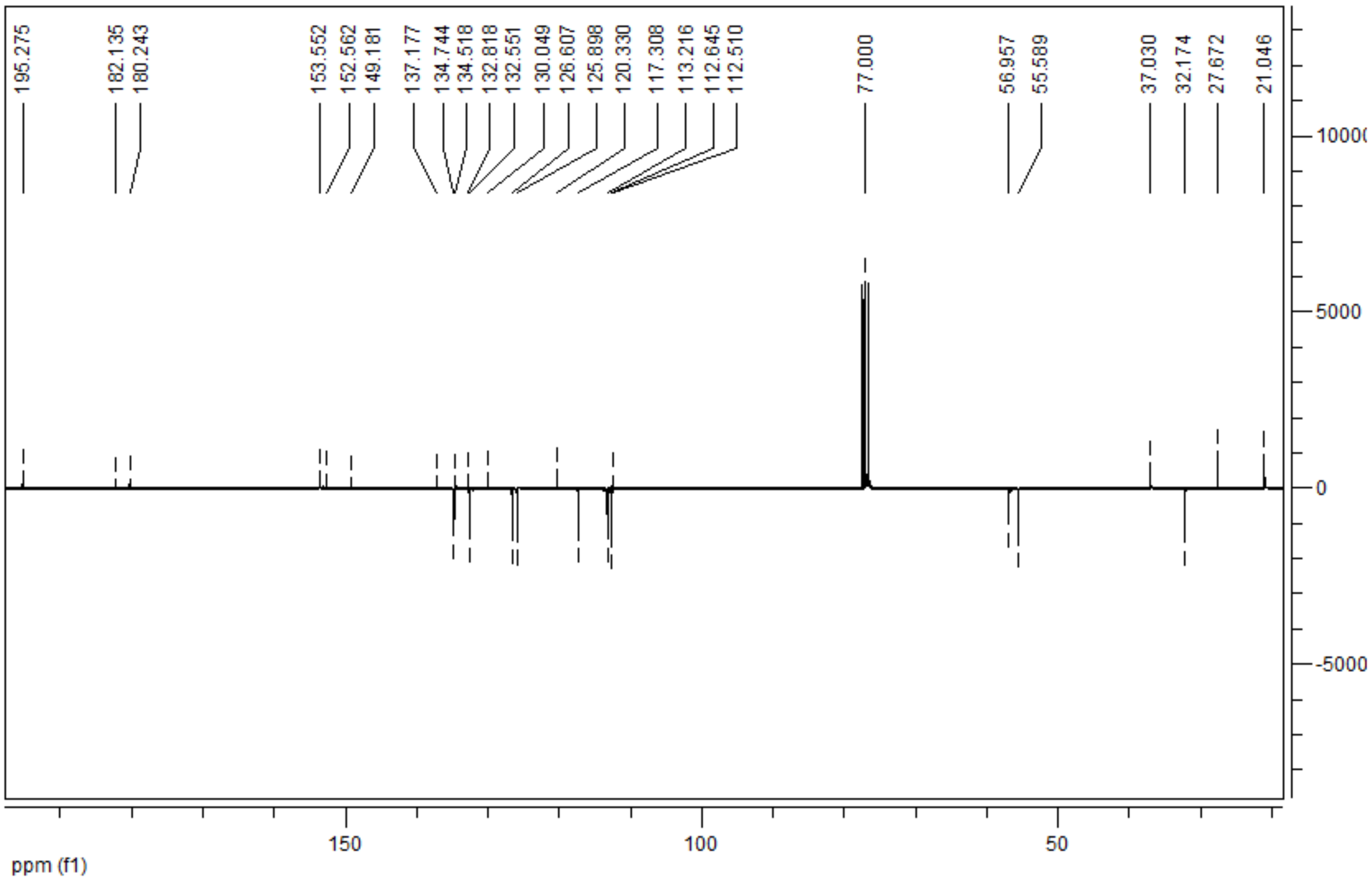

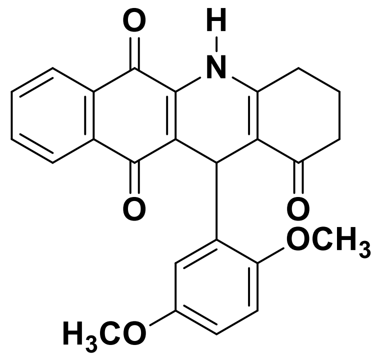

5k

^{13}C NMR spectrum of compound **5k** (125.0 MHz, $CDCl_3$)

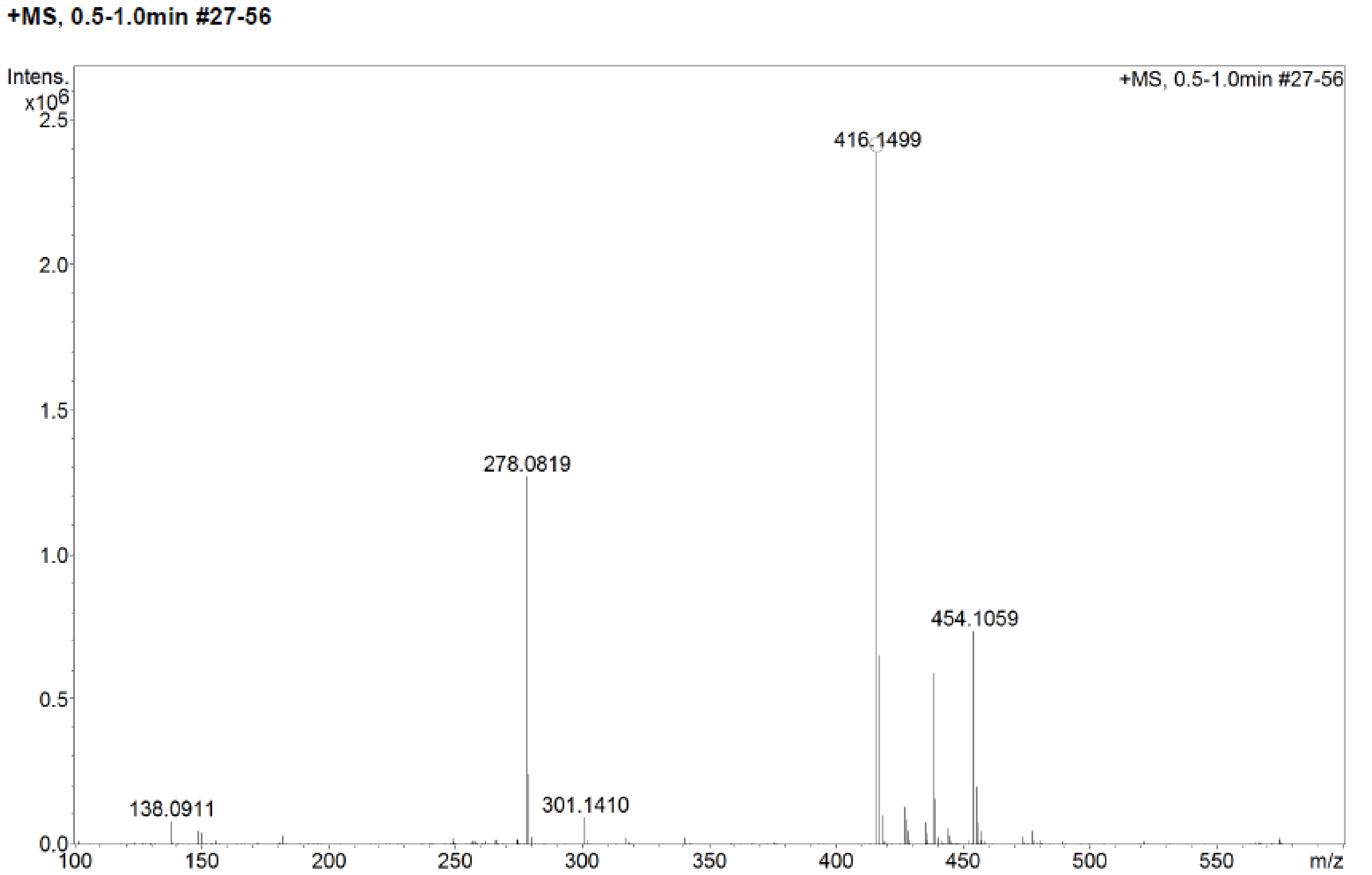

Meas. m/z	#	Ion Formula	m/z	err [ppm]	Mean err [ppm]	rdb	N-Rule	e⁻ Conf	mSigma	Std I	Std Mean m/z	Std I VarNorm	Std m/z Diff	Std Comb Dev
416.149912	1	C25H22NO5	416.149249	-1.6	-1.8	15.5	ok	even	4.0	7.1	n.a.	n.a.	n.a.	n.a.
	2	C21H18N7O3	416.146564	-8.0	-9.0	16.5	ok	even	10.9	17.2	n.a.	n.a.	n.a.	n.a.
	3	C26H18N5O	416.150587	1.6	1.1	20.5	ok	even	16.1	23.8	n.a.	n.a.	n.a.	n.a.

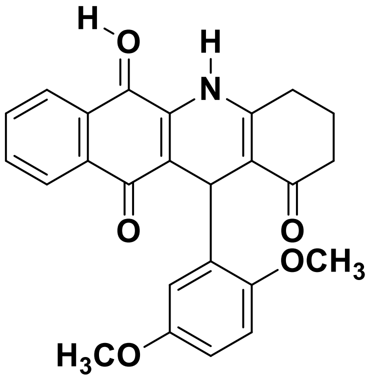

Exact Mass: 416,1498

5k

HRMS of compound **5k**

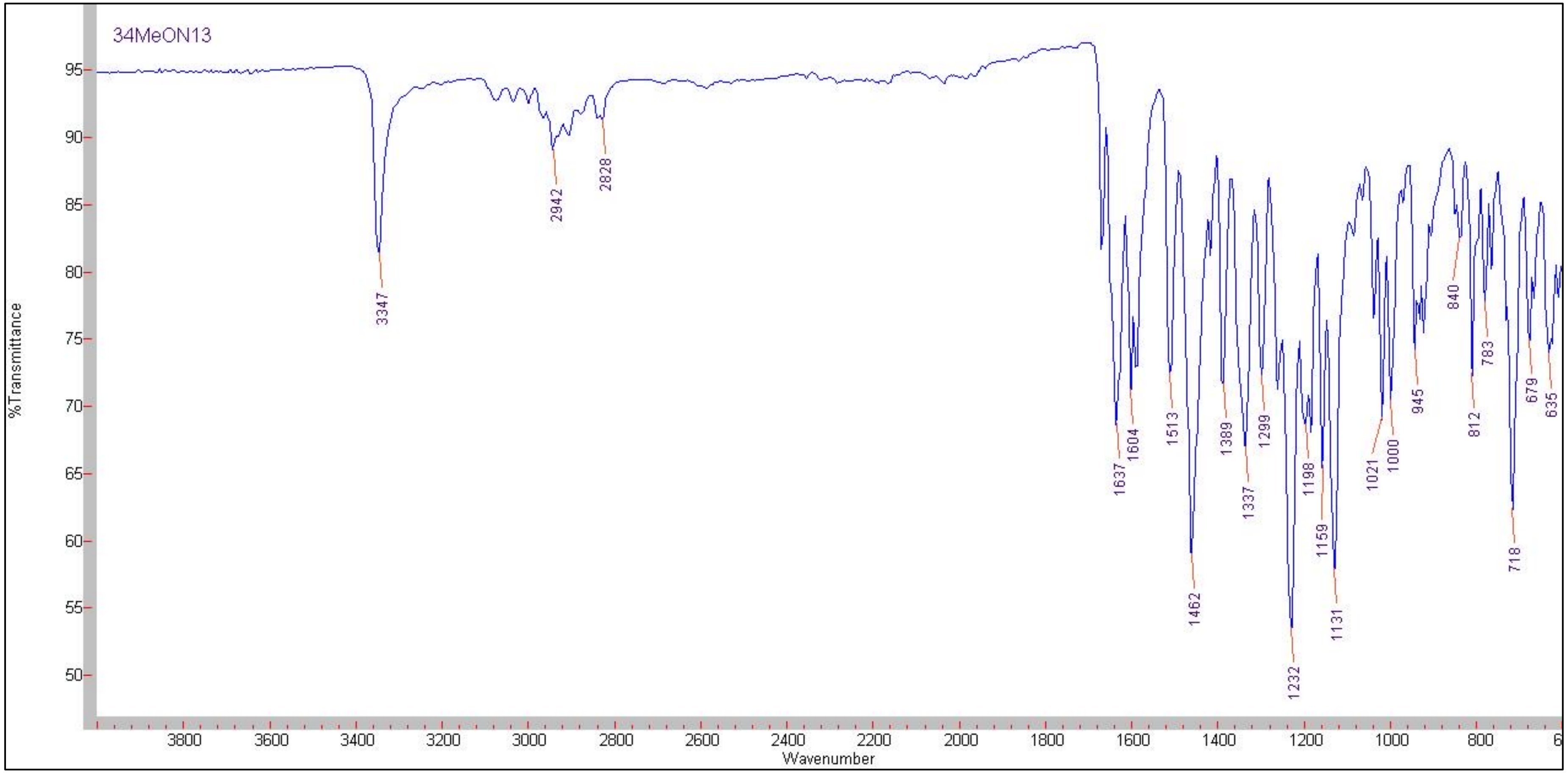

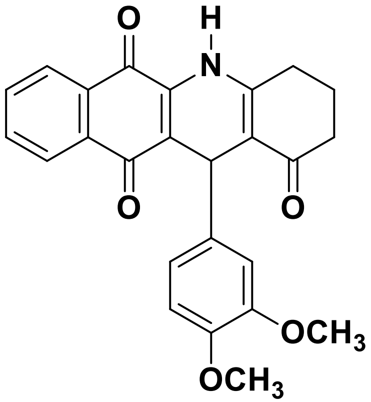

5l

IR spectrum of compound **5l**

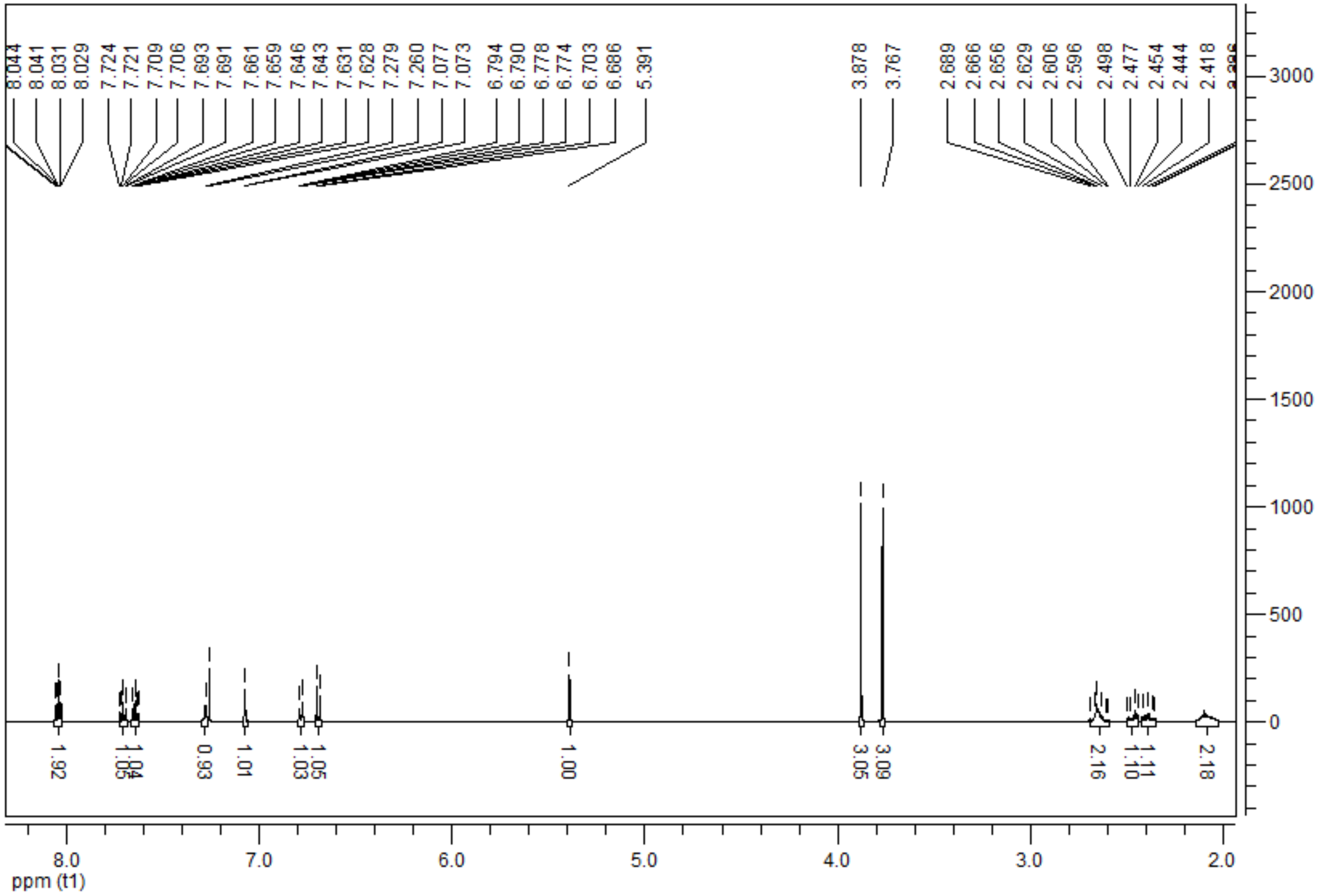

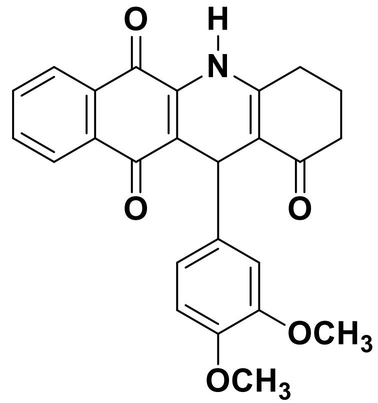

5l

^{1}H NMR spectrum of compound **5l** (500.00 MHz, $CDCl_3$)

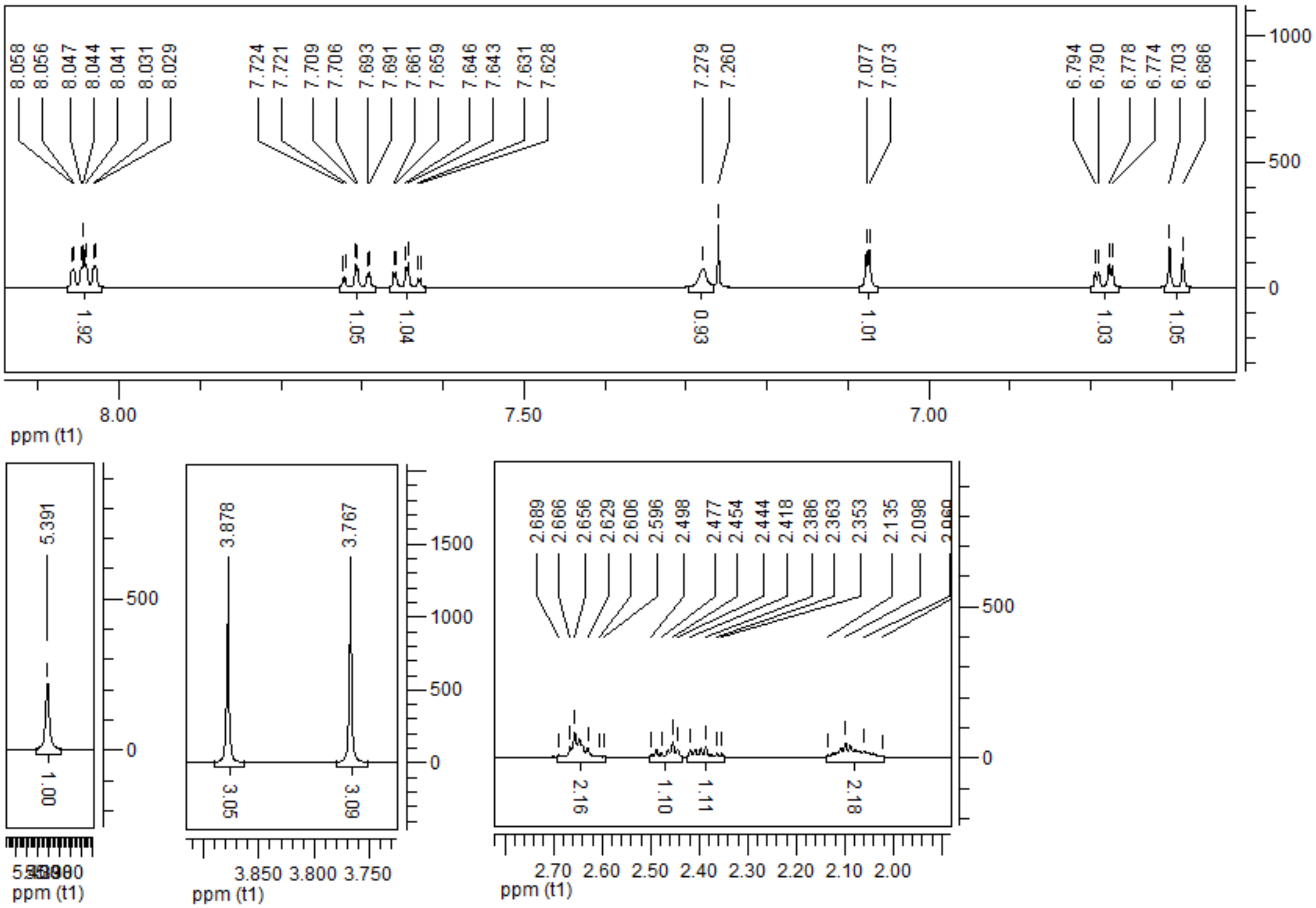

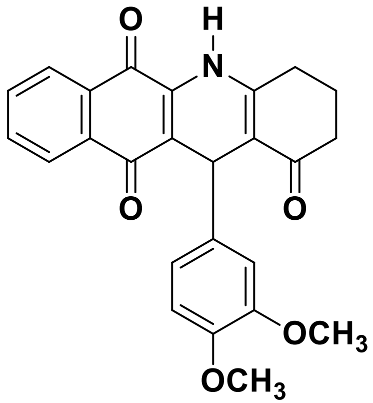

5l

^{1}H NMR spectrum of compound **5l** (500.00 MHz, $CDCl_3$)

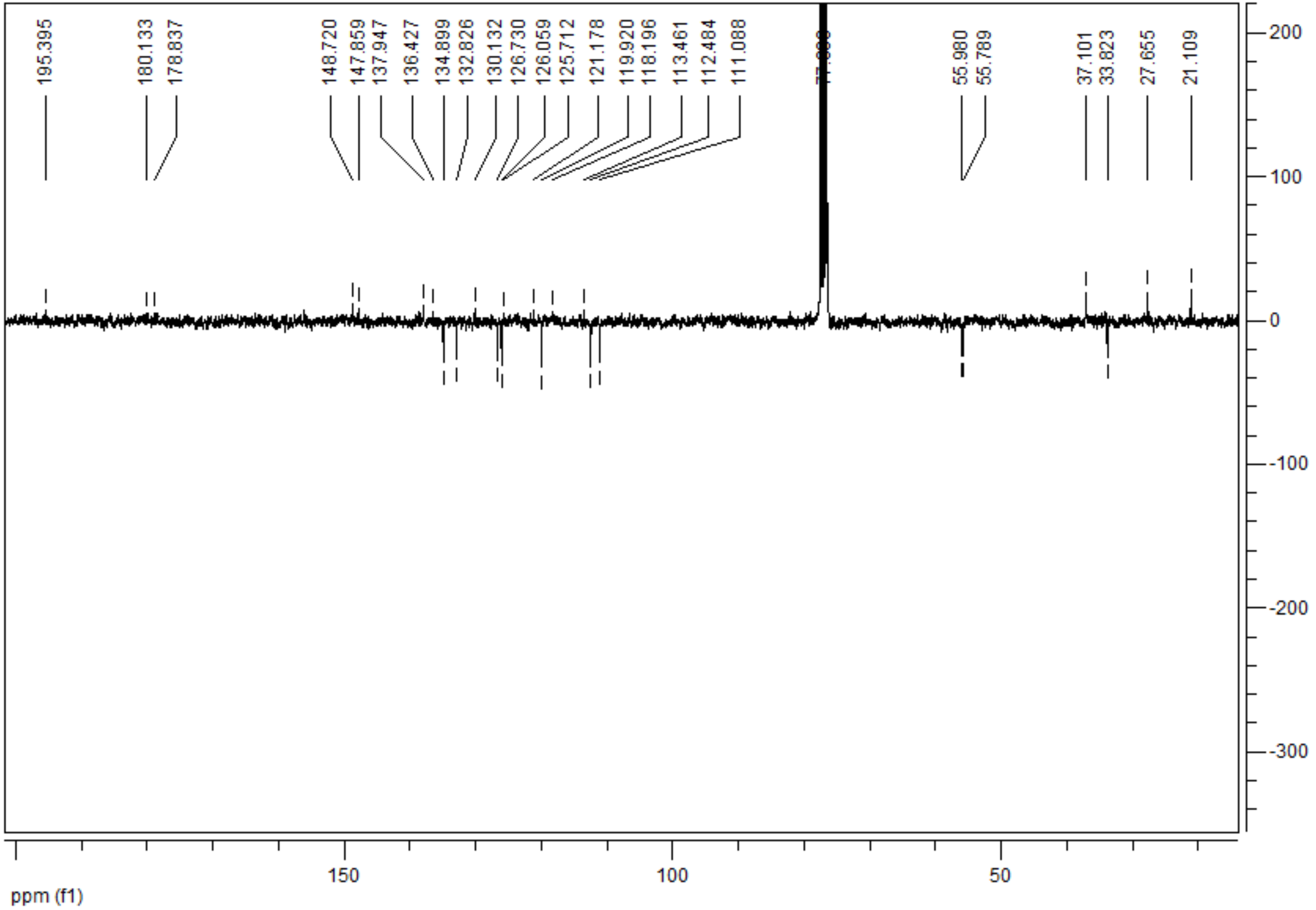

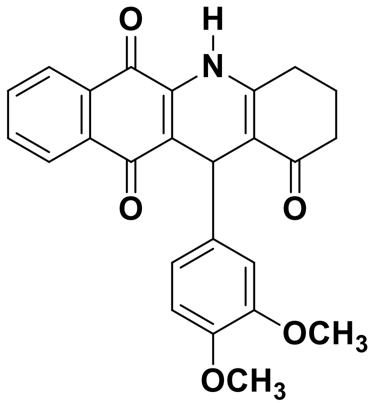

5l

^{13}C NMR spectrum of compound **5l** (75.0 MHz, $CDCl_3$)

+MS, 0.1-0.8min #7-47

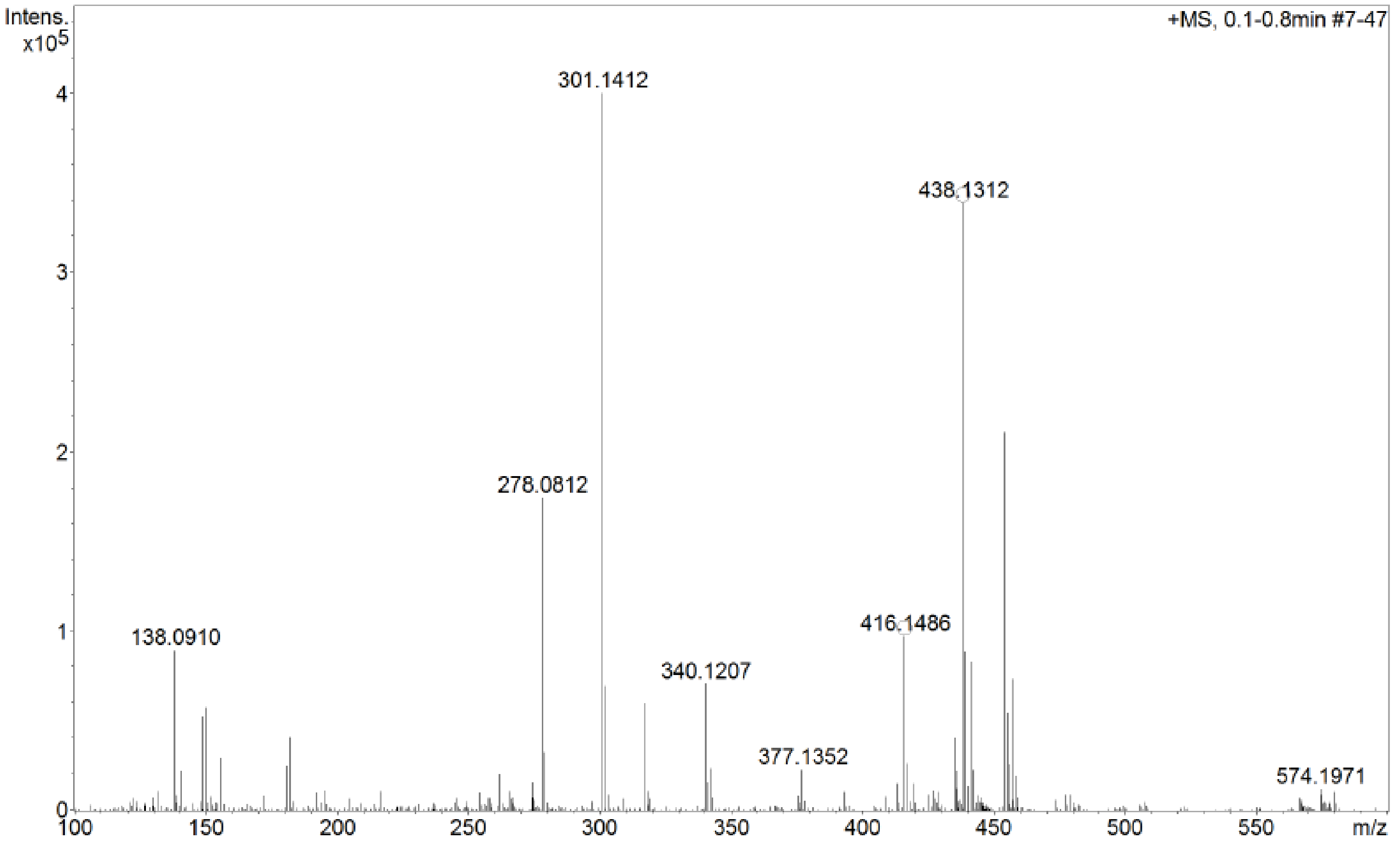

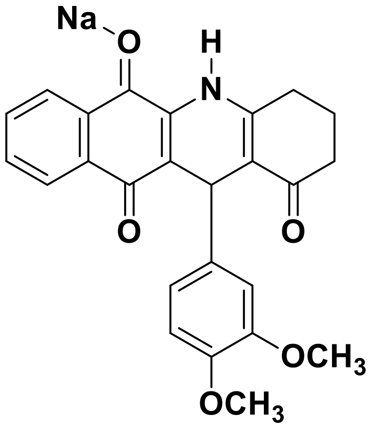

Exact Mass: 438,1317

5l

Meas. m/z	#	Ion Formula	m/z	err [ppm]	Mean err [ppm]	rdb	N-Rule	e⁻ Conf	mSigma	Std I	Std Mean m/z	Std I VarNorm	Std m/z Diff	Std Comb Dev
416.148636	1	C25H22NO5	416.149249	1.5	1.2	15.5	ok	even	8.6	11.5	n.a.	n.a.	n.a.	n.a.
	2	C21H18N7O3	416.146564	-5.0	-6.0	16.5	ok	even	9.2	16.4	n.a.	n.a.	n.a.	n.a.
	3	C20H22N3O7	416.145226	-8.2	-8.9	11.5	ok	even	19.3	32.1	n.a.	n.a.	n.a.	n.a.
	4	C26H18N5O	416.150587	4.7	4.1	20.5	ok	even	22.1	30.0	n.a.	n.a.	n.a.	n.a.
438.131237	1	C25H21NNaO5	438.131193	-0.1	0.6	15.5	ok	even	10.3	16.9	n.a.	n.a.	n.a.	n.a.
	2	C26H17N5NaO	438.132531	3.0	3.3	20.5	ok	even	23.2	34.6	n.a.	n.a.	n.a.	n.a.

HRMS of compound **5l**

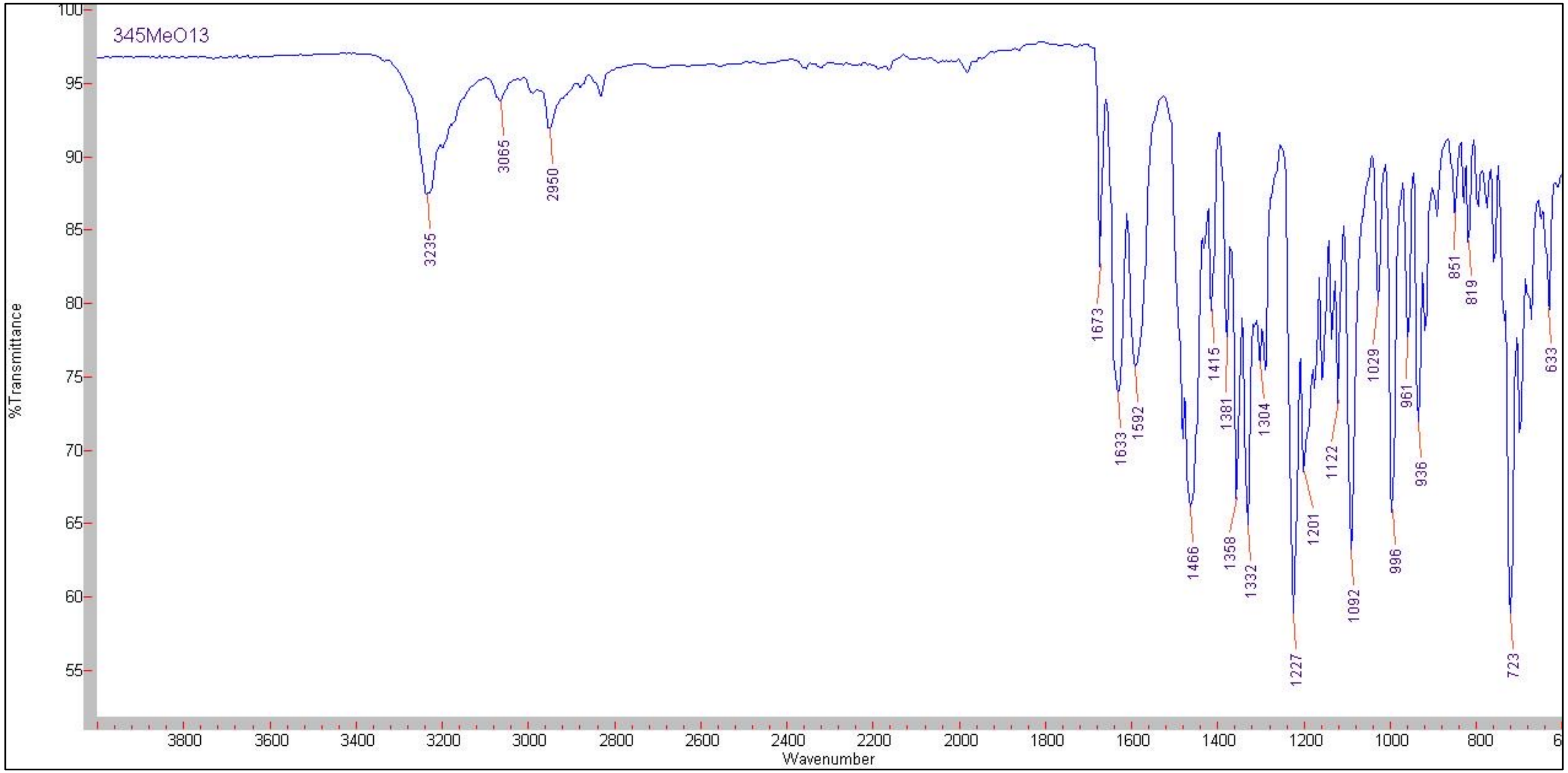

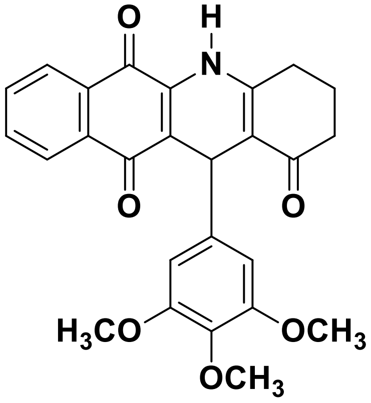

5m

IR spectrum of compound **5m**

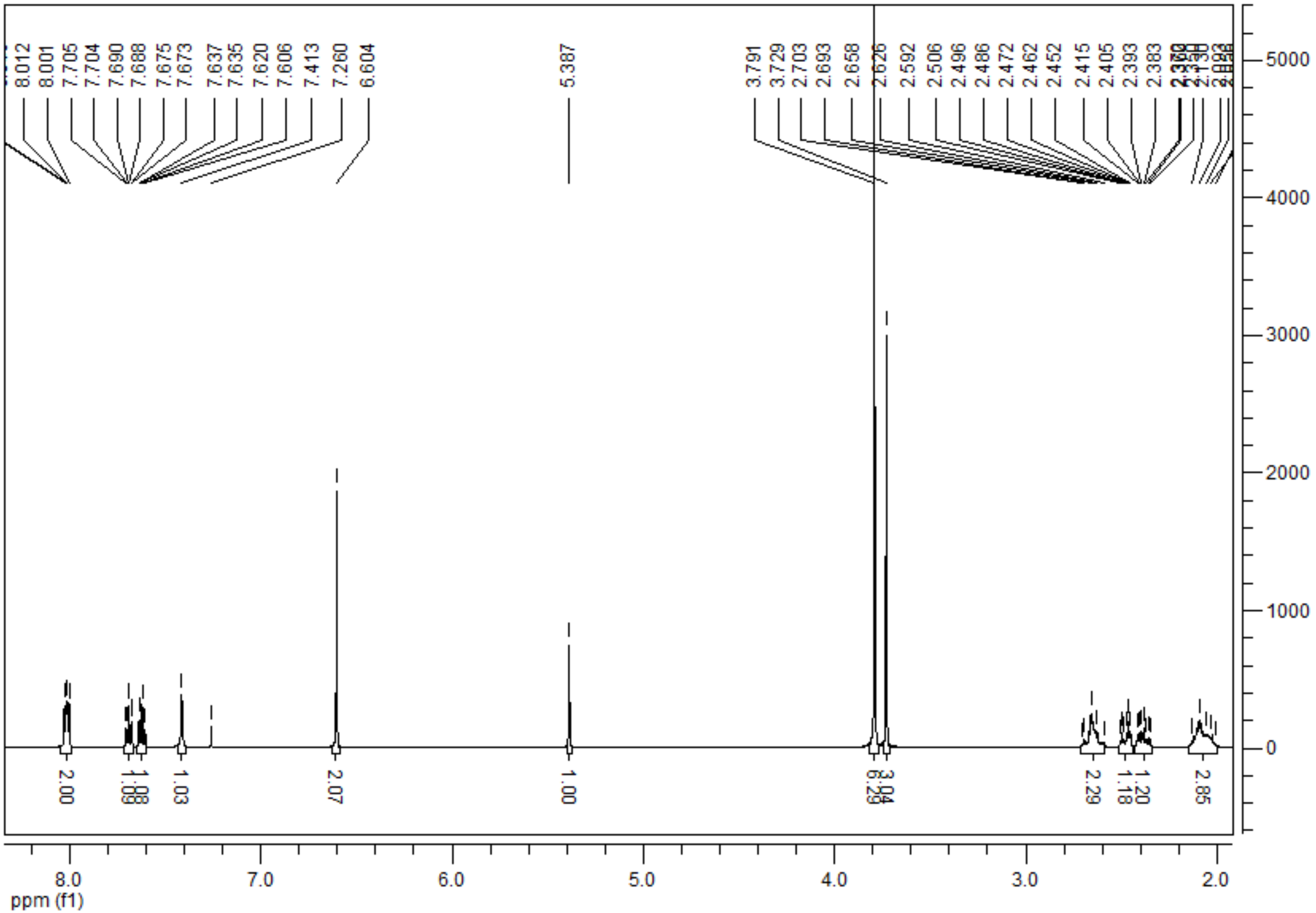

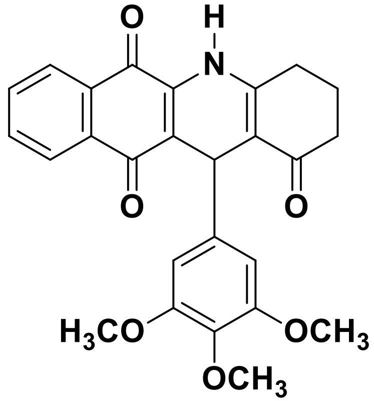

5m

^{1}H NMR spectrum of compound **5m** (500.00 MHz, $CDCl_3$)

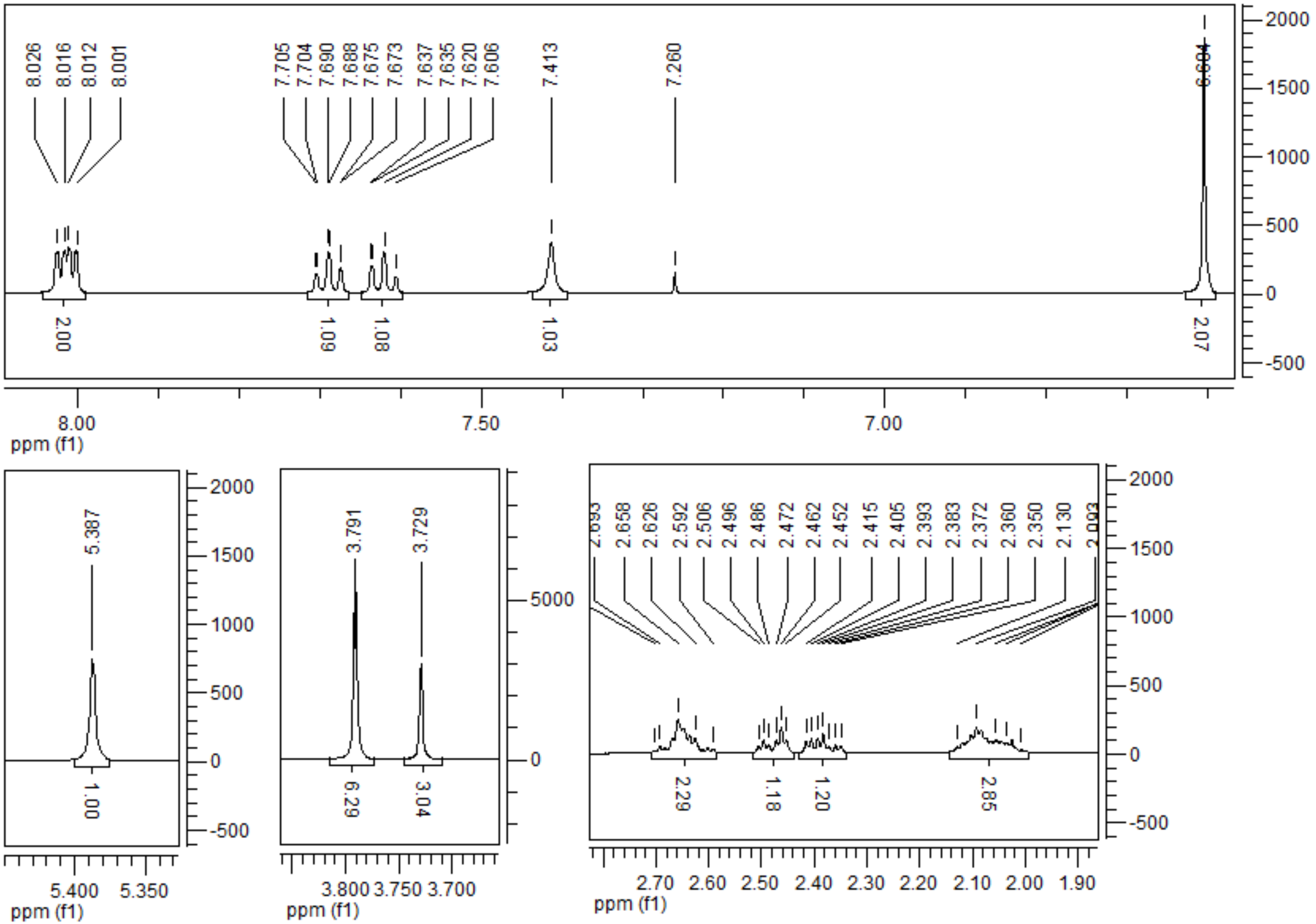

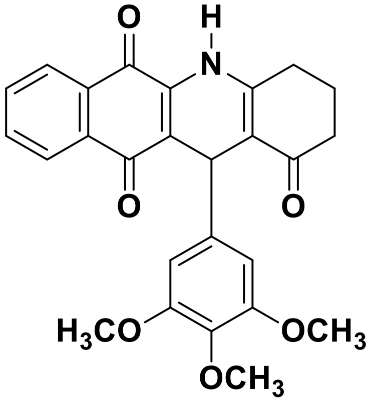

5m

^{1}H NMR spectrum of compound **5m** (500.00 MHz, $CDCl_3$)

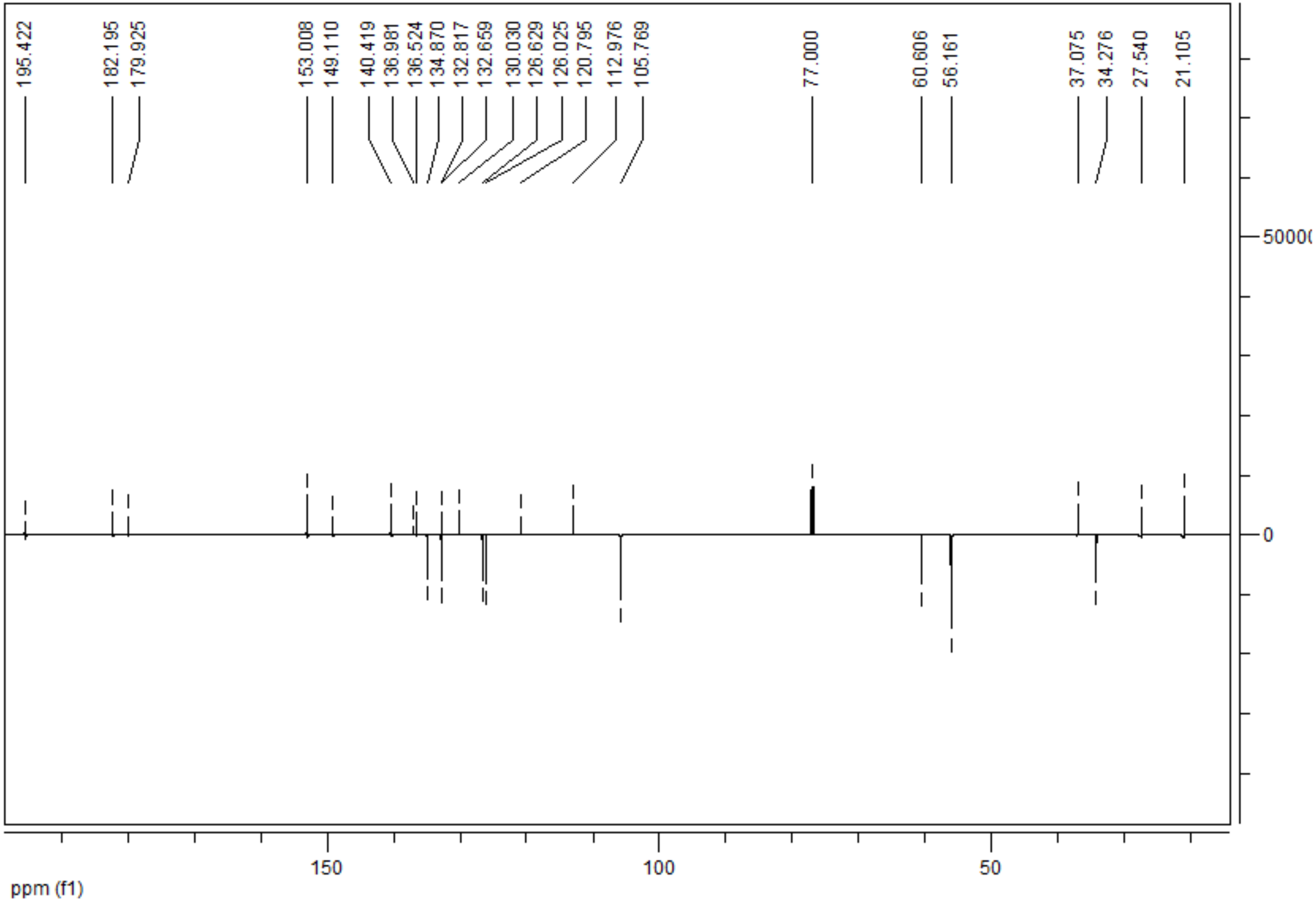

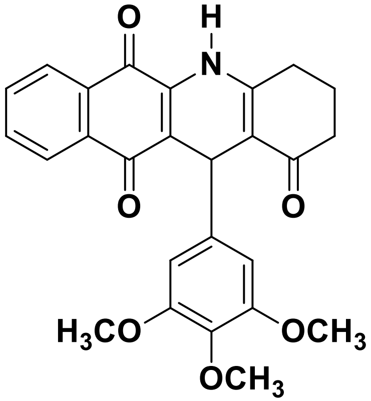

5m

^{13}C NMR spectrum of compound **5m** (75.0 MHz, $CDCl_3$)

+MS, 0.3-0.7min #16-43

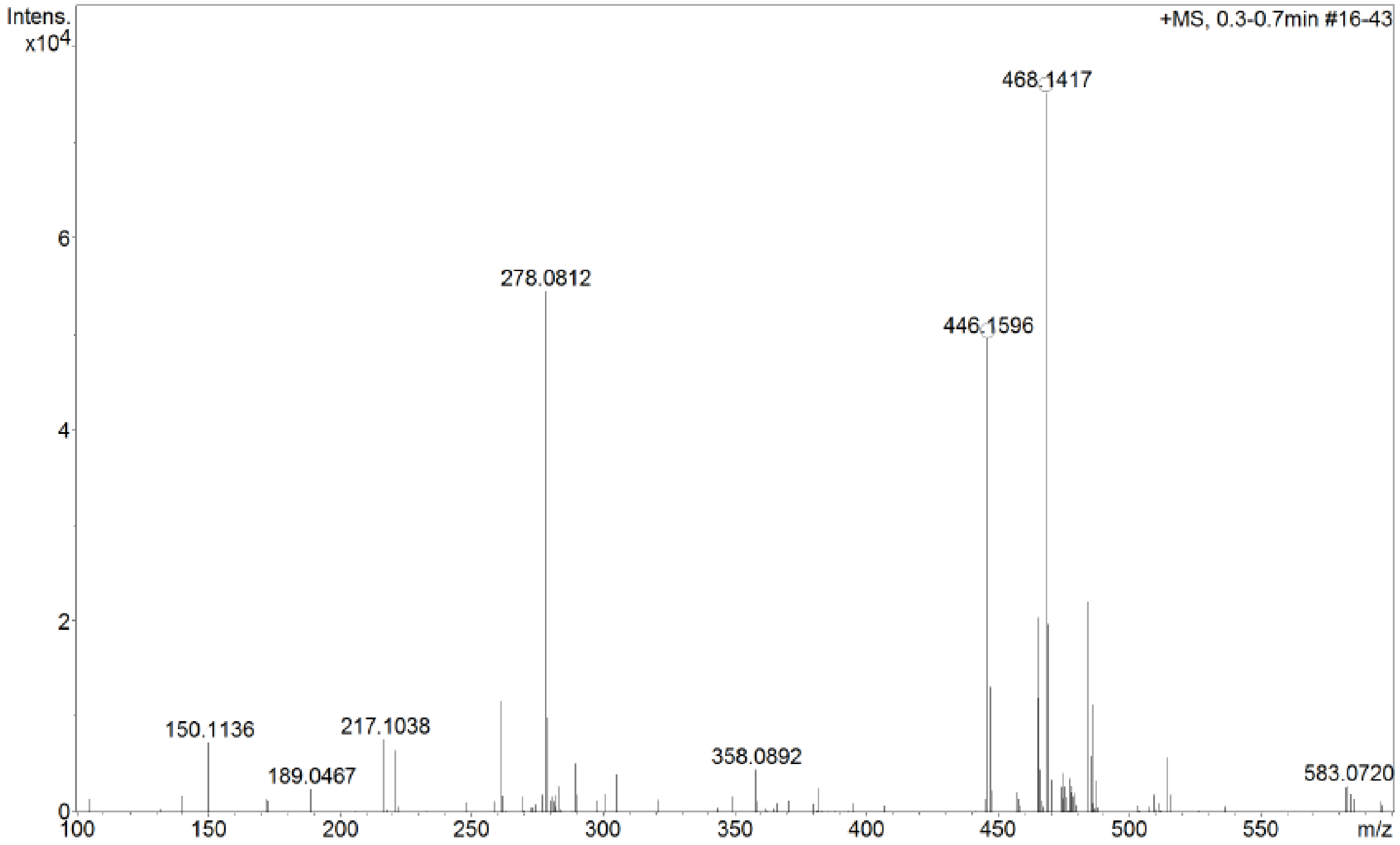

Meas. m/z	#	Ion Formula	m/z	err [ppm]	Mean err [ppm]	rdb	N-Rule	e⁻ Conf	mSigma	Std I	Std Mean m/z	Std I VarNorm	Std m/z Diff	Std Comb Dev
446.159604	1	C26H24NO6	446.159814	0.5	1108.5	15.5	ok	even	14.0	27.5	n.a.	n.a.	n.a.	n.a.
	2	C23H16N11	446.158466	-2.6	-2.7	21.5	ok	even	15.6	21.4	n.a.	n.a.	n.a.	n.a.
	3	C27H20N5O2	446.161151	3.5	4.1	20.5	ok	even	28.7	42.0	n.a.	n.a.	n.a.	n.a.
468.141660	1	C26H23NNaO6	468.141758	0.2	1056.4	15.5	ok	even	14.9	29.0	n.a.	n.a.	n.a.	n.a.
	2	C23H15N11Na	468.140410	-2.7	-2.6	21.5	ok	even	16.8	23.1	n.a.	n.a.	n.a.	n.a.
	3	C27H19N5NaO2	468.143096	3.1	3.8	20.5	ok	even	29.8	43.6	n.a.	n.a.	n.a.	n.a.

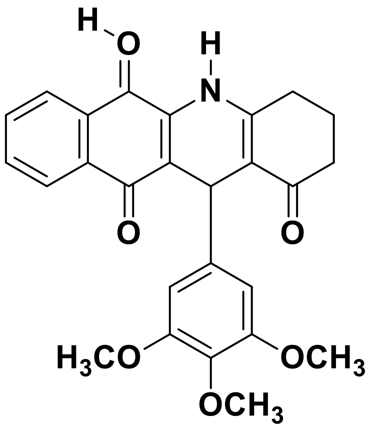

Exact Mass: 446,1604

5m

HRMS of compound **5m**
